# Revisions to the Safety Assurance Factors for Electronic Health Record Resilience (SAFER) Guides to update national recommendations for safe use of electronic health records

**DOI:** 10.1093/jamia/ocaf018

**Published:** 2025-04-12

**Authors:** Dean F Sittig, Trisha Flanagan, Patricia Sengstack, Rosann T Cholankeril, Sara Ehsan, Amanda Heidemann, Daniel R Murphy, Hojjat Salmasian, Jason S Adelman, Hardeep Singh

**Affiliations:** Department of Clinical and Health Informatics, McWilliams School of Biomedical Informatics, University of Texas Health Science Center at Houston, Houston, TX 77030, United States; Informatics Review LLC, Lake Oswego, OR 97034, United States; Center for Innovations in Quality, Effectiveness and Safety, Michael E. DeBakey Veterans Affairs Medical Center, Houston, TX 77030, United States; Vanderbilt University School of Nursing, Nashville, TN 37240, United States; Center for Innovations in Quality, Effectiveness and Safety, Michael E. DeBakey Veterans Affairs Medical Center, Houston, TX 77030, United States; Center for Innovations in Quality, Effectiveness and Safety, Michael E. DeBakey Veterans Affairs Medical Center, Houston, TX 77030, United States; Wolters Kluwer Health, St. Louis, MO 63146, United States; Center for Innovations in Quality, Effectiveness and Safety, Michael E. DeBakey Veterans Affairs Medical Center, Houston, TX 77030, United States; Department of Medicine, Baylor College of Medicine, Houston, TX 77030, United States; Division of General Internal Medicine and Primary Care, Brigham and Women’s Hospital, and Harvard Medical School, Boston, MA 02115, United States; Department of Medicine, Columbia University Irving Medical Center, New York, NY 10032, United States; Department of Quality and Patient Safety, NewYork-Presbyterian Hospital, New York, NY 10032, United States; Center for Innovations in Quality, Effectiveness and Safety, Michael E. DeBakey Veterans Affairs Medical Center, Houston, TX 77030, United States; Department of Medicine, Baylor College of Medicine, Houston, TX 77030, United States

**Keywords:** electronic health records, health policy, safety

## Abstract

The Safety Assurance Factors for Electronic Health Record (EHR) Resilience (SAFER) Guides provide recommendations to healthcare organizations for conducting proactive self-assessments of the safety and effectiveness of their EHR implementation and use. Originally released in 2014, they were last updated in 2016. In 2022, the Centers for Medicare and Medicaid Services required their annual attestation by US hospitals.

**Objectives:**

This case study describes how SAFER Guide recommendations were updated to align with current evidence and clinical practice.

**Materials and Methods:**

Over nine months, a multidisciplinary team updated SAFER Guides through literature reviews, iterative feedback, and online meetings.

**Results:**

We reduced the number of recommended practices across all Guides by 40% and consolidated 9 Guides into 8 to maximize ease of use, feasibility, and utility. We provide a 4-level evidence grading hierarchy for each recommendation and a new 5-point rating scale to self-assess implementation status of the recommendation. We included 429 citations of which 289 (67%) were published since the 2016 revision.

**Discussion:**

SAFER Guides were revised to offer EHR best practices, adaptable to unique organizational needs, with interactive content available at: https://www.healthit.gov/topic/safety/safer-guides.

**Conclusion:**

Revisions ensure that the 2025 SAFER Guides represent the best available current evidence for EHR developers and healthcare organizations.

## Introduction

The Safety Assurance Factors for Electronic Health Record (EHR) Resilience (SAFER) Guides provide recommendations to healthcare organizations for conducting proactive self-assessments of the safety and effectiveness of their EHRs as implemented and used across their organizations.^1^ EHR-related safety concerns are often created by continuously changing sociotechnical contextual factors within and external to healthcare organizations. Technology, clinical practice standards, regulations, and policy are constantly evolving. Periodic self-assessments using the SAFER Guides help organizations identify areas they should prioritize to ensure the safety and safe use of EHRs, improve the overall safety of patient care, and improve patient outcomes. In 2025, a revised set of SAFER Guides were released to provide recommendations for addressing the highest risk and most common safety concerns through technology or practice changes. These recommendations can ensure EHR and organizational resilience. This case report describes the process of updating the 2025 SAFER Guides.

## Background

In 2012, the Office of the National Coordinator for Health Information Technology (ONC), now the Assistant Secretary for Technology and Planning (ASTP), commissioned the development of the SAFER Guides.^2^ The first versions of the Guides were released in 2014^3^ and subsequently updated in 2016.^4^ The original SAFER Guides consisted of 8 distinct Guides with 147 recommendations and 540 examples describing how these recommendations could be implemented. In addition, an overarching High Priority Practices Guide that contained the 18 most important recommendations for clinicians was created from the other eight Guides. In 2022, the Centers for Medicare and Medicaid Services (CMS) required that all healthcare organizations participating in the Medicare Promoting Interoperability Program (MPIP) attest annually to having reviewed all nine SAFER Guides.^5^ CMS also required that clinicians participating in the Merit-based Incentive Payment System (MIPS) program attest annually to reviewing the High-Priority Practices SAFER Guide.^6^ Recently, we completed a substantial revision of the original SAFER Guides that were released for use by hospitals, clinicians, and EHR developers in 2025.

## Principles for updating the SAFER Guides

Based on our experience in using the SAFER Guides coupled with feedback received from healthcare organizations around the country, we undertook the SAFER Guide updating process with the following general principles:

1. **No new SAFER Guides would be added:** Even though there have been significant increases in the adoption of several EHR-related health information technologies (eg, personal health records, health information exchanges, and clinical documentation), we concluded that new information could be added to existing SAFER Guides without the need for additional new SAFER Guides.
2. **The sensitivity of the implementation status self-assessment scoring system needs to be increased**: The three-level scoring system used in the 2014 version of the Guides (ie, not implemented, partially implemented, or fully implemented) did not provide organizations with enough information to help them prioritize their work on the partially implemented recommendations in the subsequent year. We needed to explore several alternative ways to increase sensitivity.
3. **An estimate of the level of scientific evidence available for each recommendation is needed**: We increased the clarity of the quality of evidence available and transparency in how it was evaluated.
4. **The literature used to support each recommendation would be updated:** One of the main premises of updating the Guides was to account for the tremendous increase in EHR adoption and accompanying clinical informatics research since the 2016 SAFER update.
5. **Clinicians need a SAFER Guide that can account for their real-world clinical needs:** Because of the organizational focus on the SAFER Guides’ assessment, clinicians often cannot relate as directly to some of the recommendations. We would thus need to highlight the most critical recommendations for clinicians from each of the other SAFER Guides.
6. **SAFER Guide recommendations and descriptions would be simplified and streamlined:** Following the CMS requirements regarding hospitals and clinicians attesting to having reviewed the SAFER Guides, we aimed to consolidate both the number of Guides and the number of recommendations in those Guides to maximize feasibility and utility.
7. **Feedback is needed from a large group of experienced clinicians and informaticians:** Realizing that there continues to be less scientific evidence to support many of the SAFER recommendations than is ideal, we needed to rely on expert opinion and experience in many cases.
8. **Estimates of the overall percentage of SAFER Guide recommendations implemented by an organization are needed:** While not currently required by any EHR safety oversight organizations, we sought to propose a method for estimating the overall percentage of SAFER Guide recommendations implemented across all unique SAFER Guides.

## Methods

We convened a multidisciplinary SAFER Guide core team [D.F.S., T.F., P.S., J.S.A., H.Si.] consisting of experts from informatics, health IT, patient safety, nursing, organizational operations, and clinical medicine. We held several teleconferences with the core team to develop and agree upon the principles for updating the SAFER Guides. We also developed a literature review search strategy and a list of potential peer reviewers who could offer critical feedback on the revised Guides. We reviewed the nine existing SAFER Guides to identify potential recommendations for elimination, combination, or clarification. Next, the nine existing SAFER Guides were allocated to one or more leads from the core team. Each lead was asked to carefully review the existing SAFER recommendations in light of the agreed upon SAFER update principles, findings from the SAFER Guide reviews, feedback from organizations that had completed the SAFER Guides, and recent findings from the scientific literature. Through several online meetings for each SAFER Guide, we discussed each recommendation and decided how it should be addressed in the 2025 revision. Updating the SAFER Guides began in late 2023 and took approximately nine months. After revision, each Guide was reviewed by one or more external expert peer-reviewers who provided feedback for our team’s consideration. The external experts included practicing clinicians, informaticians, and information technology professionals. The final Guides were then delivered to ASTP for formatting and release.

## Results

The 2025 SAFER Guides are based on the best available (2024) evidence from the scientific literature and consensus expert opinion. Several subject matter experts, including those from clinical medicine, patient safety, informatics, quality improvement, risk management, human factors engineering, and usability, helped revise the Guides. There are now 7 unique Guides with 524 examples of implementation guidance, plus the High Priority Practices Guide, which is a compilation of key recommendations from each of the other 7 Guides. An overview of the changes made to the SAFER Guides, organized according to each of the eight updating principles, is listed below.

### 1. Integration of new practices into existing SAFER Guides

Rather than develop additional SAFER Guides, we decided to add several new recommendations to existing Guides to cover important new topics such as patient-clinician communication,^7^ patient access to clinical notes and test results in light of the 21st century cures Act,^8^ use of artificial intelligence for clinical care,^9^ cybersecurity,^10^ integration of FDA-approved medical device data into EHRs,^11^ and more robust software testing procedures.^12^

### 2. Changes to self-assessment scoring

In the original SAFER Guides (2014), the EHR assessment team was asked to “score their implementation status as fully implemented, partially implemented, or not implemented.”^1^ In debriefing with different organizational assessment teams, we learned that organizations often implemented a specific recommendation across most (eg, 95%) of their organization and realized there were often justifiable extenuating circumstances that would prevent them from ever reaching 100% implementation. In these cases, they felt that they had achieved “full implementation” but that the scoring system required them to choose “partially implemented.” The number of “partially implemented” recommendations became problematic when they tried to prioritize work in the coming year.

In an attempt to relieve some of the concerns of these organizations, we developed a 5-point rating scale for the 2025 version of the Guides (see Table 1). This rating scale estimates the percent adherence to the “implementation guidance” suggestions. In addition, assessors were instructed to check the “EHR Limitations” box if there were any limitations with the current version of their EHR that precluded them from fully implementing that recommendation.

**Table 1.** Definitions of the five-point SAFER guide implementation status scale and the EHR limitation indicator.

Implementation status	Definition
Not implemented—(0%)	The organization has not implemented this recommendation.
Making progress (1%-30%)	The organization is in the early or pilot phase of implementing this recommendation as evidenced by following or adopting less than 30% of the implementation guidance.
Halfway there (31%-60%)	The organization is implementing this recommendation and is following or has adopted approximately half of the implementation guidance.
Substantial progress (61%-90%)	The organization has nearly implemented this recommendation and is following or has adopted much of the implementation guidance.
Fully implemented (91%-100%)	The organization follows this recommendation and most implementation guidance is followed consistently and widely adopted.
EHR limitation	The EHR does not offer the features/functionality required to fully implement this recommendation or the implementation guidance.

### 3. Addition of evidence level for each recommendation

Due to the comparatively low level of scientific evidence regarding most EHR configuration and implementation decisions, we created a new three-level evidence hierarchy (Table 2). This hierarchy is based on the concepts that form the Grading of Recommendations Assessment, Development, and Evaluation (GRADE) guidelines used by the Cochrane Collaboration.^13^ It can be used as a first iteration to help evaluate the quality of evidence available for each recommendation.

**Table 2.** Definitions of the evidence levels used to rate each recommendation.

Evidence level	Definition
Required	A rule, regulation, or law requires something identical or similar to this recommendation.
Strong: research-level evidence	Randomized controlled clinical trials, large clinical before/after, or case studies have demonstrated increased patient safety associated with the recommendation.
Medium: practice-level evidence	Reports of adverse patient outcomes have been associated with failure to implement this practice or recommendation or consensus expert opinion supports the recommendation.
Low: context-level evidence	High-performing healthcare organizations routinely follow this best practice or recommendation, but no rigorous research or consensus expert-based opinion exists.

In addition, since many of the SAFER Guide recommendations are required by existing government rules, regulations, and laws, we assigned these as “required,” given that it would be unethical to conduct prospective clinical trials and difficult to find enough instances in which these recommendations have not been implemented to evaluate their effectiveness (eg, requirements for a backup database server or to test the EHR prior to clinical use after major system upgrades). To promote clarity regarding the quality of evidence available and transparency in how it was evaluated, we added an estimate of the level of evidence available to support each recommendation.

### 4. Updating literature to support each recommendation

The 2025 SAFER Guides include citations to 429 resources (eg, peer-reviewed scientific literature, government documents, and websites), which is an absolute increase of 40 citations. Of these, 289 (67%) are resources published since the 2016 SAFER Guide revision.

### 5. A SAFER Guide specifically for clinicians

In 2022, CMS published a rule that required clinicians eligible for their merit-based incentive payment system (MIPS) to attest annually to conducting an annual self-assessment using the High Priority Practices SAFER Guide.^14^ To support these MIPS-eligible clinicians, we reconfigured the High Priority Practices SAFER Guide so it consisted of the most relevant and important recommendations from the other seven Guides for clinicians to understand, support, or implement.

### 6. Simplify and streamline SAFER Guide recommendations and descriptions

Each of the 2014 SAFER Guides was envisioned as a stand-alone Guide that could be used by different groups within a healthcare organization to address known or potential EHR-related safety issues within the particular domain addressed by the Guide. Following CMS’s requirements that all hospitals must review all Guides, we decided to reduce the overlap among several categories of recommendations that were spread across multiple Guides (eg, EHR testing and training, data standardization, technical support staffing, and patient identification). In addition, a recommendation that limited the number of open patient charts on a single workstation to one was removed following the publication of a large, single-site, randomized clinical trial that failed to show that limiting the number of open patient charts reduced wrong-patient errors.^15^ Taken together, these steps allowed us to reduce the total number of recommended practices across all Guides from 147 to 88, a 40% reduction, and the number of implementation guidance examples from 540 to 524, a 3% reduction. In addition, we reduced the number of Guides hospitals must review from 9 to 7. This was made possible because we combined the System Interfaces and System Configuration Guides into a new System Management Guide and because hospitals no longer need to review the High Priority Practices Guide separately given it is now just an aggregation of recommendations from the other Guides.

### 7. Review by a large group of experienced clinicians and informaticians

Drafts of each revised SAFER Guide were iteratively reviewed and revised, often multiple times, by at least five experienced clinicians with extensive informatics training or informaticians with extensive clinical experience. Many reviewers also had experience working for commercial EHR vendors or healthcare organizations that developed their EHRs. In addition, many reviewers were experienced with using the 2014 or 2016 versions of the SAFER Guides to assess EHR safety within their organizations.

### 8. Estimating the overall percentage of SAFER recommendations implemented by an organization

Often, healthcare organizations are interested in summarizing and potentially comparing their overall EHR safety based on the estimated implementation status for all SAFER Guide recommendations. To accomplish this, an organization could take the mean of the midpoints of each SAFER Guide’s recommendation implementation status [eg, Substantial progress midpoint = (61% + 90%)/2 = 75.5%] for each Guide and across all seven unique Guides to generate an estimate of percent adherence to each SAFER Guide or for all SAFER Guides.^16^ We expect that most organizations will be implementing additional best practices for safe and effective EHRs and hence the score should increase year-to-year.

**The 2025 SAFER Guides are organized as follows (see Files S1)**

***Infrastructure Guides—*** A set of Guides that focus on technical recommendations (hardware and software) to run the clinical applications.

- **Contingency Planning:** Includes recommendations associated with mitigating or reducing planned and unplanned EHR unavailability and data corruption or loss during transmission.
- **System Management:** Includes recommendations on the configuration, validation, and maintenance of EHR hardware, software, and application programming interfaces (APIs).

***Clinical Process Guides—*** A set of Guides that focus on several mission-critical clinical processes.

- **Computerized Provider Order Entry (CPOE) with Decision Support:** Includes recommendations on the design, implementation, use, and monitoring of orders and clinical decision support.
- **Test Results Reporting and Follow-Up**: Includes recommendations for optimizing processes and technology for electronic communication and management of test results.
- **Patient Identification**: Includes recommendations to help ensure the reliable identification of patients in the EHR.
- **Clinician Communication**: Includes recommendations to improve communication among clinicians, care teams, and patients.

**Organizational Responsibilities:** An overarching Guide that touches on all the areas covered in the other Guides and focuses on organizational-level recommendations designed to create and maintain a safe, reliable EHR environment by standardizing processes and activities around patient safety.

**High Priority Practices:** Includes 16 recommendations selected from the other seven Guides because of their relevance and importance for practicing clinicians and their organizations.

Each of the eight SAFER Guides begins with a checklist of 6 to 18 recommended practices. Each recommendation is explained in detail on a Practice Worksheet that gives a rationale for the recommendation, provides examples of how to implement each recommended practice, and includes the level of scientific evidence available to support it. The worksheets contain the following fields:

- A list of recommended participants, based on organizational roles, to ensure comprehensive review and accurate estimates of implementation status.
- An area to record:
  - The names and roles of the multidisciplinary team members participating in the assessment.
  - The extent to which each recommended practice has been implemented throughout the organization using a 5-point rating scale.
  - Planned follow-up actions based on the team’s findings and discussions.

## Discussion

The recommended practices in the SAFER Guides are intended to be useful for all EHR users and healthcare organizations. We revised the SAFER Guides to ensure that the 2025 versions represent the best available current evidence for EHR developers and healthcare organizations. In addition to the downloadable version, the content of each SAFER Guide, with interactive references and supporting materials, can also be viewed on ASTP/ONC’s website at: https://www.healthit.gov/topic/safety/safer-guides

Implementation of these recommendations can ensure that the EHR is safe and is used safely. However, to ensure patient safety, healthcare organizations must invest time, resources, and staff to assess, implement, and sustain the best practice recommendations fully. In addition, some of the specific implementation guidance in the SAFER Guides may not apply to an organization due to local sociotechnical factors (eg, patient population, access to in-house technical personnel, or other resource limitations). There is a particularly problematic and well-known humorous dictum in computer science that could be modified to refer to the more general class of implementing any computer-based workflow to read, “The first 90% of the [project] accounts for the first 90% of the [implementation] time. The remaining 10% of the [project] accounts for the other 90%.”^17^ In any time- and financially constrained organization, the leaders often decide to stop work before any project is 100% completed to allow them to shift their resources to other more pressing and less complete projects. Nevertheless, the Guides are a useful resource to support EHR safety and reliability at a healthcare organization. Finally, the SAFER Guides are designed to provide a high-level overview of a set of necessary, but non-exhaustive recommendations that healthcare organizations can use to improve the safety and effectiveness of their EHR implementations. Details related to the process of configuring the EHR software and implementing the clinical and administrative workflows should be carefully explored, studied, and worked through with internal information technology, clinical operations, administrators, legal representatives, and clinicians.^6^

## Conclusion

The recommendations and implementation guidance in the 2025 SAFER Guides represent the best available evidence for EHR developers and healthcare organizations to follow to ensure that their EHRs and subsequent implementations and use are safer and more effective. The Guides were released in early 2025 for use in the 2026 MIPS attestation cycle.

## Supplementary Material

ocaf018_Supplementary_Data

## Data Availability

Revised versions of the SAFER Guides are available at: https://www.healthit.gov/topic/safety/safer-guides.

## References

[1] Sittig DF , Salimi M, Aiyagari R, et al Adherence to recommended electronic health record safety practices across eight health care organizations. J Am Med Inform Assoc. 2018;25:913-918. 10.1093/jamia/ocy033 29701854 PMC7647041

[2] Singh H , Ash JS, Sittig DF. Safety Assurance Factors for Electronic Health Record Resilience (SAFER): study protocol. BMC Med Inform Decis Mak. 2013;13:46. 10.1186/1472-6947-13-46 23587208 PMC3639028

[3] Sittig DF , Ash JS, Singh H. The SAFER guides: empowering organizations to improve the safety and effectiveness of electronic health records. Am J Manag Care. 2014;20:418-423. 25181570

[4] Sittig DF , Singh H. Toward more proactive approaches to safety in the electronic health record era. Jt Comm J Qual Patient Saf. 2017;43:540-547. 10.1016/j.jcjq.2017.06.005 28942779 PMC8136246

[5] Sittig DF , Sengstack P, Singh H. Guidelines for US hospitals and clinicians on assessment of electronic health record safety using SAFER guides. JAMA. 2022;327:719-720. 10.1001/jama.2022.0085 35129591

[6] Office of the National Coordinator for Health Information Technology. High Priority Practices SAFER guide. Accessed October 18, 2024. https://www.healthit.gov/sites/default/files/safer/guides/safer_high_priority_practices.pdf

[7] Rotenstein LS , Landman A, Bates DW. The electronic inbox-benefits, questions, and solutions for the road ahead. JAMA. 2023;330:1735-1736. 10.1001/jama.2023.19195 37812413

[8] Giardina TD , Baldwin J, Nystrom DT, Sittig DF, Singh H. Patient perceptions of receiving test results via online portals: a mixed-methods study. J Am Med Inform Assoc. 2018;25:440-446. 10.1093/jamia/ocx140 29240899 PMC5885801

[9] Sittig DF , Singh H. Recommendations to ensure safety of AI in real-world clinical care. JAMA. 2024. 10.1001/jama.2024.24598 39602298

[10] Sittig DF , Singh H. A socio-technical approach to preventing, mitigating, and recovering from ransomware attacks. Appl Clin Inform. 2016;7:624-632. 10.4338/ACI-2016-04-SOA-0064 27437066 PMC4941865

[11] Sittig DF , Boxwala A, Wright A, et al Patient-centered clinical decision support challenges and opportunities identified from workflow execution models. J Am Med Inform Assoc. 2024;31:1682-1692. 10.1093/jamia/ocae138 38907738 PMC11258405

[12] Darrow JJ , Avorn J, Kesselheim AS. FDA regulation and approval of medical devices: 1976-2020. JAMA. 2021;326:420-432. 10.1001/jama.2021.11171 34342614

[13] Guyatt G , Oxman AD, Akl EA, et al GRADE guidelines: 1. Introduction—GRADE evidence profiles and summary of findings tables. J Clin Epidemiol. 2011;64:383-394. 21195583 10.1016/j.jclinepi.2010.04.026

[14] DEPARTMENT OF HEALTH AND HUMAN SERVICES. Centers for Medicare & Medicaid Services, Medicare program; CY 2022 payment policies under the physician fee schedule and other changes to part B payment policies. Fed Regist. 2021;86:65475-65477. Accessed January 29, 2025. https://www.govinfo.gov/content/pkg/FR-2021-11-19/pdf/2021-23972.pdf

[15] Adelman JS , Applebaum JR, Schechter CB, et al Effect of restriction of the number of concurrently open records in an electronic health record on wrong-patient order errors: a randomized clinical trial. JAMA. 2019 May 14;321:1780-1787. 10.1001/jama.2019.3698 31087021 PMC6518341

[16] Harpe SE. How to analyze Likert and other rating scale data. Curr Pharm Teach Learn. 2015;7:836-850.

[17] Bentley JL. Programming pearls: bumper sticker computer science. Commun ACM. 1985;28:896-901. Accessed 20 December, 2024, https://tildesites.bowdoin.edu/∼ltoma/teaching/cs340/spring05/coursestuff/Bentley_BumperSticker.pdf

