# Supplementary material for: Revisions to the Safety Assurance Factors for Electronic Health Record Resilience (SAFER) Guides to update national recommendations for safe use of electronic health records
Source: J Am Med Inform Assoc. 2025 Apr 12;32(4):755–60. doi: 10.1093/jamia/ocaf018 (PMC12005625; doi:10.1093/jamia/ocaf018)
Supplement: ocaf018_Supplementary_Data [file ocaf018_supplementary_data.zip › ocaf018_Supplementary_Data/Clinical Communication Final.pdf]

Self-Assessment

Clinician Communication

General Instructions for the SAFER Self-Assessment Guides

The Safety Assurance Factors for EHR Resilience (SAFER) guides are designed to help healthcare organizations conduct proactive self-assessments to evaluate the safety and effectiveness of their electronic health record (EHR) implementations. The 2025 SAFER guides have been updated and streamlined to focus on the highest risk, most commonly occurring issues that can be addressed through technology or practice changes to build system resilience in the following areas:

- Organizational Responsibilities
- Patient Identification
- Clinician Communication
- Test Results Reporting and Follow-up
- Computerized Provider Order Entry with Decision Support
- Systems Management
- Contingency Planning
- High Priority Practices - A collection of 16 Recommendation from the other 7 Guides

Each of the eight SAFER Guides begins with a Checklist of recommended practices. The downloadable SAFER Guides provide fillable circles that can be used to indicate the extent to which each recommended practice has been implemented in the organization using a 5-point Likert scale. The Practice Worksheet gives a rationale for the practice and provides examples of how to implement each recommended practice. It contains fields to record team member involvement and follow-up actions based on the assessment. The Worksheet also lists the stakeholders who can provide input to assess each practice (sources of input). In addition to the downloadable version, the content of each SAFER Guide, with interactive references and supporting materials, can also be viewed on ONC's website at: https://www.healthit.gov/topic/safety/safer-guides.

The SAFER guides are based on the best available (2024) evidence from the literature and consensus expert opinion. Subject matter experts in patient safety, informatics, quality improvement, risk management, human factors engineering, and usability developed them. Furthermore, they were reviewed by an external group of practicing clinicians, informaticians, and information technology professionals.

Each guide contains between 6 and 18 recommended practices including its rationale, implementation guidance, and evidence level. The recommended practices in the SAFER Guides are intended to be useful for all EHR users. However, every organization faces unique circumstances and may implement a particular recommended practice differently. As a result, some of the specific implementation guidance in the SAFER Guides for recommended practices may not be applicable to an organization.

The High Priority Practices guide consists of 16 of the most important and relevant recommendations selected from the other 7 guides. It is designed for practicing clinicians to help them understand, implement, and support EHR safety and safe use within their organization. The other seven guides consist of 88 unique recommendations that are relevant for all healthcare providers and organizations.

The SAFER Guides are designed in part to help deal with safety concerns created by the continuously changing sociotechnical landscape that healthcare organizations face. Therefore, changes in technology, clinical practice standards, regulations, and policy should be taken into account when using the SAFER Guides. Periodic self-assessments using the SAFER Guides may also help organizations identify areas where it is particularly important to address the implications of these practice or EHR-based changes for the safety and safe use of EHRs. Ultimately, the goal is to improve the overall safety of our health care system and improve patient outcomes.

The SAFER Guides are not intended to be used for legal compliance purposes, and implementation of a recommended practice does not guarantee compliance with the HIPAA Security or Privacy Rules, Medicare or Medicaid Conditions of Participation, or any other laws or regulations. The SAFER Guides are for informational purposes only and are not intended to be an exhaustive or definitive source. They do not constitute legal advice. Users of the SAFER Guides are encouraged to consult with their own legal counsel regarding compliance with Medicare or Medicaid program requirements, and any other laws.

For additional information on Medicare and Medicaid program requirements, please visit the Centers for Medicare & Medicaid Services website at www.cms.gov. For more information on HIPAA, please visit the HHS Office for Civil Rights website at www.hhs.gov/ocr.

Self-Assessment

Clinician Communication

Introduction

The Clinician Communication SAFER Guide identifies recommended safety practices for communication among clinicians, care teams, and patients. This guide focuses on ensuring reliable electronic communication using EHR-related messaging systems (e.g., SMS text messages, secure text messages, and EHR-based clinician-to-clinician messages) to facilitate care transitions such as discharges and referrals, and patient portal-related communication (such as appointment requests, result reporting to patients, and clinician-to-patient communication). Additionally, this guide covers the implementation of monitoring systems to identify opportunities for communication improvement.

Communication breakdowns are a common contributing factor in preventable adverse events. Optimal design, implementation, and use of EHR communication functionality can help ensure safe, transparent, and efficient information exchange among clinicians, support staff, ancillary services, patients, and their healthcare proxies.

Several components of communication can pose safety risks. For example, full EHR inboxes can be a source of information overload causing critical information to be overlooked. Transitions of care (e.g., hospital discharges or referrals to specialists) are vulnerable to communication gaps related to incomplete care handoffs. Limitations in configuring patient portals may reduce how well patients and their proxies access, navigate, use, and understand the portal and the notes and results it may contain. Many of the concepts involved in clinician-to-clinician communication apply to clinician-patient communication as well.

Completing this self-assessment requires the engagement of people within and outside the organization, including EHR vendors. Because this guide is designed to help organizations prioritize EHR-related safety concerns, clinician leadership should be engaged in collaboration with clinicians and staff members to enable an accurate and thorough assessment. This collaboration should result in consensus about the organization’s future path to optimize EHR-related safety and quality by setting priorities among the recommended practices not yet fully implemented and ensuring a plan is in place to maintain recommended practices already in place. It is vitally important that healthcare organizations and EHR vendors dedicate the required resources and work together to mitigate the highest priority EHR-related communication risks.

Self-Assessment

Clinician Communication

Table of Contents

General Instructions	1
Introduction	2
About the Checklist	5
Checklist	6
Team Worksheet	7
About the Recommended Practice Worksheets	8
Recommended Practice Worksheets	9
References	17

Authors and Peer Reviewers

The SAFER Self-Assessment Guides were developed by health IT safety researchers and informatics experts whose contributions are acknowledged as follows:

Primary authors who contributed to the development of all guides:

Trisha Flanagan, RN, MSN, CPPS, Health Informatics Nurse, Center for Innovations in Quality, Effectiveness and Safety, Michael E. DeBakey Veterans Affairs Medical Center, Houston TX

Hardeep Singh, MD, MPH, Co-Chief, Health Policy, Quality and Informatics Program, Center for Innovations in Quality, Effectiveness and Safety and Professor of Medicine at the Michael E. DeBakey Veterans Affairs Medical Center and Baylor College of Medicine, Houston, TX

Dean F. Sittig MS, PhD, FACMI, FAMIA, FHIMSS, FIAHSI, Professor of Biomedical Informatics, Department of Clinical and Health Sciences, McWilliams School of Biomedical Informatics, University of Texas Health Science Center at Houston, TX and Informatics Review LLC, Lake Oswego, OR

Support staff for the primary authorship team

Rosann Cholankeril, MD, MPH, Center for Innovations in Quality, Effectiveness and Safety, Michael E. DeBakey Veterans Affairs Medical Center and Baylor College of Medicine

Sara Ehsan, MBBS, MPH, Center for Innovations in Quality, Effectiveness and Safety, Michael E. DeBakey Veterans Affairs Medical Center and Baylor College of Medicine

Additional authors who contributed to at least one guide:

Jason S. Adelman, MD, MS, (Patient ID) Chief Patient Safety Officer & Associate Chief Quality Officer, Executive Director, Patient Safety Research, Co-Director, Patient Safety Research Fellowship in Hospital Medicine, New York-Presbyterian Hospital/Columbia University Irving Medical Center, New York, NY

Daniel R. Murphy, MD, MBA, (Clinician Communication, Test Results) Chief Quality Officer, Baylor Medicine, Houston, TX

Patricia Sengstack, DNP, NI-BC, FAAN, FACMI, (Organizational Responsibilities) Senior Associate Dean for Informatics, Director, Nursing Informatics Specialty Program, Vanderbilt University School of Nursing, Vanderbilt University, Nashville, TN

Additional contributors who provided feedback on various guides or parts of guides

Miriam Callahan, MD (Patient ID)
David C. Classen, MD (CPOE, AI recommendation)
Anne Grauer, MD, MS (Patient ID)
Ing Haviland (Patient ID)
Amanda Heidemann, MD (All Guides)
I-Fong Sun Lehman, DrPH, MS (Patient ID)
Christoph U. Lehmann, MD (AI recommendation)
Christopher A. Longhurst, MD, MS (AI recommendation)
Edward R. Melnick, MD (Clinician Communication)
Robert E. Murphy, MD (Organizational Responsibilities)
Ryan P. Radecki, MD, MS (AI recommendation)
Raj Ratwani, PhD (AI recommendation)
Trent Rosenbloom, MD (Clinician Communication)
Lisa Rotenstein, MD (Clinician Communication)
Hojjat Salmasian, MD, PhD (All Guides)
Richard Schreiber, MD (CPOE)
Danny Sands, MD (Clinician Communication)
Debora Simmons, PhD, RN (Organizational Responsibilities)
Carina Sirochinsky (Patient ID)
Neha Thummala, MPH (Patient ID)
Emma Weatherford (Patient ID)
Adam Wright, PhD (CPOE)
Andrew Zimolzak, MD, MMSc (Test Results, Clinician Communication)

This guide was developed under the contract Unintended Consequences of Health IT and Health Information Exchange, Task Order HHSP23337003T/HHSP23320095655WC.

The ATSP/ONC composite mark is a mark of the U.S. Department of Health and Human Services. The contents of the publication or project are solely the responsibility of the authors and do not necessarily represent the official views of the U.S. Department of Health and Human Services, Asssistant Secretary for the Technology Policy/Office of the National Coordinator for Health Information Technology.

The *Checklist* is structured as a quick way to enter and print your self-assessment.

Select the level of implementation achieved by your organization for each Recommended Practice. Your Implementation Status will be reflected on the Recommended Practice Worksheet in this PDF. The implementation status scales are as followed:

**Not Implemented – (0%)** The organization has not implemented this recommendation.	**Making Progress (1 - 30%)** The organization is in the early or pilot phase of implementing this recommendation as evidenced by following or adopting less than 30% of the implementation guidance.	**Halfway there (31 – 60%)** The organization is implementing this recommendation and is following or has adopted approximately half of the implementation guidance.	**Substantial Progress (61-90%)** The organization has nearly implemented this recommendation and is following or has adopted much of the implementation guidance.	**Fully Implemented (91-100%)** The organization follows this recommendation, and most implementation guidance is followed consistently and widely adopted.

The organization should check the following box if there are some limitations with the current version of their EHR that preclude them from fully implementing this recommendation.

EHR Limitation - The EHR does not offer the features/functionality required to fully implement this recommendation or the implementation guidance.

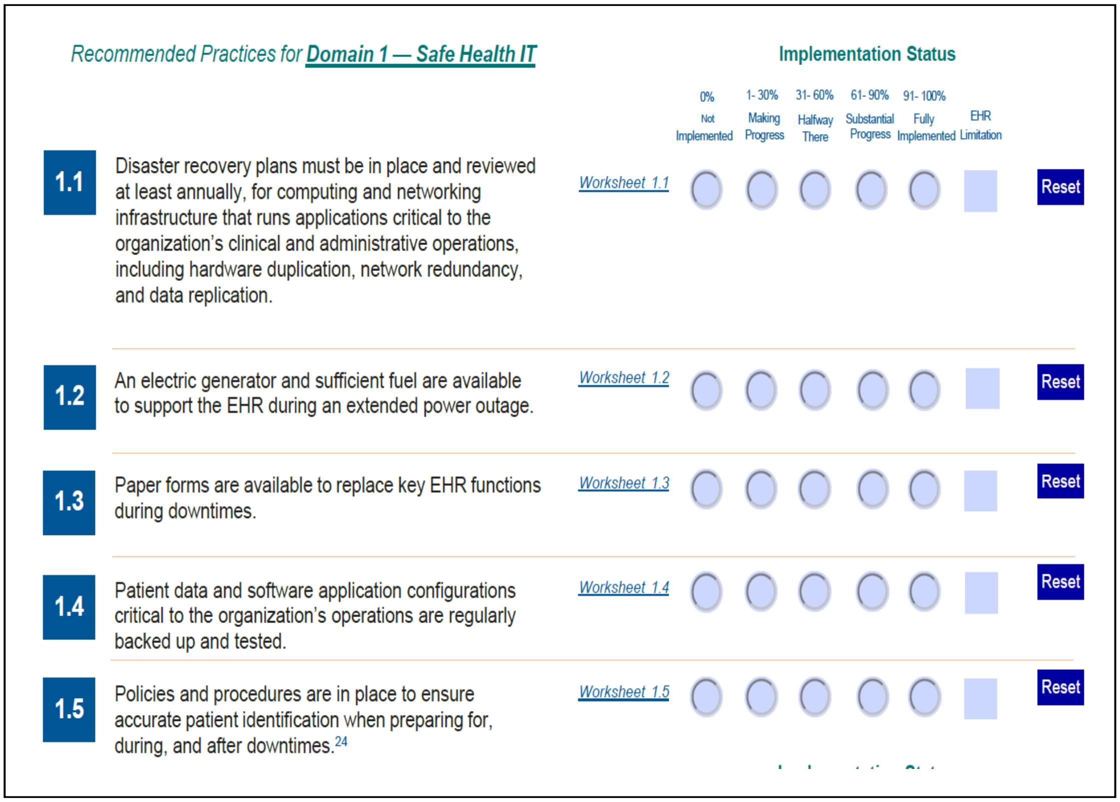

Recommended Practices for Domain 1 — Safe Health IT

Implementation Status

0% Not Implemented | 1-30% Making Progress | 31-60% Halfway There | 61-90% Substantial Progress | 91-100% Fully Implemented | EHR Limitation

1.1 Disaster recovery plans must be in place and reviewed at least annually, for computing and networking infrastructure that runs applications critical to the organization's clinical and administrative operations, including hardware duplication, network redundancy, and data replication. Worksheet 1.1 Reset

1.2 An electric generator and sufficient fuel are available to support the EHR during an extended power outage. Worksheet 1.2 Reset

1.3 Paper forms are available to replace key EHR functions during downtimes. Worksheet 1.3 Reset

1.4 Patient data and software application configurations critical to the organization's operations are regularly backed up and tested. Worksheet 1.4 Reset

1.5 Policies and procedures are in place to ensure accurate patient identification when preparing for, during, and after downtimes.[24] Worksheet 1.5 Reset

The *Domain* associated with the *Recommended Practice(s)* appears at the top of the column

The *Recommended Practice(s)* for the topic appears below the associated *Domain*.

To the right of each *Recommended Practice* is a link to the Recommended Practice Worksheet in this PDF.

The *Worksheet* provides guidance on implementing the practice.

Recommended Practices for Domain 1 — Safe Health IT

	Recommended Practice	Worksheet	0% Not Implemented	1-30% Making Progress	31-60% Halfway There	61-90% Substantial Progress	91-100% Fully Implemented	EHR Limitation	
1.1	EHR-based secure messaging systems ensure accurate, reliable, and efficient transmission of high-risk information.	Worksheet 1.1	○	○	○	○	○		Reset
1.2	Within a single healthcare delivery system, the EHR promotes efficient and timely bidirectional closed-loop communication between referring providers and specialists through the design and implementation of reliable and effective information exchange workflows.	Worksheet 1.2	○	○	○	○	○		Reset
1.3	The EHR facilitates the efficient collection and transmission of relevant clinical information necessary for safe and effective communication during transitions of care.	Worksheet 1.3	○	○	○	○	○		Reset
1.4	The EHR's patient portal is capable of providing patients with access to clinician notes, test results, and secure messages, and is designed and configured to ensure that clinical information displays are easily navigable and understandable for patients.	Worksheet 1.4	○	○	○	○	○		Reset

Recommended Practices for Domain 2 — Using Health IT Safely

	Recommended Practice	Worksheet	0% Not Implemented	1-30% Making Progress	31-60% Halfway There	61-90% Substantial Progress	91-100% Fully Implemented	EHR Limitation	
2.1	The EHR inbox and its use is optimized to reduce clinician burden.	Worksheet 2.1	○	○	○	○	○		Reset

Recommended Practices for Domain 3 - Using Health IT to Monitor Safely

	Recommended Practice	Worksheet	0% Not Implemented	1-30% Making Progress	31-60% Halfway There	61-90% Substantial Progress	91-100% Fully Implemented	EHR Limitation	
3.1	The EHR enables the monitoring of important communication patterns related to clinical messages, referrals, and patient portal notifications.	Worksheet 3.1	○	○	○	○	○		Reset

A Clinician team should complete this self-assessment and evaluate potential health IT-related patient safety risks addressed by this specific SAFER Guide within the context of your particular healthcare organization.

This Team Worksheet is intended to help organizations document the names and roles of the self-assessment team, as well as individual team members' activities. Typically team members will be drawn from a number of different areas within your organization, and in some instances, from external sources. The suggested Sources of Input section in each Recommended Practice Worksheet identifies the types of expertise or services to consider engaging. It may be particularly useful to engage specific clinician and other leaders with accountability for safety practices identified in this guide.

The Worksheet includes fillable boxes that allow you to document relevant information. The Assessment Team Leader box allows documentation of the person or persons responsible for ensuring that the self-assessment is completed. The section labeled Assessment Team Members enables you to record the names of individuals, departments, or other organizations that contributed to the self-assessment. The date that the self-assessment is completed can be recorded in the Assessment Completion Date section and can also serve as a reminder for periodic reassessments. The section labeled Assessment Team Notes is intended to be used, as needed, to record important considerations or conclusions arrived at through the assessment process. This section can also be used to track important factors such as pending software updates, vacant key leadership positions, resource needs, and challenges and barriers to completing the self-assessment or implementing the Recommended Practices in this SAFER Guide.

Assessment Team Leader

Assessment Completion Date

Assessment Team Members

Assessment Team Notes

Reset

Each *Recommended Practice Worksheet* provides guidance on implementing a specific *Recommended Practice*, and allows you to enter and print information about your self-assessment.

The *Suggested Sources of Input* section indicates categories of personnel who can provide information to help evaluate your level of implementation.

The *Rationale* section provides guidance about "why" the safety activities are needed.

Strength of Recommendation section provides an estimate of the strength of evidence available in the scientific literature, or states that it is "required" due to a federal rule, regulation, or conditions of participation, for each recommendation.

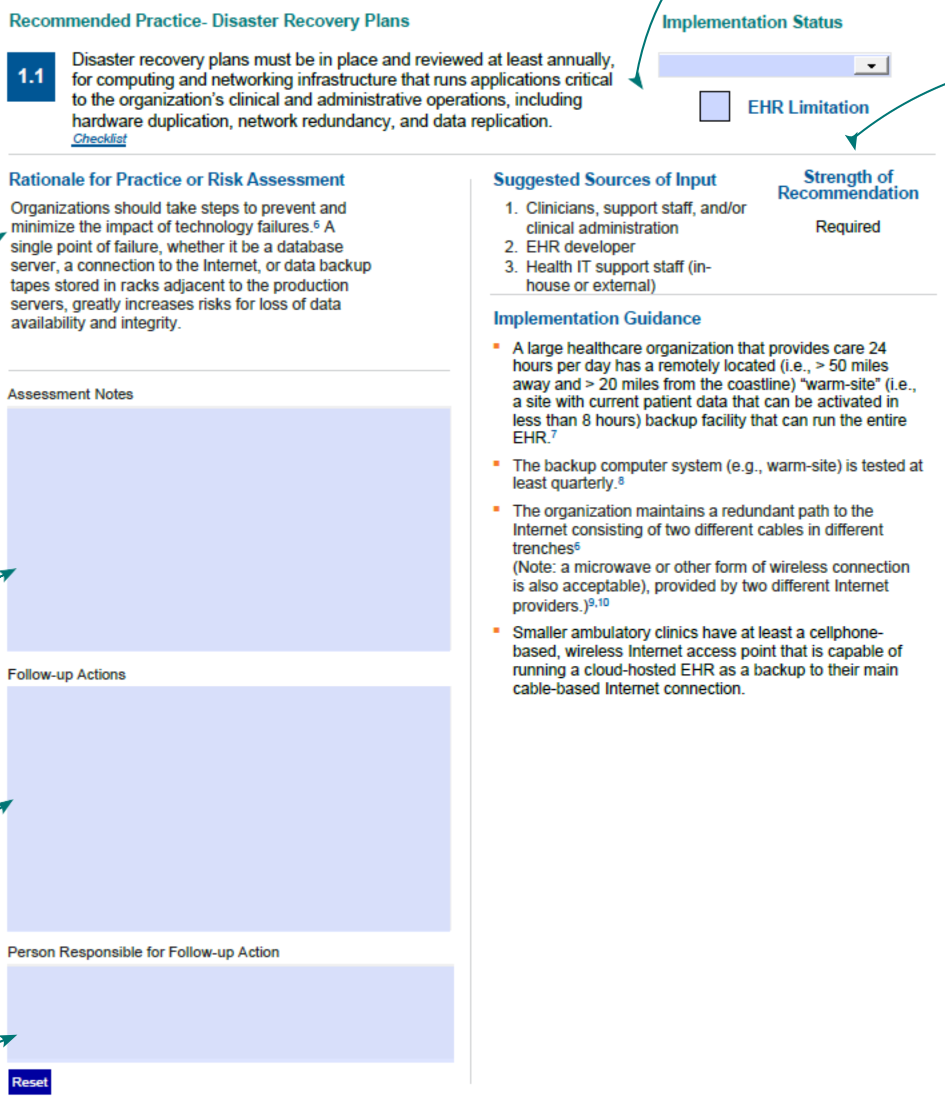
Recommended Practice- Disaster Recovery Plans

Implementation Status

1.1 Disaster recovery plans must be in place and reviewed at least annually, for computing and networking infrastructure that runs applications critical to the organization's clinical and administrative operations, including hardware duplication, network redundancy, and data replication.

Checklist

EHR Limitation

Rationale for Practice or Risk Assessment

Organizations should take steps to prevent and minimize the impact of technology failures.[6] A single point of failure, whether it be a database server, a connection to the Internet, or data backup tapes stored in racks adjacent to the production servers, greatly increases risks for loss of data availability and integrity.

Suggested Sources of Input

1. Clinicians, support staff, and/or clinical administration
2. EHR developer
3. Health IT support staff (in-house or external)

Strength of Recommendation

Required

Implementation Guidance

- A large healthcare organization that provides care 24 hours per day has a remotely located (i.e., > 50 miles away and > 20 miles from the coastline) "warm-site" (i.e., a site with current patient data that can be activated in less than 8 hours) backup facility that can run the entire EHR.[7]
- The backup computer system (e.g., warm-site) is tested at least quarterly.[8]
- The organization maintains a redundant path to the Internet consisting of two different cables in different trenches[6] (Note: a microwave or other form of wireless connection is also acceptable), provided by two different Internet providers.)[9,10]
- Smaller ambulatory clinics have at least a cellphone-based, wireless Internet access point that is capable of running a cloud-hosted EHR as a backup to their main cable-based Internet connection.

Assessment Notes

Follow-up Actions

Person Responsible for Follow-up Action

Reset

Enter any notes about your self-assessment.

Enter any follow-up activities required.

Enter the name of the person responsible for the follow-up activities.

The Implementation Guidance section lists potentially useful practices or scenarios to inform your assessment and implementation of the specific Recommended Practice.

Recommended Practice - Secure Messaging

1.1 EHR-based secure messaging systems ensure accurate, reliable, and efficient transmission of high-risk information.
Checklist

Implementation Status

☐ **EHR Limitation**

Rationale for Practice or Risk Assessment

To avoid unnecessary interruptions and distractions, critical and time-sensitive messages and results to clinicians should be clearly differentiated from routine or information-only communication that does not require immediate attention or action.

Assessment Notes

Follow-up Actions

Person Responsible for Follow-up Action

Reset

Suggested Sources of Input

1. Clinicians
2. Ancillary staff
3. Laboratory and diagnostic imaging staff
4. IT staff
5. Vendors

Strength of Recommendation

Medium

Implementation Guidance

- The EHR ensures closed-loop communication, which implies that "all patient data and information that may require an action are delivered and communicated to the right individuals, at the right time, through the right mode to allow interpretation, critical review, reconciliation, initiation of action, acknowledgment, and appropriate documentation."[1]
- Critical and time-sensitive messages to clinicians are clearly differentiated from routine or information-only communication that does not require immediate attention or action.[2]
- EHR allows urgency levels to be assigned to messages and presents urgent messages in a visually distinct manner. The organization provides guidance to promote succinct and intuitive message content.[3,4]
- Messages can be marked for follow-up on a future date and are automatically re-sent on the specified date and appear as a new message.[5]
- Organization policy for communication requires EHR documentation of patient-specific communication that occurs outside the EHR (e.g., e-mail or text messages sent via computer, smartphone, pager, wireless local area network-based communication devices, or other communication system not integrated with the EHR) within the patient's EHR. Information that should be recorded in the patient's EHR includes sender, recipient, content, time sent, and time acknowledged (if applicable).
- EHR messaging modules automatically capture and store message sender, recipient, content, time, and acknowledgment data.
- The EHR and the organization enable escalation of messages that are unread within a time period (or if no response has been received by the sender depending on urgency). Escalation could involve automatically forwarding the message to an alternate or supervising clinician if the intended recipient is unavailable.[2]

Recommended Practice - Referrals and Consults

1.2 Within a single healthcare delivery system, the EHR promotes efficient and timely bidirectional closed-loop communication between referring providers and specialists through the design and implementation of reliable and effective information exchange workflows.
Checklist

Implementation Status

☐ EHR Limitation

Rationale for Practice or Risk Assessment

Effective and timely electronic communication facilitates information sharing and avoids breakdowns in the referral and consultation process and subsequent care delays. Closing the loop includes mechanisms to ensure that "all patient data and information that may require an action are delivered and communicated to the right individuals, at the right time, through the right mode to allow interpretation, critical review, reconciliation, initiation of action, acknowledgment, and appropriate documentation."[6] This includes acknowledging receipt of the consult or referral with an anticipated turnaround time to completion, and also providing recommendations and findings to the referring provider and the patient.

Assessment Notes

Follow-up Actions

Person Responsible for Follow-up Action

Reset

Suggested Sources of Input

1. Clinicians
2. Clinical leadership
3. Healthcare system IT staff
4. Vendor

Strength of Recommendation

Medium

Implementation Guidance

- The organization has implemented relevant recommendations from "Closing the Loop: A Guide to Safer Ambulatory Referrals in the EHR Era" and considered the following general guidance[6]:
  - Ensure interoperability between EHRs of referring PCPs and specialists
  - Create and use collaborative care agreements to delineate expectations for PCPs and specialists
  - Improve and standardize referral handoff including the ability to indicate the urgency and acuity of the request
  - Create and review process map workflows prior to referral process implementation
  - Ensure clear accountability of patient follow-up
  - Track referral status at the patient level until closed, including anticipated time to scheduling and completion
  - Apply evidence-based patient communication techniques
  - Monitor progress in improving referral communication
- Referral templates are optimized to promote efficiency and reduce data entry burden by auto-populating fields with patient-specific data (e.g., demographics, medications, recent diagnostic test results). Input of specific relevant data elements is required based on organizational or provider preferences (e.g., including the reason for referral).[7]
- There is a mechanism for specialists to electronically provide feedback to referring clinicians, including use of electronic consults that occur only between providers.[7-11]

Recommended Practice - Referrals and Consults (continued)

1.2 Within a single healthcare delivery system, the EHR promotes efficient and timely bidirectional closed-loop communication between referring providers and specialists through the design and implementation of reliable and effective information exchange workflows.

Checklist

Implementation Guidance (continued)

- The organization has established acknowledgment response timeframes for urgent and non-urgent referrals (e.g., within 2 days for urgent referrals, 2 weeks for non-urgent referrals) after which unacknowledged referrals trigger messaging back to the referring provider.[9]
- Referrals and consults that remain unacknowledged after a specified time - which may be variable based on urgency - are automatically identified and escalated for further action.
- The EHR facilitates referral tracking and automatic notification to the referring provider if specialist appointments are not scheduled within a standard timeframe after the referral is accepted or if patients do not show up for the referral visit.[12]
- Referrals that are declined by the specialist trigger a message including rationale to the referring provider.[13]
- Special attention should be paid to referrals and consults involving clinicians in practices using EHR products that are not certified, not able to send or receive computer-interpretable data or documents, or those not even using an EHR. Special care must be taken by the certified EHR-enabled practice to ensure they are recording (e.g., scanning in copies of hand-delivered or faxed copies of records) what is sent and received from these practices along with the time of each interaction and the sender or recipient of each record.

Recommended Practice - Transitions of Care

1.3 The EHR facilitates the efficient collection and transmission of relevant clinical information necessary for safe and effective communication during transitions of care.
Checklist

Implementation Status

☐ **EHR Limitation**

Rationale for Practice or Risk Assessment

Rapid and efficient communication is critical when patients transition from one clinician to another or from one care setting to another, including but not limited to changes in covering service or shift-change, admissions, discharges and/or transfers between different care settings. Clinicians accepting responsibility for the patient should be able to promptly assume care without an in-depth review of the EHR or phone call to the sending clinician.

Assessment Notes

Follow-up Actions

Person Responsible for Follow-up Action

Reset

Suggested Sources of Input

1. Clinicians
2. IT staff
3. Vendors

Strength of Recommendation

Medium

Implementation Guidance

- Essential data elements (e.g., applicable meaningful use common data set items) are automatically populated into transition of care templates (e.g., demographics, problems, discharge medications, time of last medication administration, pending orders and follow-up appointments).[14-17]
- Transition of care documents (i.e., inpatient to outpatient – discharge documents or outpatient to outpatient – referral documents or outpatient to inpatient – admission requests) automatically include a description of the information provided to the patient when appropriate (e.g., on discharge summaries or referrals).[18]
- The EHR enables discharge summaries to be labeled as preliminary or final to allow the addition of pending results or other details.[19]
- The EHR enables the automatic inclusion of inpatient clinician contact information to facilitate future communication.[15]
- The organization has a policy for timely communication of relevant clinical and administrative information during transitions of care, as well as policies regarding who is responsible for follow-up of pending results and completion of specific care plan elements at the time of a transition of care.[20]
- The accuracy of any transition of care documentation that includes large language model (LLM) generated data is validated by the clinician prior to signoff to ensure accuracy, completeness, and absence of hallucination.[21,22]
- The organization has a policy and process for reconciling transition of care documents that are not received electronically (e.g., patient presenting with a paper copy of a discharge summary) by entering key information into the appropriate structured data entry fields, or at least scanning or otherwise incorporating a copy of the document into the patient record.

Recommended Practice - Patient Portals

1.4 The EHR's patient portal is capable of providing patients with access to clinician notes, test results, and secure messages, and is designed and configured to ensure that clinical information displays are easily navigable and understandable for patients.

Checklist

Implementation Status

☐ EHR Limitation

Rationale for Practice or Risk Assessment

Patient portals should promote engagement of patients and their designated proxies in safe and effective care by providing personalized, meaningful, and comprehensible information and facilitating bidirectional communication with the healthcare team and administrative support staff via an easily accessible and usable browser and/or smartphone-based app.

Assessment Notes

Follow-up Actions

Person Responsible for Follow-up Action

Reset

Suggested Sources of Input

1. Clinicians
2. Clinical staff
3. Administrative staff
4. Healthcare system IT staff
5. Vendors
6. Patient representatives, including those knowledgeable of the unique needs of adolescents (if relevant to the practice), and caregivers

Strength of Recommendation

Required

Implementation Guidance

- Portal content, visual display, and navigation are configured to maximize usability and easy learning for patients (e.g., accommodating patient digital and health literacy and numeracy, and preferred language).[23-26]
- The EHR portal is designed to allow access to approved patient-friendly educational content and options for further information either via information buttons adjacent to the clinical concept or via electronic information packets sent to patients by their clinician about results, conditions, wellness strategies, and other health-related information.[23,27]
- The organization has policies and procedures in place to make patient portal use a standard part of how healthcare is delivered. This includes standard procedures to offer training and access to the portal to encourage adoption and continued use by patients.[28,29]
- Patients are informed that their messages may be read and/or answered by someone other than the clinician to whom they were addressed.
- The organization has identified and taken steps to mitigate potential barriers to portal adoption (e.g., using interviews with users or analysis of usage logs to determine usability and uptake).[24]
- Patients regularly receive and have access to educational information about different functions available within the patient portal (e.g., scheduling appointments, downloading information, paying bills, messaging providers) as well as typical turnaround times for bidirectional communication responses.[30]
- The organization has a process, accessible from the patient portal, allowing patient-generated requests for updating information and correcting errors within the EHR (e.g., completed medication courses showing as active or problems that have fully resolved).
- Pending orders, including consult and referral requests are visible within the patient portal.

Recommended Practice - Patient Portals (continued)

1.4 The EHR's patient portal is capable of providing patients with access to clinician notes, test results, and secure messages, and is designed and configured to ensure that clinical information displays are easily navigable and understandable for patients.

Checklist

Implementation Guidance (continued)

- The patient portal provides patients and internal staff with access to a transaction log showing when messages were sent, who sent them, whether/when they were read, and by whom.
- The patient portal provides patients and internal staff with free text search capability of message content to facilitate finding relevant messages.
- The EHR enables the implementation of specific privacy and security features to support caregivers with proxy logins and adolescents and their parents and/or guardians.[31]
- The organization has policies and procedures describing mitigation approaches for patients who receive potentially distressing diagnostic results via the portal before a clinician has had an opportunity to discuss the findings with them.[32]
- The organization provides guidance and sets expectations with patients around the timeliness and content of communication via the patient portal.[33-35]

Recommended Practice - Inbox Design, Configuration, and Management

2.1 The EHR inbox and its use is optimized to reduce clinician burden.
Checklist

Implementation Status

EHR Limitation

Rationale for Practice or Risk Assessment

Inbox configuration aligned with its effective and efficient management can help clinicians focus on important and high-priority information.

Assessment Notes

Follow-up Actions

Person Responsible for Follow-up Action

Reset

Suggested Sources of Input

1. Clinicians
2. IT staff
3. Vendors

Strength of Recommendation

Medium

Implementation Guidance

- High-priority messages, abnormal test results, or otherwise time-sensitive inbox messages and tasks are visually distinct from routine inbox communication.[2]
- The EHR allows users to organize and prioritize inbox content, including allowing sorting, filtering, and flagging features preferred by individual clinicians (e.g., based on date, source, patient, urgency, message type).[2,36]
- Inbox configuration and management allows support staff to triage and act on messages within their scope of practice (e.g., processing refill requests, communicating normal test results, scheduling visits) without requiring the clinician to read or sort through administrative and non-medical queries.[37,38]
- Inbox functionality includes the ability to flag, forward, and add comments to messages and tasks.[2,37]
- Out-of-office messaging functionality is enabled to make it clear to the sender that an inbox is not being monitored.[2]
- The EHR allows automatic message forwarding to a surrogate clinician during a specific time period or circumstance, such as when the clinician is absent from work.
- The organization's clinical leadership actively works to identify and mitigate inbox-related burdens by implementing processes designed to facilitate team communication and streamline inbox content.[39]
- Appropriately tested and effective artificial intelligence solutions are integrated to help categorize messages and draft suggested responses to patients.[38]

Recommended Practice - Monitor Communication Patterns

3.1 The EHR enables the monitoring of important communication patterns related to clinical messages, referrals, and patient portal notifications.
Checklist

Implementation Status

☐ EHR Limitation

Rationale for Practice or Risk Assessment

Monitoring time-sensitive and important clinical communications can identify opportunities to improve safety by finding and addressing potential problems related to informing and responding to messages between clinicians and the care team and ancillary staff, as well as to and from patients.

Suggested Sources of Input

1. Clinicians
2. Clinical staff
3. Quality Improvement staff
4. Health IT Support Staff
5. Vendors

Strength of Recommendation

Medium

Assessment Notes

Follow-up Actions

Person Responsible for Follow-up Action

Reset

Implementation Guidance

- The organization monitors rates of unacknowledged clinician inbox messages, messages sent to patients in their portal's inbox, and tasks.[40]
- The organization defines and tracks expected response timeframes for specific types of messages (e.g., urgent referrals responded to within two days, hospital discharge summaries sent to primary care provider within three days of discharge, inpatient order to admit is signed off at or before the time of admission). Findings are used to identify and resolve any deficiencies.
- Inbox message monitoring identifies quality improvement projects and targets interventions for clinicians with higher rates of unacknowledged inbox messages and tasks.[41]
- Physician burnout, turnover, productivity, and EHR use metrics are analyzed to identify opportunities to identify physicians at high risk of departure who may benefit from targeted inbox management interventions.[42,43]
- Patient portal adoption and utilization rates are monitored and analyzed, including how these differ by patient language, race/ethnicity, and other demographics.[44]
- Messaging content and response patterns are periodically reviewed to identify opportunities for improving communication quality.[45]
- The organization provides sufficient administrative time for clinicians to appropriately manage inbox messages and the clinical work associated with them.[36,37,46]

References

1. Partnership for Health IT Patient Safety, ECRI Institute. Health IT safe practices for closing the loop: Mitigating delayed, missed, and incorrect diagnoses related to diagnostic testing and medication changes using health IT. www.ecri.org Web site. https://www.ecri.org/ Resources/HIT/Closing_Loop/Closing_the_Loop_Toolkit.pdf. Updated 2018. Accessed Jul 22, 2024.

2. Murphy DR, Giardina TD, Satterly T, Sittig DF, Singh H. An exploration of barriers, facilitators, and suggestions for improving electronic health record inbox-related usability: A qualitative analysis. JAMA Netw Open. 2019;2(10):e1912638. https:// pubmed.ncbi.nlm.nih.gov/31584683/. doi: 10.1001/jamanetworkopen.2019.12638; PMID: 31584683; PMC6784746.

3. Murphy DR, Reis B, Sittig DF, Singh H. Notifications received by primary care practitioners in electronic health records: A taxonomy and time analysis. Am J Med. 2012;125(2):209.e1-7. https://pubmed.ncbi.nlm.nih.gov/22269625/. doi: 10.1016/j.amjmed.2011.07.029; PMID: 22269625.

4. Murphy DR, Reis B, Kadiyala H, et al. Electronic health record-based messages to primary care providers: Valuable information or just noise? Arch Intern Med. 2012;172(3):283-5. https://pubmed.ncbi.nlm.nih.gov/22332167/. doi: 10.1001/archinternmed.2011.740; PMID: 22332167.

5. Jin J, Jerzak J, Sinsky CA. Taming the EHR playbook: implement effective system-level policies to reduce the burden of EHR work. www.edhub.ama-assn.org Web site. https://edhub.ama-assn.org/steps-forward/module/2798925. Updated 2022. Accessed Aug 6, 2024.

6. Institute for Healthcare Improvement. Closing the loop: A guide to safer ambulatory referrals in the EHR era. www.ihi.org Web site. https://www.ihi.org/resources/publications/closing-loop-guide-safer-ambulatory-referrals-ehr-era. Updated 2017. Accessed Jul 22, 2024.

7. Esquivel A, Sittig DF, Murphy DR, Singh H. Improving the effectiveness of electronic health record-based referral processes. BMC Med Inform Decis Mak. 2012;12:107. https://pubmed.ncbi.nlm.nih.gov/22973874/. doi: 10.1186/1472-6947-12-107; PMID: 22973874; PMC3492108.

8. Cifra CL, Sittig DF, Singh H. Bridging the feedback gap: A sociotechnical approach to informing clinicians of patients' subsequent clinical course and outcomes. BMJ Qual Saf. 2021;30(7):591-597. https://pubmed.ncbi.nlm.nih.gov/33958442/. doi: 10.1136/
bmjqs-2020-012464; PMID: 33958442; PMC8237185.

9. Barnett ML, Mehrotra A, Frolkis JP, et al. Implementation science workshop: Implementation of an electronic referral system in a large academic medical center. J Gen Intern Med. 2016;31(3):343-352. https://pubmed.ncbi.nlm.nih.gov/26556594/. doi: 10.1007/ s11606-015-3516-y; PMID: 26556594; PMC4762816.

10. Thompson MA, Fuhlbrigge AL, Pearson DW, Saxon DR, Oberst-Walsh LA, Thomas JF. Building eConsult (electronic consults) capability at an academic medical center to improve efficiencies in delivering specialty care. J Prim Care Community Health. 2021;12:21501327211005303. https://pubmed.ncbi.nlm.nih.gov/33759622/. doi: 10.1177/21501327211005303; PMID: 33759622; PMC8010812.

11. Meeker D, Friedberg MW, Knight TK, et al. Effect of peer benchmarking on specialist electronic consult performance in a los angeles safety-net: A cluster randomized trial. J Gen Intern Med. 2022;37(6):1400-1407. https://pubmed.ncbi.nlm.nih.gov/34505234/. doi: 10.1007/s11606-021-07002-1; PMID: 34505234; PMC8428492.

12. Singh H, Naik AD, Rao R, Petersen LA. Reducing diagnostic errors through effective communication: Harnessing the power of information technology. J Gen Intern Med. 2008;23(4):489-494. https://pubmed.ncbi.nlm.nih.gov/18373151/. doi: 10.1007/ s11606-007-0393-z; PMID: 18373151; PMC2359508.

13. Singh H, Esquivel A, Sittig DF, et al. Follow-up actions on electronic referral communication in a multispecialty outpatient setting. J Gen Intern Med. 2011;26(1):64-69. https://pubmed.ncbi.nlm.nih.gov/20848235/. doi: 10.1007/s11606-010-1501-z; PMID: 20848235; PMC3024094.

14. Lakhaney D, Banker SL. An evaluation of the content of pediatric discharge summaries. Hospital Pediatrics. 2020;10(11):949-954. https://pubmed.ncbi.nlm.nih.gov/33097565/. doi: 10.1542/hpeds.2020-0148; PMID: 33097565.

References

15. Victor Lane Rose MBA. Recommendations to improve EHR-generated discharge summaries for better care transitions to aging services. Annals of Long-Term Care. 2020;28(1). https://www.hmpgloballearningnetwork.com/site/altc/articles/recommendations-improve-ehr-generated-discharge-summaries-better-care-transitions-aging.

16. Horwitz LI, Jenq GY, Brewster UC, et al. Comprehensive quality of discharge summaries at an academic medical center. J Hosp Med. 2013;8(8):436-443. https://pubmed.ncbi.nlm.nih.gov/23526813/. doi: 10.1002/jhm.2021; PMID: 23526813; PMC3695055.

17. Office of the National Coordinator for Health Information Technology. 2015 edition common clinical data set reference document. https://www.healthit.gov/sites/default/files/ccds_reference_document_v1_1.pdf. Updated 2015. Accessed Aug 6, 2024.

18. Patel S, Utting I, Ang WW, et al. Improving the quality of inpatient discharge summaries. Int J Risk Saf Med. 2022;33(S1):S63-S67. https://pubmed.ncbi.nlm.nih.gov/35871370/. doi: 10.3233/JRS-227026; PMID: 35871370; PMC9844071.

19. Unnewehr M, Schaaf B, Marev R, Fitch J, Friederichs H. Optimizing the quality of hospital discharge summaries--a systematic review and practical tools. Postgrad Med. 2015;127(6):630-639. https://pubmed.ncbi.nlm.nih.gov/26074128/. doi: 10.1080/00325481.2015.1054256; PMID: 26074128.

20. Singh H, Zwaan L. Web exclusives. annals for hospitalists inpatient notes - reducing diagnostic error-A new horizon of opportunities for hospital medicine. Ann Intern Med. 2016;165(8):HO2-HO4. https://pubmed.ncbi.nlm.nih.gov/27750328/. doi: 10.7326/M16-2042; PMID: 27750328.

21. Tung JYM, Gill SR, Sng GGR, et al. Comparison of the quality of discharge letters written by large language models and junior clinicians: Single-blinded study. J Med Internet Res. 2024;26:e57721. https://pubmed.ncbi.nlm.nih.gov/39047282/. doi: 10.2196/57721; PMID: 39047282.

22. Williams CYK, Bains J, Tang T, et al. Evaluating large language models for drafting emergency department discharge summaries. medRxiv. 2024:2024.04.03.24305088. https://pubmed.ncbi.nlm.nih.gov/38633805/. doi: 10.1101/2024.04.03.24305088; PMID: 38633805; PMC11023681.

23. Giardina TD, Baldwin J, Nystrom DT, Sittig DF, Singh H. Patient perceptions of receiving test results via online portals: A mixed-methods study. J Am Med Inform Assoc. 2017;25(4):440-446. https://www.ncbi.nlm.nih.gov/pmc/articles/PMC5885801/. doi: 10.1093/jamia/ocx140; PMID: 29240899; PMC5885801.

24. Lyles CR, Nelson EC, Frampton S, Dykes PC, Cemballi AG, Sarkar U. Using electronic health record portals to improve patient engagement: Research priorities and best practices. Ann Intern Med. 2020;172(11 Suppl):S123-S129. https://www.ncbi.nlm.nih.gov/pmc/articles/PMC7800164/. doi: 10.7326/M19-0876; PMID: 32479176; PMC7800164.

25. Turchioe MR, Myers A, Isaac S, et al. A systematic review of patient-facing visualizations of personal health data. Appl Clin Inform. 2019;10(4):751-770. https://pubmed.ncbi.nlm.nih.gov/31597182/. doi: 10.1055/s-0039-1697592; PMID: 31597182; PMC6785326.

26. Yin R, Law K, Neyens D. Examining how internet users trust and access electronic health record patient portals: Survey study. JMIR Hum Factors. 2021;8(3):e28501. https://pubmed.ncbi.nlm.nih.gov/34546182/. doi: 10.2196/28501; PMID: 34546182; PMC8493465.

27. Sittig DF, Ash JS, Wright A, et al. How can we partner with electronic health record vendors on the complex journey to safer health care? J Healthc Risk Manag. 2020;40(2):34-43. https://pubmed.ncbi.nlm.nih.gov/32648286/. doi: 10.1002/jhrm.21434; PMID: 32648286.

28. Richwine C. Progress and persistent disparities in patient access to electronic health information. JAMA Health Forum. 2023;4(11):e233883. https://pubmed.ncbi.nlm.nih.gov/37948063/. doi: 10.1001/jamahealthforum.2023.3883; PMID: 37948063; PMC10638642.

References

29. Richwine C, Johnson C, Patel V. Disparities in patient portal access and the role of providers in encouraging access and use. J Am Med Inform Assoc. 2023;30(2):308-317. https://pubmed.ncbi.nlm.nih.gov/36451262/. doi: 10.1093/jamia/ocac227; PMID: 36451262; PMC9846679.

30. Swoboda CM, DePuccio MJ, Fareed N, McAlearney AS, Walker DM. Patient portals: Useful for whom and for what? A cross-sectional analysis of national survey data. Appl Clin Inform. 2021;12(3):573-581. https://pubmed.ncbi.nlm.nih.gov/34233367/. doi: 10.1055/s-0041-1731339; PMID: 34233367; PMC8263128.

31. Steitz BD, Wong JIS, Cobb JG, Carlson B, Smith G, Rosenbloom ST. Policies and procedures governing patient portal use at an academic medical center. JAMIA Open. 2019;2(4):479-488. https://pubmed.ncbi.nlm.nih.gov/32025645/. doi: 10.1093/jamiaopen/ooz039; PMID: 32025645; PMC6994003.

32. van Kuppenveld SI, van Os-Medendorp H, Tiemessen NA, van Delden JJ. Real-time access to electronic health record via a patient portal: Is it harmful? A retrospective observational study. J Med Internet Res. 2020;22(2):e13622. https://pubmed.ncbi.nlm.nih.gov/32044753/. doi: 10.2196/13622; PMID: 32044753; PMC7055752.

33. Rotenstein LS, Holmgren AJ, Downing NL, Bates DW. Differences in total and after-hours electronic health record time across ambulatory specialties. JAMA Intern Med. 2021;181(6):863-865. https://pubmed.ncbi.nlm.nih.gov/33749732/. doi: 10.1001/jamainternmed.2021.0256; PMID: 33749732; PMC7985815.

34. Rotenstein LS, Fong AS, Jeffery MM, et al. Gender differences in time spent on documentation and the electronic health record in a large ambulatory network. JAMA Netw Open. 2022;5(3):e223935. https://pubmed.ncbi.nlm.nih.gov/35323954/. doi: 10.1001/jamanetworkopen.2022.3935; PMID: 35323954; PMC8948526.

35. Rotenstein LS, Melnick ER, Jeffery M, et al. Association of primary care physicians' electronic inbox activity patterns with patients' likelihood to recommend the physician. J Gen Intern Med. 2024;39(1):150-152. https://pubmed.ncbi.nlm.nih.gov/37731135/. doi: 10.1007/s11606-023-08417-8; PMID: 37731135; PMC10817856.

36. Murphy DR, Meyer AND, Russo E, Sittig DF, Wei L, Singh H. The burden of inbox notifications in commercial electronic health records. JAMA Intern Med. 2016;176(4):559-560. https://www.ncbi.nlm.nih.gov/pmc/articles/PMC4860883/. doi: 10.1001/jamainternmed.2016.0209; PMID: 26974737; PMC4860883.

37. Murphy DR, Satterly T, Giardina TD, Sittig DF, Singh H. Practicing clinicians' recommendations to reduce burden from the electronic health record inbox: A mixed-methods study. J Gen Intern Med. 2019;34(9):1825-1832. https://pubmed.ncbi.nlm.nih.gov/31292905/. doi: 10.1007/s11606-019-05112-5; PMID: 31292905; PMC6712240.

38. Rotenstein LS, Landman A, Bates DW. The electronic inbox-benefits, questions, and solutions for the road ahead. JAMA. 2023;330(18):1735-1736. https://pubmed.ncbi.nlm.nih.gov/37812413/. doi: 10.1001/jama.2023.19195; PMID: 37812413.

39. Dymek C, Kim B, Melton GB, Payne TH, Singh H, Hsiao C. Building the evidence-base to reduce electronic health record-related clinician burden. J Am Med Inform Assoc. 2021;28(5):1057-1061. https://pubmed.ncbi.nlm.nih.gov/33340326/. doi: 10.1093/jamia/ocaa238; PMID: 33340326; PMC8068419.

40. Barbieri AL, Fadare O, Fan L, Singh H, Parkash V. Challenges in communication from referring clinicians to pathologists in the electronic health record era. J Pathol Inform. 2018;9:8. https://pubmed.ncbi.nlm.nih.gov/29692945/. doi: 10.4103/jpi.jpi_70_17; PMID: 29692945; PMC5896165.

41. Richmond RT, McFadzean IJ, Vallabhaneni P. Reaching the summit of discharge summaries: A quality improvement project. BMJ Open Quality. 2021;10(1):e001142; PMID: 33589507.

42. Melnick ER, Fong A, Nath B, et al. Analysis of electronic health record use and clinical productivity and their association with physician turnover. JAMA Netw Open. 2021;4(10):e2128790. https://pubmed.ncbi.nlm.nih.gov/34636911/. doi: 10.1001/jamanetworkopen.2021.28790; PMID: 34636911; PMC8511970.

43. Lopez K, Li H, Paek H, et al. Predicting physician departure with machine learning on EHR use patterns: A longitudinal cohort from a large multi-specialty ambulatory practice - PubMed. PLoS One . 2023;18(2):e0280251. https://pubmed.ncbi.nlm.nih.gov/36724149/. doi: 10.1371/journal.pone.0280251; PMID: 36724149; PMC9891518.

References

44. Hansen MA, Hirth J, Zoorob R, Langabeer J. Demographics and clinical features associated with rates of electronic message utilization in the primary care setting. Int J Med Inform. 2024;183(105339). https://pubmed.ncbi.nlm.nih.gov/38219417/. doi: 10.1016/j.ijmedinf.2024.105339; PMID: 38219417.

45. Heisey-Grove D, Rathert C, McClelland LE, Jackson K, DeShazo J. Classification of patient- and clinician-generated secure messages using a theory-based taxonomy. Health Sci Rep. 2021;4(2):e295. https://pubmed.ncbi.nlm.nih.gov/34084944/. doi: 10.1002/hsr2.295; PMID: 34084944; PMC8142627.

46. Martinez KA, Schulte R, Rothberg MB, Tang MC, Pfoh ER. Patient portal message volume and time spent on the EHR: An observational study of primary care clinicians. J Gen Intern Med. 2024;39(4):566-572. https://pubmed.ncbi.nlm.nih.gov/38129617/. doi: 10.1007/s11606-023-08577-7; PMID: 38129617; PMC10973312.
