# Supplementary material for: Revisions to the Safety Assurance Factors for Electronic Health Record Resilience (SAFER) Guides to update national recommendations for safe use of electronic health records
Source: J Am Med Inform Assoc. 2025 Apr 12;32(4):755–60. doi: 10.1093/jamia/ocaf018 (PMC12005625; doi:10.1093/jamia/ocaf018)
Supplement: ocaf018_Supplementary_Data [file ocaf018_supplementary_data.zip › ocaf018_Supplementary_Data/Contingency Planning Final.pdf]

Self-Assessment

Contingency Planning

General Instructions for the SAFER Self-Assessment Guides

The Safety Assurance Factors for EHR Resilience (SAFER) guides are designed to help healthcare organizations conduct proactive self-assessments to evaluate the safety and effectiveness of their electronic health record (EHR) implementations. The 2025 SAFER guides have been updated and streamlined to focus on the highest risk, most commonly occurring issues that can be addressed through technology or practice changes to build system resilience in the following areas:

- Organizational Responsibilities
- Patient Identification
- Clinician Communication
- Test Results Reporting and Follow-up
- Computerized Provider Order Entry with Decision Support
- Systems Management
- Contingency Planning
- High Priority Practices - A collection of 16 Recommendations from the other 7 Guides

Each of the eight SAFER Guides begins with a Checklist of recommended practices. The downloadable SAFER Guides provide fillable circles that can be used to indicate the extent to which each recommended practice has been implemented in the organization using a 5-point Likert scale. The Practice Worksheet gives a rationale for the practice and provides examples of how to implement each recommended practice. It contains fields to record team member involvement and follow-up actions based on the assessment. The Worksheet also lists the stakeholders who can provide input to assess each practice (sources of input). In addition to the downloadable version, the content of each SAFER Guide, with interactive references and supporting materials, can also be viewed on ONC's website at: https://www.healthit.gov/topic/safety/safer-guides.

The SAFER guides are based on the best available (2024) evidence from the literature and consensus expert opinion. Subject matter experts in patient safety, informatics, quality improvement, risk management, human factors engineering, and usability developed them. Furthermore, they were reviewed by an external group of practicing clinicians, informaticians, and information technology professionals.

Each guide contains between 6 and 18 recommended practices including its rationale, implementation guidance, and evidence level. The recommended practices in the SAFER Guides are intended to be useful for all EHR users. However, every organization faces unique circumstances and may implement a particular recommended practice differently. As a result, some of the specific implementation guidance in the SAFER Guides for recommended practices may not be applicable to an organization.

The High Priority Practices guide consists of 16 of the most important and relevant recommendations selected from the other 7 guides. It is designed for practicing clinicians to help them understand, implement, and support EHR safety and safe use within their organization. The other seven guides consist of 88 unique recommendations that are relevant for all healthcare providers and organizations.

The SAFER Guides are designed in part to help deal with safety concerns created by the continuously changing sociotechnical landscape that healthcare organizations face. Therefore, changes in technology, clinical practice standards, regulations, and policy should be taken into account when using the SAFER Guides. Periodic self-assessments using the SAFER Guides may also help organizations identify areas where it is particularly important to address the implications of these practice or EHR-based changes for the safety and safe use of EHRs. Ultimately, the goal is to improve the overall safety of our health care system and improve patient outcomes.

The SAFER Guides are not intended to be used for legal compliance purposes, and implementation of a recommended practice does not guarantee compliance with the HIPAA Security or Privacy Rules, Medicare or Medicaid Conditions of Participation, or any other laws or regulations. The SAFER Guides are for informational purposes only and are not intended to be an exhaustive or definitive source. They do not constitute legal advice. Users of the SAFER Guides are encouraged to consult with their own legal counsel regarding compliance with Medicare or Medicaid program requirements, and any other laws.

For additional information on Medicare and Medicaid program requirements, please visit the Centers for Medicare & Medicaid Services website at www.cms.gov. For more information on HIPAA, please visit the HHS Office for Civil Rights website at www.hhs.gov/ocr.

Self-Assessment

Contingency Planning

Introduction

The *Contingency Planning SAFER Guide* identifies recommended safety practices associated with planned or unplanned EHR unavailability — instances in which clinicians or other end users cannot access all or part of the EHR. Occasional temporary unavailability of EHRs is inevitable, due to failures of software and hardware infrastructure, as well as power outages and natural and man-made disasters. Such unavailability can introduce substantial safety risks to organizations that have not adequately prepared. Effective contingency planning addresses the causes and consequences of EHR unavailability, and involves processes and preparations that can minimize the frequency and impact of such events, ensuring continuity of care.

EHR unavailability, which will occur in every EHR-enabled healthcare environment,[1] represents a significant potential patient safety hazard that directly affects patient care. Documented potential hazards include an increased risk of medication errors,[2] unavailability of images,[3] delayed follow-up of tests[4] and canceled procedures. The potential impact of EHR unavailability increases as such systems are deployed across multiple, geographically dispersed facilities within a healthcare system.[5] The contingency planning team should include practicing clinicians to ensure that the technical components align with and support the clinical processes and workflows impacted by their decisions. The substitute workflows that must be designed and then employed during downtimes are particularly sensitive to clinician input and cooperation. In addition to the substantial initial contingency planning effort, a continuous, reliable review and maintenance process must be developed and followed. EHR safety and effectiveness can be improved by establishing proper downtime procedures, policies, and practices. The collaboration between clinicians and staff members in completing the self- assessment in this guide will enable an accurate snapshot of the organization’s EHR contingency planning status (in terms of safety) and, even more importantly, should lead to a consensus about the organization’s future path to optimize EHR-related safety and quality.

Interaction with HIPAA

Many recommendations herein overlap with standards and implementation specifications of the HIPAA Security Rule, which focuses on ensuring the confidentiality, integrity, and availability of electronically protected health information. Because the focus of the guide differs from that of the Security Rule, completing the checklist here will not equate with compliance with HIPAA. However, creating a contingency plan as required by the HIPAA Security Rule will address many, but not all, of the recommended safety-oriented practices in this guide. We encourage coordination of completion of the self-assessment in this SAFER Guide with contingency planning for purposes of HIPAA compliance to provide a uniform approach to patient safety and data protection.

Self-Assessment

Contingency Planning

Table of Contents

General Instructions	1
Introduction	2
About the Checklist	5
Checklist	6
Team Worksheet	8
About the Recommended Practice Worksheets	9
Practice Worksheets	10
References	23

Authors and Peer Reviewers

The SAFER Self-Assessment Guides were developed by health IT safety researchers and informatics experts whose contributions are acknowledged as follows:

Primary authors who contributed to the development of all guides:

Trisha Flanagan, RN, MSN, CPPS, Health Informatics Nurse, Center for Innovations in Quality, Effectiveness and Safety, Michael E. DeBakey Veterans Affairs Medical Center, Houston TX

Hardeep Singh, MD, MPH, Co-Chief, Health Policy, Quality and Informatics Program, Center for Innovations in Quality, Effectiveness and Safety and Professor of Medicine at the Michael E. DeBakey Veterans Affairs Medical Center and Baylor College of Medicine, Houston, TX

Dean F. Sittig MS, PhD, FACMI, FAMIA, FHIMSS, FIAHSI, Professor of Biomedical Informatics, Department of Clinical and Health Sciences, McWilliams School of Biomedical Informatics, University of Texas Health Science Center at Houston, TX and Informatics Review LLC, Lake Oswego, OR

Support staff for the primary authorship team

Rosann Cholankeril, MD, MPH, Center for Innovations in Quality, Effectiveness and Safety, Michael E. DeBakey Veterans Affairs Medical Center and Baylor College of Medicine

Sara Ehsan, MBBS, MPH, Center for Innovations in Quality, Effectiveness and Safety, Michael E. DeBakey Veterans Affairs Medical Center and Baylor College of Medicine

Additional authors who contributed to at least one guide:

Jason S. Adelman, MD, MS, (Patient ID) Chief Patient Safety Officer & Associate Chief Quality Officer, Executive Director, Patient Safety Research, Co-Director, Patient Safety Research Fellowship in Hospital Medicine, New York-Presbyterian Hospital/Columbia University Irving Medical Center, New York, NY

Daniel R. Murphy, MD, MBA, (Clinician Communication, Test Results) Chief Quality Officer, Baylor Medicine, Houston, TX

Patricia Sengstack, DNP, NI-BC, FAAN, FACMI, (Organizational Responsibilities) Senior Associate Dean for Informatics, Director, Nursing Informatics Specialty Program, Vanderbilt University School of Nursing, Vanderbilt University, Nashville, TN

Additional contributors who provided feedback on various guides or parts of guides

Miriam Callahan, MD (Patient ID)
David C. Classen, MD (CPOE, AI recommendation)
Anne Grauer, MD, MS (Patient ID)
Ing Haviland (Patient ID)
Amanda Heidemann, MD (All Guides)
I-Fong Sun Lehman, DrPH, MS (Patient ID)
Christoph U. Lehmann, MD (AI recommendation)
Christopher A. Longhurst, MD, MS (AI recommendation)
Edward R. Melnick, MD (Clinician Communication)
Robert E. Murphy, MD (Organizational Responsibilities)
Ryan P. Radecki, MD, MS (AI recommendation)
Raj Ratwani, PhD (AI recommendation)
Trent Rosenbloom, MD (Clinician Communication)
Lisa Rotenstein, MD (Clinician Communication)
Hojjat Salmasian, MD, PhD (All Guides)
Richard Schreiber, MD (CPOE)
Danny Sands, MD (Clinician Communication)
Debora Simmons, PhD, RN (Organizational Responsibilities)
Carina Sirochinsky (Patient ID)
Neha Thummala, MPH (Patient ID)
Emma Weatherford (Patient ID)
Adam Wright, PhD (CPOE)
Andrew Zimolzak, MD, MMSc (Test Results, Clinician Communication)

This guide was developed under the contract Unintended Consequences of Health IT and Health Information Exchange, Task Order HHSP23337003T/HHSP23320095655WC.

The ATSP/ONC composite mark is a mark of the U.S. Department of Health and Human Services. The contents of the publication or project are solely the responsibility of the authors and do not necessarily represent the official views of the U.S. Department of Health and Human Services, Asssistant Secretary for the Technology Policy/Office of the National Coordinator for Health Information Technology.

>Table of Contents | >About the Checklist | >Team Worksheet | >About the Practice Worksheets | >Practice Worksheets

The *Checklist* is structured as a quick way to enter and print your self-assessment.

Select the level of implementation achieved by your organization for each Recommended Practice. Your Implementation Status will be reflected on the Recommended Practice Worksheet in this PDF. The implementation status scales are as followed:

Not Implemented – (0%)
The organization has not implemented this recommendation.

Making Progress (1 - 30%)
The organization is in the early or pilot phase of implementing this recommendation as evidenced by following or adopting less than 30% of the implementation guidance.

Halfway there (31 – 60%)
The organization is implementing this recommendation and is following or has adopted approximately half of the 6 of 26 implementation guidance.

Substantial Progress (61-90%)
The organization has nearly implemented this recommendation and is following or has adopted much of the implementation guidance.

Fully Implemented (91-100%)
The organization follows this recommendation, and most implementation guidance is followed consistently and widely adopted.

The organization should check the following box if there are some limitations with the current version of their EHR that preclude them from fully implementing this recommendation.

EHR Limitation - The EHR does not offer the features/functionality required to fully implement this recommendation or the implementation guidance.

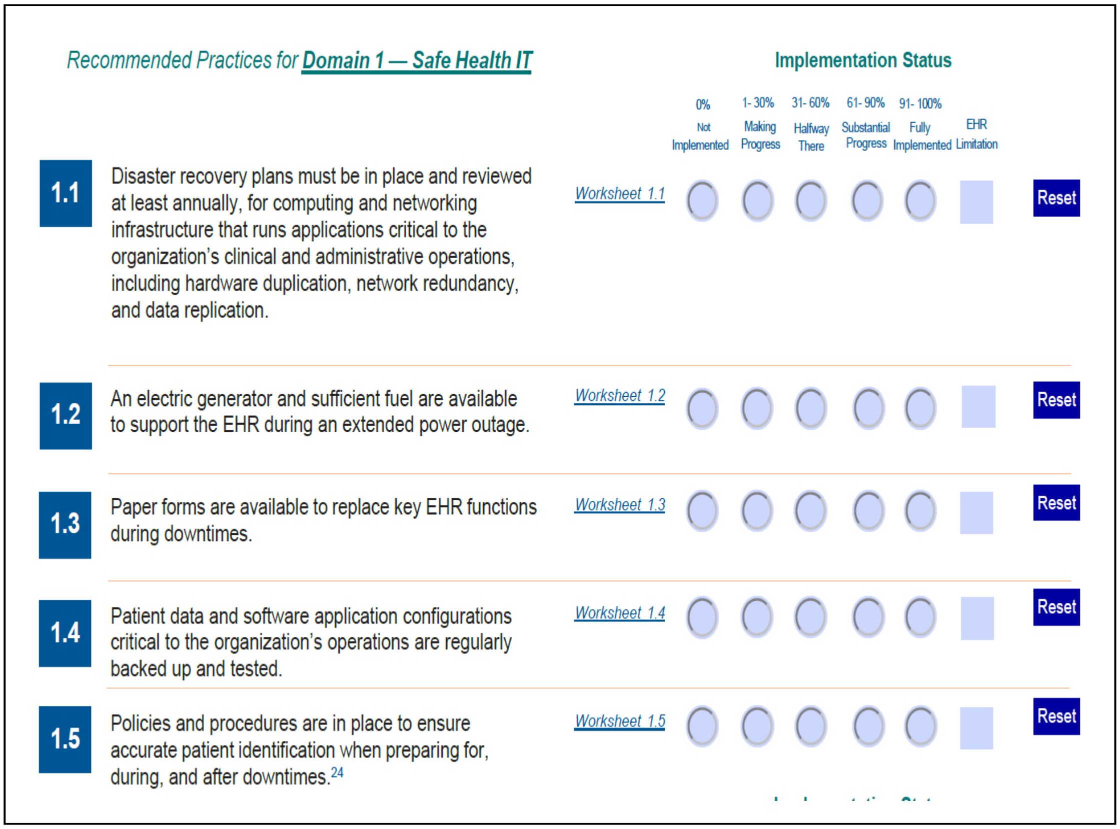

Recommended Practices for ***Domain 1 — Safe Health IT***

Implementation Status

	Practice		0% Not Implemented	1-30% Making Progress	31-60% Halfway There	61-90% Substantial Progress	91-100% Fully Implemented	EHR Limitation	
1.1	Disaster recovery plans must be in place and reviewed at least annually, for computing and networking infrastructure that runs applications critical to the organization's clinical and administrative operations, including hardware duplication, network redundancy, and data replication.	Worksheet 1.1							Reset
1.2	An electric generator and sufficient fuel are available to support the EHR during an extended power outage.	Worksheet 1.2							Reset
1.3	Paper forms are available to replace key EHR functions during downtimes.	Worksheet 1.3							Reset
1.4	Patient data and software application configurations critical to the organization's operations are regularly backed up and tested.	Worksheet 1.4							Reset
1.5	Policies and procedures are in place to ensure accurate patient identification when preparing for, during, and after downtimes.[24]	Worksheet 1.5							Reset

The *Domain* associated with the *Recommended Practice(s)* appears at the top of the column

The *Recommended Practice(s)* for the topic appears below the associated *Domain*.

To the right of each *Recommended Practice* is a link to the Recommended Practice Worksheet in this PDF.

The *Worksheet* provides guidance on implementing the practice.

Recommended Practices for Domain 1 — Safe Health IT

	Practice	Worksheet	Implementation Status: 0% Not Implemented	1-30% Making Progress	31-60% Halfway There	61-90% Substantial Progress	91-100% Fully Implemented	EHR Limitation	
1.1	Disaster recovery plans must be in place and reviewed at least annually, for computing and networking infrastructure that runs applications critical to the organization's clinical and administrative operations, including hardware duplication, network redundancy, and data replication.	Worksheet 1.1							Reset
1.2	An electric generator and sufficient fuel are available to support the EHR during an extended power outage.[11,12]	Worksheet 1.2							Reset
1.3	Paper forms are available to replace key EHR functions during downtimes.[16]	Worksheet 1.3							Reset
1.4	Patient data and software application configuration settings critical to the organization's operations are regularly backed up and tested.[19]	Worksheet 1.4							Reset
1.5	Policies and procedures are in place to ensure accurate patient identification when preparing for, during, and after downtimes.[24]	Worksheet 1.5							Reset

Recommended Practices for Domain 2 — Using Health IT Safely

	Practice	Worksheet	Implementation Status: 0% Not Implemented	1-30% Making Progress	31-60% Halfway There	61-90% Substantial Progress	91-100% Fully Implemented	EHR Limitation	
2.1	Staff are trained and tested on downtime and recovery procedures.[6]	Worksheet 2.1							Reset
2.2	The communication strategy for downtime and recovery periods is independent of the computing infrastructure that supports the EHR.[24]	Worksheet 2.2							Reset
2.3	Written policies and procedures on EHR downtimes and recovery processes ensure continuity of operations with regard to safe patient care and critical business operations.[32-34]	Worksheet 2.3							Reset
2.4	Users are trained on ransomware prevention strategies, including how to identify malicious emails and fraudulent telephone callers asking for login access or other privileged information.[38,39]	Worksheet 2.4							Reset
2.5	Policies and procedures describe how to stop and restart the exchange of data across system interfaces in an orderly manner following a downtime event.	Worksheet 2.5							Reset

Recommended Practices for ***Domain 3 — Monitoring Safety***

Implementation Status

		Worksheet	0% Not Implemented	1- 30% Making Progress	31- 60% Halfway There	61- 90% Substantial Progress	91- 100% Fully Implemented	EHR Limitation	
**3.1**	A comprehensive testing, monitoring, and auditing strategy is in place to prevent, detect, and manage EHR downtime events.	*Worksheet 3.1*	○	○	○	○	○		Reset
**3.2**	Functional system downtimes (i.e., unacceptably slow response time) are identified and addressed proactively.	*Worksheet 3.2*	○	○	○	○	○		Reset
**3.3**	Conduct an in-depth review of unexpected system downtimes lasting over 24 hours using root-cause or failure modes and effects analysis or similar approaches.[49]	*Worksheet 3.3*	○	○	○	○	○		Reset

Clinicians should complete this self-assessment and evaluate potential health IT-related patient safety risks addressed by this specific SAFER Guide within the context of your particular healthcare organization.

This Team Worksheet is intended to help organizations document the names and roles of the self-assessment team, as well as individual team members' activities. Typically team members will be drawn from a number of different areas within your organization, and in some instances, from external sources. The suggested Sources of Input section in each Recommended Practice Worksheet identifies the types of expertise or services to consider engaging. It may be particularly useful to engage specific clinician and other leaders with accountability for safety practices identified in this guide.

The Worksheet includes fillable boxes that allow you to document relevant information. The Assessment Team Leader box allows documentation of the person or persons responsible for ensuring that the self-assessment is completed.

The section labeled Assessment Team Members enables you to record the names of individuals, departments, or other organizations that contributed to the self-assessment. The date that the self-assessment is completed can be recorded in the Assessment Completion Date section and can also serve as a reminder for periodic reassessments. The section labeled Assessment Team Notes is intended to be used, as needed, to record important considerations or conclusions arrived at through the assessment process. This section can also be used to track important factors such as pending software updates, vacant key leadership positions, resource needs, and challenges and barriers to completing the self-assessment or implementing the Recommended Practices in this SAFER Guide.

Assessment Team Leader

Assessment Completion Date

Assessment Team Members

Assessment Team Notes

Reset

Each *Recommended Practice Worksheet* provides guidance on implementing a specific *Recommended Practice*, and allows you to enter and print information about your self-assessment.

The *Suggested Sources of Input* section indicates categories of personnel who can provide information to help evaluate your level of implementation.

Strength of Recommendation section provides an estimate of the strength of evidence available in the scientific literature, or states that it is "required" due to a federal rule, regulation, or conditions of participation, for each recommendation.

The *Rationale* section provides guidance about "why" the safety activities are needed.

Recommended Practice- Disaster Recovery Plans

Implementation Status

1.1 Disaster recovery plans must be in place and reviewed at least annually, for computing and networking infrastructure that runs applications critical to the organization's clinical and administrative operations, including hardware duplication, network redundancy, and data replication.
Checklist

EHR Limitation

Rationale for Practice or Risk Assessment

Organizations should take steps to prevent and minimize the impact of technology failures.[6] A single point of failure, whether it be a database server, a connection to the Internet, or data backup tapes stored in racks adjacent to the production servers, greatly increases risks for loss of data availability and integrity.

Suggested Sources of Input

1. Clinicians, support staff, and/or clinical administration
2. EHR developer
3. Health IT support staff (in-house or external)

Strength of Recommendation

Required

Implementation Guidance

- A large healthcare organization that provides care 24 hours per day has a remotely located (i.e., > 50 miles away and > 20 miles from the coastline) "warm-site" (i.e., a site with current patient data that can be activated in less than 8 hours) backup facility that can run the entire EHR.[7]
- The backup computer system (e.g., warm-site) is tested at least quarterly.[8]
- The organization maintains a redundant path to the Internet consisting of two different cables in different trenches[6]
(Note: a microwave or other form of wireless connection is also acceptable), provided by two different Internet providers.)[9,10]
- Smaller ambulatory clinics have at least a cellphone-based, wireless Internet access point that is capable of running a cloud-hosted EHR as a backup to their main cable-based Internet connection.

Assessment Notes

Enter any notes about your self-assessment.

Follow-up Actions

Enter any follow-up activities required.

Person Responsible for Follow-up Action

Enter the name of the person responsible for the follow-up activities.

Reset

The Implementation Guidance section lists potentially useful practices or scenarios to inform your assessment and implementation of the specific Recommended Practice.

Recommended Practice- Disaster Recovery Plans

1.1 Disaster recovery plans must be in place and reviewed at least annually, for computing and networking infrastructure that runs applications critical to the organization's clinical and administrative operations, including hardware duplication, network redundancy, and data replication.
Checklist

Implementation Status

☐ EHR Limitation

Rationale for Practice or Risk Assessment

Organizations should take steps to prevent and minimize the impact of technology failures.[6] A single point of failure, whether it be a database server, a connection to the Internet, or data backup tapes stored in racks adjacent to the production servers, greatly increases risks for loss of data availability and integrity.

Suggested Sources of Input

1. Clinicians, support staff, and/or clinical administration
2. EHR developer
3. Health IT support staff (in-house or external)

Strength of Recommendation

Required

Implementation Guidance

- A large healthcare organization that provides care 24 hours per day has a remotely located (i.e., > 50 miles away and > 20 miles from the coastline) "warm-site" (i.e., a site with current patient data that can be activated in less than 8 hours) backup facility that can run the entire EHR.[7]
- The backup computer system (e.g., warm-site) is tested at least quarterly.[8]
- The organization maintains a redundant path to the Internet consisting of two different cables in different trenches.[6] (Note: a microwave or other form of wireless connection is also acceptable, provided by two different Internet providers.)[9,10]
- Smaller ambulatory clinics have at least a cellphone-based, wireless Internet access point that is capable of running a cloud-hosted EHR as a backup to their main cable-based Internet connection.

Assessment Notes

Follow-up Actions

Person Responsible for Follow-up Action

Reset

Recommended Practice- Electric Generator

1.2 An electric generator and sufficient fuel are available to support the EHR during an extended power outage.[11,12]

Checklist

Implementation Status

☐ EHR Limitation

Rationale for Practice or Risk Assessment

Most healthcare organizations must be able to continue running their health IT infrastructure and preserve data and communication capabilities in cases of sustained power outages.

Suggested Sources of Input

1. Clinicians, support staff, and/or clinical administration
2. Health IT support staff

Strength of Recommendation

Required

Implementation Guidance

- Organizations evaluate the consequences to patient safety and business operations due to loss of power that shuts down the EHR, and implement concrete plans to keep the EHR running to the extent needed to avoid unacceptable consequences.
- Recently, CMS provided a waiver for some healthcare organizations to replace their existing, gas or diesel generator-supplied backup power systems with electrical microgrid systems, small-scale electrical grids where the sources of electricity can be provided by clean energy technologies (e.g., fuel cells, solar, wind, energy storage, etc.).[13]
- In the event of a power failure, there is an uninterruptible power supply (UPS), either batteries or a "flywheel," capable of providing instantaneous power to maintain the EHR for at least 10 minutes.
- The UPS is tested regularly (optimally on at least a monthly basis).
- The on-site, backup electrical generator can maintain EHR functions critical to the organization's operation (e.g., results review, order entry, clinical documentation).[14]
- The organization maintains 2 days of fuel for the generator on-site. For a larger supply (e.g., 96 hours), organizations must consider the risks of storing a large amount of highly flammable fuel close to a healthcare facility or the surrounding community.[12]
- The generator is tested regularly (optimally, at least monthly).
- The UPS and the generator are kept in secure locations that are not likely to flood.[15]

Assessment Notes

Follow-up Actions

Person Responsible for Follow-up Action

Reset

Recommended Practice- Paper Forms Available

1.3 Paper forms are available to replace key EHR functions during downtimes.[16]

Checklist

Implementation Status

☐ **EHR Limitation**

Rationale for Practice or Risk Assessment

Clinical and administrative operations need to continue in the event of a downtime.

Assessment Notes

Follow-up Actions

Person Responsible for Follow-up Action

Reset

Suggested Sources of Input

1. Clinicians, support staff, and/or clinical administration

Strength of Recommendation

Required

Implementation Guidance

- The organization maintains enough paper forms within each patient care area to care for their patients for at least 8 hours. Paper forms should include those required to enter orders and document the administration of medications, laboratory, and radiology tests on each unit.[17,18]
- There is a process in place to ensure that the information recorded on paper during the downtime gets entered and reconciled into the EHR following its reactivation (e.g., entering information, such as orders, as coded data along with scanning of paper documents whose contents are not otherwise entered into the EHR).[12]

Recommended Practice- Data Back Up

1.4 Patient data and software application configuration settings critical to the organization's operations are regularly backed up and tested.[19]

Checklist

Implementation Status

☐ EHR Limitation

Rationale for Practice or Risk Assessment

Failure of electro-mechanical devices is inevitable. Backup of mission-critical patient data and EHR system configuration allows system restoration to a "pre-failure" state with minimal data and time loss.

Suggested Sources of Input

1. Clinicians, support staff, and/or clinical administration
2. EHR developer
3. Health IT support staff

Strength of Recommendation

Required

Implementation Guidance

- The organization has a daily, off-site, complete, encrypted backup of patient data.[20]
- Critically important patient data should be backed up as close as possible to real-time.
- If using a remotely hosted EHR (e.g., cloud-based solution), the EHR provider backs up data with tape, Internet, redundant drives, or any means necessary to allow full recovery from incidents.[21]
- The off-site backup is tested regularly (i.e., complete system and patient data restore) (optimally on at least a monthly basis).[22]
- The content required to configure the system is backed up regularly (optimally every month and always before every EHR or supporting computer system upgrade).
- The organization maintains multiple backups, which are created at different times.
- Backup media are physically secured in a location separate from the operational data stores.
- The backup storage media should be separate and distinct (e.g., Air gap) from normal file storage to facilitate recovery from ransomware attacks. [23]
- Backup media are rendered unreadable (i.e., use software to scramble media contents or physically destroy/shred media) before disposal.
- The organization has a "read-only" backup EHR system that is updated frequently (optimally at least hourly).
- The read-only EHR system is tested regularly (optimally at least weekly).
- Users can print from the read-only EHR system.
- If there is a "unit-level" read-only backup EHR system, it is connected to a local UPS or "red plug" (i.e., an outlet connected to the organization's backup electrical generator).

Assessment Notes

Follow-up Actions

Person Responsible for Follow-up Action

Reset

Recommended Practice- Patient Identification

1.5 Policies and procedures are in place to ensure accurate patient identification when preparing for, during, and after downtimes.[24]

Checklist

Implementation Status

☐ EHR Limitation

Rationale for Practice or Risk Assessment

Without policies, procedures, and processes in place to manage patient identification during downtimes, mismatches and lost records could compromise patient confidentiality, data integrity, and patient safety.

Assessment Notes

Follow-up Actions

Person Responsible for Follow-up Action

Reset

Suggested Sources of Input

1. Clinicians, support staff, and/or clinical administration
2. EHR developer

Strength of Recommendation

Required

Implementation Guidance

- There is a mechanism in place to register new patients during downtime, including the assignment of unique temporary patient record numbers along with a process for reconciling these new patient IDs once the EHR comes back online.
- There are standardized processes for patient identification during laboratory specimen collection, medication administration, imaging procedures, and delivery of results.[18]

Recommended Practice- Training for Downtime

2.1 Staff are trained and tested on downtime and recovery procedures.[6]
Checklist

Implementation Status

EHR Limitation

Rationale for Practice or Risk Assessment

At any given time, many organizations are likely to have employees who do not know how to function in a paper record-based clinical or administrative environment.[25]

Suggested Sources of Input

1. Clinicians, support staff, and/or clinical administration

Strength of Recommendation

Required

Implementation Guidance

- Organizations establish and follow training requirements so that each employee knows what to do to keep the organization operating safely during EHR downtimes.[26]
- Clinicians are trained in the use of paper-based ordering and charting tools.
- The organization offers a job aid, such as a small, self-contained reference card or checklist, to help clinical staff find available resources and actions during EHR downtimes.[27]
- The organization conducts unannounced EHR "downtime drills" at least once a year.[28]
- Clinicians have been trained on how and when to activate and use the "read-only" backup EHR system.[29]
- Clinicians and other staff members have reliable access to the login information for the emergency, downtime, read-only backup EHR system, which may be different than user-specific credentials used for the live or production EHR.
- The organization maintains a comprehensive list of system-to-system interfaces or computer connections that is reviewed on a regular basis (e.g., every six months or annually) as a part of on-going contingency planning. The list should have a specific indication of whether there are legal/regulatory issues that may require special notification to the other party if there is a downtime such as a state-based immunization registry or prescription drug monitoring program. [30]

Assessment Notes

Follow-up Actions

Person Responsible for Follow-up Action

Reset

Recommended Practice- Communication During Downtime

2.2 The communication strategy for downtime and recovery periods is independent of the computing infrastructure that supports the EHR.[24]

Checklist

Implementation Status

☐ EHR Limitation

Rationale for Practice or Risk Assessment

The organization needs to be prepared to communicate with key personnel without the use of the computer or computer network used by the EHR.

Suggested Sources of Input

1. Clinicians, support staff, and/or clinical administration
2. Health IT support staff

Strength of Recommendation

Required

Implementation Guidance

- The organization has methods other than those that rely on the same computing infrastructure as the EHR (i.e., not email, a website, X (formerly Twitter), or voice-over-IP) to notify key organizational administrators and clinicians when the EHR is down (either planned or unplanned), for example, a mobile phone-based call tree.[28, 31]
- The organization has a mechanism in place to activate the read-only backup EHR system and notify clinicians how to access it.
- The organization has a mechanism to notify clinicians when the EHR is back online (planned or unplanned) and ready for use.[25]

Assessment Notes

Follow-up Actions

Person Responsible for Follow-up Action

Reset

Recommended Practice- Policies Regarding Downtime

2.3 Written policies and procedures on EHR downtimes and recovery processes ensure continuity of operations with regard to safe patient care and critical business operations.[32-34]

Checklist

Implementation Status

☐ EHR Limitation

Rationale for Practice or Risk Assessment

Written policies and procedures on EHR downtime and recovery ensure that everyone has the same understanding on how to care for patients and maintain critical business operations during inevitable downtimes, whether planned or unplanned.

Assessment Notes

Follow-up Actions

Person Responsible for Follow-up Action

Reset

Suggested Sources of Input

1. Clinicians, support staff, and/or clinical administration
2. Health IT support staff

Strength of Recommendation

Required

Implementation Guidance

- The organization has a written EHR downtime and recovery policy that describes key elements such as when a downtime should be called; how often further communication will be delivered; who will be in charge during the downtime (both on the clinical and technical side); how everyone will be notified; and how information collected during the downtime is entered into the EHR.[34-36]
- The EHR downtime policy is reviewed at least every 2 years.[37]
- The EHR downtime policy describes when the warm-site backup process should be activated (ideally before the system has been unavailable due to unplanned activities for 2 hours).
- A paper copy of the current EHR downtime and recovery policy is available in clinical units.
- A paper copy of the current EHR downtime and recovery policy is also stored in a safe, off-site location.

Recommended Practice- Ransomware Prevention

2.4 Users are trained on ransomware prevention strategies, including how to identify malicious emails and fraudulent telephone callers asking for login access or other privileged information. [38,39]

Checklist

Implementation Status

☐ EHR Limitation

Rationale for Practice or Risk Assessment

Malicious email attachments or callers asking for personal login information are often the first point of entry for ransomware attacks.

Suggested Sources of Input

1. Clinicians, support staff, and/or clinical administration
2. EHR developer

Strength of Recommendation

Required

Implementation Guidance

- Users are trained to first, hover over links to see the URL (Uniform Resource Locator) destination before clicking, and second, think about the attachment or link - do you know the sender, does the email have a sense of urgency or deadline to take action, are their spelling or grammatical errors in the message? Do not click on the link or attachment if not sure. When in doubt call or email (in a separate email) the sender or the organization requesting information to confirm it is legitimate.[40]
- The organization trains users to identify spam, phishing, and spear-phishing messages, and users avoid clicking on potentially weaponized attachments (such as *.exe,*.zip, *.rar, *.7z, *.js, *.wsf, *.docm, *.xlsm, *.pptm, *.rtf, *.msi, *.bat, *.com, *.cmd, *.hta, *.scr, *.pif, *.reg, *.vbs, *.cpl, *.jar files). Safer file attachment formats include (*.jpg, *.png, *.pdf, *.docx, *.xlsx, and *.pptx).[41,42]
- Training should reinforce that legitimate organizational mail messages (e.g., your employer's IT department, your bank, your credit card company, companies you work with) should always meet the following requirements: 1) never ask you to download and open file attachments; 2) never ask for you to enter account or password information; 3) always have a telephone number you can call (i.e., out-of-band check); 4) always be associated with an email address and name that people can check in their local directory; and 5) contain website links that display the complete internet address (URL) to build trust.
- The organization restricts users' ability to install and run software applications using the principle of "Least Privilege", or minimizes users' access to only those systems, services, and data required by their job.
- The organization considers disabling the USB ports on the organization's computers.[43]
- The organization conducts simulated phishing attacks (i.e., sends fraudulent but safe email messages or websites that appear to be from legitimate sources) to raise user's awareness of the problem.[44]
- The organization conducts simulated ransomware attack detection and recovery drills from both the clinical[45] and technical[46] perspectives.

Assessment Notes

Follow-up Actions

Person Responsible for Follow-up Action

Reset

Recommended Practice- Restarting System Interfaces

2.5 Policies and procedures describe how to stop and restart the exchange of data across the system interfaces in an orderly manner following a downtime event.

Checklist

Implementation Status

☐ EHR Limitation

Rationale for Practice or Risk Assessment

Failure to stop and restart an internal or external computer system interface properly can result in "in transit" data being lost or corrupted without any warning to users.

Suggested Sources of Input

1. Diagnostic services
2. EHR developer
3. Health IT support staff
4. Pharmacy

Strength of Recommendation

Medium

Implementation Guidance

- Ensure that all system interface buffers are empty prior to stopping or restarting the system.
- If the interface must be disconnected while the sending system continues to produce data for transmission (e.g., laboratory tests ordered through CPOE), the buffers are of adequate size and behavior to prevent data loss.
- The organization has a method of communicating to users when a clinical interface is not functioning properly (e.g., an alert on the login page, or a user-appropriate alert in the EHR whenever data retrieval or transmission is attempted but not completed).
- The organization or IT department has a policy and procedure that describes how to start, stop, re-start, test, and monitor, both internal (e.g., EHR to locally-maintained nutrition management system) and external (e.g., EHR to Surescripts) system-to-system computer interfaces. The procedures are available and consulted during hardware/ software upgrades.

Assessment Notes

Follow-up Actions

Person Responsible for Follow-up Action

Reset

Recommended Practice- Strategy for Downtime

3.1 A comprehensive testing, monitoring, and auditing strategy is in place to prevent, detect, and manage EHR downtime events.
Checklist

Implementation Status

☐ **EHR Limitation**

Rationale for Practice or Risk Assessment

Comprehensive testing and monitoring strategies can prevent and minimize the impact of natural disasters, technology failures, or cybersecurity attacks.

Suggested Sources of Input

1. Clinicians, support staff, and/or clinical administration
2. EHR developer
3. Health support IT staff

Strength of Recommendation

Required

Implementation Guidance

- The organization regularly monitors and reports on system downtime events.[47]
- The organization regularly monitors, tracks, and audits access to patient information on EHR systems.[38]
- The organization regularly monitors and reports on system response time (optimally under 2 seconds) for important clinical tasks (e.g., results review, order entry, patient look-up).[48]
- The organization has a written policy describing the different hardware, software, process, and people-related downtime testing procedures.
- The organization maintains a log of all downtime-related testing activities.
- Unplanned downtimes and the effectiveness of follow-up to prevent them from recurring are monitored by the top leadership.

Assessment Notes

Follow-up Actions

Person Responsible for Follow-up Action

Reset

Recommended Practice- Functional Downtimes

3.2 Functional system downtimes (i.e., unacceptably slow response time) are identified and addressed proactively.
Checklist

Implementation Status

☐ EHR Limitation

Rationale for Practice or Risk Assessment

Slow computer response times significantly impede user efficiency and can result in "type ahead" errors in which the computer saves commands (e.g., repeated enter key presses) and enters them (unbeknownst to the user) in the default data entry field once the form loads, resulting in unexpected application behavior and potentially untoward outcomes.

Assessment Notes

Follow-up Actions

Person Responsible for Follow-up Action

Reset

Suggested Sources of Input

1. Clinicians, support staff, and/or clinical administration
2. EHR developer

Strength of Recommendation

Medium

Implementation Guidance

- Create strategies to calculate system response times. One such strategy is to create an application to submit a simple medication order for a "test patient" every day of the year at midnight and run a simple automated query to request this order's details be displayed on a workstation in a clinical setting every minute for the next 24 hours (i.e., 1440 times). Mean system response time is the time from order being requested until the time the details are available. Functional system downtime can be defined by any hourly mean response time greater than 5 seconds or 3 standard deviations above the mean.[48]
- The organization creates easy mechanisms for users to report slow system response time to the IT Helpdesk.

Recommended Practice- Review Downtimes

3.3 Conduct an in-depth review of unexpected system downtimes lasting over 24 hours using root-cause or failure modes and effects analysis or similar approaches.[49]

Checklist

Implementation Status

☐ **EHR Limitation**

Rationale for Practice or Risk Assessment

Experiences with an unexpected downtime over 24 hours are likely to provide learning opportunities for future management and prevention of similar events.

Assessment Notes

Follow-up Actions

Person Responsible for Follow-up Action

Reset

Suggested Sources of Input

1. Clinicians, support staff, and/or clinical administration
2. EHR developer

Strength of Recommendation

Medium

Implementation Guidance

- The organization convenes a multi-disciplinary group of clinicians and IT professionals to review the event and its management, identify potential root causes, and discuss future prevention or mitigating procedures.
- The organization reviews the effect of extended downtimes on patient care quality, safety, and/or timeliness.[4]
- The organization considers consulting with additional experts in IT system reliability to review and report on recommendations for improvements in key system components, configurations, and policies and procedures.

References

1. Kilbridge P. Computer crash--lessons from a system failure. N Engl J Med. 2003;348(10):881–882. https://pubmed.ncbi.nlm.nih.gov/12621131/. doi: 10.1056/NEJMp030010; PMID: 12621131.

2. Hanuscak TL, Szeinbach SL, Seoane-Vazquez E, Reichert BJ, McCluskey CF. Evaluation of causes and frequency of medication errors during information technology downtime. Am J Health Syst Pharm. 2009;66(12):1119–1124. https://pubmed.ncbi.nlm.nih.gov/19498129/. doi: 10.2146/ajhp080389; PMID: 19498129.

3. McBiles M, Chacko AK. Coping with PACS downtime in digital radiology. J Digit Imaging. 2000;13(3):136–142. https://www.ncbi.nlm.nih.gov/pmc/articles/PMC3452972/. doi: 10.1007/BF03168387; PMID: 15359753; PMC3452972.

4. Wang Y, Coiera E, Gallego B, et al. Measuring the effects of computer downtime on hospital pathology processes. J Biomed Inform. 2016;59:308–315. https://pubmed.ncbi.nlm.nih.gov/26732996/. doi: 10.1016/j.jbi.2015.12.016; PMID: 26732996.

5. Sittig DF, Singh H. Electronic health records and national patient-safety goals. N Engl J Med. 2012;367(19):1854–1860. https://pubmed.ncbi.nlm.nih.gov/23134389/. doi: 10.1056/NEJMsb1205420; PMID: 23134389; PMC3690003.

6. Lyon R, Jones A, Burke R, Baysari MT. What goes up, must come down: A state-of-the-art electronic health record downtime and uptime procedure in a metropolitan health setting. Appl Clin Inform. 2023;14(3):513–520. https://pubmed.ncbi.nlm.nih.gov/37406674/. doi: 10.1055/s-0043-1768995; PMID: 37406674; PMC10322225.

7. Lee OF, Guster DC. Virtualized disaster recovery model for large scale hospital and healthcare systems. International Journal of Healthcare Information Systems and Informatics (IJHISI). 2010;5(3):69–81. https://www.igi-global.com/article/virtualized-disaster-recovery-model-large/46093.

8. Martin G, Ghafur S, Cingolani I, et al. The effects and preventability of 2627 patient safety incidents related to health information technology failures: A retrospective analysis of 10 years of incident reporting in england and wales. Lancet Digit Health. 2019;1(3):e127–e135. https://pubmed.ncbi.nlm.nih.gov/33323263/. doi: 10.1016/S2589-7500(19)30057-3; PMID: 33323263.

9. Sittig DF, Singh H. Defining health information technology-related errors: New developments since to err is human. Arch Intern Med. 2011;171(14):1281–1284. https://pubmed.ncbi.nlm.nih.gov/21788544/. doi: 10.1001/archinternmed.2011.327; PMID: 21788544; PMC3677061.

10. Dooling JA. Meaningful use and disaster infrastructure Q&A. J AHIMA. 2013;84(10):64–65. https://pubmed.ncbi.nlm.nih.gov/24245091/; PMID: 24245091.

11. The Joint Commission. Prepublication requirements | new and revised emergency management (EM) requirements. . https://www.jointcommission.org/standards/prepublication-standards/new-and-revised-emergency-management-standards/. Updated 2023. Accessed Jun 6, 2024.

12. Son C, Larsen E, Sasangohar F, Peres S. Opportunities and challenges for resilient hospital incident management: Case study of a hospital's response to hurricane harvey. Journal of Critical Infrastructure Policy. 2020. https://www.jcip1.org/uploads/1/3/6/5/136597491/opportunties_and_challenges__jcip_1.1.pdf. doi: 10.18278/jcip.1.1.7.

13. Centers for Medicare & Medicaid Services (U.S.). Categorical waiver – health care microgrid systems (HCMSs) | CMS. https://www.cms.gov/medicare/provider-enrollment-and-certification/surveycertificationgeninfo/policy-and-memos-states/categorical-waiver-health-care-microgrid-systems-hcmss. Updated 2023. Accessed Jun 6, 2024.

14. Hiller M, Bone E, Timmins M. Healthcare system resiliency: The case for taking disaster plans further - part 2. Journal of business continuity & emergency planning. 2015;8:356–75. https://www.researchgate.net/publication/277085500_Healthcare_system_resiliency_The_case_for_taking_disaster_plans_further_-_Part_2.

References

15. Hines E, Reid CE. Hospital preparedness, mitigation, and response to hurricane harvey in harris county, texas. Disaster Med Public Health Prep. 2021;17:e18. https://pubmed.ncbi.nlm.nih.gov/34180391/. doi: 10.1017/dmp.2021.146; PMID: 34180391; PMC8896825.

16. Lei J, Guan P, Gao K, et al. Characteristics of health IT outage and suggested risk management strategies: An analysis of historical incident reports in china. Int J Med Inform. 2014;83(2):122–130. https://pubmed.ncbi.nlm.nih.gov/24246272/. doi: 10.1016/j.ijmedinf.2013.10.006; PMID: 24246272.

17. McKinney M. Technology. what happens when the IT system goes down? Hosp Health Netw. 2007;81(12):14. https://pubmed.ncbi.nlm.nih.gov/18260574/; PMID: 18260574.

18. Patient safety guidance for electronic health record downtime, recommendations of the electronic health record downtime task force. Academic Medical Center Patient Safety Organization (AMC PSO) EHR Downtime Task Force, Harvard Risk Management Foundation, Boston MA. 2017. https://flbog.sip.ufl.edu/wp-content/uploads/2019/11/AMC-PSO-EHR-Downtime.pdf.

19. Sittig DF, Gonzalez D, Singh H. Contingency planning for electronic health record-based care continuity: A survey of recommended practices. Int J Med Inform. 2014;83(11):797–804. https://pubmed.ncbi.nlm.nih.gov/25200197/. doi: 10.1016/j.ijmedinf.2014.07.007; PMID: 25200197.

20. T. C. Piliouras, R. J. Suss, P. L. Yu. Digital imaging & electronic health record systems: Implementation and regulatory challenges faced by healthcare providers. . 2015:1–6. https://ieeexplore.ieee.org/document/7160179. doi: 10.1109/LISAT.2015.7160179.

21. Schweitzer EJ. Reconciliation of the cloud computing model with US federal electronic health record regulations. J Am Med Inform Assoc. 2012;19(2):161–165. https://pubmed.ncbi.nlm.nih.gov/21727204/. doi: 10.1136/amiajnl-2011-000162; PMID: 21727204; PMC3277612.

22. Schackow TE, Palmer T, Epperly T. EHR meltdown: How to protect your patient data. Fam Pract Manag. 2008;15(6):3. https://pubmed.ncbi.nlm.nih.gov/18595558/; PMID: 18595558.

23. Thomas J, Galligher G. Improving backup system evaluations in information security risk assessments to combat ransomware. Compute and Information Science. 2018;11(1). https://www.ccsenet.org/journal/index.php/cis/article/view/72481. doi: 10.5539/cis.v11n1p14.

24. Larsen E, Fong A, Wernz C, Ratwani RM. Implications of electronic health record downtime: An analysis of patient safety event reports. J Am Med Inform Assoc. 2018;25(2):187–191. https://www.ncbi.nlm.nih.gov/pmc/articles/PMC7647128/. doi: 10.1093/jamia/ocx057; PMID: 28575417; PMC7647128.

25. Larsen EP, Haskins Lisle A, Law B, Gabbard JL, Kleiner BM, Ratwani RM. Identification of design criteria to improve patient care in electronic health record downtime. J Patient Saf. 2021;17(2):90–94. https://pubmed.ncbi.nlm.nih.gov/30747861/. doi: 10.1097/PTS.0000000000000580; PMID: 30747861.

26. Oral B, Cullen RM, Diaz DL, Hod EA, Kratz A. Downtime procedures for the 21st century: Using a fully integrated health record for uninterrupted electronic reporting of laboratory results during laboratory information system downtimes. Am J Clin Pathol. 2015;143(1):100–104. https://pubmed.ncbi.nlm.nih.gov/25511148/. doi: 10.1309/AJCPM0O7MNVGCEVT; PMID: 25511148.

27. Gecomo JG, Klopp A, Rouse M, Online Journal of Nursing Informatics contributors. Implementation of an evidence-based electronic health record (EHR) downtime readiness and recovery plan | HIMSS. On - Line Journal of Nursing Informatics (OJNI). 2020;24(1). https://www.himss.org/resources/implementation-evidence-based-electronic-health-record-ehr-downtime-readiness-and.

References

28. Genes N, Chary M, Chason KW. An academic medical center's response to widespread computer failure. Am J Disaster Med. 2013;8(2):145–150. https://pubmed.ncbi.nlm.nih.gov/24352930/. doi: 10.5055/ajdm.2013.0121; PMID: 24352930.

29. Poterack KA, Gottlieb O. Are you ready for EHR downtime? questions to ask. ASA Monitor. 2016;80(2):30–31. https://pubs.asahq.org/monitor/article-abstract/81/4/30/5896/Paper-Charting-Anesthetics-Forgotten-But-Not-Gone?redirectedFrom=fulltext.

30. Electronic prescribing of controlled substances (EPCS). https://portal.ct.gov/dcp/drug-control-division/drug-control/epcs-information-page. Accessed Jun 17, 2024.

31. Nelson NC. Downtime procedures for a clinical information system: A critical issue. J Crit Care. 2007;22(1):45–50. https://pubmed.ncbi.nlm.nih.gov/17371746/. doi: 10.1016/j.jcrc.2007.01.004; PMID: 17371746.

32. Menon S, Singh H, Meyer AND, Belmont E, Sittig DF. Electronic health record-related safety concerns: A cross-sectional survey. J Healthc Risk Manag. 2014;34(1):14–26. https://pubmed.ncbi.nlm.nih.gov/25070253/. doi: 10.1002/jhrm.21146; PMID: 25070253.

33. Joint Commission International. 6th edition in-depth: Planned and unplanned downtime (part 2: Data recovery tactics). https://store.jointcommissioninternational.org/assets/3/7/January_JCInsight_2018.pdf. Updated 2018. Accessed Jun 7, 2024.

34. American Medical Association. Guidelines for developing EHR downtime procedures. https://view.officeapps.live.com/op/view.aspx?src=https%3A%2F%2Fedhub.ama-assn.org%2Fdata%2FJournals%2Fsteps-forward%2F937327%2F10.1001stepsforward.2017.0017supp3.docx&wdOrigin=BROWSELINK. Updated 2017. Accessed Jun 8, 2024.

35. National Institute of Mental Health, Department of Clinical Research Informatics (DCRI) Clinical Center. Downtime policy and procedure for unavailability of electronic clinical systems. https://www.cc.nih.gov/sites/nihinternet/files/internet-files/dcri/pdfs/downtime_policy.pdf. Accessed Jun 7, 2024.

36. Scholl MA, Stine K, Hash J, et al. An introductory resource guide for implementing the health insurance portability and accountability act (HIPAA) security rule. Archived NIST Technical Series Publication. 2008(Special Publication 800-66). https://www.govinfo.gov/content/pkg/GOVPUB-C13-a8e8580b3e0cf851b2f274741bd28878/pdf/GOVPUB-C13-a8e8580b3e0cf851b2f274741bd28878.pdf.

37. Fernández MT, Gómez AR, Santojanni AM, Cancio AH, Luna DR, Benítez SE. Electronic health record system contingency plan coordination: A strategy for continuity of care considering users' needs. Stud Health Technol Inform. 2015;216:472–476. https://pubmed.ncbi.nlm.nih.gov/26262095/; PMID: 26262095.

38. Healthcare & Public Health Sector Coordinating Council. Health industry cybersecurity practices: Managing threats and protecting patients. https://405d.hhs.gov/Documents/HICP-Main-508.pdf. Updated 2023. Accessed Jun 8, 2024.

39. U.S. Department of Health and Human Services, Office for Civil Rights. Fact sheet: Ransomware and HIPAA. https://www.hhs.gov/hipaa/for-professionals/security/guidance/cybersecurity/ransomware-fact-sheet/index.html. Updated 2021. Accessed Jun 7, 2024.

40. Sittig DF, Singh H. A socio-technical approach to preventing, mitigating, and recovering from ransomware attacks. Appl Clin Inform. 2016;7(2):624–632. https://www.ncbi.nlm.nih.gov/pmc/articles/PMC4941865/. doi: 10.4338/ACI-2016-04-SOA-0064.

41. Greenberg A. Hackers behind the change healthcare ransomware attack just received a $22 million payment. Wired. 2024. https://www.wired.com/story/alphv-change-healthcare-ransomware-payment/.

References

42. Souppaya M, Scarfone K. National institute of standards and technology special publication 800-83 revision 1, guide to malware incident prevention and handling for desktops and laptops. Natl. Inst. Stand. Technol. Spec. Publ. 2013;800-83r1:47. https://csrc.nist.rip/external/nvlpubs.nist.gov/nistpubs/SpecialPublications/NIST.SP.800-83r1.pdf. doi: 10.6028/NIST.SP.800-83r1.

43. Wright A, Sittig DF. Security threat posed by USB-based personal health records. Ann Intern Med. 2007;146(4):314–315. https://pubmed.ncbi.nlm.nih.gov/17310061/. doi: 10.7326/0003-4819-146-4-200702200-00020; PMID: 17310061.

44. Gordon WJ, Wright A, Aiyagari R, et al. Assessment of employee susceptibility to phishing attacks at US health care institutions. JAMA Netw Open. 2019;2(3):e190393. pubmed.ncbi.nlm.nih.gov/30848810/. doi: 10.1001/jamanetworkopen.2019.0393; PMID: 30848810; PMC6484661.

45. Marsh-Armstrong B, Pacheco F, Dameff C, Tully J. Design and pilot study of a high-fidelity medical simulation of a hospital-wide cybersecurity attack. Res Sq. 2024:rs.3.rs–3959502. https://pubmed.ncbi.nlm.nih.gov/38645079/. doi: 10.21203/rs.3.rs-3959502/v1; PMID: 38645079; PMC11030511.

46. Angafor G, Yevseyeva I, Maglaras L. MalAware: A tabletop exercise for malware security awareness education and incident response training. Internet of Things and Cyber-Physical Systems. 2024;4:280–292. https://www.sciencedirect.com/science/article/pii/S2667345224000063. doi: 10.1016/j.iotcps.2024.02.003.

47. Blecker S, Austrian JS, Shine D, Braithwaite RS, Radford MJ, Gourevitch MN. Monitoring the pulse of hospital activity: Electronic health record utilization as a measure of care intensity. J Hosp Med. 2013;8(9):513–518. https://pubmed.ncbi.nlm.nih.gov/23908140/. doi: 10.1002/jhm.2068; PMID: 23908140.

48. Sittig DF, Campbell E, Guappone K, Dykstra R, Ash JS. Recommendations for monitoring and evaluation of in-patient computer-based provider order entry systems: Results of a delphi survey. AMIA Annu Symp Proc. 2007;2007:671–675. https://www.ncbi.nlm.nih.gov/pmc/articles/PMC2655851/.

49. Yusof MM, Takeda T, Shimai Y, Mihara N, Matsumura Y. Evaluating health information systems-related errors using the human, organization, process, technology-fit (HOPT-fit) framework. Health Informatics J. 2024;30(2):14604582241252763. https://pubmed.ncbi.nlm.nih.gov/38805345/. doi: 10.1177/14604582241252763; PMID: 38805345.
