# Supplementary material for: Revisions to the Safety Assurance Factors for Electronic Health Record Resilience (SAFER) Guides to update national recommendations for safe use of electronic health records
Source: J Am Med Inform Assoc. 2025 Apr 12;32(4):755–60. doi: 10.1093/jamia/ocaf018 (PMC12005625; doi:10.1093/jamia/ocaf018)
Supplement: ocaf018_Supplementary_Data [file ocaf018_supplementary_data.zip › ocaf018_Supplementary_Data/CPOE Final.pdf]

## Self-Assessment

# Computerized Provider Order Entry with Decision Support

## General Instructions for the SAFER Self-Assessment Guides

The Safety Assurance Factors for EHR Resilience (SAFER) guides are designed to help healthcare organizations conduct proactive self-assessments to evaluate the safety and effectiveness of their electronic health record (EHR) implementations. The 2025 SAFER guides have been updated and streamlined to focus on the highest risk, most commonly occurring issues that can be addressed through technology or practice changes to build system resilience in the following areas:

- Organizational Responsibilities
- Patient Identification
- Clinician Communication
- Test Results Reporting and Follow-up
- Computerized Provider Order Entry with Decision Support
- Systems Management
- Contingency Planning
- High Priority Practices - A collection of 16 Recommendations from the other 7 Guides

Each of the eight SAFER Guides begins with a Checklist of recommended practices. The downloadable SAFER Guides provide fillable circles that can be used to indicate the extent to which each recommended practice has been implemented in the organization using a 5-point Likert scale. The Practice Worksheet gives a rationale for the practice and provides examples of how to implement each recommended practice. It contains fields to record team member involvement and follow-up actions based on the assessment. The Worksheet also lists the stakeholders who can provide input to assess each practice (sources of input). In addition to the downloadable version, the content of each SAFER Guide, with interactive references and supporting materials, can also be viewed on ONC's website at: https://www.healthit.gov/topic/safety/safer-guides.

The SAFER guides are based on the best available (2024) evidence from the literature and consensus expert opinion. Subject matter experts in patient safety, informatics, quality improvement, risk management, human factors engineering, and usability developed them. Furthermore, they were reviewed by an external group of practicing clinicians, informaticians, and information technology professionals.

Each guide contains between 6 and 18 recommended practices including its rationale, implementation guidance, and evidence level. The recommended practices in the SAFER Guides are intended to be useful for all EHR users. However, every organization faces unique circumstances and may implement a particular recommended practice differently. As a result, some of the specific implementation guidance in the SAFER Guides for recommended practices may not be applicable to an organization.

The High Priority Practices guide consists of 16 of the most important and relevant recommendations selected from the other 7 guides. It is designed for practicing clinicians to help them understand, implement, and support EHR safety and safe use within their organization. The other seven guides consist of 88 unique recommendations that are relevant for all healthcare providers and organizations.

The SAFER Guides are designed in part to help deal with safety concerns created by the continuously changing sociotechnical landscape that healthcare organizations face. Therefore, changes in technology, clinical practice standards, regulations, and policy should be taken into account when using the SAFER Guides. Periodic self-assessments using the SAFER Guides may also help organizations identify areas where it is particularly important to address the implications of these practice or EHR-based changes for the safety and safe use of EHRs. Ultimately, the goal is to improve the overall safety of our health care system and improve patient outcomes.

The SAFER Guides are not intended to be used for legal compliance purposes, and implementation of a recommended practice does not guarantee compliance with the HIPAA Security or Privacy Rules, Medicare or Medicaid Conditions of Participation, or any other laws or regulations. The SAFER Guides are for informational purposes only and are not intended to be an exhaustive or definitive source. They do not constitute legal advice. Users of the SAFER Guides are encouraged to consult with their own legal counsel regarding compliance with Medicare or Medicaid program requirements, and any other laws.

For additional information on Medicare and Medicaid program requirements, please visit the Centers for Medicare & Medicaid Services website at www.cms.gov. For more information on HIPAA, please visit the HHS Office for Civil Rights website at www.hhs.gov/ocr.

## Self-Assessment

# Computerized Provider Order Entry with Decision Support

## Introduction

The Computerized Provider Order Entry with Decision Support SAFER Guide identifies recommended safety practices associated with the design, implementation, use, and monitoring of orders and clinical decision support (CDS). This includes order structure, mapping, libraries, alerts, and warnings that users rely on during patient care. This guide focuses on strategies for optimizing safety during clinical decision-making and order-entry processes.

Providers rely on EHR technology to help them navigate the complexities of patient care, including diagnosis and treatment. This requires accurate and reliable CPOE functionality, with navigable pick lists based on structured orders that are mapped to standard vocabularies to assure interoperability. CDS suggestions should be unambiguous and appropriate and should not generate excessive warnings or irrelevant alerts.

Unsafe practices, including inappropriate use of free-text orders, bloated and inaccurate allergy lists, or vague alert wording, can overwhelm providers and potentially result in preventable patient harm. Equally important is the ability to configure decision support to meet organizational safety and quality standards and to address the needs of specialized patient populations.

CPOE and CDS should be developed and approved by practicing clinical staff who are most closely impacted by the functionality. Physicians and other ordering providers, pharmacists, and nurses representing a variety of clinical specialties can advise not only on the relevance of functionality, but also on workflow integration across disciplines, settings, and patient populations.

Completing the self-assessment in the Computerized Provider Order Entry with Decision Support SAFER Guide requires the engagement of a multidisciplinary team including ordering providers with decision-making responsibility who should review this guide and include other clinical, technical, and informatics staff. This may require people both within and outside of the healthcare organization.

Collaboration between multidisciplinary clinicians and other staff members while completing the self-assessment in this guide will enable an accurate snapshot of the safety of the organization’s CPOE and CDS content and functionality. This collaboration should lead to a consensus about the organization’s future path to optimize EHR-related safety and quality: setting priorities among the recommended practices not yet addressed, ensuring a plan is in place to maintain recommended practices already in place, dedicating the required resources to make necessary improvements, and working together to mitigate the CPOE- and CDS-related safety risks introduced by the EHR.

Self-Assessment

# Computerized Provider Order Entry with Decision Support

## Table of Contents

| | |
|---|---|
| General Instructions | 1 |
| Introduction | 2 |
| About the Checklist | 5 |
| Checklist | 6 |
| Team Worksheet | 8 |
| About the Recommended Practice Worksheets | 9 |
| Recommended Practice Worksheets | 10 |
| References | 20 |

# Authors and Peer Reviewers

The SAFER Self-Assessment Guides were developed by health IT safety researchers and informatics experts whose contributions are acknowledged as follows:

Primary authors who contributed to the development of all guides:

**Trisha Flanagan, RN, MSN, CPPS,** Health Informatics Nurse, Center for Innovations in Quality, Effectiveness and Safety, Michael E. DeBakey Veterans Affairs Medical Center, Houston TX

**Hardeep Singh, MD, MPH,** Co-Chief, Health Policy, Quality and Informatics Program, Center for Innovations in Quality, Effectiveness and Safety and Professor of Medicine at the Michael E. DeBakey Veterans Affairs Medical Center and Baylor College of Medicine, Houston, TX

**Dean F. Sittig MS, PhD, FACMI, FAMIA, FHIMSS, FIAHSI,** Professor of Biomedical Informatics, Department of Clinical and Health Sciences, McWilliams School of Biomedical Informatics, University of Texas Health Science Center at Houston, TX and Informatics Review LLC, Lake Oswego, OR

Support staff for the primary authorship team

**Rosann Cholankeril, MD, MPH,** Center for Innovations in Quality, Effectiveness and Safety, Michael E. DeBakey Veterans Affairs Medical Center and Baylor College of Medicine

**Sara Ehsan, MBBS, MPH,** Center for Innovations in Quality, Effectiveness and Safety, Michael E. DeBakey Veterans Affairs Medical Center and Baylor College of Medicine

Additional authors who contributed to at least one guide:

**Jason S. Adelman, MD, MS,** (Patient ID) Chief Patient Safety Officer & Associate Chief Quality Officer, Executive Director, Patient Safety Research, Co-Director, Patient Safety Research Fellowship in Hospital Medicine, New York-Presbyterian Hospital/Columbia University Irving Medical Center, New York, NY

**Daniel R. Murphy, MD, MBA,** (Clinician Communication, Test Results) Chief Quality Officer, Baylor Medicine, Houston, TX

**Patricia Sengstack, DNP, NI-BC, FAAN, FACMI,** (Organizational Responsibilities) Senior Associate Dean for Informatics, Director, Nursing Informatics Specialty Program, Vanderbilt University School of Nursing, Vanderbilt University, Nashville, TN

Additional contributors who provided feedback on various guides or parts of guides

**Miriam Callahan, MD (Patient ID)**
**David C. Classen, MD (CPOE, AI recommendation)**
**Anne Grauer, MD, MS (Patient ID)**
**Ing Haviland (Patient ID)**
**Amanda Heidemann, MD (All Guides)**
**I-Fong Sun Lehman, DrPH, MS (Patient ID)**
**Christoph U. Lehmann, MD (AI recommendation)**
**Christopher A. Longhurst, MD, MS (AI recommendation)**
**Edward R. Melnick, MD (Clinician Communication)**
**Robert E. Murphy, MD (Organizational Responsibilities)**
**Ryan P. Radecki, MD, MS (AI recommendation)**
**Raj Ratwani, PhD (AI recommendation)**
**Trent Rosenbloom, MD (Clinician Communication)**
**Lisa Rotenstein, MD (Clinician Communication)**
**Hojjat Salmasian, MD, PhD (All Guides)**
**Richard Schreiber, MD (CPOE)**
**Danny Sands, MD (Clinician Communication)**
**Debora Simmons, PhD, RN (Organizational Responsibilities)**
**Carina Sirochinsky (Patient ID)**
**Neha Thummala, MPH (Patient ID)**
**Emma Weatherford (Patient ID)**
**Adam Wright, PhD (CPOE)**
**Andrew Zimolzak, MD, MMSc (Test Results, Clinician Communication)**

This guide was developed under the contract Unintended Consequences of Health IT and Health Information Exchange, Task Order HHSP23337003T/HHSP23320095655WC.

The ATSP/ONC composite mark is a mark of the U.S. Department of Health and Human Services. The contents of the publication or project are solely the responsibility of the authors and do not necessarily represent the official views of the U.S. Department of Health and Human Services, Asssistant Secretary for the Technology Policy/Office of the National Coordinator for Health Information Technology.

The *Checklist* is structured as a quick way to enter and print your self-assessment.

Select the level of implementation achieved by your organization for each Recommended Practice. Your Implementation Status will be reflected on the Recommended Practice Worksheet in this PDF. The implementation status scales are as followed:

**Not Implemented – (0%)** The organization has not implemented this recommendation.

**Making Progress (1 - 30%)** The organization is in the early or pilot phase of implementing this recommendation as evidenced by following or adopting less than 30% of the implementation guidance.

**Halfway there (31 – 60%)** The organization is implementing this recommendation and is following or has adopted approximately half of the implementation guidance.

**Substantial Progress (61-90%)** The organization has nearly implemented this recommendation and is following or has adopted much of the implementation guidance.

**Fully Implemented (91-100%)** The organization follows this recommendation, and most implementation guidance is followed consistently and widely adopted.

The organization should check the following box if there are some limitations with the current version of their EHR that preclude them from fully implementing this recommendation.

**EHR Limitation -** The EHR does not offer the features/functionality required to fully implement this recommendation or the implementation guidance.

The *Domain* associated with the *Recommended Practice(s)* appears at the top of the column

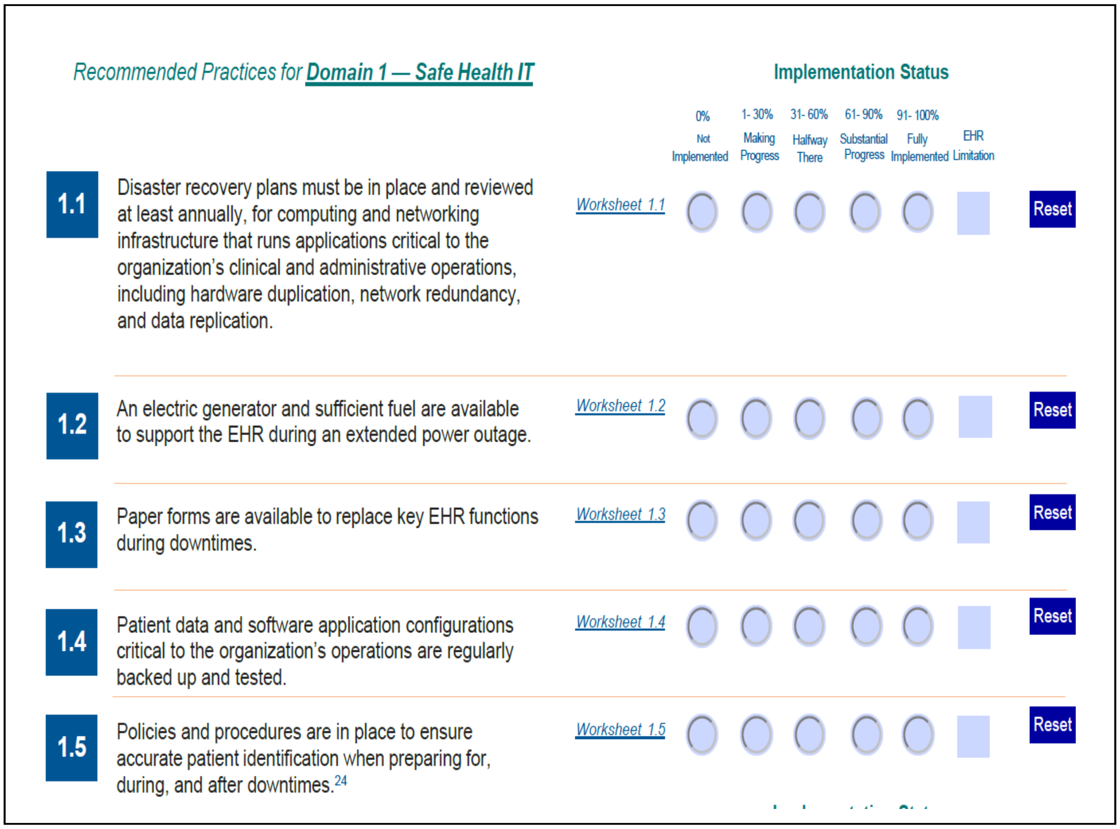

Recommended Practices for **Domain 1 — Safe Health IT**

Implementation Status

| | | | 0% Not Implemented | 1-30% Making Progress | 31-60% Halfway There | 61-90% Substantial Progress | 91-100% Fully Implemented | EHR Limitation | |
|---|---|---|---|---|---|---|---|---|---|
| 1.1 | Disaster recovery plans must be in place and reviewed at least annually, for computing and networking infrastructure that runs applications critical to the organization's clinical and administrative operations, including hardware duplication, network redundancy, and data replication. | Worksheet 1.1 | | | | | | | Reset |
| 1.2 | An electric generator and sufficient fuel are available to support the EHR during an extended power outage. | Worksheet 1.2 | | | | | | | Reset |
| 1.3 | Paper forms are available to replace key EHR functions during downtimes. | Worksheet 1.3 | | | | | | | Reset |
| 1.4 | Patient data and software application configurations critical to the organization's operations are regularly backed up and tested. | Worksheet 1.4 | | | | | | | Reset |
| 1.5 | Policies and procedures are in place to ensure accurate patient identification when preparing for, during, and after downtimes.[24] | Worksheet 1.5 | | | | | | | Reset |

The *Recommended Practice(s)* for the topic appears below the associated *Domain*.

To the right of reach *Recommended Practice* is a link to the Recommended Practice Worksheet in this PDF.

The *Worksheet* provides guidance on implementing the practice.

## Recommended Practices for Domain 1 — Safe Health IT

| | | | Implementation Status | | | | | |
|---|---|---|---|---|---|---|---|---|
| | | | 0% Not Implemented | 1-30% Making Progress | 31-60% Halfway There | 61-90% Substantial Progress | 91-100% Fully Implemented | EHR Limitation |
| 1.1 | Use of structured orders is maximized to the extent possible for medications, diagnostic testing, procedures, referrals, and care transitions (e.g., patient handoffs between settings, discharges, and admissions). | Worksheet 1.1 | ◯ | ◯ | ◯ | ◯ | ◯ | Reset |
| 1.2 | Allergies to medications, contrast agents, and latex are entered and updated as structured data before order entry and environmental allergens are included in the problem list. | Worksheet 1.2 | ◯ | ◯ | ◯ | ◯ | ◯ | Reset |
| 1.3 | CDS content and configuration (including but not limited to alerts, order sets, preventative care, and screening reminders) are based on current best practice guidance that is developed, reviewed, and updated by clinical staff representing a variety of specialties and disciplines. | Worksheet 1.3 | ◯ | ◯ | ◯ | ◯ | ◯ | Reset |

## Recommended Practices for Domain 2 — Using Health IT Safely

| | | | Implementation Status | | | | | |
|---|---|---|---|---|---|---|---|---|
| | | | 0% Not Implemented | 1-30% Making Progress | 31-60% Halfway There | 61-90% Substantial Progress | 91-100% Fully Implemented | EHR Limitation |
| 2.1 | CDS alerts and reminders provide unambiguous guidance in the correct clinical context at relevant points in the workflow. Alerts and reminders are informative, actionable, and judiciously limited to the most significant, patient-specific notifications. | Worksheet 2.1 | ◯ | ◯ | ◯ | ◯ | ◯ | Reset |
| 2.2 | EHR ordering and decision support functionality is configured to provide safe, relevant, and effective content for pediatric, geriatric, and other patient populations requiring special considerations for their conditions and diagnoses. | Worksheet 2.2 | ◯ | ◯ | ◯ | ◯ | ◯ | Reset |
| 2.3 | EHR enables the documentation of additional safeguards (e.g., pharmacy review, supervising clinician signoff, independent double check) to reduce the possibility of patient harm from high-risk medications. | Worksheet 2.3 | ◯ | ◯ | ◯ | ◯ | ◯ | Reset |
| 2.4 | Referrals and transition of care orders are standardized, structured, and supported by functionality that tracks the order to completion. | Worksheet 2.4 | ◯ | ◯ | ◯ | ◯ | ◯ | Reset |

*Recommended Practices for* ***Domain 3 — Monitoring Safety***

| | | | Implementation Status | | | | | |
|---|---|---|---|---|---|---|---|---|
| | | | 0% Not Implemented | 1-30% Making Progress | 31-60% Halfway There | 61-90% Substantial Progress | 91-100% Fully Implemented | EHR Limitation |
| **3.1** | Key metrics related to CPOE and CDS functionality are defined, monitored, and used to optimize safety and efficiency. | *Worksheet 3.1* | ◯ | ◯ | ◯ | ◯ | ◯ | Reset |

> Table of Contents | > About the Checklist | >Team Worksheet | > About the Practice Worksheets | >Practice Worksheets

Clinicians should complete this self-assessment and evaluate potential health IT-related patient safety risks addressed by this specific SAFER Guide within the context of your particular healthcare organization.

This Team Worksheet is intended to help organizations document the names and roles of the self-assessment team, as well as individual team members' activities. Typically, team members will be drawn from a number of different areas within your organization, and in some instances, from external sources. The suggested Sources of Input section in each Recommended Practice Worksheet identifies the types of expertise or services to consider engaging. It may be particularly useful to engage specific clinician and other leaders with accountability for safety practices identified in this guide.

The Worksheet includes fillable boxes that allow you to document relevant information. The Assessment Team Leader box allows documentation of the person or persons responsible for ensuring that the self-assessment is completed. The section labeled Assessment Team Members enables you to record the names of individuals, departments, or other organizations that contributed to the self-assessment. The date that the self-assessment is completed can be recorded in the Assessment Completion Date section and can also serve as a reminder for periodic reassessments. The section labeled Assessment Team Notes is intended to be used, as needed, to record important considerations or conclusions arrived at through the assessment process. This section can also be used to track important factors such as pending software updates, vacant key leadership positions, resource needs, and challenges and barriers to completing the self-assessment or implementing the Recommended Practices in this SAFER Guide.

Assessment Team Leader

Assessment Completion Date

Assessment Team Members

Assessment Team Notes

Reset Page

Each *Recommended Practice Worksheet* provides guidance on implementing a specific *Recommended Practice*, and allows you to enter and print information about your self-assessment.

The Suggested Sources of Input section indicates categories of personnel who can provide information to help evaluate your level of implementation.

The *Rationale* section provides guidance about "why" the safety activities are needed.

The Strength of Recommendation section provides an estimate of the strength of evidence available in the scientific literature, or states that it is "required" due to a federal rule, regulation, or conditions of participation, for each recommendation.

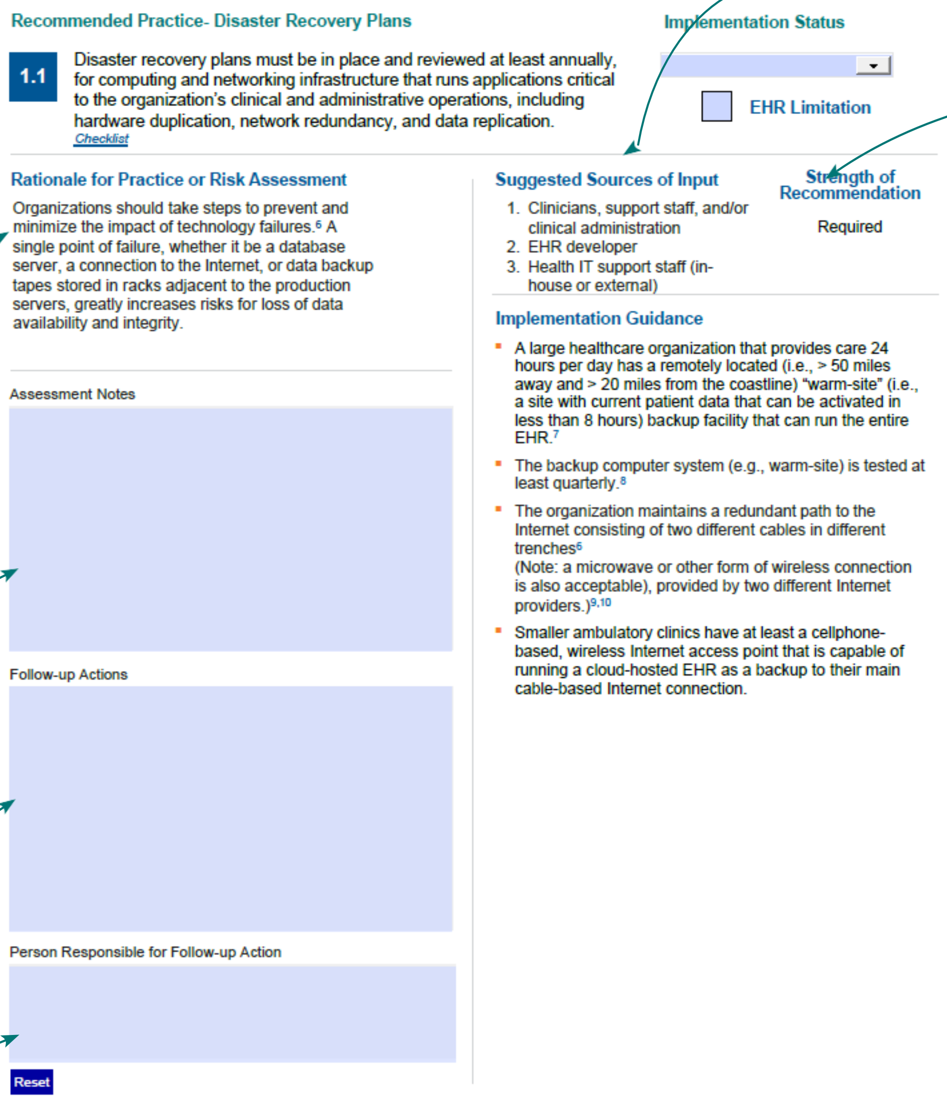

Recommended Practice- Disaster Recovery Plans

**1.1** Disaster recovery plans must be in place and reviewed at least annually, for computing and networking infrastructure that runs applications critical to the organization's clinical and administrative operations, including hardware duplication, network redundancy, and data replication.
Checklist

Implementation Status

EHR Limitation

Rationale for Practice or Risk Assessment

Organizations should take steps to prevent and minimize the impact of technology failures.[6] A single point of failure, whether it be a database server, a connection to the Internet, or data backup tapes stored in racks adjacent to the production servers, greatly increases risks for loss of data availability and integrity.

Suggested Sources of Input

1. Clinicians, support staff, and/or clinical administration
2. EHR developer
3. Health IT support staff (in-house or external)

Strength of Recommendation

Required

Implementation Guidance

- A large healthcare organization that provides care 24 hours per day has a remotely located (i.e., > 50 miles away and > 20 miles from the coastline) "warm-site" (i.e., a site with current patient data that can be activated in less than 8 hours) backup facility that can run the entire EHR.[7]
- The backup computer system (e.g., warm-site) is tested at least quarterly.[8]
- The organization maintains a redundant path to the Internet consisting of two different cables in different trenches[6] (Note: a microwave or other form of wireless connection is also acceptable), provided by two different Internet providers.)[9,10]
- Smaller ambulatory clinics have at least a cellphone-based, wireless Internet access point that is capable of running a cloud-hosted EHR as a backup to their main cable-based Internet connection.

Assessment Notes

Follow-up Actions

Person Responsible for Follow-up Action

Reset

Enter any notes about your self-assessment.

The Implementation Guidance section lists potentially useful practices or scenarios to inform your assessment and implementation of the specific *Recommended Practice*.

Enter any follow-up activities required.

Enter the name of the person responsible for the follow-up activities.

## Recommended Practice - Structured Data

**1.1** Use of structured orders is maximized to the extent possible for medications, diagnostic testing, procedures, referrals, and care transitions (e.g., patient handoffs between settings, discharges, and admissions).

Checklist

### Implementation Status

EHR Limitation

### Rationale for Practice or Risk Assessment

Clinical decision support alerting requires structured data elements. Free-text entries may be misspelled or include inconsistent wording or unsafe abbreviations, and will not trigger automated interaction checking or other warnings to ordering providers, pharmacists, nurses, and other healthcare staff. This may result in preventable adverse drug reactions or other potentially harmful outcomes.

Assessment Notes

Follow-up Actions

Person Responsible for Follow-up Action

Reset Page

### Suggested Sources of Input

1. Clinicians
2. Clinician support staff
3. Clinical administration
4. EHR developer

### Strength of Recommendation

Medium

### Implementation Guidance

- A standard, controlled vocabulary of orderable items is available and used.
- There is a policy designed to support the use of structured orders while also describing specific scenarios in which free-text orders may be allowed.[1]
- There is a policy that requires regular review of free text orders and free text special instructions to identify whether additional education is needed for specific providers (e.g., those who use free text often) and to identify gaps in the structured orders or special instruction fields that require mitigation, for example, a new medication, laboratory test, or special handling instruction that is not yet available in a structured format.[2]
- User settings may be customized to optimize the display of order preference lists.[3]
- There is a mechanism for alerting providers who enter free-text orders that a structured order exists.
- In addition to allergens, the allergy list consists of coded reactions, reaction type (e.g. allergy, contraindication, intolerance), and reaction severity.[3]

## Recommended Practice - Allergy Documentation

**1.2** Allergies to medications, contrast agents, and latex are entered and updated as structured data before order entry and environmental allergens are included in the problem list.

Checklist

### Implementation Status

EHR Limitation

### Rationale for Practice or Risk Assessment

Complete and accurate documentation of drug and food allergies including reaction type and severity promotes meaningful clinical relevance and may improve the accessibility and usability of the allergy list.

Assessment Notes

Follow-up Actions

Person Responsible for Follow-up Action

Reset Page

### Suggested Sources of Input

1. Clinicians
2. Clinician support staff
3. Clinical administration
4. EHR developer

### Strength of Recommendation

Medium

### Implementation Guidance

- Allergy data is updated regularly to include drugs newly approved by the Food and Drug Administration (FDA).
- Environmental allergens (e.g., pollen, animal dander, mold, insects) are documented in the problem list and are not included in the drug allergy section of the EHR.[4, 5]
- Allergen and reaction picklists are consolidated by mapping synonymous data elements (e.g., “dyspnea” and “shortness of breath”).[6]
- Staff who document allergies are trained to use only structured data elements including “No Known Allergies” checkboxes. This includes physicians, nurses, and medical assistants.[4, 5]
- Staff who document allergies are trained to avoid documenting allergens in the “other” category except for rare circumstances in which no structured allergen is available.
- A regular review of “other” allergens is undertaken to create appropriate structured responses and to reassign such allergens.
- Healthcare team members who routinely enter data in the allergy module are trained in basic allergy terminology, types of adverse reactions, and documentation best practices.[4]

## Recommended Practice - CDS Development

**1.3** CDS content and configuration (including but not limited to alerts, order sets, preventative care, and screening reminders) are based on current best practice guidance that is developed, reviewed, and updated by clinical staff representing a variety of specialties and disciplines. *Checklist*

## Implementation Status

EHR Limitation

## Rationale for Practice or Risk Assessment

As clinical knowledge evolves, leveraging up-to-date CDS endorsed and optimized by practicing clinical staff (e.g., physicians, pharmacists, and nurses) ensures relevant and appropriate integration with workflows across roles and settings while promoting positive patient outcomes.

Assessment Notes

Follow-up Actions

Person Responsible for Follow-up Action

Reset Page

## Suggested Sources of Input

1. Clinicians, support staff, and/or clinical administration
2. EHR developer
3. Health IT support staff

## Strength of Recommendation

Medium

## Implementation Guidance

- The organization has established a CDS governance committee with representatives including, but not limited to, physicians, nurses, pharmacists, informaticians, and IT staff.[7]
- A process is in place to periodically review interruptive CDS (e.g., pop-ups indicating that a medication requires additional approval) to ensure that only the most significant and useful alerts, as determined by the organization, interrupt clinicians.[8]
- Evidence-based order sets are available for common tasks and conditions and are updated regularly based on evolving clinical specialty society and other evidence-based guidance.
- Organization-wide order set utilization is regularly reviewed and findings are used to update content accordingly to address evolving needs:[9]
  - Most commonly used items (to allow default adjustment and removal of low-value items)
  - Orders placed after order sets are used (a possible indicator of missing items)
- Clinical care standardization efforts should consider analysis of variations in practice and feasibility across care settings.[10]
- CDS assets are reviewed and revised as necessary to align with updates to the drug database as well as to other relevant terminologies (e.g., LOINC, SNOMED, ICD-10, CPT).
- If vendor(s) provide CDS assets, healthcare organization clinical staff are provided with an opportunity to review and optimize the content prior to implementation.
- The EHR offers a wide range of trigger options to CDS implementers.

## Recommended Practice - Alerts and Reminders

**2.1** CDS alerts and reminders provide unambiguous guidance in the correct clinical context at relevant points in the workflow. Alerts and reminders are informative, actionable, and judiciously limited to the most significant, patient-specific notifications.

Checklist

## Implementation Status

EHR Limitation

## Rationale for Practice or Risk Assessment

Well-designed and configured alerts within clinical workflows can promote patient safety and positive patient outcomes without overwhelming ordering providers and other clinical staff with irrelevant information. Whether they are warnings about critical drug interactions or notifications based on preventive care guidelines, alerts should be tiered by severity and clearly and concisely describe the next action to take. Careful consideration should be given to defining alert levels, determining the context in which they will fire, and understanding the risks and benefits of potential clinical workflow disruptions (e.g., hard stops that require documentation of override rationale or soft stops that are dismissible without further action).

Assessment Notes

Follow-up Actions

Reset Page

Person Responsible for Follow-up Action

## Suggested Sources of Input

1. Clinicians
2. Clinical support staff
3. Clinical administration
4. Pharmacists
5. Nurses
6. Informatics staff
7. Health IT support staff
8. EHR developer

## Strength of Recommendation

Strong

## Implementation Guidance

- The organization's CDS governance has a process for developing, maintaining, and regularly revising alerts based on clinical user feedback, emerging knowledge, and high override rates.[11]
- The EHR allows users to provide feedback on CDS content directly within the workflow.[12]
- Alerts are designed to appear in the right place in the workflow for the right user (e.g., for the provider during order selection, the pharmacist during order fulfillment, and the nurse during medication administration).[7]
- If CDS uses AI such as a predictive model, the model's calculations are sufficiently explained (e.g., decision trees, templated text, or feature importance) along with its recommendations.[13]
- The organization has established standards limiting the use of interruptive alerts to only the most critical warnings.[14]
- Alerts requiring action include the ability to perform or jump directly to the intended action.[11]
- Interaction checking occurs for all active medications when a new allergy is entered (i.e., reverse checking).
- Dose range and maximum daily dose checking occur before medication orders are submitted for dispensing.

## Recommended Practice - Alerts and Reminders (Cont'd)

**2.1** CDS alerts and reminders provide unambiguous guidance in the correct clinical context at relevant points in the workflow. Alerts and reminders are informative, actionable, and judiciously limited to the most significant, patient-specific notifications.

*Checklist*

### Implementation Guidance

- Medication dosing alerts take into consideration relevant patient-specific data such as patient age, gender, and laboratory result values (e.g., metformin ordered for patients with impaired renal function as evidenced by decreased estimated glomerular filtration rate [eGFR]).[15, 16]
- Order sets are configured to facilitate appropriate corollary or consequent orders and reflect changes made to the original order (e.g., rescheduling, renewing, or discontinuing).[17]
- Incomplete orders requiring further actions (e.g., answers to specific questions) are clearly communicated to the ordering provider during order entry and prior to submission.
- The organization has a robust process for managing feedback, responding to users, and tracking improvements made.[12]

## Recommended Practice - Special Populations

**2.2** EHR ordering and decision support functionality is configured to provide safe, relevant, and effective content for pediatric, geriatric, and other patient populations requiring special considerations for their conditions and diagnoses.

*Checklist*

### Implementation Status

EHR Limitation

### Rationale for Practice or Risk Assessment

In addition to basic drug-drug and drug-allergy interaction checking, accurate integrated ordering and decision support are necessary to reduce the risk of harm to neonates, infants, children, and older adults. More sophisticated CDS rules customized to address transient and permanent patient conditions (e.g., pregnancy, hepatic insufficiency, end-stage renal disease) may be challenging due to EHR functionality limitations and variability in documentation of conditions in a structured format capable of triggering CDS.

Assessment Notes

Follow-up Actions

Person Responsible for Follow-up Action

Reset Page

### Suggested Sources of Input

1. Clinicians, support staff, and/or clinical administration
2. Pharmacists
3. Informaticists
4. EHR developer
5. Health IT support staff

### Strength of Recommendation

Strong

### Implementation Guidance

- **General considerations:**
  - Alerts and notifications are designed to consider patient-specific characteristics as well as specific target medications in order to generate less generic, more relevant decision support.[18]
  - There is a process in place to allow specific departments to suppress burdensome drug - condition or other types of warnings (e.g., anesthesia clinicians should not receive preventive care reminders during surgery; oncology clinicians should not receive warnings that specific medications should only be used in oncology; clinicians in geriatrics should not receive 'use with caution in the elderly' warnings).
  - Users can access authoritative clinical reference materials directly from the EHR (e.g., Up-To-Date, Prescribers' Digital Reference).[19]
- **Older adults:**
  - Drug-age interaction alerts for older adults are based on recommendations from reputable specialty societies (e.g., American Geriatrics Society Beers Criteria® for Potentially Inappropriate Medication Use in Older Adults)[20] or (e.g., STOPP Screening Tool of Older Persons' Prescriptions) and should be used judiciously.[21,22]
- **Infants, children, and adolescents:**
  - A weight-based dosing calculator is integrated within the EHR.[23]
  - Dose rounding is to the appropriate decimal necessary for precision dosing of low-weight patients (e.g., nearest 0.1 mL/hour or third decimal point for kilograms).[23, 24]
  - The system supports display of corrected/adjusted age for preterm infants and neonates in addition to chronological age.[23]
  - Pediatric ordering functionality includes the ability to specify appropriate and available drug concentrations and strengths for neonates, infants, and children.

## Recommended Practice - Special Populations (Cont'd)

**2.2** EHR ordering and decision support functionality is configured to provide safe, relevant, and effective content for pediatric, geriatric, and other patient populations requiring special considerations for their conditions and diagnoses.

*Checklist*

### Implementation Guidance

- Patients with certain conditions:
    - Ordering providers and pharmacists collaborate in developing, implementing, and maintaining clinically relevant drug-disease decision support while reducing insignificant interaction warnings.[25]
    - Drug interaction alerts are configurable for medications ordered or active during pregnancy and/or breastfeeding.

## Recommended Practice - Medications

**2.3** EHR enables the documentation of additional safeguards (e.g., pharmacy review, supervising clinician signoff, independent double check) to reduce the possibility of patient harm from high-risk medications.

*Checklist*

### Implementation Status

**EHR Limitation**

### Rationale for Practice or Risk Assessment

Organization-specific and broadly utilized medication management safety practices may include additional documentation requirements beyond order entry signatures. The EHR should provide mechanisms for ordering providers, pharmacists, and nurses to enter information necessary to meet these additional requirements.

Assessment Notes

Follow-up Actions

Person Responsible for Follow-up Action

Reset Page

### Suggested Sources of Input

1. Ordering providers
2. Pharmacists
3. Nurses
4. Patient safety staff
5. Informatics staff

### Strength of Recommendation

Medium

### Implementation Guidance

- Special attention is given to the development of safeguards for high alert medications in collaboration with and targeted toward ordering providers, pharmacists, nurses, and informatics staff.[26]
- Critical information that may delay treatment is available during order entry (e.g., alternatives for non-formulary medications, notification of drug shortages, prescription cost).[27]
- The EHR supports reconciliation of orders at key care delivery milestones (e.g., hospital admission, before discharge, before major procedures).[28]
- High alert medication doses, routes, and warnings are reviewed at regular intervals and updated as needed to reflect the latest information from authoritative sources such as the Institute for Safe Medication Practices.[29]
- Critical patient information (e.g., age, weight, allergies, pregnancy status) is visible during the order entry process.
- Independent double check documentation is enabled for high alert medications.
- The display of brand and generic drug names is standardized within CPOE and across medication lists to reduce confusion and possible duplication of orders.[30]
- Tall Man Lettering is implemented throughout the EHR to reduce ordering, fulfilling, and administering medications with names that are similar in appearance or sound (e.g., hydrALAZINE/hydrOXYzine, vinBLAStine/vinCRIStine).[31]

## Recommended Practice - Closed Loop Orders

**2.4** Referrals and transition of care orders are standardized, structured, and supported by functionality that tracks the order to completion.
*Checklist*

### Implementation Status

EHR Limitation

### Rationale for Practice or Risk Assessment

Structured referral orders enhance interoperability and standardized templates reduce variability and ensure relevant data is included for order recipients. Bidirectional communication between the ordering provider and order recipient can reduce delays based on incomplete information, follow-up questions, delays, or denials.

Assessment Notes

Follow-up Actions

Person Responsible for Follow-up Action

Reset Page

### Suggested Sources of Input

1. Clinicians
2. Clinical consultants
3. Informaticists
4. Health IT staff
5. Vendor

### Strength of Recommendation

Medium

### Implementation Guidance

- Referral and transition of care orders are available in a structured format.
- Referral order recipients are selected from an up-to-date directory of providers.
- Changes to medications and other time-sensitive orders trigger notifications of occurrence to the person responsible for carrying out the order and provide for the ability to record an acknowledgment of receipt, and action taken by the recipient.[32]
- Bidirectional communication is implemented for orders sent to third-party systems via application programming interfaces (APIs).[32]
- Referral order status can be tracked at the patient level and includes automated notification for referrals that remain open after a specified interval.[33]
- Referral information is transmitted to external organizations via FHIR standards as soon as technically feasible.[34]
- Orders are routed reliably, and if undeliverable or unrenderable, are automatically escalated via notification to the ordering provider and/or staff.
- The provider is automatically notified about orders placed but not completed (e.g., downstream cancellation, patient refused, patient expired).
- The organization monitors order routing (e.g., notifications or error queues).
- The organization has a policy describing the urgency of notification about types of orders placed but not completed (e.g., missed anticoagulation clinic visit may be more urgent than missed lipid screening).

## Recommended Practice - Monitoring CPOE and CDS

**3.1** Key metrics related to CPOE and CDS functionality are defined, monitored, and used to optimize safety and efficiency.

*Checklist*

### Implementation Status

EHR Limitation

### Rationale for Practice or Risk Assessment

Monitoring findings from the analysis of CPOE and CDS metrics can uncover potential problems with utilization and effectiveness.

Assessment Notes

Follow-up Actions

Person Responsible for Follow-up Action

Reset Page

### Suggested Sources of Input

1. Quality and safety staff
2. Organizational leadership

### Strength of Recommendation

Strong

### Implementation Guidance

- Rates of CPOE utilization for orders including medications, diagnostic testing, procedures, and referrals are monitored and compared with benchmarks.
- CDS alert overrides are classified and analyzed to identify potential improvements.
- There is a process for multidisciplinary clinical users (e.g., ordering providers, pharmacists, and nurses) to provide feedback about CPOE and CDS functionality.
- Statistical anomaly detection is enabled for alert firing logs.[35]
- Special attention is paid to monitoring potential malfunctions after migration to a new EHR system, version upgrades and patches, and code and value set changes.[36]
- CPOE and CDS functionality are tested to ensure proper operation before go-live and with test patients in the production system before clinical use.[37]
- A CPOE evaluation tool (e.g., the Leapfrog Group's CPOE "flight simulator" for hospitals) is used annually on the production system to evaluate the safety and effectiveness of CPOE and CDS functionality.[38-40]
- CDS rules should also be tested in the production environment after any CDS-related change and after major EHR software upgrades. This testing should be done for both new rules and existing rules (i.e., regression testing).[37, 38]
- AI technology used for medication ordering is tested on an annual basis.[41]

## References

1. Kandaswamy S, Pruitt Z, Kazi S, et al. Clinician Perceptions on the Use of Free-Text Communication Orders. Appl Clin Inform. 2021;12:484-494 https://pubmed.ncbi.nlm.nih.gov/34077971/. doi:10.1055/s-0041-1731002; PMID: 34077971; PMC8172259.

2. Kazi S, Handley JL, Milicia AP, et al. Free Text as Part of Electronic Health Record Orders: Context or Concern? Patient Safety. 2024;6. https://patientsafetyj.com/article/118587-free-text-as-part-of-electronic-health-record-orders-context-or-concern.

3. Rabbani N, Ho M, Dash D, Calway T, Morse K, Chadwick W. Pseudorandomized Testing of a Discharge Medication Alert to Reduce Free-Text Prescribing. Appl Clin Inform. 2023;14:470-477. https://pubmed.ncbi.nlm.nih.gov/37015344/. doi: 10.1055/a-2068-6940; PMID: 37015344; PMC10266904.

4. Guyer AC, Macy E, White AA, et al. Allergy Electronic Health Record Documentation: A 2022 Work Group Report of the AAAAI Adverse Reactions to Drugs, Biologicals, and Latex Committee. J Allergy Clin Immunol Pract. 2022;10:2854-2867. https://pubmed.ncbi.nlm.nih.gov/36151034/. doi: 10.1016/j.jaip.2022.08.020; PMID: 36151034.

5. Li L, Foer D, Hallisey RK, et al. Improving Allergy Documentation: A Retrospective Electronic Health Record System-Wide Patient Safety Initiative. J Patient Saf. 2022;18:e108-e114. https://pubmed.ncbi.nlm.nih.gov/32487880/. doi: 10.1097/PTS.0000000000000711; PMID: 32487880; PMC7704710.

6. Wang L, Blackley SV, Blumenthal KG, et al. A dynamic reaction picklist for improving allergy reaction documentation in the electronic health record. J Am Med Inform Assoc. 2020;27:917-923. https://pubmed.ncbi.nlm.nih.gov/32417930/. doi:10.1093/jamia/ocaa042; PMID: 32417930; PMC7309236.

7. Schreiber R, McGreevey JD. Chapter 20 - CDS governance and implementation. In: Greenes RA, Del Fiol G, eds. Clinical Decision Support and Beyond (Third Edition). Oxford: Academic Press; 2023:561-601.

8. McCoy AB, Russo EM, Johnson KB, et al. Clinician collaboration to improve clinical decision support: the Clickbusters initiative. J Am Med Inform Assoc. 2022;29:1050-1059. https://pubmed.ncbi.nlm.nih.gov/35244165/. doi: 10.1093/jamia/ocac027; PMID: 35244165; PMC9093034.

9. Li RC, Wang JK, Sharp C, Chen JH. When order sets do not align with clinician workflow: assessing practice patterns in the electronic health record. BMJ Qual Saf. 2019;28:987-996. https://pubmed.ncbi.nlm.nih.gov/31164486/. doi: 10.1136/bmjqs-2018-008968; PMID: 31164486; PMC6868292.

10. Brunner J, Cannedy S, McCoy M, Hamilton AB, Shelton J. Software is Policy: Electronic Health Record Governance and the Implications of Clinical Standardization. J Gen Intern Med. 2023;38:949-955. https://pubmed.ncbi.nlm.nih.gov/37798574/. doi: 10.1007/s11606-023-08280-7; PMID: 37798574; PMC10593671.

11. McGreevey JD, Mallozzi CP, Perkins RM, Shelov E, Schreiber R. Reducing Alert Burden in Electronic Health Records: State of the Art Recommendations from Four Health Systems. Appl Clin Inform. 2020;11:1-12. https://pubmed.ncbi.nlm.nih.gov/31893559/. doi:10.1055/s-0039-3402715; PMID: 31893559; PMC6938713.

12. Rubins D, McCoy AB, Dutta S, et al. Real-Time User Feedback to Support Clinical Decision Support System Improvement. Appl Clin Inform. 2022;13:1024-1032. https://pubmed.ncbi.nlm.nih.gov/36288748/. doi: 10.1055/s-0042-1757923; PMID: 36288748; PMC9605820.

13. Gombolay GY, Silva A, Schrum M, et al. Effects of explainable artificial intelligence in neurology decision support. Ann Clin Transl Neurol. 2024;11:1224-1235. https://www.ncbi.nlm.nih.gov/pmc/articles/PMC11093252/. doi: 10.1002/acn3.52036; PMID: 38581138; PMC11093252.

14. Chaparro JD, Beus JM, Dziorny AC, et al. Clinical Decision Support Stewardship: Best Practices and Techniques to Monitor and Improve Interruptive Alerts. Appl Clin Inform. 2022;13:560-568. https://pubmed.ncbi.nlm.nih.gov/35613913/. doi: 10.1055/s-0042-1748856; PMID: 35613913; PMC9132737

15. Al-Jazairi AS, AlQadheeb EK, AlShammari LK, et al. Clinical Validity Assessment of Integrated Dose Range Checking Tool in a Tertiary Care Hospital Using an Electronic Health Information System. Hosp Pharm. 2021;56:95-101. https://pubmed.ncbi.nlm.nih.gov/33790484/. doi: 10.1177/0018578719867663; PMID: 33790484; PMCID: PMC7958367.

## References

16. Ibáñez-Garcia S, Rodriguez-Gonzalez C, Escudero-Vilaplana V, et al. Development and Evaluation of a Clinical Decision Support System to Improve Medication Safety. Appl Clin Inform. 2019;10:513-520. https://pubmed.ncbi.nlm.nih.gov/31315138/. doi: 10.1055/s-0039-1693426; PMID: 31315138; PMC6637024.

17. Page N, Baysari MT, Westbrook JI. A systematic review of the effectiveness of interruptive medication prescribing alerts in hospital CPOE systems to change prescriber behavior and improve patient safety. Int J Med Inform. 2017;105:22-30. https://pubmed.ncbi.nlm.nih.gov/28750908/. doi: 10.1016/j.ijmedinf.2017.05.011; PMID: 28750908.

18. Scott IA, Pillans PI, Barras M, Morris C. Using EMR-enabled computerized decision support systems to reduce prescribing of potentially inappropriate medications: a narrative review. Ther Adv Drug Saf. 2018;9:559-573. https://pubmed.ncbi.nlm.nih.gov/30181862/. doi: 10.1177/2042098618784809; PMID: 30181862; PMC6116772.

19. SteelFisher GK, Hero JO, Caporello HL, et al. Obstetrician-Gynecologist Views of Pregnancy-Related Medication Safety. J Womens Health (Larchmt). 2020;29:1113-1121. https://pubmed.ncbi.nlm.nih.gov/32233962/. doi: 10.1089/jwh.2019.8007; PMID: 32233962; PMC10614027.

20. American Geriatrics Society 2023 updated AGS Beers Criteria® for potentially inappropriate medication use in older adults. J Am Geriatr Soc. 2023;71:2052-2081. https://pubmed.ncbi.nlm.nih.gov/37139824/. doi: 10.1111/jgs.18372; PMID: 37139824.

21. Sallevelt BT, Huibers CJ, Heij JMO, et al. Frequency and acceptance of clinical decision support system-generated STOPP/START signals for hospitalised older patients with polypharmacy and multimorbidity. Drugs Aging. 2022;39:59-73. https://pubmed.ncbi.nlm.nih.gov/34877629/. doi: 10.1007/s40266-021-00904-z; PMID: 34877629; PMC8752546.

22. Ie K, Hirose M, Sakai T, et al. Protocol of a randomised controlled trial on the efficacy of medication optimisation in elderly inpatients: medication optimisation protocol efficacy for geriatric inpatients (MPEG) trial. BMJ Open. 2020;10:e041125. https://pubmed.ncbi.nlm.nih.gov/33046478/. doi: 10.1136/bmjopen-2020-041125; PMID: 33046478; PMC7552871.

23. Dufendach KR, Eichenberger JA, McPheeters ML, et al. Core Functionality in Pediatric Electronic Health Records. Report No.: 15-EHC014-EF. ed. Rockville (MD): Agency for Healthcare Research and Quality (US); 2015. https://pubmed.ncbi.nlm.nih.gov/25950078/; PMID: 25950078.

24. Whalen K, Lynch E, Moawad I, John T, Lozowski D, Cummings BM. Transition to a new electronic health record and pediatric medication safety: lessons learned in pediatrics within a large academic health system. J Am Med Inform Assoc. 2018;25:848-854. https://pubmed.ncbi.nlm.nih.gov/29688461/. doi: 10.1093/jamia/ocy034; PMID: 29688461; PMC7647031.

25. Bubp JL, Park MA, Kapusnik-Uner J, et al. Successful deployment of drug-disease interaction clinical decision support across multiple Kaiser Permanente regions. J Am Med Inform Assoc. 2019;26:905-910. https://pubmed.ncbi.nlm.nih.gov/30986823/. doi: 10.1093/jamia/ocz020; PMID: 30986823; PMCID: PMC7647201.

26. Booth JP, Hartman AD. Developing a Comprehensive Framework of Safeguarding Strategies to Address Anticipated Errors With Organizational High-Alert Medications. Hosp Pharm. 2024;59:47-55.

27. Pedersen CA, Schneider PJ, Scheckelhoff DJ. ASHP national survey of pharmacy practice in hospital settings: Prescribing and transcribing-2016. Am J Health Syst Pharm. 2017;74:1336-1352. https://pubmed.ncbi.nlm.nih.gov/38223857/.doi: 10.1177/00185787231185871; PMID: 38223857; PMC1078606.

28. Ciapponi A, Fernandez Nievas SE, Seijo M, et al. Reducing medication errors for adults in hospital settings. Cochrane Database Syst Rev. 2021;11:CD009985. https://pubmed.ncbi.nlm.nih.gov/34822165/. doi: 10.1002/14651858.CD009985.pub2; PMID: 34822165; PMC8614640.

29. Institute for Safe Medication Practice, an ECRI affiliate. ISMP updates high-alert medication list. https://www.ismp.org/ sites/default/files/attachments/2024-01/Release%20on%20high%20alert%20list%20update%201-12-24.docx. Accessed Jul 22, 2024.

30. Quist AJL, Hickman TT, Amato MG, et al. Analysis of variations in the display of drug names in computerized prescriber-order-entry systems. Am J Health Syst Pharm. 2017;74:499-509. https://pubmed.ncbi.nlm.nih.gov/28336760/. doi: 10.2146/ajhp151051; PMID: 28336760

## References


31. Iglesias Gomez R, Font Noguera I, Correa Ballester M, Ruiz Caldes MJ, Poveda Andres JL. Tall man lettering application in medication information systems as a quality and safety strategy in hospital organization. J Clin Pharm Ther. 2022;47:1570-1575. https://pubmed.ncbi.nlm.nih.gov/35641241/. doi: 10.1111/jcpt.13703; PMID: 35641241.

32. Partnership for Health IT Patient Safety, ECRI Institute. Health IT safe practices for closing the loop: mitigating delayed, missed, and incorrect diagnoses related to diagnostic testing and medication changes using health IT. https://www.ecri.org/Resources/ HIT/Closing_Loop/Closing_the_Loop_Toolkit.pdf. Accessed Jul 22, 2024.

33. Institute for Healthcare Improvement. Closing the Loop: A Guide to Safer Ambulatory Referrals in the EHR Era. https:// www.ihi.org/resources/publications/closing-loop-guide-safer-ambulatory-referrals-ehr-era. Accessed Jul 22, 2024.

34. Health Data, Technology, and Interoperability: Patient Engagement, Information Sharing, and Public Health Interoperability (HTI-2) Proposed Rule | HealthIT.gov. 2024. https://www.healthit.gov/topic/laws-regulation-and-policy/health-data-technology- and-interoperability-patient-engagement. Accessed Jul 30, 2024.

35. Wright A, Hickman TT, McEvoy D, et al. Methods for Detecting Malfunctions in Clinical Decision Support Systems. Stud Health Technol Inform. 2017;245:1385. https://pubmed.ncbi.nlm.nih.gov/29295464/; PMID: 29295464.

36. Wright A, Ai A, Ash J, et al. Clinical decision support alert malfunctions: analysis and empirically derived taxonomy. J Am Med Inform Assoc. 2018;25:496-506. https://pubmed.ncbi.nlm.nih.gov/29045651/. doi: 10.1093/jamia/ocx106; PMID: 29045651; PMC6019061.

37. Wright A, Aaron S, Sittig DF. Testing electronic health records in the “production” environment: an essential step in the journey to a safe and effective health care system. J Am Med Inform Assoc. 2017;24:188-192. https://www.ncbi.nlm.nih.gov/pmc/articles/PMC6822893/. doi: 10.7759/cureus.5649; PMID: 31700751; PMC6822893.

38. Classen DC, Holmgren AJ, Co Z, et al. National Trends in the Safety Performance of Electronic Health Record Systems From 2009 to 2018. JAMA Netw Open. 2020;3:e205547. https://pubmed.ncbi.nlm.nih.gov/32469412/. doi: 10.1001/jamanetworkopen.2020.5547;PMID: 32469412; PMC7260621.

39. Holmgren AJ, Co Z, Newmark L, Danforth M, Classen D, Bates D. Assessing the safety of electronic health records: a national longitudinal study of medication-related decision support. BMJ Qual Saf. 2020;29:52-59.

40. Classen DC, Longhurst CA, Davis T, Milstein JA, Bates DW. Inpatient EHR User Experience and Hospital EHR Safety Performance. JAMA Netw Open. 2023;6:e2333152. https://pubmed.ncbi.nlm.nih.gov/31320497/. doi: 10.1136/bmjqs-2019-009609; PMID: 31320497.

41. Chalasani SH, Syed J, Ramesh M, Patil V, Pramod Kumar TM. Artificial intelligence in the field of pharmacy practice: A literature review. Explor Res Clin Soc Pharm. 2023;12:100346. https://pubmed.ncbi.nlm.nih.gov/37885437/. doi: 10.1016/j.rcsop.2023.100346; PMID: 37885437; PMCID: PMC10598710.
