# Supplementary material for: Revisions to the Safety Assurance Factors for Electronic Health Record Resilience (SAFER) Guides to update national recommendations for safe use of electronic health records
Source: J Am Med Inform Assoc. 2025 Apr 12;32(4):755–60. doi: 10.1093/jamia/ocaf018 (PMC12005625; doi:10.1093/jamia/ocaf018)
Supplement: ocaf018_Supplementary_Data [file ocaf018_supplementary_data.zip › ocaf018_Supplementary_Data/ocaf018_Supplementary_Data.pdf]

Self-Assessment

High Priority Practices

General Instructions for the SAFER Self-Assessment Guides

The Safety Assurance Factors for EHR Resilience (SAFER) guides are designed to help healthcare organizations conduct proactive self-assessments to evaluate the safety and effectiveness of their electronic health record (EHR) implementations. The 2025 SAFER guides have been updated and streamlined to focus on the highest risk, most commonly occurring issues that can be addressed through technology or practice changes to build system resilience in the following areas:

- Organizational Responsibilities
- Patient Identification
- Clinician Communication
- Test Results Reporting and Follow-up
- Computerized Provider Order Entry with Decision Support
- Systems Management
- Contingency Planning
- High Priority Practices - A collection of 16 Recommendations from the other 7 Guides

Each of the eight SAFER Guides begins with a Checklist of recommended practices. The downloadable SAFER Guides provide fillable circles that can be used to indicate the extent to which each recommended practice has been implemented in the organization using a 5-point Likert scale. The Practice Worksheet gives a rationale for the practice and provides examples of how to implement each recommended practice. It contains fields to record team member involvement and follow-up actions based on the assessment. The Worksheet also lists the stakeholders who can provide input to assess each practice (sources of input). In addition to the downloadable version, the content of each SAFER Guide, with interactive references and supporting materials, can also be viewed on ONC's website at: https://www.healthit.gov/topic/safety/safer-guides.

The SAFER guides are based on the best available (2024) evidence from the literature and consensus expert opinion. Subject matter experts in patient safety, informatics, quality improvement, risk management, human factors engineering, and usability developed them. Furthermore, they were reviewed by an external group of practicing clinicians, informaticians, and information technology professionals.

Each guide contains between 6 and 18 recommended practices including its rationale, implementation guidance, and evidence level. The recommended practices in the SAFER Guides are intended to be useful for all EHR users. However, every organization faces unique circumstances and may implement a particular recommended practice differently. As a result, some of the specific implementation guidance in the SAFER Guides for recommended practices may not be applicable to an organization.

The High Priority Practices guide consists of 16 of the most important and relevant recommendations selected from the other 7 guides. It is designed for practicing clinicians to help them understand, implement, and support EHR safety and safe use within their organization. The other seven guides consist of 88 unique recommendations that are relevant for all healthcare providers and organizations.

The SAFER Guides are designed in part to help deal with safety concerns created by the continuously changing sociotechnical landscape that healthcare organizations face. Therefore, changes in technology, clinical practice standards, regulations, and policy should be taken into account when using the SAFER Guides. Periodic self-assessments using the SAFER Guides may also help organizations identify areas where it is particularly important to address the implications of these practice or EHR-based changes for the safety and safe use of EHRs. Ultimately, the goal is to improve the overall safety of our health care system and improve patient outcomes.

The SAFER Guides are not intended to be used for legal compliance purposes, and implementation of a recommended practice does not guarantee compliance with the HIPAA Security or Privacy Rules, Medicare or Medicaid Conditions of Participation, or any other laws or regulations. The SAFER Guides are for informational purposes only and are not intended to be an exhaustive or definitive source. They do not constitute legal advice. Users of the SAFER Guides are encouraged to consult with their own legal counsel regarding compliance with Medicare or Medicaid program requirements, and any other laws.

For additional information on Medicare and Medicaid program requirements, please visit the Centers for Medicare & Medicaid Services website at www.cms.gov. For more information on HIPAA, please visit the HHS Office for Civil Rights website at www.hhs.gov/ocr.

Self-Assessment

High Priority Practices

Introduction

As the modern healthcare delivery system continues to evolve, the safe and effective design, development, implementation, and use of electronic health records (EHRs) as the primary means of patient data collection, storage, retrieval, and communication becomes more apparent. The SAFER guides were designed to help clinicians, healthcare organizations, and EHR vendors carry out self-assessments of their EHRs as implemented within a healthcare delivery organization. Many of the SAFER recommendations require that EHR vendors design and develop the software required to enable users to complete the recommendation, but all recommendations require effort on the part of clinicians and healthcare delivery organizations to configure, implement, and use the EHR to its fullest extent to meet the recommendations.

The High Priority Practices SAFER Guide includes 16 recommendations selected from the other seven guides because of their relevance and importance for practicing clinicians to understand and support. While front-line clinicians need to be aware of the full complement of 88 recommendations across all of the guides, these are the most critical for clinicians to help mitigate safety risks while advocating for organization-wide prioritization of recommendations that have not yet been implemented.

SAFER recommendations should help healthcare organizations identify, prevent, measure, and monitor EHR-related patient safety risks. These risks result from both “social” (involving people, leadership, workflow, and policies) and “technical” (involving EHR hardware and software and system-to-system interfaces, EHR configurations, upgrades, and maintenance) challenges. This guide will help people responsible for EHR safety in each specific complex “sociotechnical” healthcare organization focus on the most important safety challenges and risks introduced by EHRs.

The 2024 revision of the High-Priority SAFER guide includes many new recommendations. One focuses on the safe and effective use of artificial intelligence (AI)-enabled applications and another on the use of patient portals and patient-clinician communication. These relatively new features offer great promise for improvements in the delivery of safe and effective healthcare, but both have risks to patient safety that must be managed.

While each of the seven individual SAFER guides is designed to be used by a multi-disciplinary group, this High Priority Guide is for front-line clinicians. No one expects every clinician to understand the depth and breadth of every recommendation or the accompanying implementation guidance suggestions. The SAFER guides should prompt clinicians to ask questions and hopefully start a conversation among other clinicians, administrators, and information technology professionals as they work collaboratively to design, develop, and implement safe and effective electronic health record systems.

We hope that this collaboration will lead to a consensus about the organization’s future path to optimize EHR-related safety and quality: setting priorities among the recommended practices not yet addressed, ensuring a plan is in place to maintain recommended practices already in place, dedicating the required resources to make necessary improvements, and working together to mitigate the highest priority safety risks introduced by the EHR.

Self-Assessment

High Priority Practices

Table of Contents

General Instructions 1

Introduction 2

About the Checklist 5

Checklist 6

Team Worksheet 7

About the Recommended Practice Worksheets 9

Recommended Practice Worksheets 10

References 28

Authors and Peer Reviewers

The SAFER Self-Assessment Guides were developed by health IT safety researchers and informatics experts whose contributions are acknowledged as follows:

Primary authors who contributed to the development of all guides:

Trisha Flanagan, RN, MSN, CPPS, Health Informatics Nurse, Center for Innovations in Quality, Effectiveness and Safety, Michael E. DeBakey Veterans Affairs Medical Center, Houston TX

Hardeep Singh, MD, MPH, Co-Chief, Health Policy, Quality and Informatics Program, Center for Innovations in Quality, Effectiveness and Safety and Professor of Medicine at the Michael E. DeBakey Veterans Affairs Medical Center and Baylor College of Medicine, Houston, TX

Dean F. Sittig MS, PhD, FACMI, FAMIA, FHIMSS, FIAHSI, Professor of Biomedical Informatics, Department of Clinical and Health Sciences, McWilliams School of Biomedical Informatics, University of Texas Health Science Center at Houston, TX and Informatics Review LLC, Lake Oswego, OR

Support staff for the primary authorship team

Rosann Cholankeril, MD, MPH, Center for Innovations in Quality, Effectiveness and Safety, Michael E. DeBakey Veterans Affairs Medical Center and Baylor College of Medicine

Sara Ehsan, MBBS, MPH, Center for Innovations in Quality, Effectiveness and Safety, Michael E. DeBakey Veterans Affairs Medical Center and Baylor College of Medicine

Additional authors who contributed to at least one guide:

Jason S. Adelman, MD, MS, (Patient ID) Chief Patient Safety Officer & Associate Chief Quality Officer, Executive Director, Patient Safety Research, Co-Director, Patient Safety Research Fellowship in Hospital Medicine, New York-Presbyterian Hospital/Columbia University Irving Medical Center, New York, NY

Daniel R. Murphy, MD, MBA, (Clinician Communication, Test Results) Chief Quality Officer, Baylor Medicine, Houston, TX

Patricia Sengstack, DNP, NI-BC, FAAN, FACMI, (Organizational Responsibilities) Senior Associate Dean for Informatics, Director, Nursing Informatics Specialty Program, Vanderbilt University School of Nursing, Vanderbilt University, Nashville, TN

Additional contributors who provided feedback on various guides or parts of guides

Miriam Callahan, MD (Patient ID)
David C. Classen, MD (CPOE, AI recommendation)
Anne Grauer, MD, MS (Patient ID)
Ing Haviland (Patient ID)
Amanda Heidemann, MD (All Guides)
I-Fong Sun Lehman, DrPH, MS (Patient ID)
Christoph U. Lehmann, MD (AI recommendation)
Christopher A. Longhurst, MD, MS (AI recommendation)
Edward R. Melnick, MD (Clinician Communication)
Robert E. Murphy, MD (Organizational Responsibilities)
Ryan P. Radecki, MD, MS (AI recommendation)
Raj Ratwani, PhD (AI recommendation)
Trent Rosenbloom, MD (Clinician Communication)
Lisa Rotenstein, MD (Clinician Communication)
Hojjat Salmasian, MD, PhD (All Guides)
Richard Schreiber, MD (CPOE)
Danny Sands, MD (Clinician Communication)
Debora Simmons, PhD, RN (Organizational Responsibilities)
Carina Sirochinsky (Patient ID)
Neha Thummala, MPH (Patient ID)
Emma Weatherford (Patient ID)
Adam Wright, PhD (CPOE)
Andrew Zimolzak, MD, MMSc (Test Results, Clinician Communication)

This guide was developed under the contract Unintended Consequences of Health IT and Health Information Exchange, Task Order HHSP23337003T/HHSP23320095655WC.

The ATSP/ONC composite mark is a mark of the U.S. Department of Health and Human Services. The contents of the publication or project are solely the responsibility of the authors and do not necessarily represent the official views of the U.S. Department of Health and Human Services, Asssistant Secretary for the Technology Policy/Office of the National Coordinator for Health Information Technology.

The *Checklist* is structured as a quick way to enter and print your self-assessment.

Select the level of implementation achieved by your organization for each Recommended Practice. Your Implementation Status will be reflected on the Recommended Practice Worksheet in this PDF. The implementation status scales are as followed:

Not Implemented – (0%) The organization has not implemented this recommendation.

Making Progress (1 - 30%) The organization is in the early or pilot phase of implementing this recommendation as evidenced by following or adopting less than 30% of the implementation guidance.

Halfway there (31 – 60%) The organization is implementing this recommendation and is following or has adopted approximately half of the implementation guidance.

Substantial Progress (61-90%) The organization has nearly implemented this recommendation and is following or has adopted much of the implementation guidance.

Fully Implemented (91-100%) The organization follows this recommendation, and most implementation guidance is followed consistently and widely adopted.

The organization should check the following box if there are some limitations with the current version of their EHR that preclude them from fully implementing this recommendation.

EHR Limitation - The EHR does not offer the features/functionality required to fully implement this recommendation or the implementation guidance.

The *Domain* associated with the *Recommended Practice(s)* appears at the top of the column

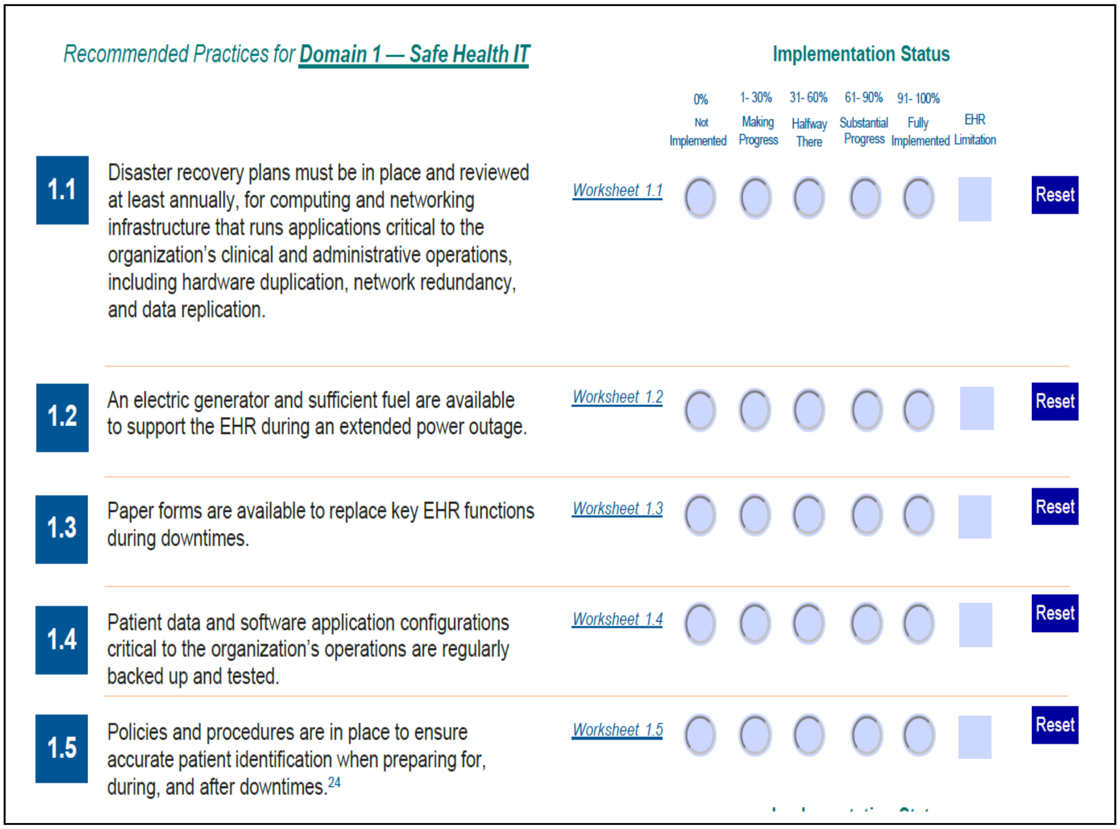

The *Recommended Practice(s)* for the topic appears below the associated *Domain*.

To the right of reach *Recommended Practice* is a link to the Recommended Practice Worksheet in this PDF.

The *Worksheet* provides guidance on implementing the practice.

Recommended Practices for **Domain 1 — Safe Health IT**

Implementation Status

#	Practice	Worksheet	0% Not Implemented	1- 30% Making Progress	31- 60% Halfway There	61- 90% Substantial Progress	91- 100% Fully Implemented	EHR Limitation	
1.1	Highest-level decision makers in the organization (e.g., boards of directors, owners of physician practices, C-suite executives, and clinical leaders) commit to promoting a culture of safety that incorporates the safety and safe use of EHRs.	Worksheet 1.1							Reset
1.2	Users are warned when they attempt to create a record for a new patient whose first and last names are the same as another patient, or when a patient search result returns multiple patients with the same or similar names.[5]	Worksheet 1.2							Reset
1.3	Patient data and software application configuration settings critical to the organization's operations are regularly backed up and tested.[10]	Worksheet 1.3							Reset
1.4	EHR-based secure messaging systems ensure accurate, reliable, and efficient transmission of high-risk information.	Worksheet 1.4							Reset
1.5	Artificial Intelligence (AI)-enabled application developers, EHR vendors, and healthcare organizations using AI-enabled systems or EHRs with enhanced AI features or functions share responsibility (based on their ability and resources available) for ensuring AI safety. This shared responsibility includes appropriate clinical, technical, and administrative governance, policies, procedures, people, and technologies to ensure AI is monitored and that its use is safe, secure, private, ethical, and equitable.[20]	Worksheet 1.5							Reset

Recommended Practices for **Domain 2 — Using Health IT Safely**

Implementation Status

#	Practice	Worksheet	0% Not Implemented	1- 30% Making Progress	31- 60% Halfway There	61- 90% Substantial Progress	91- 100% Fully Implemented	EHR Limitation	
2.1	Healthcare organizations and EHR vendors share responsibility for identifying and addressing EHR safety concerns.	Worksheet 2.1							Reset
2.2	The EHR inbox and its use is optimized to reduce inbox burden.	Worksheet 2.2							Reset
2.3	Patient photographs are collected during patient registration and displayed in multiple places in the EHR to improve patient identification.[37]	Worksheet 2.3							Reset
2.4	Written policies specify unambiguous responsibility for test result follow-up with a shared understanding of that responsibility among all involved in providing follow-up care.[45-54]	Worksheet 2.4							Reset

Recommended Practices for Domain 2 — Using Health IT Safely

Implementation Status

	Practice	Worksheet	0% Not Implemented	1- 30% Making Progress	31- 60% Halfway There	61- 90% Substantial Progress	91- 100% Fully Implemented	EHR Limitation	
**2.5**	System hardware, operating and network software, and clinical application version updates, modifications, and local customizations are tested individually and in the context of other integrated systems using a standardized approach. This consists of:<br>• Testing before go-live and as installed in production to ensure adequate performance and data integrity<br>• Testing based on real-world, clinically authentic, and relevant scenarios incorporating collaborative workflows[59]<br>• Monitoring all systems for a short time following any hardware or software changes<br>• Notifying end users before, and reminded them after, potentially impactful changes to applications or clinical content assets	Worksheet 2.5	○	○	○	○	○		Reset
**2.6**	CDS alerts and reminders provide unambiguous guidance in the correct clinical context at relevant points in the workflow. Alerts and reminders are informative, actionable, and judiciously limited to the most significant, patient-specific notifications.	Worksheet 2.6	○	○	○	○	○		Reset
**2.7**	Users are trained on ransomware prevention strategies, including how to identify malicious emails and fraudulent telephone callers asking for login access or other privileged information.[70,71]	Worksheet 2.7	○	○	○	○	○		Reset
**2.8**	Staff are trained and tested on downtime and recovery procedures.[79]	Worksheet 2.8	○	○	○	○	○		Reset

Recommended Practices for Domain 3 — Monitoring Safety

Implementation Status

	Practice	Worksheet	0% Not Implemented	1- 30% Making Progress	31- 60% Halfway There	61- 90% Substantial Progress	91- 100% Fully Implemented	EHR Limitation	
**3.1**	Organizations have a strategy and mechanisms for prevention, identification, measurement, monitoring, and mitigation of high priority EHR safety risks and hazards.	Worksheet 3.1	○	○	○	○	○		Reset
**3.2**	Organizational policies and procedures ensure timely patient notification of both normal and abnormal test results, and the timeliness of notification is monitored.[57]	Worksheet 3.2	○	○	○	○	○		Reset
**3.3**	The EHR enables the monitoring of important communication patterns related to clinical messages, referrals, and patient portal notifications.	Worksheet 3.3	○	○	○	○	○		Reset

Clinicians should complete this self-assessment and evaluate potential health IT-related patient safety risks addressed by this specific SAFER Guide within the context of your particular healthcare organization.

This Team Worksheet is intended to help organizations document the names and roles of the self-assessment team, as well as individual team members' activities. Typically, team members will be drawn from a number of different areas within your organization, and in some instances, from external sources. The Suggested Sources of Input section in each Recommended Practice Worksheet identifies the types of expertise or services to consider engaging. It may be particularly useful to engage specific clinician and other leaders with accountability for safety practices identified in this guide.

The Worksheet includes fillable boxes that allow you to document relevant information. The Assessment Team Leader box allows documentation of the person or persons responsible for ensuring that the self-assessment is completed. The section labeled Assessment Team Members enables you to record the names of individuals, departments, or other organizations that contributed to the self-assessment. The date that the self-assessment is completed can be recorded in the Assessment Completion Date section and can also serve as a reminder for periodic reassessments. The section labeled Assessment Team Notes is intended to be used, as needed, to record important considerations or conclusions arrived at through the assessment process. This section can also be used to track important factors such as pending software updates, vacant key leadership positions, resource needs, and challenges and barriers to completing the self-assessment or implementing the Recommended Practices in this SAFER Guide.

Assessment Team Leader

Assessment Completion Date

Assessment Team Members

Assessment Team Notes

Reset

Each *Recommended Practice Worksheet* provides guidance on implementing a specific *Recommended Practice*, and allows you to enter and print information about your self-assessment.

The *Suggested Sources of Input* section indicates categories of personnel who can provide information to help evaluate your level of implementation.

The *Rationale* section provides guidance about "why" the safety activities are needed.

Strength of Recommendation section provides an estimate of the strength of evidence available in the scientific literature, or states that it is "required" due to a federal rule, regulation, or conditions of participation, for each recommendation.

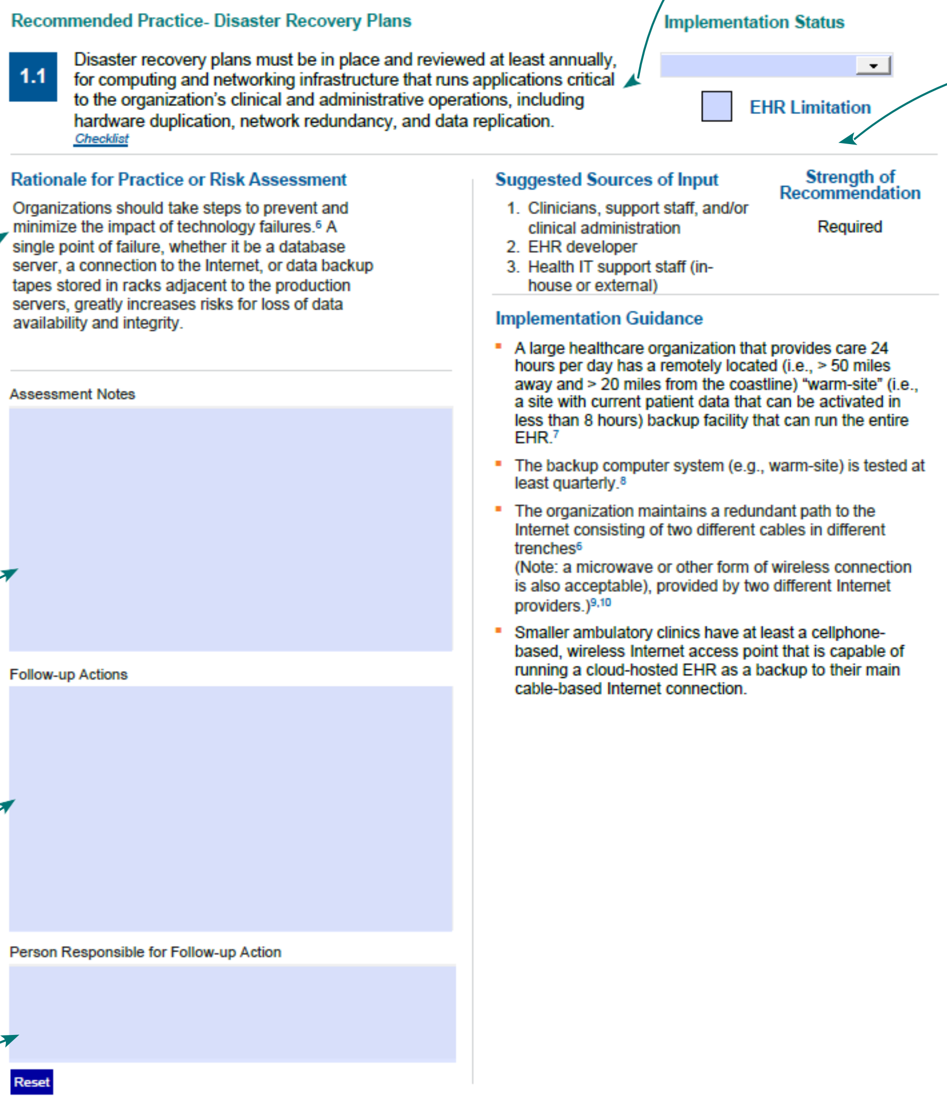

Recommended Practice- Disaster Recovery Plans

1.1 Disaster recovery plans must be in place and reviewed at least annually, for computing and networking infrastructure that runs applications critical to the organization's clinical and administrative operations, including hardware duplication, network redundancy, and data replication.
Checklist

Implementation Status

EHR Limitation

Rationale for Practice or Risk Assessment

Organizations should take steps to prevent and minimize the impact of technology failures.[6] A single point of failure, whether it be a database server, a connection to the Internet, or data backup tapes stored in racks adjacent to the production servers, greatly increases risks for loss of data availability and integrity.

Assessment Notes

Follow-up Actions

Person Responsible for Follow-up Action

Reset

Suggested Sources of Input

1. Clinicians, support staff, and/or clinical administration
2. EHR developer
3. Health IT support staff (in-house or external)

Strength of Recommendation

Required

Implementation Guidance

- A large healthcare organization that provides care 24 hours per day has a remotely located (i.e., > 50 miles away and > 20 miles from the coastline) "warm-site" (i.e., a site with current patient data that can be activated in less than 8 hours) backup facility that can run the entire EHR.[7]
- The backup computer system (e.g., warm-site) is tested at least quarterly.[8]
- The organization maintains a redundant path to the Internet consisting of two different cables in different trenches[6]
(Note: a microwave or other form of wireless connection is also acceptable), provided by two different Internet providers.)[9,10]
- Smaller ambulatory clinics have at least a cellphone-based, wireless Internet access point that is capable of running a cloud-hosted EHR as a backup to their main cable-based Internet connection.

Enter any notes about your self-assessment.

Enter any follow-up activities required.

Enter the name of the person responsible for the follow-up activities.

The Implementation Guidance section lists potentially useful practices or scenarios to inform your assessment and implementation of the specific Recommended Practice.

Recommended Practice - Safety Culture

1.1 Highest-level decision makers in the organization (e.g., boards of directors, owners of physician practices, C-suite executives, and clinical leaders) commit to promoting a culture of safety that incorporates the safety and safe use of EHRs.

Checklist

Implementation Status

EHR Limitation

Rationale for Practice or Risk Assessment

A culture of safety promoted by top executives encourages continuous learning, improvement, and engagement from all levels of the organization. By actively and transparently prioritizing safety, organizational leadership can help ensure systems and processes remain effective and responsive to emerging EHR-related threats and challenges. By prioritizing EHR safety, leadership promotes collaboration across all levels, engaging clinical staff, IT professionals, and administrative personnel in a unified approach to addressing safety concerns and implementing effective solutions. Ultimately, this high-level focus on EHR safety ensures strategic investments in reliable and efficient health IT systems, further solidifying the organization's commitment to safety and excellence.

Suggested Sources of Input

1. Large organization: Board of directors, President/Vice President, C-Suite executives, Clinical leaders
2. Small organization: Owners, Clinical leaders, COO

Strength of Recommendation

Medium

Implementation Guidance

- High-level decision makers recognize that EHR safety is integral to patient safety. They ensure that EHR safety is integrated into organizational policies and procedures and risk management practices.[1,2]
- High-level decision makers provide adequate staffing and resources to ensure that safety issues associated with adoption and use of EHRs can be addressed in a timely fashion.[3]
- High-level decision makers review the results of EHR safety assessments, such as those from SAFER Guide use.
- High-level decision makers identify EHR-related patient safety goals (e.g., percentage of abnormal laboratory test results that are acknowledged within a timeframe appropriate for the importance, severity, and healthcare setting or percentage of medications administered following barcode identification), assess whether those goals are being reached, and address any shortcomings.[3]
- High-level decision makers identify and support staff members who can provide systematic feedback to the EHR vendors regarding perceived safety issues with their EHRs.[4]

Assessment Notes

Follow-up Actions

Person Responsible for Follow-up Action

Reset

Recommended Practice - Multiple Patient Warning

1.2 Users are warned when they attempt to create a record for a new patient whose first and last names are the same as another patient, or when a patient search result returns multiple patients with the same or similar names.[5]

Checklist

Implementation Status

EHR Limitation

Rationale for Practice or Risk Assessment

Using automated EHR processes to prevent duplicate records can prevent unintentional human errors that could lead to patient harm.[6] Patients with similar names are at a higher risk for wrong-patient errors.[7]

Assessment Notes

Follow-up Actions

Person Responsible for Follow-up Action

Suggested Sources of Input

1. EHR developer
2. Health IT support staff

Strength of Recommendation

Medium

Implementation Guidance

- During the creation of a new patient record, a phonetic algorithm such as Soundex[8] is used to check for patients with similar sounding names in the system and display an alert or warning if one exists.
- When looking up a patient, if the results list returns multiple patients with similar demographic data, the names are displayed in a visually distinct manner.
- The system monitors for similar names, name variants (e.g., Robert, Rob, Bob, Robbie), or changed last names (e.g., marriage, divorce, adoption), when other demographics match.
- An alert provides additional demographic information context for the existing patient to help the user confirm or rule out that it is the same patient.
- Organizations implement an ID reentry intervention and/or a distinct naming intervention to reduce wrong-patient errors in the nursery or NICU, where sets of twins, triplets, and higher-order multiples are prevalent.[7]
- Name alerts in combination with other interventions (e.g., blood type testing) prevent patient record confusion in critical areas such as blood transfusions.[9]

Reset

> Table of Contents | > About the Checklist | > Team Worksheet | > About the Practice Worksheets | >Practice Worksheets

Recommended Practice - Backup Data

1.3 Patient data and software application configuration settings critical to the organization's operations are regularly backed up and tested.[10]

Checklist

Implementation Status

EHR Limitation

Rationale for Practice or Risk Assessment

Failure of electro-mechanical devices is inevitable. Backup of mission-critical patient data and EHR system configuration allows system restoration to a "pre-failure" state with minimal data and time loss.

Suggested Sources of Input

1. Clinicians, support staff, and/or clinical administration
2. EHR developer
3. Health IT support staff

Strength of Recommendation

Required

Implementation Guidance

- The organization has a daily, off-site, complete, encrypted backup of patient data.[11]
- Critically important patient data should be backed up as close as possible to real-time.
- If using a remotely hosted EHR (e.g., cloud-based solution), the EHR provider backs up data with tape, Internet, redundant drives, or any means necessary to allow full recovery from incidents.[12]
- The off-site backup is tested regularly (i.e., complete system and patient data restore) (optimally on at least a monthly basis).[13]
- The content required to configure the system is backed up regularly (optimally every month and always before every EHR or supporting computer system upgrade).
- The organization maintains multiple backups, which are created at different times.
- Backup media are physically secured in a location separate from the operational data stores.
- The backup storage media should be separate and distinct (e.g., Air gap) from normal file storage to facilitate recovery from ransomware attacks.[14]
- Backup media are rendered unreadable (i.e., use software to scramble media contents or physically destroy/shred media) before disposal.
- The organization has a "read-only" backup EHR system that is updated frequently (optimally in real-time, but at least hourly).
- The read-only EHR system is tested regularly (optimally at least weekly).
- Users can print from the read-only EHR system.
- If there is a "unit-level" read-only backup EHR system, it isconnected to a local UPS or "red plug" (i.e., an outlet connected to the organization's backup electrical generator).

Assessment Notes

Follow-up Actions

Person Responsible for Follow-up Action

Reset

Recommended Practice - Secure Messaging

1.4 EHR-based secure messaging systems ensure accurate, reliable, and efficient transmission of high-risk information.
Checklist

Implementation Status

EHR Limitation

Rationale for Practice or Risk Assessment

To avoid unnecessary interruptions and distractions, critical and time-sensitive messages and results to clinicians should be clearly differentiated from routine or information-only communication that does not require immediate attention or action.

Suggested Sources of Input

1. Clinicians
2. Ancillary staff
3. Laboratory and diagnostic imaging staff
4. IT staff
5. Vendors

Strength of Recommendation

Medium

Assessment Notes

Follow-up Actions

Person Responsible for Follow-up Action

Reset

Implementation Guidance

- The EHR ensures close loop communication, which implies that "all patient data and information that may require an action are delivered and communicated to the right individuals, at the right time, through the right mode to allow interpretation, critical review, reconciliation, initiation of action, acknowledgment, and appropriate documentation."[15]
- Critical and time-sensitive messages to clinicians are clearly differentiated from routine or information-only communication that does not require immediate attention or action.[16]
- EHR allows urgency levels to be assigned to messages and presents urgent messages in a visually distinct manner. The organization provides guidance to promote succinct and intuitive message content.[17,18]
- Messages can be marked for follow-up on a future date and are automatically re-sent on the specified date and appear as a new message.[19]
- Organization policy for communication requires EHR documentation of patient-specific communication that occurs outside the EHR (e.g., e-mail or text messages sent via computer, smartphone, pager, wireless local area network-based communication devices, or other communication system not integrated with the EHR) within the patient's EHR. Information that should be recorded in the patient's EHR includes sender, recipient, content, time sent, and time acknowledged (if applicable).
- EHR messaging modules automatically capture and store message sender, recipient, content, time, and acknowledgment data.
- The EHR and the organization enable escalation of messages that are unread within a time period (or if no response has been received by the sender depending on urgency). Escalation could involve automatically forwarding the message to an alternate or supervising clinician if the intended recipient is unavailable.[16]

Recommended Practice - Artificial Intelligence

1.5 Artificial Intelligence (AI)-enabled application developers, EHR vendors, and healthcare organizations using AI-enabled systems or EHRs with enhanced AI features or functions share responsibility (based on their ability and resources available) for ensuring AI safety. This shared responsibility includes appropriate clinical, technical, and administrative governance, policies, procedures, people, and technologies to ensure AI is monitored and that its use is safe, secure, private, ethical, and equitable.[20]

Checklist

Implementation Status

EHR Limitation

Rationale for Practice or Risk Assessment

The integration of AI-enabled systems in healthcare has the potential to revolutionize clinical decision-making, but it also introduces known and unknown risks that must be mitigated.[21] As healthcare organizations adapt their clinical and administrative workflows to new AI-driven technologies, unintended adverse consequences will inevitably occur, particularly during the transition period. Early AI applications have already exhibited unintended biases and "hallucinations," leading to false information that can harm patients. To address these risks, healthcare organizations and AI/EHR developers must collaborate, leveraging their complementary expertise to ensure AI systems are robust, reliable, and transparent. Continuous monitoring and updating are crucial to maintain system integrity, prioritize patient safety, and ensure data security. Conducting a risk assessment of AI is essential to identify and mitigate these risks, build trust among users and stakeholders, and promote safe and effective adoption of AI in healthcare.

Assessment Notes

Follow-up Actions

Person Responsible for Follow-up Action

Reset

Suggested Sources of Input

1. Large organizations: Clinicians, Clinical Administration, Health IT Support Staff, EHR (or AI) developer, AI experts
2. Small organizations: Wait for better evidence

Strength of Recommendation

Medium

Implementation Guidance

- Organizations conduct ongoing real-world testing and monitoring with local data to minimize the risk to patient safety while these new AI-enabled systems mature.
- Healthcare organizations should conduct, or wait for real-world, clinical evaluations published in high-quality medical journals (e.g., NLM's new list of Clinically Useful Journals - https://jmla.mlanet.org/ojs/jmla/article/view/1631) before they start using AI-enabled systems on a routine basis. While peer-reviewed publication does not ensure safety or effectiveness of any clinical or administrative intervention, it can provide an external, unbiased assessment of the development, testing, implementation, or use of an AI-enabled system, tool, or intervention.
- Healthcare organizations should add additional people with AI expertise such as data scientists, informaticians, machine-learning and AI operational personnel, human factors experts, and clinical expert(s) to their existing multidisciplinary EHR or CDS oversight committee(s). These individuals, as a group, should be capable of understanding and evaluating the performance of AI-enabled systems. These new committee members should meet regularly to review requests for new applications and proactively monitor the performance of AI- enabled applications in use.
- The committee should maintain an inventory of clinically deployed, AI-enabled systems that includes information on deployment date, current version, responsible personnel, last reviewed date, authorized users, authorized purpose, source of data used to generate, or train, the AI system, and external source(s) of validation, verification, and performance comparison.

Recommended Practice - Artificial Intelligence (cont'd)

1.5 Artificial Intelligence* (AI)-enabled application developers, EHR vendors, and healthcare organizations using AI-enabled systems or EHRs with enhanced AI features or functions share responsibility (based on their ability and resources available) for ensuring AI safety. This shared responsibility includes appropriate clinical, technical, and administrative governance, policies, procedures, people, and technologies to ensure AI is monitored and that its use is safe, secure, private, ethical, and equitable.[20]

Checklist

Implementation Guidance (cont'd)

- Before organizations use AI-enabled systems for patient care (e.g., respond to patient messages, generate differential diagnoses, treatment plans, or notes describing the findings from visits), they must have policies and procedures to ensure that patients and clinicians are aware, when possible, that AI-enabled systems are being used for clinical and/or administrative decision making.[22]
- Organizations should ensure that patients understand when and where AI-enabled systems were developed, how they may be used, and the role of clinicians in reviewing the AI system's output before giving their consent.[23]
- AI-generated recommendations should be reviewed and approved by humans who take responsibility for the recommendation(s) before they are sent to patients.
- Organizations should maintain and regularly review a transaction log of AI system use (i.e., similar to the audit log of the EHR) that includes the AI version in use, date/time of AI system use, patient ID, responsible clinical user ID, input data used by the AI system, AI recommendation or output.
- Organizations have an internal process to evaluate AI-enabled system performance on local data before routine clinical use and periodically following implementation to check for drift,[24] bias,[25] or decay,[26] for example.[27] This process should include ongoing regular testing of AI applications in the (live) production system to ensure the safe performance and safe use of these program's references.[28]
- Organizations have high-quality training programs for clinicians interested in using AI systems that focus on the known and potential risks of using these systems.
- Organizations have a formal consent-style process, complete with signatures, to ensure clinicians understand the risks and benefits of using AI tools before their access is enabled.
- Organizations must provide clear written instructions and authority to enable anyone in the organization's information technology department to disable, stop, or turn off the artificial intelligence-enabled systems, 24 hours a day, seven days a week, in the event of a problem.[29]
- Similar to an organization's preparation for an EHR downtime, organizations must have an established policy and procedure to manage clinical and administrative processes that have become dependent on AI automation, when the AI is not available.
- Organizations should have a clear process for reporting AI-related safety issues and a process for analyzing these issues and mitigating risks.[30]

Recommended Practice - Involve EHR Vendor

2.1 Healthcare organizations and EHR vendors share responsibility for identifying and addressing EHR safety concerns.

Checklist

Implementation Status

EHR Limitation

Rationale for Practice or Risk Assessment

By working together, healthcare organizations and EHR vendors leverage their respective expertise to prioritize and share responsibility for patient safety. Healthcare organizations bring firsthand knowledge of clinical workflows and real-world EHR applications, while vendors contribute technical expertise and understanding of the system's architecture. Through continuous collaboration, they can ensure EHR systems evolve to meet emerging needs, address new safety concerns, and optimize patient care. This joint approach fosters a culture of shared accountability, driving ongoing improvement and mitigating risks associated with EHRs and AI integration.

Assessment Notes

Follow-up Actions

Person Responsible for Follow-up Action

Reset

Suggested Sources of Input

1. Large organization: Board of directors, EHR vendors, Clinical and IT leadership team
2. Small organization: Owners, EHR developers

Strength of Recommendation

Medium

Implementation Guidance

- Organizations should have a documented process for monitoring information provided by the EHR vendor with regard to existing defects. When defects that previously required workarounds are resolved, users receive appropriate training.
- EHR vendors create their own set of system-specific guidance to help their clients configure their EHRs to meet the SAFER Guide recommendations.[31]
- Healthcare organizations and EHR vendors review the SAFER Guide recommendations annually.[31,32]
- EHR vendors are provided feedback from clinicians on potential safety enhancements to the system.[31,32]

Recommended Practice - Inbox Design, Configuration, and Management

2.2 The EHR inbox and its use is optimized to reduce inbox burden.

Checklist

Implementation Status

EHR Limitation

Rationale for Practice or Risk Assessment

Inbox configuration aligned with its effective and efficient management can help clinicians focus on important and high-priority information.

Assessment Notes

Follow-up Actions

Person Responsible for Follow-up Action

Reset

Suggested Sources of Input

1. Clinicians
2. Vendors
3. IT staff

Strength of Recommendation

Medium

Implementation Guidance

- High-priority messages, abnormal test results, or otherwise time-sensitive inbox messages and tasks are visually distinct from routine inbox communication.[16]
- The EHR allows users to organize and prioritize inbox content, including allowing sorting, filtering, and flagging features preferred by individual clinicians (e.g., based on date, source, patient, urgency, message type).[16,33]
- Inbox configuration and management allows support staff to triage and act on messages within their scope of practice (e.g., processing refill requests, communicating normal test results, scheduling visits) without requiring the clinician to read or sort through administrative and non-medical queries.[34,35]
- Inbox functionality includes the ability to flag, forward, and add comments to messages and tasks.[16,34]
- Out-of-office messaging functionality is enabled to make it clear to the sender that an inbox is not being monitored.[16]
- The EHR allows automatic message forwarding to a surrogate clinician during a specific time period or circumstance, such as when the clinician is absent from work.
- The organization’s clinical leadership actively works to identify and mitigate inbox-related burdens by implementing processes designed to facilitate team communication and streamline inbox content.[36]
- Appropriately tested and effective artificial intelligence solutions are integrated to help categorize messages and draft suggested responses to patients.[35]

Recommended Practice - Patient Photographs

2.3 Patient photographs are collected during patient registration and displayed in multiple places in the EHR to improve patient identification.[37]

Checklist

Implementation Status

EHR Limitation

Rationale for Practice or Risk Assessment

The display of color patient photographs in the main banner of an EHR, in patient lists, and in other areas of the EHR, when utilized either on desktop computers or mobile devices, is an effective, non-interruptive method to improve patient identification and reduce wrong patient errors.[37-42]

Assessment Notes

Follow-up Actions

Person Responsible for Follow-up Action

Reset

Suggested Sources of Input

1. EHR developer
2. Registration Staff

Strength of Recommendation

Strong

Implementation Guidance

- The organization collects a color photograph of every patient older than three months of age at the time of patient registration, admission to the hospital, or any time staff believe a change in appearance warrants updating the photograph.[37,38,43]
- Patient photographs are displayed in all screens and functions of the EHR supported by the vendor, including patient banners, patient lists, patient scheduling, patient search, and secure messaging.
- Patient photographs are displayed in the EHR in all devices supported by the vendor including desktop computers and mobile devices.
- Policies and practices, that are sensitive to patient cultural and religious practices with regard to face and head coverings, are developed and implemented that provide guidance for capturing patient photographs, including when and how to capture them, and describing the optimal patient photo (e.g., the patient's face is centered and greater than 50% of the image).
- Reports are utilized to monitor the compliance of capturing patient photographs, and performance improvement projects are utilized to improve compliance.
- When patient photographs are not supported by the vendor or not available, other functions are used to improve patient identification such as patient identification alerts or "re-entering" patient identifiers (e.g., initials, name) before signing orders.[7,44]

Recommended Practice - Test Result Follow-up

2.4 Written policies specify unambiguous responsibility for test result follow-up with a shared understanding of that responsibility among all involved in providing follow-up care.[45-54]

Checklist

Implementation Status

EHR Limitation

Rationale for Practice or Risk Assessment

New workflows resulting from the introduction of EHRs can introduce new hazards related to miscommunication of responsibility for follow-up. Ambiguous responsibility increases the risk of follow-up failure.[55,56]

Suggested Sources of Input

1. Clinicians, support staff, and/or clinical administration
2. Diagnostic services

Strength of Recommendation

Medium

Implementation Guidance

- In the outpatient setting, the ordering clinician is responsible for follow-up unless he or she delegates this responsibility (e.g., to a covering clinician). Delegation should be documented in the EHR and accepted by the delegate.[57,58]
- In organizations with trainees (e.g., residents or fellows), ultimate responsibility defaults to the supervising attending in the event of a change of service by the trainee acting as an ordering clinician.
- Ordering clinicians in any setting assume responsibility for follow-up care, unless that responsibility is unambiguously transferred to another clinician who accepts responsibility.[52]

Assessment Notes

Follow-up Actions

Person Responsible for Follow-up Action

Reset

Recommended Practice - Testing Updates

2.5 System hardware, operating and network software, and clinical application version updates, modifications, or local customizations are tested individually and in the context of other integrated systems using a standardized approach. This consists of:

- Testing before go-live and as installed in production to ensure adequate performance and data integrity
- Testing based on real-world, clinically authentic, and relevant scenarios incorporating collaborative workflows[59]
- Monitoring all systems for a short time following any hardware or software changes
- Notifying end users before, and reminded them after, potentially impactful changes to applications or clinical content assets

Checklist

Rationale for Practice or Risk Assessment

Failure to adequately test system hardware, software, and configuration or customization of clinical applications can lead to data integrity issues and impede response time, reliability, and error-free operation.

Assessment Notes

Follow-up Actions

Person Responsible for Follow-up Action

Implementation Status

EHR Limitation

Suggested Sources of Input

1. Health IT support staff
2. EHR vendor

Strength of Recommendation

Medium

Implementation Guidance

- Software enhancements and updates are installed and tested in a test environment prior to moving into the production environment.
- New versions of the EHR system are enabled in a test environment with functionality sufficient for end-to-end testing of multidisciplinary workflows prior to release in the live/production environment.
- Customizations made by the organization, department, or user are tested to ensure they do not adversely impact other aspects of the system or interoperability with internal or external systems.
- Simulation training is conducted for clinical processes such as order entry, pharmacy review, nurse notification, medication fill, medication administration, and multidisciplinary clinical documentation to ensure that the application addresses the organization's needs.
- The organization has created a comprehensive test plan that validates the performance of each major function, including screen appearance, the graphic representation of data, alerts, and the accurate generation of reports.[60]
- Data migration processes and protocols are in place to ensure data integrity after transmitting data from one EHR system to another, changing the format of data (e.g., free text to structured), and clinical code updates (e.g., SNOMED, ICD-10, LOINC).
- Users are provided with a concise, relevant summary of software or component updates that impact their workflows or the data they rely on.

Reset

Recommended Practice - Alerts and Reminders

2.6 CDS alerts and reminders provide unambiguous guidance in the correct clinical context at relevant points in the workflow. Alerts and reminders are informative, actionable, and judiciously limited to the most significant, patient-specific notifications.

Checklist

Implementation Status

EHR Limitation

Rationale for Practice or Risk Assessment

Well-designed and configured alerts within clinical workflows can promote patient safety and positive patient outcomes without overwhelming ordering providers and other clinical staff with irrelevant information. Whether they are warnings about critical drug interactions or notifications based on preventive care guidelines, alerts should be tiered by severity and clearly and concisely describe the next action to take. Careful consideration should be given to defining alert levels, determining the context in which they will fire, and understanding the risks and benefits of potential clinical workflow disruptions (e.g., hard stops that require documentation of override rationale or soft stops that are dismissible without further action).

Assessment Notes

Follow-up Actions

Person Responsible for Follow-up Action

Reset

Suggested Sources of Input

1. Clinicians
2. Clinical support staff
3. Clinical administration
4. Pharmacists
5. Nurses
6. Informatics staff
7. Health IT support staff
8. EHR developer

Strength of Recommendation

Medium

Implementation Guidance

- The organization's CDS governance has a process for developing, maintaining, and regularly revising alerts based on clinical user feedback, emerging knowledge, and high override rates.[61]
- The EHR allows users to provide feedback on CDS content directly within the workflow.[62]
- Alerts are designed to appear in the right place in the workflow for the right user (e.g., for the provider during order selection, the pharmacist during order fulfillment, and the nurse during medication administration).[63]
- If CDS uses AI such as a predictive model, the model's calculations are sufficiently explained (e.g., decision trees, templated text, or feature importance) along with its recommendations.[64]
- The organization has established standards limiting the use of interruptive alerts to only the most critical warnings.[65]
- Alerts requiring action include the ability to perform or jump directly to the intended action.[66]
- Interaction checking occurs for all active medications when a new allergy is entered (i.e., reverse checking).
- Dose range and maximum daily dose checking occur before medication orders are submitted for dispensing.
- Medication dosing alerts take into consideration relevant patient-specific data such as patient age, gender, and laboratory result values (e.g., metformin ordered for patients with impaired renal function as evidenced by decreased estimated glomerular filtration rate [eGFR]).[67,68]

Recommended Practice - Alerts and Reminders (cont'd)

2.6 CDS alerts and reminders provide unambiguous guidance in the correct clinical context at relevant points in the workflow. Alerts and reminders are informative, actionable, and judiciously limited to the most significant, patient-specific notifications.

Checklist

Implementation Guidance (cont'd)

- Order sets are configured to facilitate appropriate corollary or consequent orders and reflect changes made to the original order (e.g., rescheduling, renewing, or discontinuing).[69]
- Incomplete orders requiring further actions (e.g., answers to specific questions) are clearly communicated to the ordering provider during order entry and prior to submission.
- The organization has a robust process for managing feedback, responding to users, and tracking improvements made.[62]

Recommended Practice - Ransomware

2.7 Users are trained on ransomware prevention strategies, including how to identify malicious emails and fraudulent telephone callers asking for login access or other privileged information.[70,71]

Checklist

Implementation Status

EHR Limitation

Rationale for Practice or Risk Assessment

Malicious email attachments or callers asking for personal login information are often the first point of entry for ransomware attacks.

Suggested Sources of Input

1. Clinicians, support staff, and/or clinical administration
2. EHR developer

Strength of Recommendation

Required

Implementation Guidance

- Users are trained to first, hover over links to see the URL (Uniform Resource Locator) destination before clicking, and second, think about the attachment or link - do you know the sender, does the email have a sense of urgency or deadline to take action, are their spelling or grammatical errors in the message? Do not click on the link or attachment if not sure. When in doubt call or email (in a separate email) the sender or the organization requesting information to confirm it is legitimate.[72]
- The organization trains users to identify spam, phishing, and spear-phishing messages, and users avoid clicking on potentially weaponized attachments (such as *.exe,
- *.zip, *.rar, *.7z, *.js, *.wsf, *.docm, *.xlsm, *.pptm, *.rtf, *.msi, *.bat, *.com, *.cmd, *.hta, *.scr, *.pif, *.reg, *.vbs, *.cpl, *.jar files). Safer file attachment formats include (*.jpg, *.png, *.pdf, *.docx, *.xlsx, and *.pptx).[73,74]
- Training should reinforce that legitimate organizational mail messages (e.g., your employer's IT department, your bank, your credit card company, companies you work with) should always meet the following requirements: 1) never ask you to download and open file attachments; 2) never ask for you to enter account or password information; 3) always have a telephone number you can call (i.e., out-of-band check); 4) always be associated with an email address and name that people can check in their local directory; and 5) contain website links that display the complete internet address (URL) to build trust.
- The organization restricts users' ability to install and run software applications using the principle of "Least Privilege", or minimizes users' access to only those systems, services, and data required by their job.
- The organization considers disabling the USB ports on the organization's computers.[75]
- The organization conducts simulated phishing attacks (i.e., sends fraudulent [but safe] email messages or websites that appear to be from legitimate sources) to raise user's awareness of the problem.[76]
- The organization conducts simulated ransomware attack detection and recovery drills from both the clinical[77] and technical[78] perspectives.

Assessment Notes

Follow-up Actions

Person Responsible for Follow-up Action

Reset

> Table of Contents | > About the Checklist | > Team Worksheet | > About the Practice Worksheets | >Practice Worksheets

Recommended Practice - Training on Downtime

2.8 Staff are trained and tested on downtime and recovery procedures.

Checklist

Implementation Status

EHR Limitation

Rationale for Practice or Risk Assessment

At any given time, many organizations are likely to have employees who do not know how to function in a paper record-based clinical or administrative environment.[80]

Suggested Sources of Input

1. Clinicians, support staff, and/or clinical administration

Strength of Recommendation

Required

Implementation Guidance

- Organizations establish and follow training requirements so that each employee knows what to do to keep the organization operating safely during EHR downtimes.[81]
- Clinicians are trained in the use of paper-based ordering and charting tools.
- The organization offers a job aid, such as a small, self-contained reference card or checklist, to help clinical staff find available resources and actions during EHR downtimes.[82]
- The organization conducts unannounced EHR "downtime drills" at least once a year.[83]
- Clinicians have been trained on how and when to activate and use the "read-only" backup EHR system.[84]
- Clinicians and other staff members have reliable access to the login information for the emergency, downtime, read-only backup EHR system, which may be different than userspecific credentials used for the live or production EHR.
- The organization maintains a comprehensive list of system-to-system interfaces or computer connections that is reviewed on a regular basis (e.g., every six months or annually) as a part of on-going contingency planning. The list should have a specific indication of whether there are legal/ regulatory issues that may require special notification to the other party if there is a downtime such as a state-based immunization registry or prescription drug monitoring program.[85]

Assessment Notes

Follow-up Actions

Person Responsible for Follow-up Action

Reset

Recommended Practice - Risk Management Strategy

3.1 Organizations have a strategy and mechanisms for prevention, identification, measurement, monitoring, and mitigation of high priority EHR safety risks and hazards.

Checklist

Implementation Status

EHR Limitation

Rationale for Practice or Risk Assessment

A robust strategy enables organizations to proactively manage potential hazards, mitigating the most significant threats to patient safety by focusing on high-priority EHR safety risks. This approach is not only crucial for patient safety but also often mandated by regulatory bodies. By identifying high-priority risks, organizations can allocate resources effectively, targeting efforts on critical areas and informing targeted training programs to enhance clinicians' safe and effective use of the EHR system. This proactive risk management enables healthcare organizations to minimize adverse events, optimize EHR performance, and ensure compliance with regulatory requirements.

Suggested Sources of Input

1. Large organization: Board of directors, Clinical, Informatics, and IT leadership team, Safety officer
2. Small organization: Owners, EHR vendors, Clinicians

Strength of Recommendation

Medium

Implementation Guidance

- A plan exists for learning from incidents to improve EHR safety.[86-88]
- Real-time monitoring tools are deployed that track system performance, detect anomalies, and alert IT staff to potential issues.[89]
- Organization EHR representatives meet regularly with the EHR vendor to discuss new or ongoing issues.
- Bidirectional communication between the organization and the EHR vendor ensures timely updates, patches, and support for the system.
- A multi-stakeholder committee or task force convenes on a regular basis to review all high-priority EHR-related hazards.[90,91]
- EHR-related incidents are categorized and summarized by location (i.e., clinical and within the EHR), severity, and type to assess for any trends that need to be addressed.[92]
- The mechanism for anonymous, no-fault, internal reporting of EHR-related safety hazards is clear to all users.[90]
- Organization has a policy and procedure that addresses timeliness of addressing reported errors, including an escalation process to organization leadership when the established service level is not being met or is at risk of not being met.
- Larger organizations use specialized “help desk” software to manage internal EHR error reports and their disposition.
- The user who reported the issue, if identified, should be notified of the outcome when appropriate.
- The organization regularly monitors and reports on system downtime events.[93]

Assessment Notes

Follow-up Actions

Person Responsible for Follow-up Action

Reset

Recommended Practice - Patient Notification

3.2 Organizational policies and procedures ensure timely patient notification of both normal and abnormal test results, and the timeliness of notification is monitored.[57]

Checklist

Implementation Status

EHR Limitation

Rationale for Practice or Risk Assessment

Failure in timely patient notification of test results is a major source of diagnostic error and liability. Standardized policies and procedures for timely patient notification reduce the risk of loss of follow-up.

Assessment Notes

Follow-up Actions

Person Responsible for Follow-up Action

Suggested Sources of Input

1. Clinicians, support staff, and/or clinical administration
2. Diagnostic services

Strength of Recommendation

Required

Implementation Guidance

- National VA policy “Communicating Test Results to Providers and Patients” Directive 1088[57] states that: *“It is VHA policy that all test results must be communicated by the diagnostic provider to the ordering provider, or designee, within a time-frame that allows for prompt attention and appropriate action to be taken. All test results requiring action must be communicated by the ordering provider, or designee, to patients no later than 7 calendar days from the date on which the results are available. For test results that require no action, results must be communicated by the ordering provider, or designee, to patients no later than 14 calendar days from the date on which the results are available. Depending on the clinical context, certain test results may require review and communication in shorter time-frames.”*
- Notification of test results to patients is monitored for timeliness (i.e., whether the clinician notified the patient within the correct time frame).
- Certain time-sensitive test results, as well as results for which clear, unambiguous communication is essential (e.g., HIV status, cancer diagnosis), are discussed in person or via the telephone rather than using asynchronous electronic means (e.g., secure messaging, voicemail, or patient portals).
- Organizations use patient portals to automatically release test results to patients who have activated their accounts. To explain their test results in more detail, portal users are provided with a link to lab test interpretations (https://www.testing.com/news/labtestsonline-org-is-now-testing-com/).
- For patients who have not activated their online accounts, traditional methods such as letters or phone calls are used to inform them of their results on a timely basis.
- If patient communication and acknowledgment of abnormal results are unable to be confirmed, alternative strategies are used to ensure follow-up (e.g., if the secure message is not read, telephone or send a letter).

Reset

Recommended Practice - Monitor Communication Patterns

3.3 The EHR enables the monitoring of important communication patterns related to clinical messages, referrals, and patient portal notifications.

Checklist

Implementation Status

EHR Limitation

Rationale for Practice or Risk Assessment

Monitoring time-sensitive and important clinical communications can identify opportunities to improve safety by identifying and addressing potential problems related to informing and responding to messages between clinicians and the care team and ancillary staff, as well as to and from patients.

Assessment Notes

Follow-up Actions

Person Responsible for Follow-up Action

Suggested Sources of Input

1. Clinicians
2. Clinical staff
3. Quality improvement staff
4. Health IT support staff
5. Vendors

Strength of Recommendation

Medium

Implementation Guidance

- The organization monitors rates of unacknowledged clinician inbox messages, messages sent to patients in their portal's inbox, and tasks.[94]
- The organization defines and tracks expected response time frames for specific types of messages (e.g., urgent referrals responded to within two days, hospital discharge summaries sent to primary care provider within three days of discharge, inpatient order to admit is signed off at or before the time of admission). Findings are used to identify and resolve any deficiencies.
- Inbox message monitoring identifies quality improvement projects and targets interventions for clinicians with higher rates of unacknowledged inbox messages and tasks.[95]
- Physician burnout, turnover, productivity, and EHR use metrics are analyzed to identify opportunities to identify physicians at high risk of departure who may benefit from targeted inbox management interventions.[96,97]
- Patient portal adoption and utilization rates are monitored and analyzed, including how these differ by patient language, race/ethnicity, and other demographics.[98]
- Messaging content and response patterns are periodically reviewed to identify opportunities for improving communication quality.[99]
- The organization provides sufficient administrative time for clinicians to appropriately manage inbox messages and the clinical work associated with them.[33,34,100]

Reset

References

1. Azyabi A, Karwowski W, Davahli MR. Assessing patient safety culture in hospital settings. Int J Environ Res Public Health. 2021;18(5):2466. https://pubmed.ncbi.nlm.nih.gov/33802265/. doi: 10.3390/ijerph18052466; PMID: 33802265; PMC7967599.

2. Fennelly O, Cunningham C, Grogan L, et al. Successfully implementing a national electronic health record: A rapid umbrella review. Int J Med Inform. 2020;144:104281. https://pubmed.ncbi.nlm.nih.gov/33017724/. doi: 10.1016/j.ijmedinf.2020.104281; PMID: 33017724; PMC7510429.

3. Campione JR, Mardon RE, McDonald KM. Patient safety culture, health information technology implementation, and medical office problems that could lead to diagnostic error. J Patient Saf. 2019;15(4):267–273. https://pubmed.ncbi.nlm.nih.gov/30138158/. doi: 10.1097/PTS.0000000000000531; PMID: 30138158.

4. Jianxun C, Arkorful VE, Shuliang Z. Electronic health records adoption: Do institutional pressures and organizational culture matter? Technology in Society. 2021;65:101531. https://www.sciencedirect.com/science/article/pii/S0160791X21000063. doi: 10.1016/j.techsoc.2021.101531.

5. Lowry SZ, Quinn MT, Ramaiah M, et al. Technical evaluation, testing, and validation of the usability of electronic health records. national institute of standards and technology interagency. NISTIR. 2012(780). https://www.nist.gov/publications/nistir-7804-technical-evaluation-testing-and-validation-usability-electronic-health.

6. Khunlertkit A, Dorissaint L, Chen A, Paine L, Pronovost PJ. Reducing and sustaining duplicate medical record creation by usability testing and system redesign. J Patient Saf. 2021;17(7):e665–e671. https://pubmed.ncbi.nlm.nih.gov/29076957/. doi: 10.1097/PTS.0000000000000434; PMID: 29076957.

7. Adelman JS, Aschner JL, Schechter CB, et al. Evaluating serial strategies for preventing wrong-patient orders in the NICU. Pediatrics. 2017;139(5):e20162863. https://pubmed.ncbi.nlm.nih.gov/28557730/. doi: 10.1542/peds.2016-2863; PMID: 28557730.

8. The U.S. National Archives and Records Administration. Soundex system | the soundex indexing system. www.archives.gov Web site. https://www.archives.gov/research/census/soundex. Updated 2024. Accessed Aug 9, 2024.

9. Ferrera-Tourenc V, Lassale B, Chiaroni J, Dettori I. Unreliable patient identification warrants ABO typing at admission to check existing records before transfusion. Transfus Clin Biol. 2015;22(2):66–70. https://pubmed.ncbi.nlm.nih.gov/25936944/. doi: 10.1016/j.tracli.2015.03.004; PMID: 25936944.

10. Sittig DF, Gonzalez D, Singh H. Contingency planning for electronic health record-based care continuity: A survey of recommended practices. Int J Med Inform. 2014;83(11):797–804. https://pubmed.ncbi.nlm.nih.gov/25200197/. doi: 10.1016/j.ijmedinf.2014.07.007; PMID: 25200197.

11. T. C. Piliouras, R. J. Suss, P. L. Yu. Digital imaging & electronic health record systems: Implementation and regulatory challenges faced by healthcare providers. . 2015:1–6. https://ieeexplore.ieee.org/document/7160179. doi: 10.1109/LISAT.2015.7160179.

12. Schweitzer EJ. Reconciliation of the cloud computing model with US federal electronic health record regulations. J Am Med Inform Assoc. 2012;19(2):161–165. https://pubmed.ncbi.nlm.nih.gov/21727204/. doi: 10.1136/amiajnl-2011-000162; PMID: 21727204; PMC3277612.

13. Schackow TE, Palmer T, Epperly T. EHR meltdown: How to protect your patient data. Fam Pract Manag. 2008;15(6):3. https://pubmed.ncbi.nlm.nih.gov/18595558/; PMID: 18595558.

14. Thomas J, Galligher G. Improving backup system evaluations in information security risk assessments to combat ransomware. Compute and Information Science. 2018;11(1). https://www.ccsenet.org/journal/index.php/cis/article/view/72481. doi: 10.5539/cis.v11n1p14.

References

15. ECRI Institute. Closing the loop: Using health IT to mitigate delayed, missed, and incorrect diagnoses related to diagnostic testing and medication changes. . www.ecri.org Web site. https://www.ecri.org/Resources/HIT/Closing_Loop/Closing_the_Loop_Toolkit.pdf. Updated 2018. Accessed Jul 15, 2024.

16. Murphy DR, Giardina TD, Satterly T, Sittig DF, Singh H. An exploration of barriers, facilitators, and suggestions for improving electronic health record inbox-related usability: A qualitative analysis. JAMA Netw Open. 2019;2(10):e1912638. https://pubmed.ncbi.nlm.nih.gov/31584683/. doi: 10.1001/jamanetworkopen.2019.12638; PMID: 31584683; PMC6784746.

17. Murphy DR, Reis B, Sittig DF, Singh H. Notifications received by primary care practitioners in electronic health records: A taxonomy and time analysis. Am J Med. 2012;125(2):209.e1–7. https://pubmed.ncbi.nlm.nih.gov/22269625/. doi: 10.1016/j.amjmed.2011.07.029; PMID: 22269625.

18. Murphy DR, Reis B, Kadiyala H, et al. Electronic health record-based messages to primary care providers: Valuable information or just noise? Arch Intern Med. 2012;172(3):283–285. https://pubmed.ncbi.nlm.nih.gov/22332167/. doi: 10.1001/archinternmed.2011.740; PMID: 22332167.

19. Jin J, Jerzak J, Sinsky CA. Taming the EHR playbook implement effective system-level policies to reduce the burden of EHR work. www.edhub.ama-assn.org Web site. https://edhub.ama-assn.org/steps-forward/module/2798925. Updated 2022. Accessed Aug 6, 2024.

20. Office of the National Coordinator for Health Information Technology, (ONC). Health sector AI commitments. www.healthit.gov Web site. https://www.healthit.gov/sites/default/files/2023-12/Health_Sector_AI_Commitments_FINAL_120923.pdf. Updated 2023. Accessed Jul 24, 2024.

21. The White House. Executive order on the safe, secure, and trustworthy development and use of artificial intelligence. www.whitehouse.gov Web site. https://www.whitehouse.gov/briefing-room/presidential-actions/2023/10/30/executive-order-on-the-safe-secure-and-trustworthy-development-and-use-of-artificial-intelligence/. Updated 2023. Accessed Jul 24, 2024.

22. Daneshvar N, Pandita D, Erickson S, Snyder Sulmasy L, DeCamp M. Artificial intelligence in the provision of health care: An american college of physicians policy position paper. Ann Intern Med. 2024;177(7):964–967. https://pubmed.ncbi.nlm.nih.gov/38830215/. doi: 10.7326/M24-0146; PMID: 38830215.

23. American Medical Association. Principles for augmented intelligence development, deployment, and use. www.ama-assn.org Web site. https://www.ama-assn.org/system/files/ama-ai-principles.pdf. Updated 2023. Accessed Jul 24, 2024.

24. Feng J, Phillips RV, Malenica I, et al. Clinical artificial intelligence quality improvement: Towards continual monitoring and updating of AI algorithms in healthcare. NPJ Digit Med. 2022;5(1):66. https://pubmed.ncbi.nlm.nih.gov/35641814/. doi: 10.1038/s41746-022-00611-y; PMID: 35641814; PMC9156743.

25. Chin MH, Afsar-Manesh N, Bierman AS, et al. Guiding principles to address the impact of algorithm bias on racial and ethnic disparities in health and health care. JAMA Netw Open. 2023;6(12):e2345050. https://pubmed.ncbi.nlm.nih.gov/38100101/. doi: 10.1001/jamanetworkopen.2023.45050; PMID: 38100101; PMC11181958.

26. Vela D, Sharp A, Zhang R, Nguyen T, Hoang A, Pianykh OS. Temporal quality degradation in AI models. Sci Rep. 2022;12(1):11654. https://pubmed.ncbi.nlm.nih.gov/35803963/. doi: 10.1038/s41598-022-15245-z; PMID: 35803963; PMC9270447.

27. Handley JL, Lehmann CU, Ratwani RM. Safe and equitable pediatric clinical use of AI. JAMA Pediatr. 2024;178(7):637–638. https://pubmed.ncbi.nlm.nih.gov/38739385/. doi: 10.1001/jamapediatrics.2024.0897; PMID: 38739385.

References

28. Ratwani RM, Bates DW, Classen DC. Patient safety and artificial intelligence in clinical care. JAMA Health Forum. 2024;5(2):e235514. https://pubmed.ncbi.nlm.nih.gov/38393719/. doi: 10.1001/jamahealthforum.2023.5514; PMID: 38393719.

29. Anthropic's responsible scaling policy, version 1.0. https://www-cdn.anthropic.com/1adf000c8f675958c2ee23805d91aaade1cd4613/responsible-scaling-policy.pdf. Updated 2023. Accessed Jul 25, 2024.

30. Petersen C, Smith J, Freimuth RR, et al. Recommendations for the safe, effective use of adaptive CDS in the US healthcare system: An AMIA position paper. J Am Med Inform Assoc. 2021;28(4):677–684. https://pubmed.ncbi.nlm.nih.gov/33447854/. doi: 10.1093/jamia/ocaa319; PMID: 33447854; PMC7973467.

31. Sittig DF, Ash JS, Wright A, et al. How can we partner with electronic health record vendors on the complex journey to safer health care? J Healthc Risk Manag. 2020;40(2):34–43. https://pubmed.ncbi.nlm.nih.gov/32648286/. doi: 10.1002/jhrm.21434; PMID: 32648286.

32. Hettinger AZ, Melnick ER, Ratwani RM. Advancing electronic health record vendor usability maturity: Progress and next steps. J Am Med Inform Assoc. 2021;28(5):1029–1031. https://pubmed.ncbi.nlm.nih.gov/33517394/. doi: 10.1093/jamia/ocaa329; PMID: 33517394; PMC8068416.

33. Murphy DR, Meyer AND, Russo E, Sittig DF, Wei L, Singh H. The burden of inbox notifications in commercial electronic health records. JAMA Intern Med. 2016;176(4):559–560. https://www.ncbi.nlm.nih.gov/pmc/articles/PMC4860883/. doi: 10.1001/jamainternmed.2016.0209; PMID: 26974737; PMC4860883.

34. Murphy DR, Satterly T, Giardina TD, Sittig DF, Singh H. Practicing clinicians' recommendations to reduce burden from the electronic health record inbox: A mixed-methods study. J Gen Intern Med. 2019;34(9):1825–1832. https://pubmed.ncbi.nlm.nih.gov/31292905/. doi: 10.1007/s11606-019-05112-5; PMID: 31292905; PMC6712240.

35. Rotenstein LS, Landman A, Bates DW. The electronic inbox-benefits, questions, and solutions for the road ahead. JAMA. 2023;330(18):1735–1736. https://pubmed.ncbi.nlm.nih.gov/37812413/. doi: 10.1001/jama.2023.19195; PMID: 37812413.

36. Dymek C, Kim B, Melton GB, Payne TH, Singh H, Hsiao C. Building the evidence-base to reduce electronic health record-related clinician burden. J Am Med Inform Assoc. 2021;28(5):1057–1061. https://pubmed.ncbi.nlm.nih.gov/33340326/. doi: 10.1093/jamia/ocaa238; PMID: 33340326; PMC8068419.

37. The Joint Commission. Quick safety - people, processes, health IT and accurate patient identification. www.jointcommission.org Web site. https://www.jointcommission.org/-/media/tjc/newsletters/qs_hit_and_patient_id_9_25_18_finalpdf.pdf. Updated 2018. Accessed Aug 9, 2024.

38. Riplinger L, Piera-Jiménez J, Dooling JP. Patient identification techniques - approaches, implications, and findings. Yearb Med Inform. 2020;29(1):81–86. https://pubmed.ncbi.nlm.nih.gov/32823300/. doi: 10.1055/s-0040-1701984; PMID: 32823300; PMC7442501.

39. Van Hal C, Mills JL, Gatmaitan M, Gong Y. A patient-centered approach to collecting and displaying patient identifiers. Stud Health Technol Inform. 2024;310:369–373. https://pubmed.ncbi.nlm.nih.gov/38269827/. doi: 10.3233/SHTI230989; PMID: 38269827.

40. Fortman E, Hettinger AZ, Howe JL, et al. Varying rates of patient identity verification when using computerized provider order entry. J Am Med Inform Assoc. 2020;27(6):924–928. https://pubmed.ncbi.nlm.nih.gov/32377679/. doi: 10.1093/jamia/ocaa047; PMID: 32377679; PMC7647277.

41. Salmasian H, Blanchfield BB, Joyce K, et al. Association of display of patient photographs in the electronic health record with wrong-patient order entry errors. JAMA Netw Open. 2020;3(11):e2019652. https://pubmed.ncbi.nlm.nih.gov/33175173/. doi: 10.1001/jamanetworkopen.2020.19652; PMID: 33175173; PMC7658731.

42. Rzewnicki D, Kanvinde A, Gillespie S, Orenstein E. Association of patient photographs and reduced retract-and-reorder events. JAMIA Open. 2024;7(3):ooae042.

References

43. Hyman D, Laire M, Redmond D, Kaplan DW. The use of patient pictures and verification screens to reduce computerized provider order entry errors. Pediatrics. 2012;130(1):211. https://pubmed.ncbi.nlm.nih.gov/22665415/. doi: 10.1542/peds.2011-2984; PMID: 22665415.

44. Adelman JS, Kalkut GE, Schechter CB, et al. Understanding and preventing wrong-patient electronic orders: A randomized controlled trial. J Am Med Inform Assoc. 2013;20(2):305–310. https://pubmed.ncbi.nlm.nih.gov/22753810/. doi: 10.1136/amiajnl-2012-001055; PMID: 22753810; PMC3638184.

45. Sittig DF, Singh H. Improving test result follow-up through electronic health records requires more than just an alert. J Gen Intern Med. 2012;27(10):1235–1237. https://pubmed.ncbi.nlm.nih.gov/22790618/. doi: 10.1007/s11606-012-2161-y; PMID: 22790618; PMC3445682.

46. Singh H, Vij MS. Eight recommendations for policies for communicating abnormal test results. Jt Comm J Qual Patient Saf. 2010;36(5):226–232. https://pubmed.ncbi.nlm.nih.gov/20480756/. doi: 10.1016/s1553-7250(10)36037-5; PMID: 20480756.

47. Singh H, Thomas EJ, Mani S, et al. Timely follow-up of abnormal diagnostic imaging test results in an outpatient setting; are electronic medical records achieving their potential? Arch Int Med. 2009;169(17):1578–1586. pubmed.ncbi.nlm.nih.gov/19786677/; PMID: 19786677; PMC2919821.

48. Callen JL, Westbrook JI, Georgiou A, Li J. Failure to follow-up test results for ambulatory patients: A systematic review. J Gen Intern Med. 2012;27(10):1334–1348. https://pubmed.ncbi.nlm.nih.gov/22183961/. doi: 10.1007/s11606-011-1949-5; PMID: 22183961; PMC3445672.

49. Dalal AK, Poon EG, Karson AS, Gandhi TK, Roy CL. Lessons learned from implementation of a computerized application for pending tests at hospital discharge. J Hosp Med. 2011;6(1):16–21. https://pubmed.ncbi.nlm.nih.gov/21241037/. doi: 10.1002/jhm.794; PMID: 21241037.

50. Litchfield I, Bentham L, Lilford R, McManus RJ, Hill A, Greenfield S. Test result communication in primary care: A survey of current practice. BMJ Qual Saf. 2015;24(11):691–699. https://pubmed.ncbi.nlm.nih.gov/26243888/. doi: 10.1136/bmjqs-2014-003712; PMID: 26243888; PMC4680128.

51. Litchfield IJ, Bentham LM, Lilford RJ, Greenfield SM. Test result communication in primary care: Clinical and office staff perspectives. Fam Pract. 2014;31(5):592–597. https://pubmed.ncbi.nlm.nih.gov/25070182/. doi: 10.1093/fampra/cmu041; PMID: 25070182; PMC4169669.

52. Roy CL, Rothschild JM, Dighe AS, et al. An initiative to improve the management of clinically significant test results in a large health care network. Jt Comm J Qual Patient Saf. 2013;39(11):517–527. https://pubmed.ncbi.nlm.nih.gov/24294680/. doi: 10.1016/s1553-7250(13)39068-0; PMID: 24294680.

53. Dalal AK, Schnipper JL, Poon EG, et al. Design and implementation of an automated email notification system for results of tests pending at discharge. J Am Med Inform Assoc. 2012;19(4):523–528. https://pubmed.ncbi.nlm.nih.gov/22268214/. doi: 10.1136/amiajnl-2011-000615; PMID: 22268214; PMC3384118.

54. Hysong SJ, Sawhney MK, Wilson L, et al. Understanding the management of electronic test result notifications in the outpatient setting. BMC Med Inform Decis Mak. 2011;11:22. https://pubmed.ncbi.nlm.nih.gov/21486478/. doi: 10.1186/1472-6947-11-22; PMID: 21486478; PMC3100236.

55. Powell L, Sittig DF, Chrouser K, Singh H. Assessment of health information Technology–Related outpatient diagnostic delays in the US veterans affairs health care system. JAMA Netw Open. 2020;3(6):e206752. https://www.ncbi.nlm.nih.gov/pmc/articles/PMC7317596/. doi: 10.1001/jamanetworkopen.2020.6752; PMID: 32584406; PMC7317596.

References

56. Kwan JL, Singh H. Assigning responsibility to close the loop on radiology test results. Diagnosis (Berl). 2017;4(3):173–177. https://www.ncbi.nlm.nih.gov/pmc/articles/PMC5673267/. doi: 10.1515/dx-2017-0019; PMID: 29119073; PMC5673267.

57. Department of Veteran Affairs. VHA directive 1088: Communicating test results to providers and patients. https://www.va.gov/vhapublications/ViewPublication.asp?pub_ID=10366. Updated 2023. Accessed Jul 15, 2024.

58. Atlas SJ, Tosteson ANA, Wright A, et al. A multilevel primary care intervention to improve follow-up of overdue abnormal cancer screening test results: A cluster randomized clinical trial. JAMA. 2023;330(14):1348–1358. https://pubmed.ncbi.nlm.nih.gov/37815566/. doi: 10.1001/jama.2023.18755; PMID: 37815566; PMC10565610.

59. Tutty MA, Carlasare LE, Lloyd S, Sinsky CA. The complex case of EHRs: Examining the factors impacting the EHR user experience. J Am Med Inform Assoc. 2019;26(7):673–677. https://pubmed.ncbi.nlm.nih.gov/30938754/. doi: 10.1093/jamia/ocz021; PMID: 30938754; PMC6562154.

60. Aguirre RR, Suarez O, Fuentes M, Sanchez-Gonzalez MA. Electronic health record implementation: A review of resources and toolshttps://Pubmed.ncbi.nlm.nih.gov/31700751/. Cureus. 2019;11(9):e5649. pubmed.ncbi.nlm.nih.gov/31700751/. doi: 10.7759/cureus.5649; PMID: 31700751; PMC6822893.

61. McGreevey JD, Mallozzi CP, Perkins RM, Shelov E, Schreiber R. Reducing alert burden in electronic health records: State of the art recommendations from four health systems. Appl Clin Inform. 2020;11(1):1–12. https://pubmed.ncbi.nlm.nih.gov/31893559/. doi: 10.1055/s-0039-3402715; PMID: 31893559; PMC6938713.

62. Rubins D, McCoy AB, Dutta S, et al. Real-time user feedback to support clinical decision support system improvement. Appl Clin Inform. 2022;13(5):1024–1032. https://pubmed.ncbi.nlm.nih.gov/36288748/. doi: 10.1055/s-0042-1757923; PMID: 36288748; PMC9605820.

63. Schreiber R, McGreevey JD. Chapter 20 - CDS governance and implementation. In: Greenes RA, Del Fiol G, eds. Clinical decision support and beyond (third edition). Oxford: Academic Press; 2023:561–601. https://www.sciencedirect.com/science/article/pii/B9780323912006000097. 10.1016/B978-0-323-91200-6.00009-7.

64. Gombolay GY, Silva A, Schrum M, et al. Effects of explainable artificial intelligence in neurology decision support. Ann Clin Transl Neurol. 2024;11(5):1224–1235. https://www.ncbi.nlm.nih.gov/pmc/articles/PMC11093252/. doi: 10.1002/acn3.52036.

65. Chaparro JD, Beus JM, Dziorny AC, et al. Clinical decision support stewardship: Best practices and techniques to monitor and improve interruptive alerts. Appl Clin Inform. 2022;13(3):560–568. https://pubmed.ncbi.nlm.nih.gov/35613913/. doi: 10.1055/s-0042-1748856; PMID: 35613913; PMC9132737.

66. McGreevey JD, Mallozzi CP, Perkins RM, Shelov E, Schreiber R. Reducing alert burden in electronic health records: State of the art recommendations from four health systems. Appl Clin Inform. 2020;11(1):1–12. pubmed.ncbi.nlm.nih.gov/31893559/. doi: 10.1055/s-0039-3402715; PMID: 31893559; PMC6938713.

67. Al-Jazairi AS, AlQadheeb EK, AlShammari LK, et al. Clinical validity assessment of integrated dose range checking tool in a tertiary care hospital using an electronic health information system. Hosp Pharm. 2021;56(2):95–101. https://pubmed.ncbi.nlm.nih.gov/33790484/. doi: 10.1177/0018578719867663; PMID: 33790484; PMC7958367.

68. Ibáñez-Garcia S, Rodriguez-Gonzalez C, Escudero-Vilaplana V, et al. Development and evaluation of a clinical decision support system to improve medication safety. Appl Clin Inform. 2019;10(3):513–520. https://pubmed.ncbi.nlm.nih.gov/31315138/. doi: 10.1055/s-0039-1693426; PMID: 31315138; PMC6637024.

69. Page N, Baysari MT, Westbrook JI. A systematic review of the effectiveness of interruptive medication prescribing alerts in hospital CPOE systems to change prescriber behavior and improve patient safety. Int J Med Inform. 2017;105:22–30. https://pubmed.ncbi.nlm.nih.gov/28750908/. doi: 10.1016/j.ijmedinf.2017.05.011; PMID: 28750908.

References

70. Healthcare & Public Health Sector Coordinating Council. Health industry cybersecurity practices: Managing threats and protecting patients. hhs.gov Web site. https://405d.hhs.gov/Documents/HICP-Main-508.pdf. Updated 2023. Accessed Jun 8, 2024.

71. U.S. Department of Health and Human Services, Office for Civil Rights. Fact sheet: Ransomware and HIPAA. hhs.gov Web site. https://www.hhs.gov/hipaa/for-professionals/security/guidance/cybersecurity/ransomware-fact-sheet/index.html. Updated 2021. Accessed Jun 7, 2024.

72. Sittig DF, Singh H. A socio-technical approach to preventing, mitigating, and recovering from ransomware attacks. Appl Clin Inform. 2016;7(2):624–632. https://www.ncbi.nlm.nih.gov/pmc/articles/PMC4941865/. doi: 10.4338/ACI-2016-04-SOA-0064.

73. Greenberg A. Hackers behind the change healthcare ransomware attack just received a $22 million payment. Wired. 2024. https://www.wired.com/story/alphv-change-healthcare-ransomware-payment/.

74. Souppaya M, Scarfone K. National institute of standards and technology special publication 800-83 revision 1, guide to malware incident prevention and handling for desktops and laptops. Natl. Inst. Stand. Technol. Spec. Publ. 2013;800-83r1:47. https://csrc.nist.rip/external/nvlpubs.nist.gov/nistpubs/SpecialPublications/NIST.SP.800-83r1.pdf. doi: 10.6028/NIST.SP.800-83r1.

75. Wright A, Sittig DF. Security threat posed by USB-based personal health records. Ann Intern Med. 2007;146(4):314–315. https://pubmed.ncbi.nlm.nih.gov/17310061/. doi: 10.7326/0003-4819-146-4-200702200-00020; PMID: 17310061.

76. Gordon WJ, Wright A, Aiyagari R, et al. Assessment of employee susceptibility to phishing attacks at US health care institutions. JAMA Netw Open. 2019;2(3):e190393. pubmed.ncbi.nlm.nih.gov/30848810/. doi: 10.1001/jamanetworkopen.2019.0393; PMID: 30848810; PMC6484661.

77. Marsh-Armstrong B, Pacheco F, Dameff C, Tully J. Design and pilot study of a high-fidelity medical simulation of a hospital-wide cybersecurity attack. Res Sq. 2024:rs.3.rs–3959502. https://pubmed.ncbi.nlm.nih.gov/38645079/. doi: 10.21203/rs.3.rs-3959502/v1; PMID: 38645079; PMC11030511.

78. Angafor G, Yevseyeva I, Maglaras L. MalAware: A tabletop exercise for malware security awareness education and incident response training. Internet of Things and Cyber-Physical Systems. 2024;4:280–292. https://www.sciencedirect.com/science/article/pii/S2667345224000063. doi: 10.1016/j.iotcps.2024.02.003.

79. Lyon R, Jones A, Burke R, Baysari MT. What goes up, must come down: A state-of-the-art electronic health record downtime and uptime procedure in a metropolitan health setting. Appl Clin Inform. 2023;14(3):513–520. https://pubmed.ncbi.nlm.nih.gov/37406674/. doi: 10.1055/s-0043-1768995; PMID: 37406674; PMC10322225.

80. Larsen EP, Haskins Lisle A, Law B, Gabbard JL, Kleiner BM, Ratwani RM. Identification of design criteria to improve patient care in electronic health record downtime. J Patient Saf. 2021;17(2):90–94. https://pubmed.ncbi.nlm.nih.gov/30747861/. doi: 10.1097/PTS.0000000000000580; PMID: 30747861.

81. Oral B, Cullen RM, Diaz DL, Hod EA, Kratz A. Downtime procedures for the 21st century: Using a fully integrated health record for uninterrupted electronic reporting of laboratory results during laboratory information system downtimes. Am J Clin Pathol. 2015;143(1):100–104. https://pubmed.ncbi.nlm.nih.gov/25511148/. doi: 10.1309/AJCPM0O7MNVGCEVT; PMID: 25511148.

82. Gecomo JG, Klopp A, Rouse M, Online Journal of Nursing Informatics contributors. Implementation of an evidence-based electronic health record (EHR) downtime readiness and recovery plan | HIMSS. On - Line Journal of Nursing Informatics (OJNI). 2020;24(1). https://www.himss.org/resources/implementation-evidence-based-electronic-health-record-ehr-downtime-readiness-and.

83. Genes N, Chary M, Chason KW. An academic medical center's response to widespread computer failure. Am J Disaster Med. 2013;8(2):145–150. https://pubmed.ncbi.nlm.nih.gov/24352930/. doi: 10.5055/ajdm.2013.0121; PMID: 24352930.

References

84. Poterack KA, Gottlieb O. Are you ready for EHR downtime? questions to ask. ASA Monitor. 2016;80(2):30–31. https://pubs.asahq.org/monitor/article-abstract/81/4/30/5896/Paper-Charting-Anesthetics-Forgotten-But-Not-Gone?redirectedFrom=fulltext.

85. Electronic prescribing of controlled substances (EPCS). https://portal.ct.gov/dcp/drug-control-division/drug-control/epcs-information-page. Accessed Jun 17, 2024.

86. Holmgren AJ, Co Z, Newmark L, Danforth M, Classen D, Bates D. Assessing the safety of electronic health records: A national longitudinal study of medication-related decision support. BMJ Qual Saf. 2020;29(1):52–59. https://pubmed.ncbi.nlm.nih.gov/31320497/. doi: 10.1136/bmjqs-2019-009609; PMID: 31320497.

87. Subbe CP, Tellier G, Barach P. Impact of electronic health records on predefined safety outcomes in patients admitted to hospital: A scoping review. BMJ Open. 2021;11(1):e047446. https://pubmed.ncbi.nlm.nih.gov/33441368/. doi: 10.1136/bmjopen-2020-047446; PMID: 33441368; PMC7812113.

88. The Joint Commission. 2024 hospital national patient safety goals. www.jointcommission.org Web site. https://www.jointcommission.org/-/media/tjc/documents/standards/national-patient-safety-goals/2024/hap-npsg-simple-2024-v2.pdf. Updated 2024. Accessed Jul 25, 2024.

89. Wright A, Ai A, Ash J, et al. Clinical decision support alert malfunctions: Analysis and empirically derived taxonomy. J Am Med Inform Assoc. 2018;25(5):496–506. https://pubmed.ncbi.nlm.nih.gov/29045651/. doi: 10.1093/jamia/ocx106; PMID: 29045651; PMC6019061.

90. Ash JS, Singh H, Wright A, Chase D, Sittig DF. Essential activities for electronic health record safety: A qualitative study. Health Informatics J. 2020;26(4):3140–3151. https://pubmed.ncbi.nlm.nih.gov/30848694/. doi: 10.1177/1460458219833109; PMID: 30848694.

91. Palojoki S, Saranto K, Reponen E, Skants N, Vakkuri A, Vuokko R. Classification of electronic health record-related patient safety incidents: Development and validation study. JMIR Med Inform. 2021;9(8):e30470. https://pubmed.ncbi.nlm.nih.gov/34245558/. doi: 10.2196/30470; PMID: 34245558; PMC8441612.

92. Palojoki S, Vuokko R, Vakkuri A, Saranto K. Electronic health record system-related patient safety incidents - how to classify them? Stud Health Technol Inform. 2020;275:157–161. https://pubmed.ncbi.nlm.nih.gov/33227760/. doi: 10.3233/SHTI200714; PMID: 33227760.

93. Blecker S, Austrian JS, Shine D, Braithwaite RS, Radford MJ, Gourevitch MN. Monitoring the pulse of hospital activity: Electronic health record utilization as a measure of care intensity. J Hosp Med. 2013;8(9):513–518. https://pubmed.ncbi.nlm.nih.gov/23908140/. doi: 10.1002/jhm.2068; PMID: 23908140.

94. Barbieri AL, Fadare O, Fan L, Singh H, Parkash V. Challenges in communication from referring clinicians to pathologists in the electronic health record era. J Pathol Inform. 2018;9:8. https://pubmed.ncbi.nlm.nih.gov/29692945/. doi: 10.4103/jpi.jpi_70_17; PMID: 29692945; PMC5896165.

95. Richmond RT, McFadzean IJ, Vallabhaneni P. Reaching the summit of discharge summaries: A quality improvement project. BMJ Open Quality. 2021;10(1):e001142. https://pubmed.ncbi.nlm.nih.gov/33589507/. doi: 10.1136/bmjoq-2020-001142; PMID: 33589507; PMC7887351.

96. Melnick ER, Fong A, Nath B, et al. Analysis of electronic health record use and clinical productivity and their association with physician turnover. JAMA Netw Open. 2021;4(10):e2128790. https://pubmed.ncbi.nlm.nih.gov/34636911/. doi: 10.1001/jamanetworkopen.2021.28790; PMID: 34636911; PMC8511970.

97. Lopez K, Li H, Paek H, et al. Predicting physician departure with machine learning on EHR use patterns: A longitudinal cohort from a large multi-specialty ambulatory practice - PubMed. PLoS One . 2023;18(2):e0280251. https://pubmed.ncbi.nlm.nih.gov/36724149/. doi: 10.1371/journal.pone.0280251; PMID: 36724149; PMC9891518.

References

98. Hansen MA, Hirth J, Zoorob R, Langabeer J. Demographics and clinical features associated with rates of electronic message utilization in the primary care setting. Int J Med Inform. 2024;183(105339). https://pubmed.ncbi.nlm.nih.gov/38219417/. doi: 10.1016/j.ijmedinf.2024.105339; PMID: 38219417.

99. Heisey-Grove D, Rathert C, McClelland LE, Jackson K, DeShazo J. Classification of patient- and clinician-generated secure messages using a theory-based taxonomy. Health Sci Rep. 2021;4(2):e295. https://pubmed.ncbi.nlm.nih.gov/34084944/. doi: 10.1002/hsr2.295; PMID: 34084944; PMC8142627.

100. Martinez KA, Schulte R, Rothberg MB, Tang MC, Pfoh ER. Patient portal message volume and time spent on the EHR: An observational study of primary care clinicians. J Gen Intern Med. 2024;39(4):566–572. https://pubmed.ncbi.nlm.nih.gov/38129617/. doi: 10.1007/s11606-023-08577-7; PMID: 38129617; PMC10973312.
