# Supplementary material for: Revisions to the Safety Assurance Factors for Electronic Health Record Resilience (SAFER) Guides to update national recommendations for safe use of electronic health records
Source: J Am Med Inform Assoc. 2025 Apr 12;32(4):755–60. doi: 10.1093/jamia/ocaf018 (PMC12005625; doi:10.1093/jamia/ocaf018)
Supplement: ocaf018_Supplementary_Data [file ocaf018_supplementary_data.zip › ocaf018_Supplementary_Data/Organizational Responsibilities Final.pdf]

Self-Assessment

Organizational Responsibilities

General Instructions for the SAFER Self-Assessment Guides

The Safety Assurance Factors for EHR Resilience (SAFER) guides are designed to help healthcare organizations conduct proactive self-assessments to evaluate the safety and effectiveness of their electronic health record (EHR) implementations. The 2025 SAFER guides have been updated and streamlined to focus on the highest risk, most commonly occurring issues that can be addressed through technology or practice changes to build system resilience in the following areas:

- Organizational Responsibilities
- Patient Identification
- Clinician Communication
- Test Results Reporting and Follow-up
- Computerized Provider Order Entry with Decision Support
- Systems Management
- Contingency Planning
- High Priority Practices - A collection of 16 Recommendations from the other 7 Guides

Each of the eight SAFER Guides begins with a Checklist of recommended practices. The downloadable SAFER Guides provide fillable circles that can be used to indicate the extent to which each recommended practice has been implemented in the organization using a 5-point Likert scale. The Practice Worksheet gives a rationale for the practice and provides examples of how to implement each recommended practice. It contains fields to record team member involvement and follow-up actions based on the assessment. The Worksheet also lists the stakeholders who can provide input to assess each practice (sources of input). In addition to the downloadable version, the content of each SAFER Guide, with interactive references and supporting materials, can also be viewed on ONC's website at: https://www.healthit.gov/topic/safety/safer-guides.

The SAFER guides are based on the best available (2024) evidence from the literature and consensus expert opinion. Subject matter experts in patient safety, informatics, quality improvement, risk management, human factors engineering, and usability developed them. Furthermore, they were reviewed by an external group of practicing clinicians, informaticians, and information technology professionals.

Each guide contains between 6 and 18 recommended practices including its rationale, implementation guidance, and evidence level. The recommended practices in the SAFER Guides are intended to be useful for all EHR users. However, every organization faces unique circumstances and may implement a particular recommended practice differently. As a result, some of the specific implementation guidance in the SAFER Guides for recommended practices may not be applicable to an organization.

The High Priority Practices guide consists of 16 of the most important and relevant recommendations selected from the other 7 guides. It is designed for practicing clinicians to help them understand, implement, and support EHR safety and safe use within their organization. The other seven guides consist of 88 unique recommendations that are relevant for all healthcare providers and organizations.

The SAFER Guides are designed in part to help deal with safety concerns created by the continuously changing sociotechnical landscape that healthcare organizations face. Therefore, changes in technology, clinical practice standards, regulations, and policy should be taken into account when using the SAFER Guides. Periodic self-assessments using the SAFER Guides may also help organizations identify areas where it is particularly important to address the implications of these practice or EHR-based changes for the safety and safe use of EHRs. Ultimately, the goal is to improve the overall safety of our health care system and improve patient outcomes.

The SAFER Guides are not intended to be used for legal compliance purposes, and implementation of a recommended practice does not guarantee compliance with the HIPAA Security or Privacy Rules, Medicare or Medicaid Conditions of Participation, or any other laws or regulations. The SAFER Guides are for informational purposes only and are not intended to be an exhaustive or definitive source. They do not constitute legal advice. Users of the SAFER Guides are encouraged to consult with their own legal counsel regarding compliance with Medicare or Medicaid program requirements, and any other laws.

For additional information on Medicare and Medicaid program requirements, please visit the Centers for Medicare & Medicaid Services website at www.cms.gov. For more information on HIPAA, please visit the HHS Office for Civil Rights website at www.hhs.gov/ocr.

Self-Assessment

Organizational Responsibilities

Introduction

The Organizational Responsibilities SAFER Guide identifies individual and organizational responsibilities (activities, processes, and tasks) intended to optimize the safety and safe use of EHRs. A key addition to this revised guide relates to the use of Artificial Intelligence (AI)*-enabled systems, or EHRs with enhanced AI features or functions for the administration, diagnosis, treatment, and management of patient care. Such AI-enabled systems have the potential to revolutionize administrative and clinical decision-making, but there are known and unknown risks associated with their use. During the transition period as more and better AI-enabled systems are being developed and healthcare organizations are adapting their clinical and administrative workflows to account for these new devices and applications, unintended adverse consequences will occur. Therefore, it is incumbent on healthcare organizations, AI developers, and EHR vendors to work together to identify optimal ways for these systems to be used and to share responsibility (based on their ability and resources available) for their safe and effective use.

A safe EHR or AI implementation is critically dependent on the people involved and the support provided to them by the organization. This guide, compared to all of the other SAFER Guides, focuses chiefly on human behavior and relationships. In particular, it includes the structures, processes and outcomes that apply to the people who have responsibility for EHR-related patient safety in healthcare organizations.

Safe EHR and AI implementations require attention to social as well as technical matters. This guide is designed to help safely manage individual and organizational responsibilities in a complex “sociotechnical” healthcare organization. When EHRs and AI systems are implemented, responsibilities can be shifted, forgotten, or newly created. Careful attention to the details of those responsibilities is a critical factor in system safety and in realizing the potential benefits of EHRs.

Completing the self-assessment in the Organizational Responsibilities SAFER Guide requires the engagement of a wide variety of people at all levels within the organization. Because this guide is designed to help organizations prioritize EHR-related safety concerns, clinician leadership in the organization should be engaged in assessing whether and how any particular recommended practice affects the organization’s ability to deliver safe, high-quality care. The collaboration between administrative leaders, technical specialists, clinicians, and staff members in completing the self-assessment in this guide will provide an accurate snapshot of the organization’s EHR implementation status in terms of safety. Most importantly, collaborative efforts should establish a consensus on the organization's future direction to optimize EHR-related safety and quality. This entails setting priorities among the recommended practices not yet implemented, ensuring a plan to maintain recommended practices already in place, dedicating the necessary resources for essential improvements, and working together to mitigate safety risks introduced by the EHR or AI.

The Organizational Responsibilities Guide is laid out in a similar format to the other SAFER Guides with a focus on structures, processes and the evaluation/monitoring of outcomes. Domain 1 includes the various structures required to support safe use of health IT including leadership roles, staff roles, policies and procedures. Domain 2 includes the recommended processes carried out by organizations that are instrumental in the safe use of health IT. Tasks carried out by appropriate personnel are recommended in this section, such as conducting comprehensive EHR training by high-quality trainers, reporting and following up on high-priority EHR-related errors, and involving clinicians in EHR safety-related decision-making. Domain 3 focuses on the evaluation and monitoring of health IT safety outcomes and includes reviewing the recommendations contained in all of the SAFER Guides as well as collaborative monitoring and ongoing assessments with organizational vendor partners.

*Artificial Intelligence or AI has the meaning set forth in 15 U.S.C. 9401(3): a machine-based system that can, for a given set of human-defined objectives, make predictions, recommendations, or decisions influencing real or virtual environments. Artificial intelligence systems use machine- and human-based inputs to perceive real and virtual environments; abstract such perceptions into models through analysis in an automated manner; and use model inference to formulate options for information or action.

Self-Assessment

Organizational Responsibilities

Table of Contents

General Instructions	1
Introduction	2
About the Checklist	5
Checklist	6
Team Worksheet	9
About the Recommended Practice Worksheets	10
Recommended Practice Worksheets	11
References	30

Authors and Peer Reviewers

The SAFER Self-Assessment Guides were developed by health IT safety researchers and informatics experts whose contributions are acknowledged as follows:

Primary authors who contributed to the development of all guides:

Trisha Flanagan, RN, MSN, CPPS, Health Informatics Nurse, Center for Innovations in Quality, Effectiveness and Safety, Michael E. DeBakey Veterans Affairs Medical Center, Houston TX

Hardeep Singh, MD, MPH, Co-Chief, Health Policy, Quality and Informatics Program, Center for Innovations in Quality, Effectiveness and Safety and Professor of Medicine at the Michael E. DeBakey Veterans Affairs Medical Center and Baylor College of Medicine, Houston, TX

Dean F. Sittig MS, PhD, FACMI, FAMIA, FHIMSS, FIAHSI, Professor of Biomedical Informatics, Department of Clinical and Health Sciences, McWilliams School of Biomedical Informatics, University of Texas Health Science Center at Houston, TX and Informatics Review LLC, Lake Oswego, OR

Support staff for the primary authorship team

Rosann Cholankeril, MD, MPH, Center for Innovations in Quality, Effectiveness and Safety, Michael E. DeBakey Veterans Affairs Medical Center and Baylor College of Medicine

Sara Ehsan, MBBS, MPH, Center for Innovations in Quality, Effectiveness and Safety, Michael E. DeBakey Veterans Affairs Medical Center and Baylor College of Medicine

Additional authors who contributed to at least one guide:

Jason S. Adelman, MD, MS, (Patient ID) Chief Patient Safety Officer & Associate Chief Quality Officer, Executive Director, Patient Safety Research, Co-Director, Patient Safety Research Fellowship in Hospital Medicine, New York-Presbyterian Hospital/Columbia University Irving Medical Center, New York, NY

Daniel R. Murphy, MD, MBA, (Clinician Communication, Test Results) Chief Quality Officer, Baylor Medicine, Houston, TX

Patricia Sengstack, DNP, NI-BC, FAAN, FACMI, (Organizational Responsibilities) Senior Associate Dean for Informatics, Director, Nursing Informatics Specialty Program, Vanderbilt University School of Nursing, Vanderbilt University, Nashville, TN

Additional contributors who provided feedback on various guides or parts of guides

Miriam Callahan, MD (Patient ID)
David C. Classen, MD (CPOE, AI recommendation)
Anne Grauer, MD, MS (Patient ID)
Ing Haviland (Patient ID)
Amanda Heidemann, MD (All Guides)
I-Fong Sun Lehman, DrPH, MS (Patient ID)
Christoph U. Lehmann, MD (AI recommendation)
Christopher A. Longhurst, MD, MS (AI recommendation)
Edward R. Melnick, MD (Clinician Communication)
Robert E. Murphy, MD (Organizational Responsibilities)
Ryan P. Radecki, MD, MS (AI recommendation)
Raj Ratwani, PhD (AI recommendation)
Trent Rosenbloom, MD (Clinician Communication)
Lisa Rotenstein, MD (Clinician Communication)
Hojjat Salmasian, MD, PhD (All Guides)
Richard Schreiber, MD (CPOE)
Danny Sands, MD (Clinician Communication)
Debora Simmons, PhD, RN (Organizational Responsibilities)
Carina Sirochinsky (Patient ID)
Neha Thummala, MPH (Patient ID)
Emma Weatherford (Patient ID)
Adam Wright, PhD (CPOE)
Andrew Zimolzak, MD, MMSc (Test Results, Clinician Communication)

This guide was developed under the contract Unintended Consequences of Health IT and Health Information Exchange, Task Order HHSP23337003T/HHSP23320095655WC.

The ATSP/ONC composite mark is a mark of the U.S. Department of Health and Human Services. The contents of the publication or project are solely the responsibility of the authors and do not necessarily represent the official views of the U.S. Department of Health and Human Services, Asssistant Secretary for the Technology Policy/Office of the National Coordinator for Health Information Technology.

>Table of Contents | >About the Checklist | >Team Worksheet | >About the Practice Worksheets | >Practice Worksheets

The *Checklist* is structured as a quick way to enter and print your self-assessment.

Select the level of implementation achieved by your organization for each Recommended Practice. Your Implementation Status will be reflected on the Recommended Practice Worksheet in this PDF. The implementation status scales are as followed:

Not Implemented – (0%)
The organization has not implemented this recommendation.

Making Progress (1 - 30%)
The organization is in the early or pilot phase of implementing this recommendation as evidenced by following or adopting less than 30% of the implementation guidance.

Halfway there (31 – 60%)
The organization is implementing this recommendation and is following or has adopted approximately half of the implementation guidance.

Substantial Progress (61-90%)
The organization has nearly implemented this recommendation and is following or has adopted much of the implementation guidance.

Fully Implemented (91-100%)
The organization follows this recommendation, and most implementation guidance is followed consistently and widely adopted.

The organization should check the following box if there are some limitations with the current version of their EHR that preclude them from fully implementing this recommendation.

EHR Limitation - The EHR does not offer the features/functionality required to fully implement this recommendation or the implementation guidance.

The *Domain* associated with the *Recommended Practice(s)* appears at the top of the column

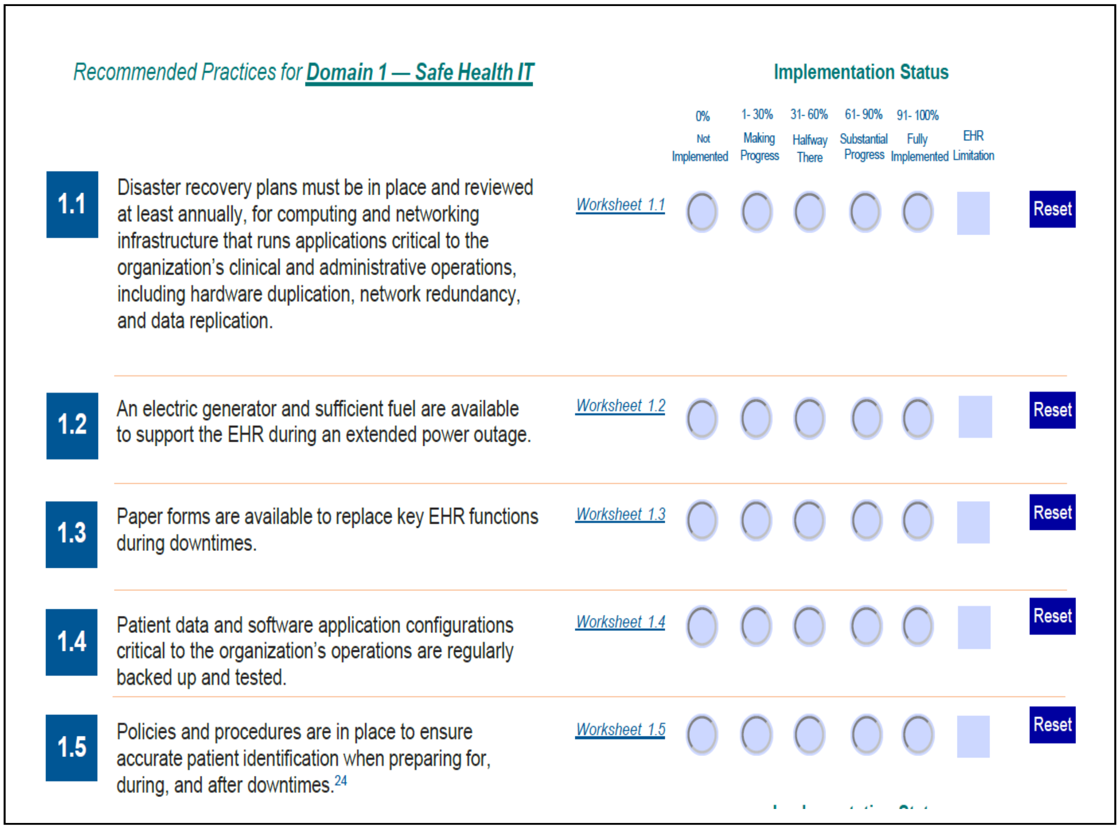
Recommended Practices for Domain 1 — Safe Health IT

Implementation Status

			0% Not Implemented	1-30% Making Progress	31-60% Halfway There	61-90% Substantial Progress	91-100% Fully Implemented	EHR Limitation	
1.1	Disaster recovery plans must be in place and reviewed at least annually, for computing and networking infrastructure that runs applications critical to the organization's clinical and administrative operations, including hardware duplication, network redundancy, and data replication.	Worksheet 1.1							Reset
1.2	An electric generator and sufficient fuel are available to support the EHR during an extended power outage.	Worksheet 1.2							Reset
1.3	Paper forms are available to replace key EHR functions during downtimes.	Worksheet 1.3							Reset
1.4	Patient data and software application configurations critical to the organization's operations are regularly backed up and tested.	Worksheet 1.4							Reset
1.5	Policies and procedures are in place to ensure accurate patient identification when preparing for, during, and after downtimes.[24]	Worksheet 1.5							Reset

The *Recommended Practice(s)* for the topic appears below the associated *Domain*.

To the right of reach *Recommended Practice* is a link to the Recommended Practice Worksheet in this PDF.

The *Worksheet* provides guidance on implementing the practice.

Recommended Practices for **Domain 1 — Structures Required for Safe Health IT**

Implementation Status

#	Recommended Practice	Worksheet	Not Implemented (0%)	Making Progress (1-30%)	Halfway There (31-60%)	Substantial Progress (61-90%)	Fully Implemented (91-100%)	EHR Limitation	
1.1	*Highest level decision makers in the organization (e.g., boards of directors, owners of physician practices, C-suite executives, and clinical leaders) commit to promoting a culture of safety that incorporates the safety and safe use of EHRs.	Worksheet 1.1	○	○	○	○	○		Reset
1.2	Organizations have designated staff to conduct the testing, implementation, maintenance, continuous monitoring, and resolution of problems with EHR hardware, software, and network/ISP components.	Worksheet 1.2	○	○	○	○	○		Reset
1.3	Organizations have established expert-level training teams equipped with technical and clinical skills, and resources to provide education and training on the safe use of EHR to all users and IT staff.	Worksheet 1.3	○	○	○	○	○		Reset
1.4	Organizations have an effective decision-making structure for managing and optimizing the safety and safe use of the EHR that includes user input.	Worksheet 1.4	○	○	○	○	○		Reset
1.5	Organizations have policies, procedures, and tools that facilitate reporting of EHR-related hazards and errors.	Worksheet 1.5	○	○	○	○	○		Reset
1.6	Organizations have policies and procedures for when, and how, clinicians can delegate aspects of EHR use including, for example, order entry, medication reconciliation, and clinical documentation.	Worksheet 1.6	○	○	○	○	○		Reset
1.7	Organizations have procedures and personnel to ensure that EHR users can obtain timely assistance for EHR-related hardware, software, CDS, or network/ISP problems.	Worksheet 1.7	○	○	○	○	○		Reset
1.8	Organizations have a designated person or team focused on the safety and safe use of health information technology.	Worksheet 1.8	○	○	○	○	○		Reset

Recommended Practices for ***Domain 1 — Structures Required for Safe Health IT***

Implementation Status

		Worksheet	Not Implemented (0%)	Making Progress (1-30%)	Halfway There (31-60%)	Substantial Progress (61-90%)	Fully Implemented (91-100%)	EHR Limitation	
1.9	Artificial Intelligence (AI)-enabled application developers, EHR vendors, and healthcare organizations using AI-enabled systems or EHRs with enhanced AI features or functions share responsibility (based on their ability and resources available) for ensuring AI safety. This shared responsibility includes appropriate clinical, technical, and administrative governance, policies, procedures, people, and technologies to ensure AI is monitored and that its use is safe, secure, private, ethical, and equitable.[28]	Worksheet 1.9	◯	◯	◯	◯	◯		Reset

Recommended Practices for ***Domain 2 — Using Health IT Safely - Processes***

Implementation Status

		Worksheet	Not Implemented (0%)	Making Progress (1-30%)	Halfway There (31-60%)	Substantial Progress (61-90%)	Fully Implemented (91-100%)	EHR Limitation	
2.1	Practicing clinicians are involved in EHR safety-related decision-making that impacts clinical use.	Worksheet 2.1	◯	◯	◯	◯	◯		Reset
2.2	Comprehensive EHR training is provided by qualified trainers, and appropriately tailored to specific users and job requirements.	Worksheet 2.2	◯	◯	◯	◯	◯		Reset
2.3	Access to the production version of the EHR is granted only after successful completion of high-quality EHR training and competency assessment.	Worksheet 2.3	◯	◯	◯	◯	◯		Reset
2.4	Workflow analysis maps administrative and clinical work and ensures that the EHR is used safely to deliver care.	Worksheet 2.4	◯	◯	◯	◯	◯		Reset
2.5	Healthcare organizations and EHR vendors share responsibility for identifying and addressing EHR safety concerns.	Worksheet 2.5	◯	◯	◯	◯	◯		Reset

Recommended Practices for Domain 3 — Monitoring/Evaluating Health IT Safety

	Recommended Practice	Worksheet	Not Implemented (0%)	Making Progress (1-30%)	Halfway There (31-60%)	Substantial Progress (61-90%)	Fully Implemented (91-100%)	EHR Limitation	
3.1	Organizations have a strategy and mechanisms for identification, measurement, monitoring, and mitigation of high priority EHR safety risks and hazards.	Worksheet 3.1	○	○	○	○	○		Reset
3.2	Healthcare and EHR vendor organizations conduct, document and disseminate comprehensive multi-stakeholder reviews or safety analyses for high-priority harm events that involve health IT. These vendor safety analyses are discussed by key stakeholders within the healthcare organization.	Worksheet 3.2	○	○	○	○	○		Reset

Clinicians should complete this self-assessment and evaluate potential health IT-related patient safety risks addressed by this specific SAFER Guide within the context of your particular healthcare organization

This Team Worksheet is intended to help organizations document the names and roles of the self-assessment team, as well as individual team members' activities. Typically, team members will be drawn from a number of different areas within your organization, and in some instances, from external sources. The suggested Sources of Input section in each Recommended Practice Worksheet identifies the types of expertise or services to consider engaging. It may be particularly useful to engage specific clinician and other leaders with accountability for safety practices identified in this guide.

The Worksheet includes fillable boxes that allow you to document relevant information. The Assessment Team Leader box allows documentation of the person or persons responsible for ensuring that the self-assessment is completed. The section labeled Assessment Team Members enables you to record the names of individuals, departments, or other organizations that contributed to the self-assessment. The date that the self-assessment is completed can be recorded in the Assessment Completion Date section and can also serve as a reminder for periodic reassessments. The section labeled Assessment Team Notes is intended to be used, as needed, to record important considerations or conclusions arrived at through the assessment process. This section can also be used to track important factors such as pending software updates, vacant key leadership positions, resource needs, and challenges and barriers to completing the self-assessment or implementing the Recommended Practices in this SAFER Guide.

Assessment Team Leader

Assessment Completion Date

Assessment Team Members

Assessment Team Notes

Reset

Each *Recommended Practice Worksheet* provides guidance on implementing a specific *Recommended Practice*, and allows you to enter and print information about your self-assessment.

The *Suggested Sources of Input* section indicates categories of personnel who can provide information to help evaluate your level of implementation.

The *Rationale* section provides guidance about "why" the safety activities are needed.

Strength of Recommendation section provides an estimate of the strength of evidence available in the scientific literature, or states that it is "required" due to a federal rule, regulation, or conditions of participation, for each recommendation.

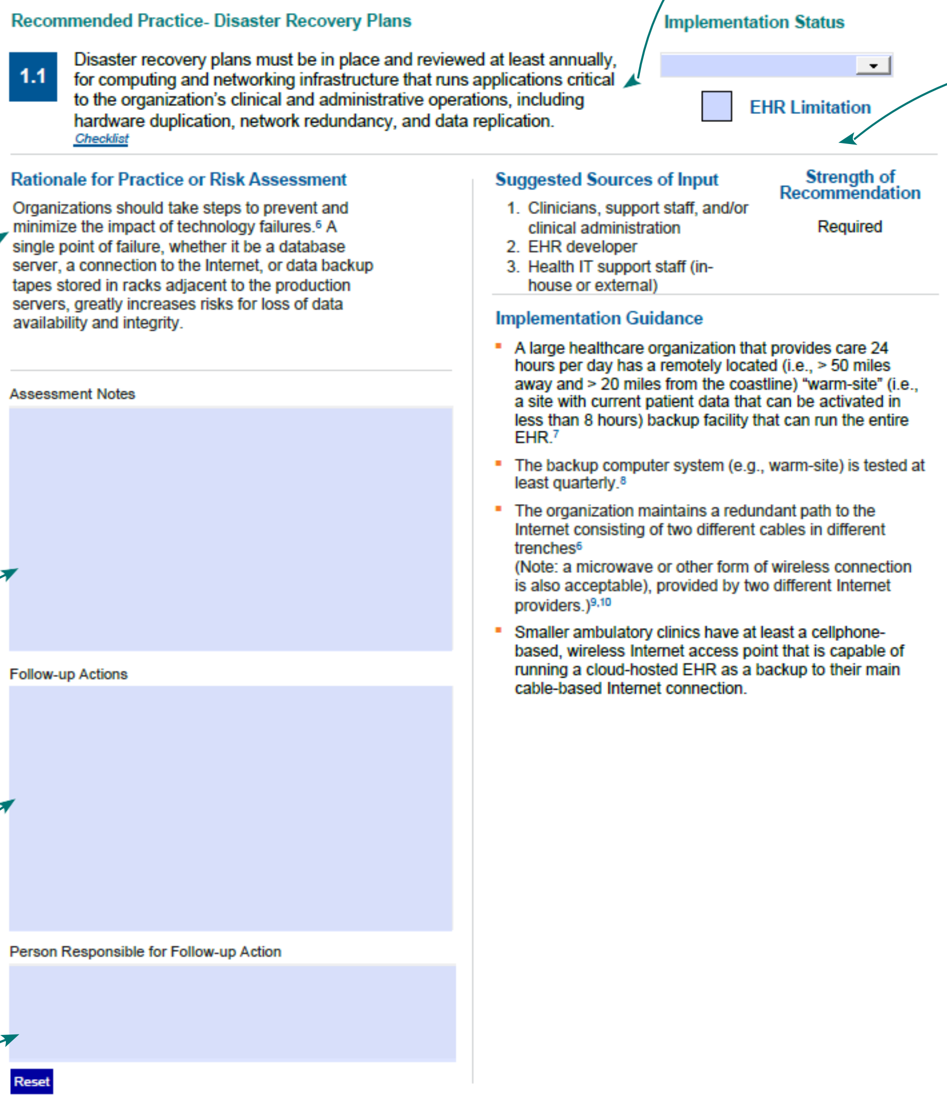

Recommended Practice- Disaster Recovery Plans

Implementation Status

1.1 Disaster recovery plans must be in place and reviewed at least annually, for computing and networking infrastructure that runs applications critical to the organization's clinical and administrative operations, including hardware duplication, network redundancy, and data replication.
Checklist

EHR Limitation

Rationale for Practice or Risk Assessment

Organizations should take steps to prevent and minimize the impact of technology failures.[6] A single point of failure, whether it be a database server, a connection to the Internet, or data backup tapes stored in racks adjacent to the production servers, greatly increases risks for loss of data availability and integrity.

Suggested Sources of Input

1. Clinicians, support staff, and/or clinical administration
2. EHR developer
3. Health IT support staff (in-house or external)

Strength of Recommendation

Required

Implementation Guidance

- A large healthcare organization that provides care 24 hours per day has a remotely located (i.e., > 50 miles away and > 20 miles from the coastline) "warm-site" (i.e., a site with current patient data that can be activated in less than 8 hours) backup facility that can run the entire EHR.[7]
- The backup computer system (e.g., warm-site) is tested at least quarterly.[8]
- The organization maintains a redundant path to the Internet consisting of two different cables in different trenches[6] (Note: a microwave or other form of wireless connection is also acceptable), provided by two different Internet providers.)[9,10]
- Smaller ambulatory clinics have at least a cellphone-based, wireless Internet access point that is capable of running a cloud-hosted EHR as a backup to their main cable-based Internet connection.

Assessment Notes

Follow-up Actions

Person Responsible for Follow-up Action

Reset

Enter any notes about your self-assessment.

Enter any follow-up activities required.

Enter the name of the person responsible for the follow-up activities.

The Implementation Guidance section lists potentially useful practices or scenarios to inform your assessment and implementation of the specific Recommended Practice.

Recommended Practice - Safety Culture

Implementation Status

1.1 Highest-level decision makers in the organization (e.g., boards of directors, owners of physician practices, C-suite executives, and clinical leaders) commit to promoting a culture of safety that incorporates the safety and safe use of EHRs.

Checklist

EHR Limitation

Rationale for Practice or Risk Assessment

A culture of safety promoted by top executives encourages continuous learning, improvement, and engagement from all levels of the organization. By actively and transparently prioritizing safety, organizational leadership can help ensure systems and processes remain effective and responsive to emerging EHR-related threats and challenges. By prioritizing EHR safety, leadership promotes collaboration across all levels, engaging clinical staff, IT professionals, and administrative personnel in a unified approach to addressing safety concerns and implementing effective solutions. Ultimately, this high-level focus on EHR safety ensures strategic investments in reliable and efficient health IT systems, further solidifying the organization's commitment to safety and excellence.

Assessment Notes

Follow-up Actions

Person Responsible for Follow-up Action

Reset

Suggested Sources of Input

1. Large organization: Board of directors, President/Vice President, C-Suite executives, Clinical leaders
2. Small organization: Owners, Clinical leaders, COO

Strength of Recommendation

Medium

Implementation Guidance

- High-level decision makers recognize that EHR safety is integral to patient safety. They ensure that EHR safety is integrated into organizational policies and procedures and risk management practices.[1,2]
- High-level decision makers provide adequate staffing and resources to ensure that safety issues associated with adoption and use of EHRs can be addressed in a timely fashion.[3]
- High-level decision makers review the results of EHR safety assessments, such as those from SAFER Guide use.
- High-level decision makers identify EHR-related patient safety goals (e.g., percentage of abnormal laboratory test results that are acknowledged within a timeframe appropriate for the importance, severity, and healthcare setting or percentage of medications administered following barcode identification), assess whether those goals are being reached, and address any shortcomings.[3]
- High-level decision makers identify and support staff members who can provide systematic feedback to the EHR vendors regarding perceived safety issues with their EHRs.[4]

Recommended Practice - Health IT Resources

Implementation Status

1.2 Organizations have designated staff to conduct the testing, implementation, maintenance, continuous monitoring, and resolution of problems with EHR hardware, software, and network/ISP components.
Checklist

EHR Limitation

Rationale for Practice or Risk Assessment

A culture of learning and improvement is essential to identify and address potential issues before they cause harm. However, the impact of changes to the EHR or interfaced applications can be unpredictable and potentially elusive, making proactive assessment and monitoring vital. Furthermore, customization of the EHR or content requires expertise to avoid introducing new hazards, particularly during upgrades. Inadequate training and preparedness of staff can lead to unaddressed problems, while insufficient maintenance can render the EHR unreliable or unavailable, posing significant safety risks. By conducting a thorough risk assessment, healthcare organizations can mitigate these risks and ensure the safe and effective use of AI in their EHR systems.

Assessment Notes

Follow-up Actions

Reset

Person Responsible for Follow-up Action

Suggested Sources of Input

1. Large organization: Safety officer, IT department, Health IT informatics-type department, Clinicians, support staff, and/or clinical administration
2. Small organization: Office management, health IT staff/contractor, providers, Health IT contractor or internal health IT-oriented person, EHR vendor

Strength of Recommendation

Medium

Implementation Guidance

- The organization has adequate numbers of trained staff members available either on-site or elsewhere to manage software/hardware.[5]
- Information technology department staff members adhere to standard practices of IT lifecycle management from industry experts (i.e., Information Technology Infrastructure Library (ITIL) – ITIL's IT Service Management Framework -ITSM)[5]
- At least some staff members working in hospital information technology are ITIL certified.
- A plan outlining who is responsible for each aspect of EHR safety monitoring (e.g., hardware/software, clinical content, usability, training, communication breakdowns, etc.) is in place.[6,7]
- Providers and others (including leadership in large organizations) are encouraged to use tools (e.g., real-time dashboards or monthly/quarterly reports) to monitor EHR safety and care quality.[7,8]
- Adequate technical staff members are available to fix hardware problems during operating hours.[5]
- Staff members are trained to identify and promptly correct common EHR-related safety concerns, related to registration, order entry, and test results communication, for example.[5]
- Risk assessments for error-prone processes are conducted prior to go-live (e.g., Failure Modes and Effects Analysis of high-risk clinical processes such as blood or chemotherapy ordering and administration).[6,8,9]
- Organization provides resources for regular maintenance of hardware, software, CDS, and the network/ISP.[5]

Recommended Practice - Health IT Resources (continued)

1.2 Organizations have designated staff to conduct the testing, implementation, maintenance, continuous monitoring, and resolution of problems with EHR hardware, software, and network/ISP components.
Checklist

Implementation Guidance (continued)

- When EHR maintenance is contracted to external parties, there is adequate oversight of contractor performance.
- EHR vendors provide recommendations and timelines for routine maintenance procedures to organization's staff members.[10]

Recommended Practice - Training Resources

Implementation Status

1.3 Organizations have established expert-level training teams equipped with technical and clinical skills, and resources to provide education and training on the safe use of EHR to all users and IT staff.

Checklist

EHR Limitation

Rationale for Practice or Risk Assessment

Expert-level training teams, equipped with both technical and clinical expertise, are essential to minimize risks and errors in patient care. These teams can ensure that all users and IT staff are proficient in the safe use of health IT, thereby reducing potential harm. Furthermore, establishing expert training programs enables organizations to comply with regulatory requirements and accreditation standards set by reputable bodies such as The Joint Commission, ISO, DNV, ECRI, and others. As health IT systems evolve through updates or new implementations, expert training teams play a vital role in managing these changes seamlessly, ensuring continuous patient safety and care quality.

Assessment Notes

Follow-up Actions

Person Responsible for Follow-up Action

Reset

Suggested Sources of Input

1. Large organization: Chief Clinical Information Officer (CCIO/CMIO/CNIO), Health IT Specialists, Informatics Specialists, Training and Education Coordinators, Clinicians
2. Small organization: Healthcare Administrator, Informatics-type specialist, Training and Education Coordinator, EHR Vendor Representatives

Strength of Recommendation

Medium

Implementation Guidance

- The organization has adequate numbers of trained staff members available to provide EHR education and training for all system users prior to system use.[7,11]
- EHR training teams are comprised of personnel with both technical and clinical expertise who contribute to a comprehensive training curriculum.[7,12]
- A variety of training modalities are offered to accommodate user schedules and learning styles.[13,14]
- The organization develops and delivers joint training sessions where hospital trainers and vendor representatives provide comprehensive education on EHR use, focusing on both technical and practical aspects.
- In larger organizations, health IT and informatics training staff receive training from the EHR vendor and become certified in the applications, features, or functions learned, as appropriate.
- Online training is complemented with in-person sessions where users can ask questions, participate in hands-on exercises, and receive immediate feedback from trainers.[13,14]
- A training plan outlines how EHR users will maintain and learn new knowledge to use the system safely.[12,13]
- Targeted training programs are developed that address common EHR safety risks and user errors.[15]

Recommended Practice - Structure and Governance

Implementation Status

1.4 Organizations have an effective decision-making structure for managing and optimizing the safety and safe use of the EHR that includes user input.

Checklist

EHR Limitation

Rationale for Practice or Risk Assessment

A well-established decision-making structure is crucial to identify, assess, and mitigate potential risks associated with EHR use, enabling proactive management of safety concerns. Moreover, a structured governance framework ensures compliance with healthcare regulations and standards, while also facilitating an open culture of feedback and continuous improvement. This governance structure should include feedback loops for users to report issues and suggest enhancements, clearly define responsibility and processes, and foster collaboration across disciplines to address critical EHR safety issues. By establishing such a governance framework, healthcare organizations can minimize risks, optimize EHR performance, and prioritize patient safety.

Assessment Notes

Follow-up Actions

Person Responsible for Follow-up Action

Reset

Suggested Sources of Input

1. Large organization: Health IT Leadership (CIO, CCIO/ CMIO/CHIO/CNIO), C-suite executives, Clinicians, Regulatory compliance experts, Informatics-type roles, Health IT team members
2. Small organization: Owners, Clinicians

Strength of Recommendation

Medium

Implementation Guidance

- Meetings and committees related to health IT safety include a mix of clinicians (physicians, nurses, pharmacists), IT specialists, risk management, and administrative staff.[16]
- For EHR changes/enhancements that impact clinicians, a governance process is in place to obtain decision-making input from impacted stakeholders.[17]
- Routine multidisciplinary health IT safety rounds or huddles are conducted that include IT personnel and frontline clinicians.[18]
- An EHR Enhancement/Change Task Force is established and includes clinicians, IT experts, data analysts, and quality improvement staff.[19]
- For emergent situations, the organization designates a few key stakeholders (e.g., CMIO, Head of the Pharmacy, or Chief Nursing Officer) who can approve safety-related EHR changes on short notice.
- For larger organizations, all of the following are represented in decision-making about EHR safety: clinicians, administrators, patients, health IT/informatics, CEO/COOs, Nursing and Pharmacy leadership, quality, regulatory compliance, and legal staff.
- For smaller ambulatory practices and small hospitals, both clinical and administrative staff members are represented in decision-making about EHR safety, with assistance from outside experts as needed.
- EHR safety is appropriately included in job performance appraisals through review of EHR usage reports that record statistics such as percentage of time user logs off or locks their workstation rather than allowing it to time-out or percentage of medication administrations by the RN that were appropriately recorded via barcode, for example.
- For a larger organization, an EHR safety oversight committee is in place, or these functions are designated to an informatics or safety oversight committee

Recommended Practice - Reporting Policies

Implementation Status

1.5 Organizations have policies, procedures, and tools that facilitate reporting of EHR-related hazards and errors.

Checklist

EHR Limitation

Rationale for Practice or Risk Assessment

A culture of safety is founded on the ability of users to report EHR-related hazards without fear of retribution. Reporting EHR-related hazards and errors is vital to identifying and mitigating risks that could compromise patient safety, and policies such as anonymous reporting can increase the frequency and comprehensiveness of reporting. Consistent reporting helps identify systemic EHR issues that may not be apparent from isolated incidents, enabling targeted training programs to enhance clinicians' safe and effective use of the system. Furthermore, analyzing reported errors and hazards can inform organizational improvement efforts, driving meaningful changes that prioritize patient safety and optimize EHR performance. By establishing a strong reporting culture and leveraging reported data, healthcare organizations can proactively address potential risks and ensure the safe integration of AI in their EHR systems.

Assessment Notes

Follow-up Actions

Reset

Person Responsible for Follow-up Action

Suggested Sources of Input

1. Large organization: Safety officer, Quality/Risk officer, all those involved in safety initiatives, informatics-type department responsibility, Clinicians, support staff, and/or clinical administration
2. Small organization: Office Management, clinicians

Strength of Recommendation

Medium

Implementation Guidance

- Policies clearly define what constitutes an EHR-related hazard or error and provide guidance on how and when to report concerns.[20-22]
- Policies explicitly state that reporting EHR-related hazards and errors will not result in punitive action and are communicated effectively to build trust.
- There is regular review of reported concerns with clinicians and feedback is provided to reporters with corrective actions.[22]
- Those who manage EHR and patient safety initiatives for the organization have a clear process for addressing identified problems and for reporting problems externally to the EHR vendor and/or a Patient Safety Organization (PSO) when appropriate.[20,23]
- A user-friendly reporting tool integrated into the EHR allows clinicians to quickly and easily report issues during their workflow, ensuring that valuable insights are captured in real time.[24]

Recommended Practice - Delegation Policies

Implementation Status

1.6 Organizations have policies and procedures for when, and how, clinicians can delegate aspects of EHR use including, for example, order entry, medication reconciliation, and clinical documentation.

Checklist

EHR Limitation

Rationale for Practice or Risk Assessment

Structured delegation policies ensure that EHR tasks are performed by individuals who are trained and competent, which can improve accuracy and reduce the risk of errors. By delegating tasks effectively and ensuring that the right individuals are performing them, healthcare organizations can minimize the potential for errors and optimize EHR performance, ultimately prioritizing patient safety and care quality.

Assessment Notes

Follow-up Actions

Person Responsible for Follow-up Action

Reset

Suggested Sources of Input

1. Large organization: Hospital departments, Clinicians, EHR training and education team members, Quality/ Legal team members
2. Small organization: Clinicians

Strength of Recommendation

Required

Implementation Guidance

- The organization has a written policy and procedure in place regarding the delegation of order entry, medication reconciliation, and clinical documentation tasks.[25,26]
- Delegation policies reflect the appropriate roles and duties as described by regulatory and licensing bodies for clinicians and staff.
- Detailed Standard Operating Procedures (SOPs) are developed for each delegated EHR task to ensure consistency and accuracy.
- SOPs ensure competency (i.e., administrative, clinical, and EHR knowledge) of those with delegated EHR data entry authority and include steps to evaluate delegated work.[26]
- Role-based access controls are implemented within the EHR system to ensure only authorized and trained individuals can perform specific tasks.[26]
- Mentorship and supervision programs support staff in performing delegated EHR tasks accurately.

Recommended Practice - Support Resources

Implementation Status

1.7 Organizations have procedures and personnel to ensure that EHR users can obtain timely assistance for EHR-related hardware, software, CDS, or network/ISP problems.

Checklist

EHR Limitation

Rationale for Practice or Risk Assessment

Timely support is particularly vital for issues involving Clinical Decision Support (CDS) tools, as errors or delays can lead to incorrect treatments or diagnoses, compromising patient safety. Prompt resolution of hardware and network issues also ensures the integrity and security of patient data within the EHR system. A well-structured support system demonstrates an organization's commitment to a collaborative and supportive work environment, where users can access reliable help when needed. In contrast, difficulties in accessing timely support can lead to inefficiencies, workarounds, and potentially harmful consequences.

Assessment Notes

Follow-up Actions

Person Responsible for Follow-up Action

Reset

Suggested Sources of Input

1. Large organization: CEO/COO, Health IT, informatics-type department, Clinicians, support staff, and/or clinical administration
2. Small organization: Office management

Strength of Recommendation

Medium

Implementation Guidance

- A dedicated IT help desk is established with highly capable staff trained to handle EHR-related issues.[5]
- Onsite IT support teams provide immediate assistance for hardware and network issues.[5,24]
- IT support teams are equipped with essential tools and access to address common hardware/software and network issues on the spot.[5,25]
- In small practices, guidelines exist for determining when to seek external help.
- Organizations provide guidance to users on how to get help and to health IT staff members on when and how to get outside assistance.[6,24]
- EHR help desk leadership collects and reports transparently their metrics on response time and abandonment rates of support calls.[5]

Recommended Practice - Health IT Safety Resource

1.8 Organizations have a designated person or team focused on the safety and safe use of health information technology.
Checklist

Implementation Status

EHR Limitation

Rationale for Practice or Risk Assessment

Assigning responsibility for tasks such as facilitating annual reviews of SAFER Guides, reviewing EHR-related incident reports, and developing comprehensive health IT safety plans helps mitigate risks, enhance patient safety, and ensure compliance with best practices and regulatory requirements.

Assessment Notes

Follow-up Actions

Person Responsible for Follow-up Action

Reset

Suggested Sources of Input

1. Large organization: IT and clinical leadership responsible for managing health IT safety efforts (CIO, CCIO/CMIO/CNIO)
2. Small organization: Administrative leadership

Strength of Recommendation

Medium

Implementation Guidance

- An EHR safety officer, or someone assigned that responsibility part-time, plays a key role in assuring safety.[26]
- Larger organizations can assign a full-time Health IT Safety Officer.[26]
- Organizations dedicate resources and personnel to facilitate the annual review of the SAFER Guides.[18,27]
- Organizations assign resources and personnel to review risks and incident reports associated with the use of the EHR (or other healthcare technology).
- Organizations assign a resource who is responsible for the development and implementation of an overall Health IT Safety Plan, including, for example, contingency planning for EHR downtimes and patient privacy breaches.[2]
- Staff members with job responsibilities for EHR safety are encouraged to participate in relevant outside professional activities, including patient safety organizations (PSOs) and communicate with others in similar positions.
- Organizations support professional development of staff assigned responsibility for any aspect of EHR safety by budgeting for and encouraging training.[54]
    - Organizations support membership in informatics related professional organizations.
    - Organizations support attendance at professional organization meetings or conferences
    - Organizations support obtaining informatics related certifications

Recommended Practice - Artificial Intelligence

1.9 Artificial Intelligence (AI)-enabled application developers, EHR vendors, and healthcare organizations using AI-enabled systems or EHRs with enhanced AI features or functions share responsibility (based on their ability and resources available) for ensuring AI safety. This shared responsibility includes appropriate clinical, technical, and administrative governance, policies, procedures, people, and technologies to ensure AI is monitored and that its use is safe, secure, private, ethical, and equitable.[28, 67]

Checklist

Implementation Status

EHR Limitation

Rationale for Practice or Risk Assessment

The integration of AI-enabled systems in healthcare has the potential to revolutionize clinical decision-making, but it also introduces known and unknown risks that must be mitigated.[29] As healthcare organizations adapt their clinical and administrative workflows to new AI-driven technologies, unintended adverse consequences will inevitably occur, particularly during the transition period. Early AI applications have already exhibited unintended biases and "hallucinations," leading to false information that can harm patients. To address these risks, healthcare organizations and AI/EHR developers must collaborate, leveraging their complementary expertise to ensure AI systems are robust, reliable, and transparent. Continuous monitoring and updating are crucial to maintain system integrity, prioritize patient safety, and ensure data security. Conducting a risk assessment of AI is essential to identify and mitigate these risks, build trust among users and stakeholders, and promote safe and effective adoption of AI in healthcare.

Assessment Notes

Follow-up Actions

Person Responsible for Follow-up Action

Reset

Suggested Sources of Input

1. Large organizations: Clinicians, Clinical Administration, Health IT Support Staff, EHR (or AI) developer, AI experts
2. Small organizations: Wait for better evidence

Strength of Recommendation

Medium

Implementation Guidance

- Organizations conduct ongoing real-world testing and monitoring with local data to minimize the risk to patient safety while these new AI-enabled systems mature.
- Healthcare organizations should conduct, or wait for real-world, clinical evaluations published in high-quality medical journals (e.g., NLM's new list of Clinically Useful Journals - https://jmla.mlanet.org/ojs/jmla/article/view/1631) before they start using AI-enabled systems on a routine basis. While peer-reviewed, publication does not ensure safety or effectiveness of any clinical or administrative intervention, it can provide an external, unbiased assessment of the development, testing, implementation, or use of an AI-enabled system, tool, or intervention.
- Healthcare organizations should add additional people with AI expertise such as data scientists, informaticians, machine-learning and AI operational personnel, human factors experts, and clinical expert(s) to their existing multidisciplinary EHR or CDS oversight committee(s). These individuals, as a group, should be capable of understanding and evaluating the performance of AI-enabled systems. These new committee members should meet regularly to review requests for new applications and proactively monitor the performance of AI-enabled applications in use.
- The committee should maintain an inventory of clinically deployed, AI-enabled systems that includes information on deployment date, current version, responsible personnel, last reviewed date, authorized users, authorized purpose, source of data used to generate, or train, the AI system, and external source(s) of validation, verification, and performance comparison.

Recommended Practice - Artificial Intelligence (cont'd)

1.9 Artificial Intelligence (AI)-enabled application developers, EHR vendors, and healthcare organizations using AI-enabled systems or EHRs with enhanced AI features or functions share responsibility (based on their ability and resources available) for ensuring AI safety. This shared responsibility includes appropriate clinical, technical, and administrative governance, policies, procedures, people, and technologies to ensure AI is monitored and that its use is safe, secure, private, ethical, and equitable.[28]

Checklist

Implementation Guidance (continued)

- Before organizations use AI-enabled systems for patient care (e.g., respond to patient messages, generate differential diagnoses, treatment plans, or notes describing the findings from visits), they must have policies and procedures to ensure that patients and clinicians are aware, when possible, that AI-enabled systems are being used for clinical and/or administrative decision making.[30]
- Organizations should ensure that patients understand when and where AI-enabled systems were developed, how they may be used, and the role of clinicians in reviewing the AI system's output before giving their consent.[31]
- AI-generated recommendations should be reviewed and approved by humans who take responsibility for the recommendation(s) before they are sent to patients.
- Organizations should maintain and regularly review a transaction log of AI system use (i.e., similar to the audit log of the EHR) that includes the AI version in use, date/time of AI system use, patient ID, responsible clinical user ID, input data used by the AI system, AI recommendation or output.
- Organizations have an internal process to evaluate AI-enabled system performance on local data before routine clinical use and periodically following implementation to check for drift,[32] bias,[33] or decay,[34] for example.[35] This process should include ongoing regular testing of AI applications in the (live) production system to ensure the safe performance and safe use of these program's references.[36]
- Organizations have high-quality training programs for clinicians interested in using AI systems that focus on the known and potential risks of using these systems.
- Organizations have a formal consent-style process, complete with signatures, to ensure clinicians understand the risks and benefits of using AI tools before their access is enabled.
- Organizations must provide clear written instructions and authority to enable anyone in the organization's information technology department to disable, stop, or turn off the artificial intelligence-enabled systems, 24 hours a day, seven days a week, in the event of a problem.[37]
- Similar to an organization's preparation for an EHR downtime, organizations must have an established policy and procedure to manage clinical and administrative processes that have become dependent on AI automation, when the AI is not available.
- Organizations should have a clear process for reporting AI-related safety issues and a process for analyzing these issues and mitigating risks.[38]

[*] Artificial Intelligence or AI has the meaning set forth in 15 U.S.C. 9401(3): a machine-based system that can, for a given set of human-defined objectives, make predictions, recommendations, or decisions influencing real or virtual environments. Artificial intelligence systems use machine- and human-based inputs to perceive real and virtual environments; abstract such perceptions into models through analysis in an automated manner; and use model inference to formulate options for information or action.

Recommended Practice - Clinician Decision-Making

Implementation Status

2.1 Practicing clinicians are involved in EHR safety-related decision-making that impacts clinical use.
Checklist

EHR Limitation

Rationale for Practice or Risk Assessment

Frontline clinicians bring practical insights into how EHR systems impact daily workflows and patient care, ensuring decision-making is grounded in real-world clinical practice. Their involvement allows for early identification and proactive resolution of issues, preventing them from escalating into larger problems. Clinicians' nuanced understanding of clinical workflows ensures EHR systems support, rather than hinder, these processes. Moreover, collaboration between clinicians, IT professionals, and administrators fosters better communication, mutual understanding, and effective problem-solving, leading to informed decision-making about clinically relevant issues and ultimately, safer and more effective AI integration in EHR systems.

Assessment Notes

Follow-up Actions

Person Responsible for Follow-up Action

Reset

Suggested Sources of Input

1. Large organization: Administration, Clinicians, Informatics team members
2. Small organization: Clinicians

Strength of Recommendation

Medium

Implementation Guidance

- Clinicians (including physicians, nurses, pharmacists, and others) are included on the EHR safety oversight committee of a large organization.[15,17]
- Clinicians are involved in decision-making about proposed changes to the EHR that affect clinical care (e.g., changes to screen design, content of order sets, charting templates, clinical alerts, role-based access to system resources and placement of workstations).[15,17]
- A formal user feedback program exists where clinicians can report EHR issues, suggest improvements, and share experiences.[15,17]
- Before rolling out major EHR updates or new modules, pilot testing is conducted with a group of clinicians who use the system daily.
- Identify and train clinical champions, or super users, within each department who can act as liaisons between the clinical staff and the IT department.
- Schedule regular meetings that include representatives from various clinical departments to discuss EHR-related issues, share best practices, and collaborate on solutions

Recommended Practice - Conduct EHR Training

Implementation Status

2.2 Comprehensive EHR training is provided by qualified trainers, and appropriately tailored to specific users and job requirements.

Checklist

EHR Limitation

Rationale for Practice or Risk Assessment

Comprehensive EHR training is a critical to equip users with the necessary skills and knowledge to effectively utilize the EHR system. It also increases proficiency, reduces errors, and ultimately enhances patient safety. Tailored training programs that cater to diverse skill levels and job roles ensure that all users receive appropriate instruction, minimizing the risk of suboptimal training, which can lead to patient harm, wasted time, and user frustration. By investing in robust training, healthcare organizations can optimize EHR performance, mitigate potential risks, and ensure the safe integration of AI in their systems.

Assessment Notes

Follow-up Actions

Person Responsible for Follow-up Action

Reset

Suggested Sources of Input

1. Large organization: Patient safety officer, compliance officer, chief information security officer, Informatics-type department, health IT, EHR vendor, Clinical, administrative and IT leadership team
2. Small organization: Owners, Office management, developer

Strength of Recommendation

Required[12]

Implementation Guidance

- Different modalities for training are offered to accommodate user schedules and learning styles.[7,13]
- Training is designed so that it is tailored to specific job roles, such as physicians, nurses, administrative staff, and billing personnel.[7,16]
- Interactive simulations that mimic real-life scenarios pertinent to the user's clinical practice are used for training.[13,39]
- Practice labs are established where users can work with a test version of the EHR system.
- Comprehensive training manuals and resources are provided that include step-by-step guides, troubleshooting tips, and FAQs.
- Training materials are reviewed and updated in parallel with major upgrades, ensuring that training materials accurately reflect the system currently in use. When minor updates or system configuration changes are applied, supplemental training materials are created to augment existing training materials until they are formally updated.
- A mechanism is established for collecting user feedback on the training program that informs ongoing improvement. Feedback about training is reviewed and addressed.
- User skills are monitored and upgraded when needed.[13,39]
- Various topics related to EHR safety, including how to function during EHR downtimes, are covered in EHR training.

Recommended Practice - Access to EHR

Implementation Status

2.3 Access to the production version of the EHR is granted only after successful completion of high-quality EHR training and competency assessment.

Checklist

EHR Limitation

Rationale for Practice or Risk Assessment

Requiring successful completion of high-quality EHR training and competency assessment before granting access to the production version of the EHR ensures that all users are proficient in navigating and using the system effectively. This prerequisite minimizes the risk of errors, enhances data accuracy, and ensures that patient information is handled securely and efficiently. By mandating high-quality training, healthcare organizations can promote best practices, maintain high standards of care, and comply with regulatory requirements, ultimately improving patient outcomes and overall operational efficiency. Requiring successful completion of high-quality EHR training and competency assessment before granting access to the production version of the EHR, healthcare organizations can ensure that all users are proficient in navigating and using the system effectively. This prerequisite minimizes the risk of errors, enhances data accuracy, and ensures secure and efficient handling of patient information. Moreover, mandating high-quality training promotes best practices, maintains high standards of care, and ensures compliance with regulatory requirements, ultimately leading to improved patient outcomes and enhanced operational efficiency.

Assessment Notes

Follow-up Actions

Person Responsible for Follow-up Action

Reset

Suggested Sources of Input

Healthcare Administration, Clinician

Strength of Recommendation

Medium

Implementation Guidance

- User attendance and training completion are carefully monitored prior to EHR use.[12,39,40]
- Users pass a test (e.g., perform actions using the EHR) that covers key aspects of their job(s) (e.g., physicians enter an order or RNs document a medication administration) to demonstrate their understanding and proficiency in using the EHR system.[41]
- Users are provided protected time to attend required training.[39]

> Table of Contents | > About the Checklist | > Team Worksheet | > About the Practice Worksheets | >Practice Worksheets

Recommended Practice - Map Workflows

Implementation Status

2.4 Workflow analysis maps administrative and clinical work and ensures that the EHR is used safely to deliver care.
Checklist

EHR Limitation

Rationale for Practice or Risk Assessment

By mapping out existing administrative and clinical processes, organizations gain a clear understanding of current task performance, enabling identification of inefficiencies and bottlenecks that hinder productivity and increase errors. This analysis allows for streamlining operations, improving efficiency, and ensuring the EHR system supports safe clinical practices and regulatory compliance. If workflow considerations are neglected, it can lead to wasted resources, end-user frustration, and potentially harmful workarounds that compromise patient safety. By prioritizing workflow analysis, healthcare organizations can optimize EHR integration, mitigate risks, and promote high-quality patient care.

Assessment Notes

Follow-up Actions

Person Responsible for Follow-up Action

Reset

Suggested Sources of Input

1. Large organization: Informatics-type department, health IT, EHR vendor, Clinicians
2. Small organization: Office management, EHR vendor, Clinicians

Strength of Recommendation

Medium

Implementation Guidance

- Workflow analysis is conducted prior to implementation of the EHR.[42-44]
- System configuration changes are informed by flowcharts that detail each step of a clinical process that has the potential to impact patient care and how clinicians interact with the EHR.[45]
- When considering making significant configuration changes, simulations are used to create scenarios on how staff will interact with the EHR system.
- Multidisciplinary teams consisting of physicians, nurses, administrative staff, IT personnel, and EHR trainers review and analyze workflows.[43,46]
- Based on the workflow analysis, an effective change management approach (e.g., strategies for promoting the adoption and effective use of EHRs) guides any needed workflow changes.

Recommended Practice - Involve EHR Vendor

2.5 Healthcare organizations and EHR vendors share responsibility for identifying and addressing EHR safety concerns.
Checklist

Implementation Status

EHR Limitation

Rationale for Practice or Risk Assessment

By working together, healthcare organizations and EHR vendors leverage their respective expertise to prioritize and share responsibility for patient safety. Healthcare organizations bring firsthand knowledge of clinical workflows and real-world EHR applications, while vendors contribute technical expertise and understanding of the system's architecture. Through continuous collaboration, they can ensure EHR systems evolve to meet emerging needs, address new safety concerns, and optimize patient care. This joint approach fosters a culture of shared accountability, driving ongoing improvement and mitigating risks associated with EHRs and AI integration.

Assessment Notes

Follow-up Actions

Person Responsible for Follow-up Action

Reset

Suggested Sources of Input

1. Large organization: Board of directors, EHR vendors, Clinical and IT leadership team
2. Small organization: Owners, EHR developers

Strength of Recommendation

Medium

Implementation Guidance

- Organizations should have a documented process for monitoring information provided by the EHR vendor with regard to existing defects. When defects that previously required workarounds are resolved, users receive appropriate training.
- EHR vendors create their own set of system-specific guidance to help their clients configure their EHRs to meet the SAFER Guide recommendations.[47]
- Healthcare organizations and EHR vendors review the SAFER Guide recommendations annually.[47,48]
- EHR vendors are provided feedback from clinicians on potential safety enhancements to the system.[47,48]

Recommended Practice - Evaluation Strategy

Implementation Status

3.1 Organizations have a strategy and mechanisms for identification, measurement, monitoring, and mitigation of high priority EHR safety risks and hazards.
Checklist

EHR Limitation

Rationale for Practice or Risk Assessment

A robust strategy enables organizations to proactively manage potential hazards, mitigating the most significant threats to patient safety by focusing on high-priority EHR safety risks. This approach is not only crucial for patient safety but also often mandated by regulatory bodies. By identifying high-priority risks, organizations can allocate resources effectively, targeting efforts on critical areas and informing targeted training programs to enhance clinicians' safe and effective use of the EHR system. This proactive risk management enables healthcare organizations to minimize adverse events, optimize EHR performance, and ensure compliance with regulatory requirements.

Assessment Notes

Follow-up Actions

Person Responsible for Follow-up Action

Reset

Suggested Sources of Input

1. Large organization: Board of directors, Clinical, Informatics, and IT leadership team, Safety officer
2. Small organization: Owners, EHR vendors, Clinicians

Strength of Recommendation

Medium

Implementation Guidance

- A plan exists for learning from incidents to improve EHR safety.[55-57]
- Real-time monitoring tools are deployed that track system performance, detect anomalies, and alert IT staff to potential issues.[58]
- Organization EHR representatives meet regularly with the EHR vendor to discuss new or ongoing issues.
- Bidirectional communication between the organization and the EHR vendor ensures timely updates, patches, and support for the system.
- A multi-stakeholder committee or task force convenes on a regular basis to review all high-priority EHR-related hazards.[18,20]
- EHR-related incidents are categorized and summarized by location (i.e., clinical and within the EHR), severity, and type to assess for any trends that need to be addressed.[19]
- The mechanism for anonymous, no-fault, internal reporting of EHR-related safety hazards is clear to all users.[18]
- Organization has a policy and procedure that addresses timeliness of addressing reported errors, including an escalation process to organization leadership when the established service level is not being met or is at risk of not being met.
- Larger organizations use specialized "help desk" software to manage internal EHR error reports and their disposition.
- The user who reported the issue, if identified, should be notified of the outcome when appropriate

Recommended Practice - In-Depth Evaluation

3.2 Healthcare and EHR vendor organizations conduct, document and disseminate comprehensive multi- stakeholder reviews or safety analyses for high-priority harm events that involve health IT. These vendor safety analyses are discussed by key stakeholders within the healthcare organization.

Checklist

Implementation Status

EHR Limitation

Rationale for Practice or Risk Assessment

Evaluating the vendor's approach to safety issues ensures prompt and effective problem-solving, minimizes risks to patient health and enhances care quality. Regular reviews of mitigation strategies hold vendors accountable, promote transparency, and build trust. Utilizing safety analysis techniques like root cause analysis (RCA) identifies underlying causes of safety hazards, leading to effective interventions that improve patient safety and prevent future incidents. Documenting and disseminating findings foster a culture of accountability and transparency, while involving multiple stakeholders brings diverse perspectives and expertise, ensuring comprehensive problem-solving and robust safety solutions. This collaborative approach ensures that safety issues are addressed thoroughly, and all stakeholders are informed, ultimately prioritizing patient safety and care quality.

Assessment Notes

Follow-up Actions

Person Responsible for Follow-up Action

Reset

Suggested Sources of Input

1. Large organization: Safety officer, those involved in safety initiatives, informatics-type department, Clinicians, support staff, and/or clinical administration, EHR Vendor
2. Small organization: Office Management, providers, EHR vendor

Strength of Recommendation

Medium

Implementation Guidance

- EHR vendors should develop their own set of system-specific guidance to help their clients configure their EHRs to meet the SAFER Guide recommendations.[27]
- Healthcare organizations should review the SAFER Guide recommendations annually in collaboration with the EHR vendor.[27]
- Effective communication protocols are established and maintained between the EHR vendor and key organizational stakeholders to ensure the timely and continuous reporting of any safety issues.[47,64]

Recommended Practice - In-Depth Evaluation (continued)

3.2

Healthcare and EHR vendor organizations conduct, document and disseminate comprehensive multi- stakeholder reviews or safety analyses for high-priority harm events that involve health IT. These vendor safety analyses are discussed by key stakeholders within the healthcare organization.

Checklist

Implementation Guidance (continued)

- RCAs are conducted for all high-risk, high-priority health IT safety incidents to identify underlying causes and develop preventive strategies.[15,55,62]
- A dedicated committee is formed that includes key stakeholders from various departments (e.g., IT, clinical staff, patient safety officers) to regularly review and address high-priority health IT safety issues.[15,62]

References

1. Azyabi A, Karwowski W, Davahli MR. Assessing patient safety culture in hospital settings. Int J Environ Res Public Health. 2021;18(5):2466. https://pubmed.ncbi.nlm.nih.gov/33802265/. doi: 10.3390/ijerph18052466; PMID: 33802265; PMC7967599.

2. Fennelly O, Cunningham C, Grogan L, et al. Successfully implementing a national electronic health record: A rapid umbrella review. Int J Med Inform. 2020;144:104281. https://pubmed.ncbi.nlm.nih.gov/33017724/. doi: 10.1016/j.ijmedinf.2020.104281; PMID: 33017724; PMC7510429.

3. Campione JR, Mardon RE, McDonald KM. Patient safety culture, health information technology implementation, and medical office problems that could lead to diagnostic error. J Patient Saf. 2019;15(4):267-273. https://pubmed.ncbi.nlm.nih.gov/30138158/. doi: 10.1097/PTS.0000000000000531; PMID: 30138158.

4. Jianxun C, Arkorful VE, Shuliang Z. Electronic health records adoption: Do institutional pressures and organizational culture matter? Technology in Society. 2021;65:101531. https://www.sciencedirect.com/science/article/pii/S0160791X21000063. doi: 10.1016/j.techsoc.2021.101531.

5. IT Governance USA. The IT infrastructure library for IT service management (ITSM). https://www.itgovernanceusa.com/itil. Updated 2011. Accessed Jul 24, 2024.

6. Aguirre RR, Suarez O, Fuentes M, Sanchez-Gonzalez MA. Electronic health record implementation: A review of resources and tools. Cureus. 2019;11(9):e5649. https://pubmed.ncbi.nlm.nih.gov/31700751/. doi: 10.7759/cureus.5649; PMID: 31700751; PMC6822893.

7. Umstead CN, Unertl KM, Lorenzi NM, Novak LL. Enabling adoption and use of new health information technology during implementation: Roles and strategies for internal and external support personnel. J Am Med Inform Assoc. 2021;28(7):1543-1547. https://pubmed.ncbi.nlm.nih.gov/33893511/. doi: 10.1093/jamia/ocab044; PMID: 33893511; PMC8279782.

8. Office of the National Coordinator for Health Information Technology (ONC), Department of Health and Human Services (HHS). Health data, technology, and interoperability: Certification program updates, algorithm transparency, and information sharing; correction. The Federal Register / FIND. 2024;89(27):8546.

9. MEASURE Evaluation UoNC. Assessment tool for electronic health record security: Guidance for low-resource settings. www.measureevaluation.org Web site. https://www.measureevaluation.org/resources/publications/ms-20-195.html. Updated 2020. Accessed Jul 24, 2024.

10. Habli I, Jia Y, White S, et al. Development and piloting of a software tool to facilitate proactive hazard and risk analysis of health information technology. Health Informatics J. 2020;26(1):683-702. https://pubmed.ncbi.nlm.nih.gov/31165661/. doi: 10.1177/1460458219852789; PMID: 31165661.

11. Tsai CH, Eghdam A, Davoody N, Wright G, Flowerday S, Koch S. Effects of electronic health record implementation and barriers to adoption and use: A scoping review and qualitative analysis of the content. Life (Basel). 2020;10(12):327. https://pubmed.ncbi.nlm.nih.gov/33291615/. doi: 10.3390/life10120327; PMID: 33291615; PMC7761950.

12. Standards MOI 6, JCI accreditation standards for hospitals and academic medical centers (AMC) 8th edition. Joint Commission International. 2023. https://www.jointcommissioninternational.org/-/media/jci/jci-documents/news-and-support/news/jci-accreditation-standards-for-hospitals/management-of-information-moi-and-health-care-technology-hct.pdf#page=9.08.

13. Ting J, Garnett A, Donelle L. Nursing education and training on electronic health record systems: An integrative review. Nurse Educ Pract. 2021;55:103168. https://pubmed.ncbi.nlm.nih.gov/34411879/. doi: 10.1016/j.nepr.2021.103168; PMID: 34411879.

14. Samadbeik M, Fatehi F, Braunstein M, et al. Education and training on electronic medical records (EMRs) for health care professionals and students: A scoping review. Int J Med Inform. 2020;142:104238. https://pubmed.ncbi.nlm.nih.gov/32828034/. doi: 10.1016/j.ijmedinf.2020.104238; PMID: 32828034.

15. Sittig DF, Wright A, Coiera E, et al. Current challenges in health information technology-related patient safety. Health Informatics J. 2020;26(1):181-189. https://pubmed.ncbi.nlm.nih.gov/30537881/. doi: 10.1177/1460458218814893; PMID: 30537881; PMC7510167.

16. Shah T, Kitts AB, Gold JA, et al. Electronic health record optimization and clinician well-being: A potential roadmap toward action. NAM Perspect. 2020;2020. https://pubmed.ncbi.nlm.nih.gov/35291737/. doi: 10.31478/202008a; PMID: 35291737; PMC8916811.

References

17. Ravi A, Arvisais-Anhalt S, Weia B, Khanna R, Adler-Milstein J, Auerbach A. Governance of electronic health record modification at U.S. academic medical centers. Appl Clin Inform. 2023;14(5):843-854. https://pubmed.ncbi.nlm.nih.gov/37553071/. doi: 10.1055/a-2150-8523; PMID: 37553071; PMC10599807.

18. Ash JS, Singh H, Wright A, Chase D, Sittig DF. Essential activities for electronic health record safety: A qualitative study. Health Informatics J. 2020;26(4):3140-3151. https://pubmed.ncbi.nlm.nih.gov/30848694/. doi: 10.1177/1460458219833109; PMID: 30848694.

19. Palojoki S, Vuokko R, Vakkuri A, Saranto K. Electronic health record system-related patient safety incidents - how to classify them? Stud Health Technol Inform. 2020;275:157-161. https://pubmed.ncbi.nlm.nih.gov/33227760/. doi: 10.3233/SHTI200714; PMID: 33227760.

20. Palojoki S, Saranto K, Reponen E, Skants N, Vakkuri A, Vuokko R. Classification of electronic health record-related patient safety incidents: Development and validation study. JMIR Med Inform. 2021;9(8):e30470. https://pubmed.ncbi.nlm.nih.gov/34245558/. doi: 10.2196/30470; PMID: 34245558; PMC8441612.

21. Singh H, Sittig DF. A sociotechnical framework for safety-related electronic health record research reporting: The SAFER reporting framework. Ann Intern Med. 2020;172(11 Suppl):S92-S100. https://pubmed.ncbi.nlm.nih.gov/32479184/. doi: 10.7326/M19-0879; PMID: 32479184.

22. Schreiber R, McGreevey JD. Chapter 20 - governance and implementation. In: Greenes RA, Del Fiol G, eds. Clinical decision support and beyond (third edition). Oxford: Academic Press; 2023:561-601. https://www.sciencedirect.com/science/article/pii/B9780323912006000097. 10.1016/B978-0-323-91200-6.00009-7.

23. Ash JS, Corby S, Mohan V, et al. Safe use of the EHR by medical scribes: A qualitative study. J Am Med Inform Assoc. 2021;28(2):294-302. https://pubmed.ncbi.nlm.nih.gov/33120424/. doi: 10.1093/jamia/ocaa199; PMID: 33120424; PMC7883983.

24. Kiepek W, Sengstack PP. An evaluation of system end-user support during implementation of an electronic health record using the model for improvement framework. Appl Clin Inform. 2019;10(5):964-971. https://pubmed.ncbi.nlm.nih.gov/31853937/. doi: 10.1055/s-0039-3402450; PMID: 31853937; PMC6920050.

25. Sequeira L, Almilaji K, Strudwick G, Jankowicz D, Tajirian T. EHR "SWAT" teams: A physician engagement initiative to improve electronic health record (EHR) experiences and mitigate possible causes of EHR-related burnout. JAMIA Open. 2021;4(2):ooab018. https://pubmed.ncbi.nlm.nih.gov/33898934/. doi: 10.1093/jamiaopen/ooab018; PMID: 33898934; PMC8054031.

26. NYU Langone Health Tech Hub. Health IT safety officer: Navigating the promise and the peril | medium. www.medium.com Web site. https://medium.com/nyu-langones-health-tech-hub/health-it-safety-officer-navigating-the-promise-and-the-peril-73107ed65727. Updated 2019. Accessed Jul 24, 2024.

27. Sittig DF, Sengstack P, Singh H. Guidelines for US hospitals and clinicians on assessment of electronic health record safety using SAFER guides. JAMA. 2022;327(8):719-720. https://pubmed.ncbi.nlm.nih.gov/35129591/. doi: 10.1001/jama.2022.0085; PMID: 35129591.

28. Office of the National Coordinator for Health Information Technology, (ONC). Health sector AI commitments. www.healthit.gov Web site. https://www.healthit.gov/sites/default/files/2023-12/Health_Sector_AI_Commitments_FINAL_120923.pdf. Updated 2023. Accessed Jul 24, 2024.

29. The White House. Executive order on the safe, secure, and trustworthy development and use of artificial intelligence. www.whitehouse.gov Web site. https://www.whitehouse.gov/briefing-room/presidential-actions/2023/10/30/executive-order-on-the-safe-secure-and-trustworthy-development-and-use-of-artificial-intelligence/. Updated 2023. Accessed Jul 24, 2024.

30. Daneshvar N, Pandita D, Erickson S, Snyder Sulmasy L, DeCamp M. Artificial intelligence in the provision of health care: An american college of physicians policy position paper. Ann Intern Med. 2024;177(7):964-967. https://pubmed.ncbi.nlm.nih.gov/38830215/. doi: 10.7326/M24-0146; PMID: 38830215.

31. American Medical Association. Principles for augmented intelligence development, deployment, and use. www.ama-assn.org Web site. https://www.ama-assn.org/system/files/ama-ai-principles.pdf. Updated 2023. Accessed Jul 24, 2024.

32. Feng J, Phillips RV, Malenica I, et al. Clinical artificial intelligence quality improvement: Towards continual monitoring and updating of AI algorithms in healthcare. NPJ Digit Med. 2022;5(1):66. https://pubmed.ncbi.nlm.nih.gov/35641814/. doi: 10.1038/s41746-022-00611-y; PMID: 35641814; PMC9156743.

References

33. Chin MH, Afsar-Manesh N, Bierman AS, et al. Guiding principles to address the impact of algorithm bias on racial and ethnic disparities in health and health care. JAMA Netw Open. 2023;6(12):e2345050. https://pubmed.ncbi.nlm.nih.gov/38100101/. doi: 10.1001/jamanetworkopen.2023.45050; PMID: 38100101; PMC11181958.

34. Vela D, Sharp A, Zhang R, Nguyen T, Hoang A, Pianykh OS. Temporal quality degradation in AI models. Sci Rep. 2022;12(1):11654. https://pubmed.ncbi.nlm.nih.gov/35803963/. doi: 10.1038/s41598-022-15245-z; PMID: 35803963; PMC9270447.

35. Handley JL, Lehmann CU, Ratwani RM. Safe and equitable pediatric clinical use of AI. JAMA Pediatr. 2024;178(7):637-638. https://pubmed.ncbi.nlm.nih.gov/38739385/. doi: 10.1001/jamapediatrics.2024.0897; PMID: 38739385.

36. Ratwani RM, Bates DW, Classen DC. Patient safety and artificial intelligence in clinical care. JAMA Health Forum. 2024;5(2):e235514. https://pubmed.ncbi.nlm.nih.gov/38393719/. doi: 10.1001/jamahealthforum.2023.5514; PMID: 38393719.

37. Anthropic's responsible scaling policy, version 1.0. https://www-cdn.anthropic.com/1adf000c8f675958c2ee23805d91aaade1cd4613/responsible-scaling-policy.pdf. Updated 2023. Accessed Jul 25, 2024.

38. Petersen C, Smith J, Freimuth RR, et al. Recommendations for the safe, effective use of adaptive CDS in the US healthcare system: An AMIA position paper. J Am Med Inform Assoc. 2021;28(4):677-684. https://pubmed.ncbi.nlm.nih.gov/33447854/. doi: 10.1093/jamia/ocaa319; PMID: 33447854; PMC7973467.

39. Heponiemi T, Gluschkoff K, Vehko T, et al. Electronic health record implementations and insufficient training endanger nurses' well-being: Cross-sectional survey study. J Med Internet Res. 2021;23(12):e27096. https://pubmed.ncbi.nlm.nih.gov/34941546/. doi: 10.2196/27096; PMID: 34941546; PMC8738988.

40. Verma G, Ivanov A, Benn F, et al. Analyses of electronic health records utilization in a large community hospital. PLoS One. 2020;15(7):e0233004. https://pubmed.ncbi.nlm.nih.gov/32609757/. doi: 10.1371/journal.pone.0233004; PMID: 32609757; PMC7329072.

41. Bryan A Wilbanks, Edwin N Aroke. Using clinical simulations to train healthcare professionals to use electronic health records: A literature review. Comput Inform Nurs. 2020;38(11):551-561. https://pubmed.ncbi.nlm.nih.gov/32520783/. doi: 10.1097/CIN.0000000000000631; PMID: 32520783

42. Moy AJ, Hobensack M, Marshall K, et al. Understanding the perceived role of electronic health records and workflow fragmentation on clinician documentation burden in emergency departments. J Am Med Inform Assoc. 2023;30(5):797-808. https://pubmed.ncbi.nlm.nih.gov/36905604/. doi: 10.1093/jamia/ocad038; PMID: 36905604; PMC10114050.

43. Moy AJ, Aaron L, Cato KD, et al. Characterizing multitasking and workflow fragmentation in electronic health records among emergency department clinicians: Using time-motion data to understand documentation burden. Appl Clin Inform. 2021;12(5):1002-1013. https://pubmed.ncbi.nlm.nih.gov/34706395/. doi: 10.1055/s-0041-1736625; PMID: 34706395; PMC8550823.

44. Nguyen OT, Jenkins NJ, Khanna N, et al. A systematic review of contributing factors of and solutions to electronic health record-related impacts on physician well-being. J Am Med Inform Assoc. 2021;28(5):974-984. https://pubmed.ncbi.nlm.nih.gov/33517382/. doi: 10.1093/jamia/ocaa339; PMID: 33517382; PMC8068432.

45. Zheng K, Ratwani RM, Adler-Milstein J. Studying workflow and workarounds in electronic health record-supported work to improve health system performance. Ann Intern Med. 2020;172(11 Suppl):S116-S122. https://pubmed.ncbi.nlm.nih.gov/32479181/. doi: 10.7326/M19-0871; PMID: 32479181; PMC8061456.

46. Kataria S, Ravindran V. Electronic health records: A critical appraisal of strengths and limitations. J R Coll Physicians Edinb. 2020;50(3):262-268. https://pubmed.ncbi.nlm.nih.gov/32936099/. doi: 10.4997/JRCPE.2020.309; PMID: 32936099.

47. Sittig DF, Ash JS, Wright A, et al. How can we partner with electronic health record vendors on the complex journey to safer health care? J Healthc Risk Manag. 2020;40(2):34-43. https://pubmed.ncbi.nlm.nih.gov/32648286/. doi: 10.1002/jhrm.21434; PMID: 32648286.

48. Hettinger AZ, Melnick ER, Ratwani RM. Advancing electronic health record vendor usability maturity: Progress and next steps. J Am Med Inform Assoc. 2021;28(5):1029-1031. https://pubmed.ncbi.nlm.nih.gov/33517394/. doi: 10.1093/jamia/ocaa329; PMID: 33517394; PMC8068416.

49. The american medical informatics association (AMIA). www.amia.org Web site. https://amia.org/. Accessed Jul 22, 2024.

50. The american nursing informatics association (ANIA) . www.ania.org Web site. https://ania.org/. Accessed Jul 22, 2024.

References

51. The healthcare information and management systems society (HIMSS). www.himss.org Web site. https://www.himss.org/. Accessed Jul 22, 2024.

52. College of healthcare information management executives (CHIME) . www.chimecentral.org Web site. https://chimecentral.org/#gsc.tab=0. Accessed Jul 22, 2024.

53. The american health information management association (AHIMA) . www.ahima.org Web site. https://www.ahima.org/. Accessed Jul 22, 2024.

54. Klinedinst J(Ed.). The handbook of continuing professional development for the health informatics professional. 2nd ed. New York: Productivity Press.; 2021. https://www.taylorfrancis.com/books/edit/10.4324/9780429398377/handbook-continuing-professional-development-health-informatics-professional-joann-klinedinst. https://doi.org/10.4324/9780429398377.

55. Holmgren AJ, Co Z, Newmark L, Danforth M, Classen D, Bates D. Assessing the safety of electronic health records: A national longitudinal study of medication-related decision support. BMJ Qual Saf. 2020;29(1):52-59. https://pubmed.ncbi.nlm.nih.gov/31320497/. doi: 10.1136/bmjqs-2019-009609; PMID: 31320497.

56. Subbe CP, Tellier G, Barach P. Impact of electronic health records on predefined safety outcomes in patients admitted to hospital: A scoping review. BMJ Open. 2021;11(1):e047446. https://pubmed.ncbi.nlm.nih.gov/33441368/. doi: 10.1136/bmjopen-2020-047446; PMID: 33441368; PMC7812113.

57. The Joint Commission. 2024 hospital national patient safety goals. www.jointcommission.org Web site. https://www.jointcommission.org/-/media/tjc/documents/standards/national-patient-safety-goals/2024/hap-npsg-simple-2024-v2.pdf. Updated 2024. Accessed Jul 25, 2024.

58. Wright A, Ai A, Ash J, et al. Clinical decision support alert malfunctions: Analysis and empirically derived taxonomy. J Am Med Inform Assoc. 2018;25(5):496-506. https://pubmed.ncbi.nlm.nih.gov/29045651/. doi: 10.1093/jamia/ocx106; PMID: 29045651; PMC6019061.

59. Salahuddin L, Ismail Z, Abdul Rahim F, Anawar S, Hashim UR. Development and validation of SafeHIT: An instrument to assess the self-reported safe use of health information technology. Appl Clin Inform. 2023;14(4):693-704. https://pubmed.ncbi.nlm.nih.gov/37648223/. doi: 10.1055/s-0043-1771394; PMID: 37648223; PMC10468731.

60. Sengstack P. Implementing the safety assurance factors for EHR resilience (SAFER) guides. Comput Inform Nurs. 2022;40(11):740-742. https://pubmed.ncbi.nlm.nih.gov/36394468/. doi: 10.1097/CIN.0000000000000988; PMID: 36394468.

61. Geneva: World Health Organization. Patient safety incident reporting and learning systems: Technical report and guidance. www.iris.who.int Web site. https://iris.who.int/bitstream/handle/10665/334323/9789240010338-eng.pdf. Updated 2020. Accessed Jun 24, 2024.

62. VHA National Center for Patient Safety (NCPS). Guide to performing a root cause analysis. https://www.patientsafety.va.gov/docs/RCA-Guidebook_02052021.pdf. Updated 2021. Accessed Jul 22, 2024.

63. Institute for Healthcare Improvement. RCA2: Improving root cause analyses and actions to prevent harm. www.ihi.org Web site. https://www.ihi.org/resources/tools/rca2-improving-root-cause-analyses-and-actions-prevent-harm. Accessed Jul 22, 2024.

64. Sittig DF, Singh H. Policies to promote shared responsibility for safer electronic health records. JAMA. 2021;326(15):1477-1478. https://pubmed.ncbi.nlm.nih.gov/34505867/. doi: 10.1001/jama.2021.13945; PMID: 34505867.

65. Menon S, Singh H, Giardina TD, Rayburn WL, Davis BP, Russo EM, Sittig DF. Safety huddles to proactively identify and address electronic health record safety. J Am Med Inform Assoc. 2017 Mar 1;24(2):261-267. doi: 10.1093/jamia/ocw153. PMID: 28031286; PMCID: PMC5391729.

66. Rubins D, McCoy AB, Dutta S, McEvoy DS, Patterson L, Miller A, Jackson JG, Zuccotti G, Wright A. Real-Time User Feedback to Support Clinical Decision Support System Improvement. Appl Clin Inform. 2022 Oct;13(5):1024-1032. doi: 10.1055/s-0042-1757923. Epub 2022 Oct 26. PMID: 36288748; PMCID: PMC9605820.

References

67. Sittig DF, Singh H. Recommendations to Ensure Safety of AI in Real-World Clinical Care. JAMA. 2024 Nov 27. doi: 10.1001/jama.2024.24598. Epub ahead of print. PMID: 39602298.
