# Supplementary material for: Revisions to the Safety Assurance Factors for Electronic Health Record Resilience (SAFER) Guides to update national recommendations for safe use of electronic health records
Source: J Am Med Inform Assoc. 2025 Apr 12;32(4):755–60. doi: 10.1093/jamia/ocaf018 (PMC12005625; doi:10.1093/jamia/ocaf018)
Supplement: ocaf018_Supplementary_Data [file ocaf018_supplementary_data.zip › ocaf018_Supplementary_Data/Patient Identification Final.pdf]

Self-Assessment

Patient Identification

General Instructions for the SAFER Self-Assessment Guides

The Safety Assurance Factors for EHR Resilience (SAFER) guides are designed to help healthcare organizations conduct proactive self-assessments to evaluate the safety and effectiveness of their electronic health record (EHR) implementations. The 2025 SAFER guides have been updated and streamlined to focus on the highest risk, most commonly occurring issues that can be addressed through technology or practice changes to build system resilience in the following areas:

- Organizational Responsibilities
- Patient Identification
- Clinician Communication
- Test Results Reporting and Follow-up
- Computerized Provider Order Entry with Decision Support
- Systems Management
- Contingency Planning
- High Priority Practices - A collection of 16 Recommendations from the other 7 Guides

Each of the eight SAFER Guides begins with a Checklist of recommended practices. The downloadable SAFER Guides provide fillable circles that can be used to indicate the extent to which each recommended practice has been implemented in the organization using a 5-point Likert scale. The Practice Worksheet gives a rationale for the practice and provides examples of how to implement each recommended practice. It contains fields to record team member involvement and follow-up actions based on the assessment. The Worksheet also lists the stakeholders who can provide input to assess each practice (sources of input). In addition to the downloadable version, the content of each SAFER Guide, with interactive references and supporting materials, can also be viewed on ONC's website at: https://www.healthit.gov/topic/safety/safer-guides.

The SAFER guides are based on the best available (2024) evidence from the literature and consensus expert opinion. Subject matter experts in patient safety, informatics, quality improvement, risk management, human factors engineering, and usability developed them. Furthermore, they were reviewed by an external group of practicing clinicians, informaticians, and information technology professionals.

Each guide contains between 6 and 18 recommended practices including its rationale, implementation guidance, and evidence level. The recommended practices in the SAFER Guides are intended to be useful for all EHR users. However, every organization faces unique circumstances and may implement a particular recommended practice differently. As a result, some of the specific implementation guidance in the SAFER Guides for recommended practices may not be applicable to an organization.

The High Priority Practices guide consists of 16 of the most important and relevant recommendations selected from the other 7 guides. It is designed for practicing clinicians to help them understand, implement, and support EHR safety and safe use within their organization. The other seven guides consist of 88 unique recommendations that are relevant for all healthcare providers and organizations.

The SAFER Guides are designed in part to help deal with safety concerns created by the continuously changing sociotechnical landscape that healthcare organizations face. Therefore, changes in technology, clinical practice standards, regulations, and policy should be taken into account when using the SAFER Guides. Periodic self-assessments using the SAFER Guides may also help organizations identify areas where it is particularly important to address the implications of these practice or EHR-based changes for the safety and safe use of EHRs. Ultimately, the goal is to improve the overall safety of our health care system and improve patient outcomes.

The SAFER Guides are not intended to be used for legal compliance purposes, and implementation of a recommended practice does not guarantee compliance with the HIPAA Security or Privacy Rules, Medicare or Medicaid Conditions of Participation, or any other laws or regulations. The SAFER Guides are for informational purposes only and are not intended to be an exhaustive or definitive source. They do not constitute legal advice. Users of the SAFER Guides are encouraged to consult with their own legal counsel regarding compliance with Medicare or Medicaid program requirements, and any other laws.

For additional information on Medicare and Medicaid program requirements, please visit the Centers for Medicare & Medicaid Services website at www.cms.gov. For more information on HIPAA, please visit the HHS Office for Civil Rights website at www.hhs.gov/ocr.

Self-Assessment

Patient Identification

Introduction

The Patient Identification SAFER Guide identifies recommended safety practices associated with the reliable identification of patients in the EHR. Accurate patient identification ensures that the information displayed and entered into the EHR is associated with the correct person. Processes related to patient identification are complex and require careful planning and attention to avoid errors. In the EHR-enabled healthcare environment, providers rely on technology to help support and manage these complex identification processes. Technology configurations alone cannot ensure accurate patient identification.[1] Staff also must be supported with adequate training and reliable procedures.

This Patient Identification self-assessment can help identify and evaluate where breakdowns related to patient identification occur in the healthcare setting. It focuses on processes within organizations related to the creation of new patient records, patient registration, retrieval of information on previously registered patients, and other types of patient identification activities. The updated recommended practices can help prevent or detect and mitigate problems caused by duplicate records, patient mix-ups, and commingled (or "overlay") records.[2-11]

This guide is meant to support and enable patient matching technology and capabilities, focusing on best practices for improving data accuracy, which is the first step to ensuring accurate patient matching. Although patient matching between organizations is not the focus of this guide, examples herein demonstrate their potential value and typical scenarios in which they are used.

The recommended practices in this Patient Identification SAFER Guide provide support for many, varied patient matching technologies, as well as alternatives and best practices on specific patient attributes for patient matching, which are likely to change over time. New evidence on the importance of incorporating appropriate interventions such as the display of patient photographs, barcoding, and palm scanning are discussed. Other research herein highlights emerging issues related to EHR systems, internal workflow processes, and their potential interactions and impacts.

Completing the self-assessment in the Patient Identification SAFER Guide requires the engagement of people both within and outside the organization (e.g., EHR technology vendors). Because this guide is designed to help organizations prioritize EHR-related safety concerns, clinician leadership in the organization should be engaged in assessing whether and how any particular recommended practice affects the organization's ability to deliver safe, high-quality care. Collaboration between clinicians and staff members while completing the self-assessment in this guide will enable an accurate snapshot of the organization's patient identification status (in terms of safety), and even more importantly, should lead to a consensus about the organization's future path to optimize EHR-related safety and quality: setting priorities among the recommended practices not yet addressed, ensuring a plan is in place to maintain recommended practices already in place, dedicating the required resources to make necessary improvements, and working together to prevent and mitigate the highest priority patient identification-related safety risks introduced by the EHR.

Self-Assessment

Patient Identification

Table of Contents

General Instructions	1
Introduction	2
About the Checklist	5
Checklist	6
Team Worksheet	8
About the Recommended Practice Worksheets	9
Worksheets	10
References	24

Authors and Peer Reviewers

The SAFER Self-Assessment Guides were developed by health IT safety researchers and informatics experts whose contributions are acknowledged as follows:

Primary authors who contributed to the development of all guides:

Trisha Flanagan, RN, MSN, CPPS, Health Informatics Nurse, Center for Innovations in Quality, Effectiveness and Safety, Michael E. DeBakey Veterans Affairs Medical Center, Houston TX

Hardeep Singh, MD, MPH, Co-Chief, Health Policy, Quality and Informatics Program, Center for Innovations in Quality, Effectiveness and Safety and Professor of Medicine at the Michael E. DeBakey Veterans Affairs Medical Center and Baylor College of Medicine, Houston, TX

Dean F. Sittig MS, PhD, FACMI, FAMIA, FHIMSS, FIAHSI, Professor of Biomedical Informatics, Department of Clinical and Health Sciences, McWilliams School of Biomedical Informatics, University of Texas Health Science Center at Houston, TX and Informatics Review LLC, Lake Oswego, OR

Support staff for the primary authorship team

Rosann Cholankeril, MD, MPH, Center for Innovations in Quality, Effectiveness and Safety, Michael E. DeBakey Veterans Affairs Medical Center and Baylor College of Medicine

Sara Ehsan, MBBS, MPH, Center for Innovations in Quality, Effectiveness and Safety, Michael E. DeBakey Veterans Affairs Medical Center and Baylor College of Medicine

Additional authors who contributed to at least one guide:

Jason S. Adelman, MD, MS, (Patient ID) Chief Patient Safety Officer & Associate Chief Quality Officer, Executive Director, Patient Safety Research, Co-Director, Patient Safety Research Fellowship in Hospital Medicine, New York-Presbyterian Hospital/Columbia University Irving Medical Center, New York, NY

Daniel R. Murphy, MD, MBA, (Clinician Communication, Test Results) Chief Quality Officer, Baylor Medicine, Houston, TX

Patricia Sengstack, DNP, NI-BC, FAAN, FACMI, (Organizational Responsibilities) Senior Associate Dean for Informatics, Director, Nursing Informatics Specialty Program, Vanderbilt University School of Nursing, Vanderbilt University, Nashville, TN

Additional contributors who provided feedback on various guides or parts of guides

Miriam Callahan, MD (Patient ID)
David C. Classen, MD (CPOE, AI recommendation)
Anne Grauer, MD, MS (Patient ID)
Ing Haviland (Patient ID)
Amanda Heidemann, MD (All Guides)
I-Fong Sun Lehman, DrPH, MS (Patient ID)
Christoph U. Lehmann, MD (AI recommendation)
Christopher A. Longhurst, MD, MS (AI recommendation)
Edward R. Melnick, MD (Clinician Communication)
Robert E. Murphy, MD (Organizational Responsibilities)
Ryan P. Radecki, MD, MS (AI recommendation)
Raj Ratwani, PhD (AI recommendation)
Trent Rosenbloom, MD (Clinician Communication)
Lisa Rotenstein, MD (Clinician Communication)
Hojjat Salmasian, MD, PhD (All Guides)
Richard Schreiber, MD (CPOE)
Danny Sands, MD (Clinician Communication)
Debora Simmons, PhD, RN (Organizational Responsibilities)
Carina Sirochinsky (Patient ID)
Neha Thummala, MPH (Patient ID)
Emma Weatherford (Patient ID)
Adam Wright, PhD (CPOE)
Andrew Zimolzak, MD, MMSc (Test Results, Clinician Communication)

This guide was developed under the contract Unintended Consequences of Health IT and Health Information Exchange, Task Order HHSP23337003T/HHSP23320095655WC.

The ATSP/ONC composite mark is a mark of the U.S. Department of Health and Human Services. The contents of the publication or project are solely the responsibility of the authors and do not necessarily represent the official views of the U.S. Department of Health and Human Services, Asssistant Secretary for the Technology Policy/Office of the National Coordinator for Health Information Technology.

The *Checklist* is structured as a quick way to enter and print your self-assessment.

Select the level of implementation achieved by your organization for each Recommended Practice. Your Implementation Status will be reflected on the Recommended Practice Worksheet in this PDF. The implementation status scales are as followed:

Not Implemented – (0%)
The organization has not implemented this recommendation.

Making Progress (1 - 30%)
The organization is in the early or pilot phase of implementing this recommendation as evidenced by following or adopting less than 30% of the implementation guidance.

Halfway there (31 – 60%)
The organization is implementing this recommendation and is following or has adopted approximately half of the implementation guidance.

Substantial Progress (61-90%)
The organization has nearly implemented this recommendation and is following or has adopted much of the implementation guidance.

Fully Implemented (91-100%)
The organization follows this recommendation, and most implementation guidance is followed consistently and widely adopted.

The organization should check the following box if there are some limitations with the current version of their EHR that preclude them from fully implementing this recommendation.

EHR Limitation - The EHR does not offer the features/functionality required to fully implement this recommendation or the implementation guidance.

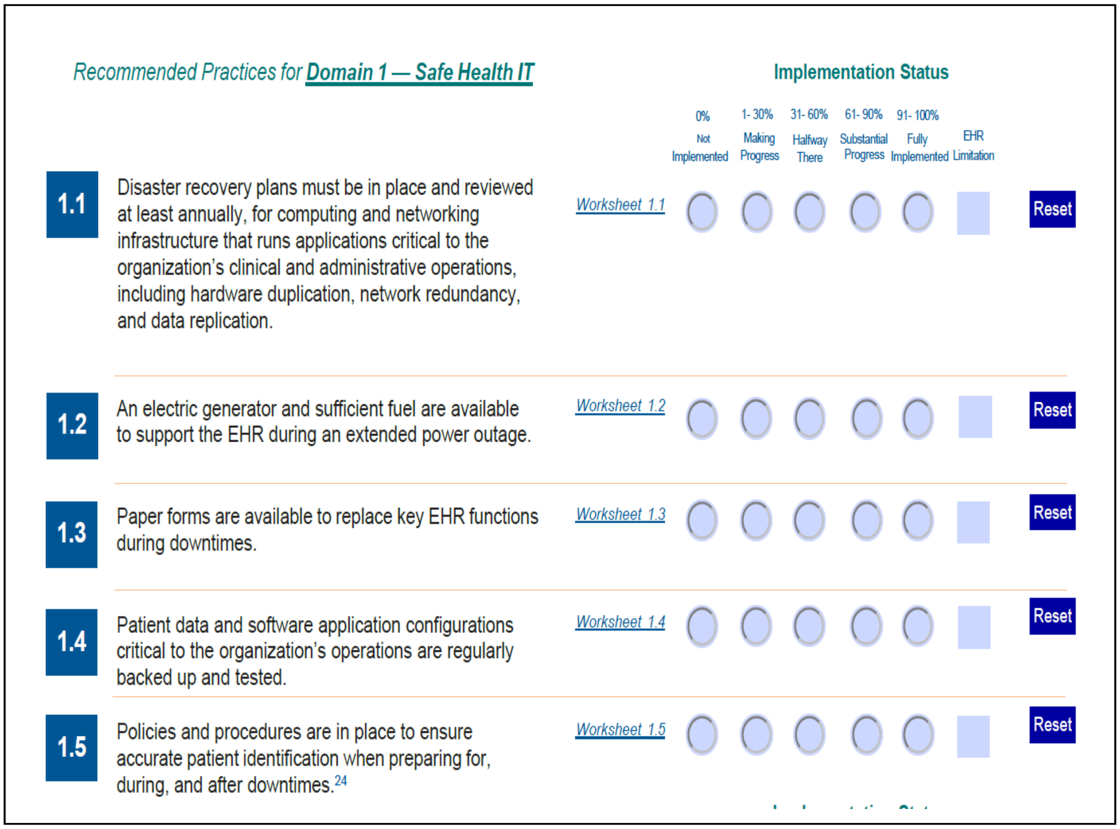

The *Domain* associated with the *Recommended Practice(s)* appears at the top of the column

The *Recommended Practice(s)* for the topic appears below the associated *Domain*.

To the right of reach *Recommended Practice* is a link to the Recommended Practice Worksheet in this PDF.

The *Worksheet* provides guidance on implementing the practice.

Recommended Practices for Domain 1 — Safe Health IT

	Practice	Worksheet	Implementation Status: 0% Not Implemented	1-30% Making Progress	31-60% Halfway There	61-90% Substantial Progress	91-100% Fully Implemented	EHR Limitation	
1.1	An enterprise-wide master patient index (EMPI) is used to identify patients before importing data. The EMPI includes patients' demographic information and medical record number (MRN) (or multiple numbers if used by different parts of the same organization, along with the primary number/key).[12]	Worksheet 1.1	○	○	○	○	○		Reset
1.2	To facilitate correct patient identification, clinicians can create personalized electronic lists of their patients according to several criteria (e.g., user, location, time, service),[21] and patient names on adjacent lines of the EHR are displayed in a visually distinct manner.[2,21,22]	Worksheet 1.2	○	○	○	○	○		Reset
1.3	Information required to accurately identify the patient is clearly displayed on all portions of the EHR user interface.[2]	Worksheet 1.3	○	○	○	○	○		Reset
1.4	Materials printed from the EHR such as wristbands, labels, and reports include multiple patient identifiers and an electronic means of verifying patients' identity (e.g., a 1- or 2-dimensional barcode/QR code).	Worksheet 1.4	○	○	○	○	○		Reset
1.5	Medical record numbers incorporate a check digit to help prevent data entry errors.	Worksheet 1.5	○	○	○	○	○		Reset
1.6	Users are warned when they attempt to create a record for a new patient whose first and last names are the same as another patient, or when a patient search result returns multiple patients with the same or similar names.[2]	Worksheet 1.6	○	○	○	○	○		Reset

Recommended Practices for Domain 2 — Using Health IT Safely

	Practice	Worksheet	Implementation Status: 0% Not Implemented	1-30% Making Progress	31-60% Halfway There	61-90% Substantial Progress	91-100% Fully Implemented	EHR Limitation	
2.1	Patients are registered in a centralized, common database using standardized procedures.[17,46]	Worksheet 2.1	○	○	○	○	○		Reset
2.2	The organization has a process to assign temporary, unique patient IDs (which are later merged into permanent IDs) for when the patient registration system is unavailable, or when patients cannot be registered under their legal names.[34,56,57]	Worksheet 2.2	○	○	○	○	○		Reset

Recommended Practices for Domain 2 — Using Health IT Safely

	Practice	Worksheet	Implementation Status: 0% Not Implemented	1-30% Making Progress	31-60% Halfway There	61-90% Substantial Progress	91-100% Fully Implemented	EHR Limitation	
2.3	The organization uses electronic patient identification such as barcode scanning or radio-frequency identification of patients' wristbands to confirm patients' identity at key points of patient care.[67-69]	Worksheet 2.3	◯	◯	◯	◯	◯		Reset
2.4	The organization uses biometrics to verify patient identity at registration and prior to providing certain types of care.	Worksheet 2.4	◯	◯	◯	◯	◯		Reset
2.5	Patient photographs are collected during patient registration and displayed in multiple places in the EHR to improve patient identification.[83]	Worksheet 2.5	◯	◯	◯	◯	◯		Reset
2.6	Patients who have died are accurately and clearly identified as deceased.	Worksheet 2.6	◯	◯	◯	◯	◯		Reset

Recommended Practices for Domain 3 — Monitoring Safety

	Practice	Worksheet	Implementation Status: 0% Not Implemented	1-30% Making Progress	31-60% Halfway There	61-90% Substantial Progress	91-100% Fully Implemented	EHR Limitation	
3.1	The organization monitors for patient identification errors.[12,92]	Worksheet 3.1	◯	◯	◯	◯	◯		Reset
3.2	The organization monitors and rapidly remediates errors that stem from the failure to create, access, and maintain one unique medical record for each patient (i.e., duplicates, overlays, and overlaps).[6,7]	Worksheet 3.2	◯	◯	◯	◯	◯		Reset

> Table of Contents | > About the Checklist | > Team Worksheet | > About the Practice Worksheets | >Practice Worksheets

Clinicians should complete this self-assessment and evaluate potential health IT-related patient safety risks addressed by this specific SAFER Guide within the context of your particular healthcare organization.

This Team Worksheet is intended to help organizations document the names and roles of the self-assessment team, as well as individual team members' activities. Typically team members will be drawn from a number of different areas within your organization, and in some instances, from external sources. The suggested Sources of Input section in each Recommended Practice Worksheet identifies the types of expertise or services to consider engaging. It may be particularly useful to engage specific clinician and other leaders with accountability for safety practices identified in this guide.

The Worksheet includes fillable boxes that allow you to document relevant information. The Assessment Team Leader box allows documentation of the person or persons responsible for ensuring that the self-assessment is completed. The section labeled Assessment Team Members enables you to record the names of individuals, departments, or other organizations that contributed to the self-assessment. The date that the self-assessment is completed can be recorded in the Assessment Completion Date section and can also serve as a reminder for periodic reassessments. The section labeled Assessment Team Notes is intended to be used, as needed, to record important considerations or conclusions arrived at through the assessment process. This section can also be used to track important factors such as pending software updates, vacant key leadership positions, resource needs, and challenges and barriers to completing the self-assessment or implementing the Recommended Practices in this SAFER Guide.

Assessment Team Leader

Assessment Completion Date

Assessment Team Members

Assessment Team Notes

Reset

Each *Recommended Practice Worksheet* provides guidance on implementing a specific *Recommended Practice*, and allows you to enter and print information about your self-assessment.

The *Suggested Sources of Input* section indicates categories of personnel who can provide information to help evaluate your level of implementation.

Strength of Recommendation section provides an estimate of the strength of evidence available in the scientific literature, or states that it is "required" due to a federal rule, regulation, or conditions of participation, for each recommendation.

The *Rationale* section provides guidance about "why" the safety activities are needed.

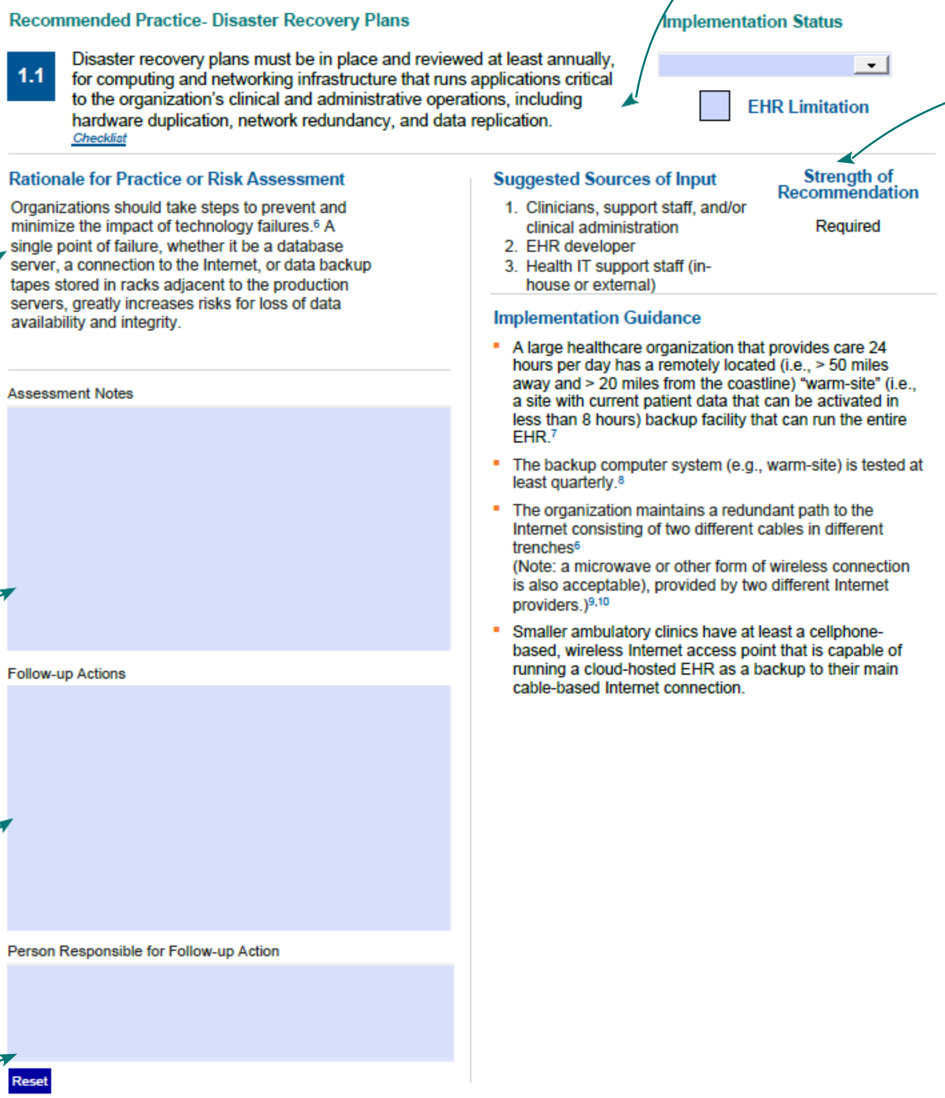

Recommended Practice- Disaster Recovery Plans

Implementation Status

1.1 Disaster recovery plans must be in place and reviewed at least annually, for computing and networking infrastructure that runs applications critical to the organization's clinical and administrative operations, including hardware duplication, network redundancy, and data replication.
Checklist

EHR Limitation

Rationale for Practice or Risk Assessment

Organizations should take steps to prevent and minimize the impact of technology failures.[6] A single point of failure, whether it be a database server, a connection to the Internet, or data backup tapes stored in racks adjacent to the production servers, greatly increases risks for loss of data availability and integrity.

Suggested Sources of Input

1. Clinicians, support staff, and/or clinical administration
2. EHR developer
3. Health IT support staff (in-house or external)

Strength of Recommendation

Required

Implementation Guidance

- A large healthcare organization that provides care 24 hours per day has a remotely located (i.e., > 50 miles away and > 20 miles from the coastline) "warm-site" (i.e., a site with current patient data that can be activated in less than 8 hours) backup facility that can run the entire EHR.[7]
- The backup computer system (e.g., warm-site) is tested at least quarterly.[8]
- The organization maintains a redundant path to the Internet consisting of two different cables in different trenches[6] (Note: a microwave or other form of wireless connection is also acceptable), provided by two different Internet providers.)[9,10]
- Smaller ambulatory clinics have at least a cellphone-based, wireless Internet access point that is capable of running a cloud-hosted EHR as a backup to their main cable-based Internet connection.

Assessment Notes

Follow-up Actions

Person Responsible for Follow-up Action

Reset

Enter any notes about your self-assessment.

Enter any follow-up activities required.

Enter the name of the person responsible for the follow-up activities.

The *Implementation Guidance* section lists potentially useful practices or scenarios to inform your assessment and implementation of the specific *Recommended Practice*.

Recommended Practice – An Enterprise-wide Master Patient Index (EMPI)

1.1 An enterprise-wide master patient index (EMPI) is used to identify patients before importing data. The EMPI includes patients' demographic information and medical record number (MRN) (or multiple numbers if used by different parts of the same organization, along with the primary number/key).[12]

Implementation Status

EHR Limitation

Rationale for Practice or Risk Assessment

When patients are not matched accurately to their existing records, their health data can be fragmented across duplicate records or commingled with another patient's data, leading to patient harm.[13,14] The occurrence of duplicate records and overlays can be reduced by using an EMPI to identify patients with existing records.[12] An EMPI also facilitates record deduplication following mergers between healthcare organizations.[15]

Assessment Notes

Follow-up Actions

Person Responsible for Follow-up Action

Reset

Suggested Sources of Input

1. Health IT support staff
2. Registration staff
3. Clinical support staff

Strength of Recommendation

Medium

Implementation Guidance

- The EMPI assigns each patient a unique identifier that is different from the patient's MRN.
- Registration staff are trained to use the EMPI to look for an existing record before creating a new record.
- Organizational policies address how to use the EMPI to ensure correct patient identification of information from external sources (e.g., external labs, pharmacies, healthcare providers).
- Records with a high degree of similarity that fail to match due to missing demographic data are flagged for manual review.[16,17]
- When a new patient record is created, the registrar is prompted to consider potential matches in the existing database.
- The organization has policies and procedures to prevent the creation of duplicate records or overlays. Usability testing of the methods chosen to prevent creation of duplicates is conducted to identify opportunities for improvement.[18] The organization reviews its EMPI-related policies and procedures at least annually, updating as new recommended practices are defined.
- The EMPI employs a probabilistic matching algorithm that uses patients' first and last names, date of birth, sex, and other attributes (e.g., middle name, zip code, telephone number, last four digits of the Social Security number).[16,18] Manual adjustment or machine learning are used to tailor the algorithm for greater accuracy within an organization's context.[18-20]

Recommended Practice – Personalized Patient Lists

Implementation Status

1.2 To facilitate correct patient identification, clinicians can create personalized electronic lists of their patients according to several criteria (e.g., user, location, time, service),[21] and patient names on adjacent lines of the EHR are displayed in a visually distinct manner.[2,21,22]

Checklist

EHR Limitation

Rationale for Practice or Risk Assessment

Wrong patient selection errors often go unrecognized by clinicians.[23] Selecting a patient from a shorter list of relevant patients and keeping patient names visually distinct in the EHR reduces the risk of unintentionally selecting the wrong patient.[21-24]

Suggested Sources of Input

1. EHR developer
2. Health IT support staff

Strength of Recommendation

Strong

Assessment Notes

Follow-up Actions

Person Responsible for Follow-up Action

Reset

Implementation Guidance

- Patient lists can be automatically generated in several formats to provide information relevant to clinical or administrative needs: person-specific (e.g., all patients for whom a clinician is responsible), location-specific (e.g., all patients in a particular nursing unit or clinic), time-specific (e.g., all patients on today's schedule), and service- or clinician-specific (e.g., all patients being cared for by a particular specialty, service, or clinician).[2,22]
- Clinicians can view, create, modify, and delete patient lists for their own clinical purposes.[21]
- Patient lists are sorted in a clinically relevant order by default (e.g., by room number, appointment time), rather than alphabetically, to reduce the chance of identical, lookalike, or sound-alike names appearing close together.[2]
- Two or more unique identifiers are included for each patient on the list (e.g., name, date of birth, medical record number, sex/gender).[2,25]
- The patient’s full name is displayed including when preferred name also is displayed.[26]
- Patient list font size and spacing are optimized to reduce the chance of inadvertently selecting the wrong record.[22]
- A patient’s name is highlighted (e.g., by a distinct color, bold or italic font) when their record is selected on a patient list.[22,23]
- On all patient lists containing two or more patients with identical, lookalike, or sound-alike surnames, the names in common are displayed in a visually distinct manner (e.g., bold, italics, different color).[2,22]

Recommended Practice – Patient Identifiers on User Interface

1.3 Information required to accurately identify the patient is clearly displayed on all portions of the EHR user interface.[2]
Checklist

Implementation Status

EHR Limitation

Rationale for Practice or Risk Assessment

Providing medical services to the wrong patient is a frequent, preventable source of patient harm.[24, 27] To reduce the risk of wrong-patient errors, steps should be taken to ensure that the person using an EHR to care for a patient is addressing the intended patient. Patient names alone are not sufficient for identification, as evidenced by scenarios of mislabeled laboratory samples[28] and the significant proportion of wrong-patient events and close calls when two identifiers were not used. [29]

Suggested Sources of Input

1. EHR developer
2. Health IT support staff

Strength of Recommendation

Strong

Implementation Guidance

- All computer-generated EHR user interface displays incorporate the following information to facilitate patient identification, with appropriate exceptions for individuals for whom such information could create other risks (e.g., survivors of domestic violence): [2,30,31]
  - Full legal name (Last name, first name, middle initial)
  - Preferred name, if different from legal name
  - Date of birth (with calculated age)
  - Legal sex – required for insurance and claims processing
  - Gender identity
  - Medical record number
  - In-patient location (home address or ZIP code for outpatients)
  - Recent photograph (**see Rec 2.5)**
  - Responsible physician, if applicable
- Patient identifiers in the EHR should be displayed in a manner that promotes identity verification (e.g., using large font sizes, distinct colors, minimal visual clutter, and consistent location across various EHR screens).[31,32] This information is best displayed on the top-left of the screen, which receives more attention from users.[33]

Assessment Notes

Follow-up Actions

Person Responsible for Follow-up Action

Reset

Recommended Practice – Identifiers on Printed Materials are Clearly Displayed

Implementation Status

1.4 Materials printed from the EHR such as wristbands, labels, and reports include multiple patient identifiers and an electronic means of verifying patients' identity (e.g., a 1- or 2-dimensional barcode/QR code).

Checklist

EHR Limitation

Rationale for Practice or Risk Assessment

Materials printed from the EHR must contain multiple patient identifiers (e.g. name, date of birth, medical record number), so that patient's identity can be verified when the material is distributed to the patient (e.g., postoperative care instructions) or when the material itself is used for verifying patient identity (e.g., wristbands).[34] Verifying identity solely by confirming a patient's name and date of birth is subject to human error .[11,35,36] By incorporating barcodes into the EHR and patient care workflows, identity can be confirmed quickly and reliably by scanning.[35,37]

Assessment Notes

Follow-up Actions

Person Responsible for Follow-up Action

Reset

Suggested Sources of Input

1. EHR developer
2. Health IT support staff

Strength of Recommendation

Strong

Implementation Guidance

- All patient-specific materials printed from the EHR include the patient's full legal name and date of birth alongside a barcode to assist with patient identification.[36,38]
- At time of registration/check-in, patients are issued a wristband including their name, date of birth, and a barcode.[36,38]
- Patient identity is verified at the time of medication administration, specimen collection, procedure performance, and other key moments in patient care by verbally confirming name and date of birth as well as scanning their wristband barcode.[37,38]
- Organizational policies and workflows incorporate use of the EHR to ensure correct patient identification, such as the use of barcodes to verify patient identity at key points in the care delivery process (**see Rec 2.3**).[39,40]

Recommended Practice – Check Digits

1.5 Medical record numbers incorporate a check digit to help prevent data entry errors.
Checklist

Implementation Status

EHR Limitation

Rationale for Practice or Risk Assessment

Check digits, an extra number automatically calculated and added to a sequence of numbers to help detect errors, have been incorporated into barcoding programs to improve patient and medication safety[3] and into personal identification numbers for national registries that are often used by research communities.[41] Use of check digits contributes to high-quality data collection, mitigates patient ID number mix-ups, can help reduce data entry errors and long-term system errors, assists in the assignment of patient ID numbers to avoid sequential assignments, and reduce errors in critical scenarios where errors are known to increase.[42]

Assessment Notes

Follow-up Actions

Person Responsible for Follow-up Action

Reset

Suggested Sources of Input

1. EHR developer
2. Health IT support staff

Strength of Recommendation

Medium

Implementation Guidance

- To minimize human-generated number insertion, deletion, substitution, or transposition errors or their effects, check digits are utilized to optimize processes for correct patient identification.
- One example of a check digit program is the “Verhoeff algorithm”, which works with strings of decimal digits of any length and detects all single-digit errors and all transposition errors involving two adjacent digits.[43]
- Check digit programs are used in systems that generate pseudo-identifiers for patients whose data are used for research, to reduce data entry errors.[4]

Recommended Practice – Name Alert

Implementation Status

1.6 Users are warned when they attempt to create a record for a new patient whose first and last names are the same as another patient, or when a patient search result returns multiple patients with the same or similar names.[2]

Checklist

EHR Limitation

Rationale for Practice or Risk Assessment

Using automated EHR processes to prevent duplicate records can prevent unintentional human errors that could lead to patient harm.[18] Patients with similar names are at a higher risk for wrong-patient errors.[5]

Suggested Sources of Input

1. EHR developer
2. Health IT support staff

Strength of Recommendation

Medium

Assessment Notes

Follow-up Actions

Person Responsible for Follow-up Action

Reset

Implementation Guidance

- During the creation of a new patient record, a phonetic algorithm such as Soundex[44] is used to check for patients with similar sounding names in the system and display an alert or warning if one exists.
- When looking up a patient, if the results list returns multiple patients with similar demographic data, the names are displayed in a visually distinct manner.
- The system monitors for similar names, name variants (e.g., Robert, Rob, Bob, Robbie), or changed last names (e.g., marriage, divorce, adoption), when other demographics match.
- An alert provides additional demographic information context for the existing patient to help the user confirm or rule out that it is the same patient.
- Organizations implement an ID reentry intervention and/or a distinct naming intervention to reduce wrong-patient errors in the nursery or NICU, where sets of twins, triplets, and higher-order multiples are prevalent.[5]
- Name alerts in combination with other interventions (e.g., blood type testing) prevent patient record confusion in critical areas such as blood transfusions.[45]

Recommended Practice – Standardized Registration

Implementation Status

2.1 Patients are registered in a centralized, common database using standardized procedures.[17,46]

Checklist

EHR Limitation

Rationale for Practice or Risk Assessment

Standardized entry of full demographic data into a common database at registration improves the accuracy of patient matching and prevents the creation of duplicate charts.[17,46]

Assessment Notes

Follow-up Actions

Person Responsible for Follow-up Action

Reset

Suggested Sources of Input

1. Registration staff
2. Clinicians, support staff, and/or clinical administration
3. Health IT support staff
4. EHR developer

Strength of Recommendation

Medium

Implementation Guidance

- Organizational policy establishes standardized registration procedures involving the EHR and a common database to serve as the "source of truth" on whether a record already exists for a person who presents for services.[6]
- Registration clerks are trained in consistent patient entry practices across portals of entry (e.g., ED, inpatient, clinic, phone, internet). Entry of demographic data is standardized using national or international guidelines when possible for full name,[7] address,[47] telephone number,[48] and sex and gender identity.[8]
- Patients are asked to provide their full legal names at registration. If possible, legal name is confirmed with government-issued identification.[17] Preferred names, nicknames, and aliases are recorded in a separate field from legal name.[7,49]
- Organizations should determine the minimum set of demographic data required for reliable patient identification and interoperability in their context.[17] This might include the patient's first, middle, and last name(s), suffix, previous name(s), date of birth, sex, and current and previous addresses and phone numbers.[9]
- A multiple birth indicator is used when registering pediatric multiple birth patients (twins, triplets, etc.) to prevent subsequent merging of charts based on the similarity of demographic information.[17]
- The organization requires a picture ID to verify the identity of new patients, with appropriate alternatives for minors and others who do not have an official photo ID.[50,51]
- Photo ID or biometrics (e.g., palm vein scanning, fingerprinting, facial recognition) are used to confirm the identity of returning patients.[10,50,52-55]
- Returning patients are asked to verify the accuracy of their demographic data.[17]

Recommended Practice – Temporary Identifiers

Implementation Status

2.2 The organization has a process to assign temporary, unique patient IDs (which are later merged into permanent IDs) for when the patient registration system is unavailable, or when patients cannot be registered under their legal names.[34,56,57]

Checklist

EHR Limitation

Rationale for Practice or Risk Assessment

In some cases, patients cannot be registered under their legal names. This may occur when typical registration procedures cannot be followed because the patient registration system is unavailable or overwhelmed by a large number of incoming patients.[58,59] This can also occur when the patient's identity is unknown (e.g., a trauma victim), when the patient has not been formally named (i.e., a newborn), or when the patient's safety or privacy could be compromised by use of their legal name (e.g., a public figure or hospital employee). In these circumstances, patients must be assigned a temporary ID, which will later be merged with a permanent ID to avoid maintaining duplicate records.

Assessment Notes

Follow-up Actions

Person Responsible for Follow-up Action

Reset

Suggested Sources of Input

1. EHR developer
2. Health IT support staff
3. Registration staff
4. Clinicians and clinical support staff

Strength of Recommendation

Required

Implementation Guidance

- The organization uses a distinctive naming convention for newborns.[34]
  - A temporary ID in the style of Janesboy Smith or BoyJane Smith may be used, with the addition of letters or numbers to distinguish multiple births.[60,61]
  - Alternatively, the child's given name or a pseudonym may be used.[62]
- Patients whose identities cannot be determined at admission are given IDs that are recognizable as temporary, easily distinguishable by look and sound from other temporary IDs (both in full and in any truncated form used by the organization), and not perceived as dehumanizing or offensive by patients.[56,63-66]
- The organization has a process for providing pseudonyms to patients who wish to disguise their identities for reasons of safety or privacy.
- A process is in place to provide unique temporary IDs to patients when the patient registration system is unavailable or overwhelmed.[57]
- Any downstream use of a temporary ID within a facility, or in transfers between facilities, is tracked and corrected in all electronic systems, including at transfer facilities.[57]
- A process exists to safely merge temporary IDs with permanent ones. If merging occurs during an episode of clinical care, the patient's name is updated in all systems, and safeguards are in place to prevent confusion about the patient's identity.[63]
- The organization monitors resolution of temporary IDs.

Recommended Practice – Barcoding and RFID

Implementation Status

2.3 The organization uses electronic patient identification such as barcode scanning or radio-frequency identification of patients' wristbands to confirm patients' identity at key points of patient care.[67-69]

Checklist

EHR Limitation

Rationale for Practice or Risk Assessment

To prevent wrong-patient errors, providers should confirm patients' identity using two identifiers such as name and date of birth at key points of the care process (e.g., prior to procedures and surgeries, vital sign recording, medication administration, specimen collection, and blood transfusion administration).[2,25] However, manual patient identification is prone to error,[11] and electronic patient identification – scanning a barcode or using radio-frequency identification (RFID) on a patient's wristband to confirm the patient's identity – improves compliance with patient identification and reduces wrong-patient errors.[37,67-69]

Suggested Sources of Input

1. Clinicians, support staff, and clinical administration
2. Health IT support staff
3. EHR developer

Strength of Recommendation

Strong

Implementation Guidance

- A patient's wristband with a patient identification barcode or radio frequency identification (RFID) is scanned to electronically confirm the patient's identity prior to procedures and surgeries, vital sign recordings, medication administration, specimen collections, blood transfusion administrations, and at other key points of patient care.
- The EHR prompts providers to use electronic patient identification for patient identity verification.[70]
- Patients or their healthcare proxy (e.g., infants or adults with diminished mental capacity), are asked to confirm their identity verbally in combination with electronic patient identification.[71]
- Patients are informed about the purpose of electronic patient identification and are encouraged to remind providers to use this process.[72]
- Electronic patient identification undergoes performance testing before rollout to identify technical issues, workflow problems, and other barriers to implementation.[73,74]
- The organization maintains a backup manual system for positive patient identification in case of equipment failure, EHR downtime, or other technical difficulties.[57,73]
- Policies, workflows, and processes are implemented that aim to optimize electronic patient identification practices and prevent workarounds.[35]
- Reports are created to measure compliance with electronic patient identification practices, and performance improvement projects are used to improve compliance.

Assessment Notes

Follow-up Actions

Person Responsible for Follow-up Action

Reset

Recommended Practice - Biometrics

Implementation Status

2.4 The organization uses biometrics to verify patient identity at registration and prior to providing certain types of care.
Checklist

EHR Limitation

Rationale for Practice or Risk Assessment

Biometric attributes such as faces, fingerprints, and vein patterns are specific, ubiquitous, and relatively unchanging.[75] Unlike other patient identifiers, these attributes cannot be stolen, traded, or left behind, and they are difficult to falsify. These factors make biometrics a promising option for confirming patients' identities, especially at times of high-risk clinical care such as prior to radiation therapy treatments. However, the benefits of using biometrics must be balanced against concerns about privacy and bias.[76,77]

Suggested Sources of Input

1. EHR developer
2. Health IT support staff

Strength of Recommendation

Medium

Implementation Guidance

- Biometric attributes are selected for patient identification with consideration for factors such as privacy, impact on workflow, infection risk (e.g., fomite transmission), feasibility in a given context, and accessibility and acceptability to an organization's patient population.[53,77-80]
- Patients are given the opportunity to offer informed consent for the collection of biometrics or to opt-out.[79]
- When possible, biometrics are gathered from new patients at the time of registration.[50]
- Biometric identification is used as part of patient identification at the point of care,[81] especially at times of high-risk clinical care, such as prior to radiation therapy treatments.
- Biometrics are used in combination with other identifiers to match patients to their existing records.[76]
- The organization has a process to handle a mismatch between a patient's stated identity and the identity associated with the patient's biometric data in an existing record. A patient may present under a false name for diverse reasons – for example, to avoid retaliation from a trafficker or to engage in medical identity fraud[54,82] – which require different responses from the organization.
- Policies, workflows, and processes are implemented that aim to optimize biometric identification practices and prevent workarounds.
- Reports are created to measure compliance with biometric identification practices, and performance improvement projects are used to improve compliance.

Assessment Notes

Follow-up Actions

Person Responsible for Follow-up Action

Reset

Recommended Practice – Patient Photos

Implementation Status

2.5 Patient photographs are collected during patient registration and displayed in multiple places in the EHR to improve patient identification.[83]

Checklist

EHR Limitation

Rationale for Practice or Risk Assessment

The display of color patient photographs in the main banner of an EHR, inpatient lists, and in other areas of the EHR, when utilized either on desktop computers or mobile devices, is an effective, non-interruptive method to improve patient identification and reduce wrong patient errors.[10,30,32,83-85]

Assessment Notes

Follow-up Actions

Person Responsible for Follow-up Action

Reset

Suggested Sources of Input

1. EHR developer
2. Registration Staff

Strength of Recommendation

Strong

Implementation Guidance

- The organization collects a color photograph of every patient older than three months of age at the time of patient registration, admission to the hospital, or any time staff believe a change in appearance warrants updating the photograph.[10,83,86]
- Patient photographs are displayed in all screens and functions of the EHR supported by the vendor, including patient banners, patient lists, patient scheduling, patient search, and secure messaging.
- Patient photographs are displayed in the EHR on all devices supported by the vendor including desktop computers and mobile devices.
- Policies and practices provide guidance for capturing patient photographs, including when and how to capture them, and describing the optimal patient photo (e.g., the patient's face is centered and greater than 50% of the image). These practices are sensitive to patient cultural and religious practices with regard to face and head coverings.
- Reports are utilized to monitor the compliance of capturing patient photographs, and performance improvement projects are utilized to improve compliance.
- When patient photographs are not supported by the vendor or unavailable, other functions are used to improve patient identification such as patient identification alerts or "re-entering" patient identifiers (e.g., initials, name) before signing orders.[5,27]

Recommended Practice – Deceased Patients

Implementation Status

2.6 Patients who have died are accurately and clearly identified as deceased.
Checklist

EHR Limitation

Rationale for Practice or Risk Assessment

Selection of a deceased patient record may lead to a wrong-patient error, yet clear flags identifying that patients have deceased are often missing in EHRs. Clinicians should be able to easily identify that patients they have selected are deceased.[87,88]

Suggested Sources of Input

1. EHR developer
2. Health IT support staff

Strength of Recommendation

Medium

Implementation Guidance

- The EHR should clearly identify which patients are deceased (e.g., through a different background color for the deceased patient header in the EHR or a pop-up alert when opening the record). Care should be taken to avoid using ambiguous, culturally, or religiously insensitive icons.
- There is a mechanism to verify the death status or indicate that death is unverified (e.g., when the death data is obtained through external data sources).[89,90]
- Linkage or probabilistic matching algorithms help confirm or supply missing data,[87,88] and may cross-check EHRs with government data or national registries.[88,91]
- Accurate death status along with mechanisms to prevent entering billing adjustments as patient visits, removing recurring radiation visits from deceased patient charts, and differentiating classification of post-mortem medical activities such as autopsy procedures and organ donation could greatly reduce instances of apparent post-death health encounters.[87]

Assessment Notes

Follow-up Actions

Person Responsible for Follow-up Action

Reset

Recommended Practice – Monitoring of Poor Compliance of Patient Identification and Wrong Patient Errors

3.1 The organization monitors for patient identification errors.[12,92]

Checklist

Implementation Status

EHR Limitation

Rationale for Practice or Risk Assessment

Patient identification errors are never events that lead to adverse outcomes including death, and should be identified and acted upon as soon as possible.[7]

Assessment Notes

Follow-up Actions

Person Responsible for Follow-up Action

Reset

Suggested Sources of Input

1. EHR developer
2. Health IT support staff

Strength of Recommendation

Strong

Implementation Guidance

- Electronic patient identification practices (e.g. barcoding, biometrics) and internal voluntary reporting error databases are monitored, and performance improvement initiatives are initiated when poor compliance or patient identification hazards are identified.
- The organization has processes to monitor for common scenarios related to wrong patient identification (e.g., changes in patient blood type over time) and to implement corrective actions as needed.[93]
- The NQF-endorsed "retract–and–reorder" (RAR) algorithm is used to measure the rate of wrong patient ordering errors, and corrective actions are implemented as needed.[27,94]

Recommended Practice – Monitoring failures to Create, Access, and Maintain One Unique Medical Record for Each Patient

3.2 The organization monitors and rapidly remediates errors that stem from the failure to create, access, and maintain one unique medical record for each patient (i.e., duplicates, overlays, and overlaps).[6,7]

Checklist

Implementation Status

EHR Limitation

Rationale for Practice or Risk Assessment

Several different process error scenarios have been identified that result in the failure to correctly produce one unique medical record for each patient.[7] A duplicate record is a redundant record created when two or more medical record numbers are created for the same person; an overlay occurs when the incorrect patient is registered, admitted, or documented in another patient's record; and an overlap occurs when there is more than one unique patient identifier for the same person across two or more facilities in the enterprise and usually arises after institutional merging.[15] To minimize patient safety issues, patient misidentification errors, and billing and coding errors,[20,95-97] organizations must implement strategies to prevent duplicates, overlays, and overlaps, and to correct patient's records by de-duplicating, disentangling, or merging records when these errors are identified.

Assessment Notes

Follow-up Actions

Person Responsible for Follow-up Action

Reset

Suggested Sources of Input

1. EHR developer
2. Health IT support staff

Strength of Recommendation

Strong

Implementation Guidance

- The organization has a stringent daily process for working with the matching error queue and remediating errors identified to facilitate better patient matching.[7,20,96]
- The organization monitors its duplicate, overlay and overlap error rates, benchmarks them to internal rates quarterly, and ensures that those rates remain at or below industry standards.[7,12,94]
- Once identified, duplicate, overlaid, and overlapped records are immediately remediated.[18,88,98,99]
- In the event that a large number of duplicates are identified, such as during a health system merger, the organization immediately flags those charts as being of concern and creates a time-bound plan for resolving the issues.
- Once identified, duplicate, overlaid, and overlapped records are reviewed to identify any clinical care provided since the creation of the anomalous record situation. Responsible clinicians are notified of the issue so that appropriate patient care interventions can be performed. In addition, the organization should have a policy and procedure describing how these charts should be notated in the event a future medicolegal issue arises.
- The organization/EHR uses algorithms for patient matching that yield the lowest rates of false positives and false negatives to prevent errors from occurring. Machine learning, deep learning, pattern-recognition, natural language processing, and referential matching models[19,20,96,100] perform better than traditional probabilistic, rules-based, and deterministic algorithms[101,102] and should be incorporated into patient matching pipelines.

References

1. Kowalczyk L. Brigham and Women's Hospital video uses slapstick to promote patient safety. Boston.Com. 2013. https://www.boston.com/uncategorized/noprimarytagmatch/2013/03/07/brigham-and-womens-hospital-video-uses-slapstick-to-promote-patient-safety/. Accessed July 23, 2024.

2. Lowry SZ, Quinn MT, Ramaiah M, et al. Technical evaluation, testing, and validation of the usability of electronic health records. National Institute of Standards and Technology. 2012.

3. Neuenschwander M, Cohen MR, Vaida AJ, Patchett JA, Kelly J, Trohimovich B. Practical guide to bar coding for patient medication safety. Am J Health Syst Pharm. 2003;60(8):768-779. https://pubmed.ncbi.nlm.nih.gov/12749163/. 10.1093/ajhp/60.8.768; PMID 12749163.

4. Olden M, Holle R, Heid IM, Stark K. Idgenerator: Unique identifier generator for epidemiologic or clinical studies. BMC Med Res Methodol. 2016;16:120. https://pubmed.ncbi.nlm.nih.gov/27628043/. 10.1186/s12874-016-0222-3; PMID 27628043; PMC5024489.

5. Adelman JS, Aschner JL, Schechter CB, et al. Evaluating serial strategies for preventing wrong-patient orders in the NICU. Pediatrics. 2017;139(5). https://pubmed.ncbi.nlm.nih.gov/28557730/. 10.1542/peds.2016-2863; PMID 28557730.

6. McCoy AB, Wright A, Kahn MG, Shapiro JS, Bernstam EV, Sittig DF. Matching identifiers in electronic health records: Implications for duplicate records and patient safety. BMJ Qual Saf. 2013;22(3):219-224. https://pubmed.ncbi.nlm.nih.gov/23362505/. 10.1136/bmjqs-2012-001419; PMID 23362505.

7. American Health Information Management Association (AHIMA). Recommended data elements for capture in the master patient index (MPI). 2021. https://ahima.org/media/mezosx50/2022-naming-policy-v3-1-21-22.pdf. Accessed July 29, 2024.

8. Office of the National Coordinator for Health Information Technology (ONC). Sex at birth, sexual orientation and gender identity. 2024. https://www.healthit.gov/isp/section/sex-birth-sexual-orientation-and-gender-identity. Accessed July 23, 2024.

9. Office of the National Coordinator for Health Information Technology (ONC). Connecting health and care for the nation: A shared nationwide interoperability roadmap. 2015. https://www.healthit.gov/sites/default/files/hie-interoperability/nationwide-interoperability-roadmap-final-version-1.0.pdf. Accessed August 1, 2024.

10. Riplinger L, Piera-Jiménez J, Dooling JP. Patient identification techniques - approaches, implications, and findings. Yearb Med Inform. 2020;29(1):81-86. https://pubmed.ncbi.nlm.nih.gov/32823300/. 10.1055/s-0040-1701984; PMID 32823300; PMC7442501.

11. Henneman PL, Fisher DL, Henneman EA, Pham TA, Campbell MM, Nathanson BH. Patient identification errors are common in a simulated setting. Ann Emerg Med. 2010;55(6):503-509. https://pubmed.ncbi.nlm.nih.gov/20031263/. 10.1016/j.annemergmed.2009.11.017; PMID 20031263.

12. Dooling JA, Durkin S, Fernandes L, et al. Managing the integrity of patient identity in health information exchange (updated). J AHIMA. 2014;85(5):60-65. https://pubmed.ncbi.nlm.nih.gov/24938040/. PMID 24938040.

13. Dennison D. Patient identity management maturity model (PIM3) for imaging information technology systems. J Digit Imaging. 2021;34(2):473-482. https://pubmed.ncbi.nlm.nih.gov/33796987/. 10.1007/s10278-021-00429-2; PMID 33796987; PMC8289952.

14. Joffe E, Bearden CF, Byrne MJ, Bernstam EV. Duplicate patient records--implication for missed laboratory results. AMIA Annu Symp Proc. 2012;2012:1269-1275. https://pubmed.ncbi.nlm.nih.gov/23304405/. PMID 23304405; PMC3540536.

15. Crew D, Houser SH. Overcoming challenges of merging multiple patient identification and matching systems: A case study. Perspect Health Inf Manag. 2021;18(Winter):1n. https://pubmed.ncbi.nlm.nih.gov/33633524/. PMID 33633524; PMC7883361.

16. Just BH, Marc D, Munns M, Sandefer R. Why patient matching is a challenge: Research on master patient index (MPI) data discrepancies in key identifying fields. Perspect Health Inf Manag. 2016;13(Spring):1e. https://pubmed.ncbi.nlm.nih.gov/27134610/. PMID 27134610; PMC4832129.

17. Heflin E, He S, Isbell K, et al. A framework for cross-organizational patient identity management. 2018. https://sequoiaproject.org/wp-content/uploads/2018/06/The-Sequoia-Project-Framework-for-Patient-Identity-Management-v31.pdf. Accessed July 23, 2024.

References

18. Khunlertkit A, Dorissaint L, Chen A, Paine L, Pronovost PJ. Reducing and sustaining duplicate medical record creation by usability testing and system redesign. J Patient Saf. 2021;17(7):e665-e671. https://pubmed.ncbi.nlm.nih.gov/29076957/. 10.1097/PTS.0000000000000434; PMID 29076957.

19. Nelson W, Khanna N, Ibrahim M, et al. Optimizing patient record linkage in a master patient index using machine learning: Algorithm development and validation. JMIR Form Res. 2023;7:e44331. https://pubmed.ncbi.nlm.nih.gov/37384382/. 10.2196/44331; PMID 37384382; PMC10365597.

20. Redfield C, Tlimat A, Halpern Y, et al. Derivation and validation of a machine learning record linkage algorithm between emergency medical services and the emergency department. J Am Med Inform Assoc. 2020;27(1):147-153. https://pubmed.ncbi.nlm.nih.gov/31605488/. 10.1093/jamia/ocz176; PMID 31605488; PMC7647245.

21. Brown B, Balatsoukas P, Williams R, Sperrin M, Buchan I. Multi-method laboratory user evaluation of an actionable clinical performance information system: Implications for usability and patient safety. J Biomed Inform. 2018;77:62-80. https://pubmed.ncbi.nlm.nih.gov/29146562 /. 10.1016/j.jbi.2017.11.008; PMID 29146562 PMC5766660.

22. Shin GW, Lee Y, Park T, et al. Investigation of usability problems of electronic medical record systems in the emergency department. Work. 2022;72(1):221-238. https://pubmed.ncbi.nlm.nih.gov/34120924/. 10.3233/WOR-205262; PMID 34120924.

23. Taieb-Maimon M, Plaisant C, Hettinger AZ, Shneiderman B. Increasing recognition of wrong-patient errors through improved interface design of a computerized provider order entry system. Int J Hum Comput Interact. 2018;34(5):383-398. 10.1080/10447318.2017.1349249.

24. Mardon R, Olinger L, Szekendi M, Williams T, Sparnon E, Zimmer K. Health information technology adverse event reporting: Analysis of two databases. 2014. https://www.healthit.gov/sites/default/files/Health_IT_PSO_Analysis_Final_Report_11-25-14.pdf. Accessed August 1, 2024.

25. The Joint Commission. 2024 Hospital National Patient Safety Goals. 2024. https://www.jointcommission.org/standards/national-patient-safety-goals/hospital-national-patient-safety-goals/. Accessed July 29, 2024.

26. Sopan A, Plaisant C, Powsner S, Shneiderman B. Reducing wrong patient selection errors: Exploring the design space of user interface techniques. AMIA Annu Symp Proc. 2014;2014:1056-1065. https://pubmed.ncbi.nlm.nih.gov/25954415/. PMID 25954415; PMC4420010.

27. Adelman JS, Kalkut GE, Schechter CB, et al. Understanding and preventing wrong-patient electronic orders: A randomized controlled trial. J Am Med Inform Assoc. 2013;20(2):305-310. https://pubmed.ncbi.nlm.nih.gov/22753810/. 10.1136/amiajnl-2012-001055; PMID 22753810; PMC3638184.

28. Hawker CD, McCarthy W, Cleveland D, Messinger BL. Invention and validation of an automated camera system that uses optical character recognition to identify patient name mislabeled samples. Clin Chem. 2014;60(3):463-470. https://pubmed.ncbi.nlm.nih.gov/24366726/. 10.1373/clinchem.2013.215434; PMID 24366726.

29. Kulju S, Morrish W, King L, Bender J, Gunnar W. Patient misidentification events in the veterans health administration: A comprehensive review in the context of high-reliability health care. J Patient Saf. 2022;18(1):e290-e296. https://pubmed.ncbi.nlm.nih.gov/32925569/. 10.1097/pts.0000000000000767; PMID 32925569.

30. Van Hal C, Mills JL, Gatmaitan M, Gong Y. A patient-centered approach to collecting and displaying patient identifiers. Stud Health Technol Inform. 2024;310:369-373. https://pubmed.ncbi.nlm.nih.gov/38269827/. 10.3233/shti230989; PMID 38269827.

31. Gomes KM, Riggs SL. Analyzing visual search techniques using eye tracking for a computerized provider order entry (CPOE) task. Proceedings of the Human Factors and Ergonomics Society. 2017;2017-October:691-695. 10.1177/1541931213601659.

32. Fortman E, Hettinger AZ, Howe JL, et al. Varying rates of patient identity verification when using computerized provider order entry. J Am Med Inform Assoc. 2020;27(6):924-928. https://pubmed.ncbi.nlm.nih.gov/32377679/. 10.1093/jamia/ocaa047; PMID 32377679; PMC7647277.

33. Segel E, Heer J. Narrative visualization: Telling stories with data. IEEE Transactions on Visualization and Computer Graphics. 2010;16(6):1139-1148. 10.1109/TVCG.2010.179.

References

34. The Joint Commission. National Patient Safety Goals effective January 2024. 2024;2024(July 30). https://www.jointcommission.org/standards/national-patient-safety-goals/. Accessed July 31, 2024.

35. Barakat S, Franklin BD. An evaluation of the impact of barcode patient and medication scanning on nursing workflow at a UK teaching hospital. Pharmacy (Basel). 2020;8(3). https://pubmed.ncbi.nlm.nih.gov/32824909/. 10.3390/pharmacy8030148; PMID 32824909; PMC7560167.

36. De Rezende HA, Melleiro MM, Marques PAO, Barker TH. Interventions to reduce patient identification errors in the hospital setting: A systematic review. Open Nurs J. 2021;15:109-121. 10.2174/1874434602115010109.

37. Vanneman MW, Balakrishna A, Lang AL, et al. Improving transfusion safety in the operating room with a barcode scanning system designed specifically for the surgical environment and existing electronic medical record systems: An interrupted time series analysis. Anesth Analg. 2020;131(4):1217-1227. https://pubmed.ncbi.nlm.nih.gov/32925343/. 10.1213/ane.0000000000005084; PMID 32925343.

38. Saathoff AM, MacDonald R, Krenzischek E. Effectiveness of specimen collection technology in the reduction of collection turnaround time and mislabeled specimens in emergency, medical-surgical, critical care, and maternal child health departments. Comput Inform Nurs. 2018;36(3):133-139. https://pubmed.ncbi.nlm.nih.gov/29120913/. 10.1097/cin.0000000000000402; PMID 29120913.

39. Ning HC, Lin CN, Chiu DT, et al. Reduction in hospital-wide clinical laboratory specimen identification errors following process interventions: A 10-year retrospective observational study. PLoS One. 2016;11(8):e0160821. https://pubmed.ncbi.nlm.nih.gov/27494020/. 10.1371/journal.pone.0160821; PMID 27494020; PMC4975414.

40. Dhatt GS, Damir HA, Matarelli S, Sankaranarayanan K, James DM. Patient safety: Patient identification wristband errors. Clin Chem Lab Med. 2011;49(5):927-929. https://pubmed.ncbi.nlm.nih.gov/21288177/. 10.1515/cclm.2011.129; PMID 21288177.

41. Sund R, Gissler M. Use of health registers. New York, NY: Springer New York; 2019.

42. Oza S, Wing K, Sesay AA, et al. Improving health information systems during an emergency: Lessons and recommendations from an ebola treatment centre in Sierra Leone. BMC Med Inform Decis Mak. 2019;19(1):100. https://pubmed.ncbi.nlm.nih.gov/31133075/. 10.1186/s12911-019-0817-9; PMID 31133075; PMC6537453.

43. Chu M, Kang G, Ryu KH. An improved check digit-based participant identification system for human biorepositories. 2023 Asia Pacific Signal and Information Processing Association Annual Summit and Conference, APSIPA ASC 2023. 2023:1614-1621. https://www.scopus.com/inward/record.uri?eid=2-s2.0-85180010293&doi=10.1109%2fAPSIPAASC58517.2023.10317197&partnerID=40&md5=90c35b152da43b2c21b9c7fd2cef6c90.

44. The U.S. National Archives and Records Administration. Soundex System | The Soundex Indexing System. Updated Jan 9, 2024. https://www.archives.gov/research/census/soundex. Accessed August 6, 2024.

45. Ferrera-Tourenc V, Lassale B, Chiaroni J, Dettori I. Unreliable patient identification warrants ABO typing at admission to check existing records before transfusion. Transfus Clin Biol. 2015;22(2):66-70. https://pubmed.ncbi.nlm.nih.gov/25936944/. 10.1016/j.tracli.2015.03.004; PMID 25936944.

46. Grannis SJ, Xu H, Vest JR, et al. Evaluating the effect of data standardization and validation on patient matching accuracy. J Am Med Inform Assoc. 2019;26(5):447-456. https://pubmed.ncbi.nlm.nih.gov/30848796/. 10.1093/jamia/ocy191; PMID 30848796; PMC7787357.

47. Project US@. Project US@: Technical specifications for patient addresses, domestic and military. 2022:65. https://asapnet.org/wp-content/uploads/2022/03/Project_US_FINAL_Technical_Specification_Version_1.0.pdf. Accessed August 1, 2024.

48. International Telecommunication Union. ITU Recommendation E.123: Notation for national and international telephone numbers, e-mail addresses and web addresses. 2001. https://www.itu.int/itu-t/recommendations/rec.aspx?rec=5341. Accessed July 24, 2024.

References

49. Frangella J, Cassarino M, Plazzotta F, Gassino F, Otero C, Luna D. Designed strategies and adaptation of a master patient index for transgender patients in a tertiary care hospital. Stud Health Technol Inform. 2019;264:1698-1699. https://pubmed.ncbi.nlm.nih.gov/31438299/. 10.3233/SHTI190603; PMID 31438299.

50. Abel L, Buegel RA, Dooling JA, et al. Best practices for patient matching at patient registration. J AHIMA. 2016;87(10):74-81.

51. LeBrón AMW, Cowan K, Lopez WD, Novak NL, Ibarra-Frayre M, Delva J. It works, but for whom? Examining racial bias in carding experiences and acceptance of a county identification card. Health Equity. 2018;2(1):239-249. https://pubmed.ncbi.nlm.nih.gov/30283873/. 10.1089/heq.2018.0022; PMID 30283873; PMC6167006.

52. Stanuch M, Wodzinski M, Skalski A. Contact-free multispectral identity verification system using palm veins and deep neural network. Sensors (Basel). 2020;20(19). https://pubmed.ncbi.nlm.nih.gov/33036259/. 10.3390/s20195695; PMID 33036259; PMC7582870.

53. Waruhari P, Babic A, Nderu L, Were MC. A review of current patient matching techniques. Stud Health Technol Inform. 2017;238:205-208. https://pubmed.ncbi.nlm.nih.gov/28679924/. PMID 28679924.

54. Katsanis SH, Huang E, Young A, et al. Caring for trafficked and unidentified patients in the EHR shadows: Shining a light by sharing the data. PLoS One. 2019;14(3):e0213766. https://pubmed.ncbi.nlm.nih.gov/30870468/. 10.1371/journal.pone.0213766; PMID 30870468; PMC6417704.

55. Basavatia A, Fret J, Lukaj A, et al. Right care for the right patient each and every time. Cureus. 2016;8(2):e492. https://pubmed.ncbi.nlm.nih.gov/27014526/. 10.7759/cureus.492; PMID 27014526; PMC4792635.

56. Landman A, Teich JM, Pruitt P, et al. The Boston Marathon bombings mass casualty incident: One emergency department's information systems challenges and opportunities. Ann Emerg Med. 2015;66(1):51-59. https://pubmed.ncbi.nlm.nih.gov/24997562/. 10.1016/j.annemergmed.2014.06.009; PMID 24997562.

57. The Joint Commission. New and revised emergency management standards for ambulatory care programs. R3 report. 2023;39(July 30). https://www.jointcommission.org/standards/r3-report/r3-report-issue-39-new-and-revised-emergency-management-standards-for-ambulatory-care-programs/ Accessed July 31, 2024.

58. Larsen E, Fong A, Wernz C, Ratwani RM. Implications of electronic health record downtime: An analysis of patient safety event reports. J Am Med Inform Assoc. 2018;25(2):187-191. https://pubmed.ncbi.nlm.nih.gov/28575417/. 10.1093/jamia/ocx057; PMID 28575417; PMC7647128.

59. Cohen R, Ning S, Yan MTS, Callum J. Transfusion safety: The nature and outcomes of errors in patient registration. Transfus Med Rev. 2019;33(2):78-83. https://pubmed.ncbi.nlm.nih.gov/30626535/. 10.1016/j.tmrv.2018.11.004; PMID 30626535.

60. Adelman J, Aschner J, Schechter C, et al. Use of temporary names for newborns and associated risks. Pediatrics. 2015;136(2):327-333. https://pubmed.ncbi.nlm.nih.gov/26169429/. 10.1542/peds.2015-0007; PMID 26169429.

61. Pfeifer E, Lozovatsky M, Abraham J, Kannampallil T. Effect of an alternative newborn naming strategy on wrong-patient errors: A quasi-experimental study. Appl Clin Inform. 2020;11(2):235-241. https://pubmed.ncbi.nlm.nih.gov/32236916/. 10.1055/s-0040-1705175; PMID 32236916; PMC7112998.

62. Besagar S, Robles PL, Rojas C, Applebaum JR, Adelman JS, Goffman D. Acceptability of using newborns' given names at birth: Survey in postpartum and antepartum units. Obstet Gynecol. 2020;135:156S-157S.

63. Janowak CF, Janowak LM. Misidentifying the unidentified – John Doe and the EHR. 2017. https://psnet.ahrq.gov/web-mm/misidentifying-unidentified-john-doe-and-ehr. Accessed August 1, 2024.

64. Brooks AJ, Macnab C, Boffard K. AKA unknown male Foxtrot 23/4: Alias assignment for unidentified emergency room patients. J Accid Emerg Med. 1999;16(3):171-173. https://pubmed.ncbi.nlm.nih.gov/10353040/. PMID 10353040; PMC1343326.

References

65. Blank-Reid CA, Kaplan LJ. A system for working with unidentified trauma patients. Int J Trauma Nurs. 1996;2(4):108-110. https://pubmed.ncbi.nlm.nih.gov/9079339/. 10.1016/S1075-4210(96)80071-X; PMID 9079339.

66. Robinson G, Fortune JB, Wachtel TL, Frank HA, Long WB. A system of alias assignment for unidentified patients requiring emergency hospital admission. J Trauma Acute Care Surg. 1985;25(4):333. https://pubmed.ncbi.nlm.nih.gov/3989892/. PMID 3989892.

67. Kaufman RM, Dinh A, Cohn CS, et al. Electronic patient identification for sample labeling reduces wrong blood in tube errors. Transfusion. 2019;59(3):972-980. https://pubmed.ncbi.nlm.nih.gov/30549289/. 10.1111/trf.15102; PMID 30549289.

68. Nayeri ND, Nadali J, Divani A, Hatefimoadab N. Ways to enhance blood transfusion safety: A systematic review. Florence Nightingale J Nurs. 2022;30(3):288-300. https://pubmed.ncbi.nlm.nih.gov/36106812/. 10.5152/fnjn.2022.21214; PMID 36106812; PMC9623141.

69. Hutton K, Ding Q, Wellman G. The effects of bar-coding technology on medication errors: A systematic literature review. J Patient Saf. 2021;17(3):e192-e206. https://pubmed.ncbi.nlm.nih.gov/28234729/. 10.1097/pts.0000000000000366; PMID 28234729.

70. Steitz BD, Li G, Wright A, Dunworth B, Freundlich RE, Wanderer JP. Non-interruptive clinical decision support to improve perioperative electronic positive patient identification. J Med Syst. 2022;46(3):15. https://pubmed.ncbi.nlm.nih.gov/35079867/. 10.1007/s10916-022-01801-7; PMID 35079867; PMC8862728.

71. Henneman PL, Marquard JL, Fisher DL, et al. Bar-code verification: Reducing but not eliminating medication errors. J Nurs Adm. 2012;42(12):562-566. https://pubmed.ncbi.nlm.nih.gov/23151928/. 10.1097/NNA.0b013e318274b545; PMID 23151928.

72. Chou SS, Chen YJ, Shen YT, Yen HF, Kuo SC. Implementation and effectiveness of a bar code-based transfusion management system for transfusion safety in a tertiary hospital: Retrospective quality improvement study. JMIR Med Inform. 2019;7(3):e14192. https://pubmed.ncbi.nlm.nih.gov/31452517/. 10.2196/14192; PMID 31452517; PMC6732972.

73. Al-Eshaq DH, Bradley RT, McBride ERA, Ford JC. Patient and specimen identification in a tertiary care pediatric hospital: Barcodes do not scan themselves. Transfusion. 2023;63(7):1310-1317. https://pubmed.ncbi.nlm.nih.gov/37226989/. 10.1111/trf.17399; PMID 37226989.

74. San TH, Lin SKS, Fai CM. Factors affecting registered nurses' use of medication administration technology in acute care settings: A systematic review. JBI Evidence Synthesis. 2012;10(8):471. https://pubmed.ncbi.nlm.nih.gov/27820547/. 10.11124/jbisrir-2012-55; PMID 27820547.

75. Barboi C, Dixon BE, McFarlane TD, Grannis SJ. Chapter 12 - Client Registries: Identifying and linking patients. Academic Press; 2023.

76. Rudin RS, Hillestad R, Ridgely MS, Qureshi N, Davis JS, II, Fischer SH. Defining and evaluating patient-empowered approaches to improving record matching. 2018. https://www.rand.org/pubs/research_reports/RR2275.html. Accessed August 1, 2024.

77. Khan LM, Slaughter RK, Bedyoa A. Policy statement of the Federal Trade Commission on biometric information and Section 5 of the Federal Trade Commission act. 2023. https://www.ftc.gov/system/files/ftc_gov/pdf/p225402biometricpolicystatement.pdf. Accessed July 29, 2024.

78. Jeon B, Jeong B, Jee S, et al. A facial recognition mobile app for patient safety and biometric identification: Design, development, and validation. JMIR Mhealth Uhealth. 2019;7(4):e11472. https://pubmed.ncbi.nlm.nih.gov/30958275/. 10.2196/11472; PMID 30958275; PMC6475824.

79. Wells A, Usman AB. Privacy and biometrics for smart healthcare systems: Attacks, and techniques. Information Security Journal: A Global Perspective. 2024;33(3):307-331. 10.1080/19393555.2023.2260818.

80. Tay KY, Pang YH, Ooi SY, Goh FL. Contactless patient authentication for registration using face recognition technology. Paper presented at: Lecture Notes in Electrical Engineering 2021.

References

81. Sawa M, Inoue T, Manabe S. Biometric palm vein authentication of psychiatric patients for reducing in-hospital medication errors: A pre-post observational study. BMJ Open. 2022;12(4):e055107. https://pubmed.ncbi.nlm.nih.gov/35487740/. 10.1136/bmjopen-2021-055107; PMID 35487740; PMC9058808.

82. Judson T, Haas M, Lagu T. Medical identity theft: Prevention and reconciliation initiatives at Massachusetts General Hospital. Jt Comm J Qual Patient Saf. 2014;40(7):291-295. https://pubmed.ncbi.nlm.nih.gov/25130011/. 10.1016/s1553-7250(14)40038-2; PMID 25130011.

83. The Joint Commission. People, processes, health IT and accurate patient identification. 2018. https://www.jointcommission.org/-/media/tjc/newsletters/qs_hit_and_patient_id_9_25_18_finalpdf.pdf Accessed July 29, 2024.

84. Salmasian H, Blanchfield BB, Joyce K, et al. Association of display of patient photographs in the electronic health record with wrong-patient order entry errors. JAMA Netw Open. 2020;3(11):e2019652. https://pubmed.ncbi.nlm.nih.gov/33175173/. 10.1001/jamanetworkopen.2020.19652; PMID 33175173; PMC7658731.

85. Rzewnicki D, Kanvinde A, Gillespie S, Orenstein E. Association of patient photographs and reduced retract-and-reorder events. JAMIA Open. 2024;7(3):ooae042. https://pubmed.ncbi.nlm.nih.gov/38957593/. 10.1093/jamiaopen/ooae042; PMID 38957593; PMC11218880.

86. Hyman D, Laire M, Redmond D, Kaplan DW. The use of patient pictures and verification screens to reduce computerized provider order entry errors. Pediatrics. 2012;130(1):e211-219. https://pubmed.ncbi.nlm.nih.gov/22665415/. 10.1542/peds.2011-2984; PMID 22665415.

87. Delgado M, Dard S, Jonsson Funk M, Carey T. Explaining the inexplicable: Irregularities in electronic health record derived data. Pharmacoepidemiol Drug Saf. 2020;29(S3):335. 10.1002/pds.5114.

88. Li X, Xu H, Grannis S. The data-adaptive fellegi-sunter model for probabilistic record linkage: Algorithm development and validation for incorporating missing data and field selection. J Med Internet Res. 2022;24(9):e33775. https://pubmed.ncbi.nlm.nih.gov/36173664/. 10.2196/33775; PMID 36173664; PMC9562057.

89. Shao P, Tepsick JG, Walker B, Ray HE. Improving real-world mortality data quality in oncology research: Augmenting electronic medical records with obituary, social security death index, and commercial claims data. JCO Clin Cancer Inform. 2023;7:e2300014. https://pubmed.ncbi.nlm.nih.gov/37695983/. 10.1200/cci.23.00014; PMID 37695983; PMC10569778.

90. Curtis MD, Griffith SD, Tucker M, et al. Development and validation of a high-quality composite real-world mortality endpoint. Health Serv Res. 2018;53(6):4460-4476. https://pubmed.ncbi.nlm.nih.gov/29756355/. 10.1111/1475-6773.12872; PMID 29756355; PMC6232402.

91. Conway RBN, Armistead MG, Denney MJ, Smith GS. Validating the matching of patients in the linkage of a large hospital system's EHR with state and national death databases. Appl Clin Inform. 2021;12(1):82-89. https://pubmed.ncbi.nlm.nih.gov/33567463/. 10.1055/s-0040-1722220; PMID 33567463; PMC7875675.

92. Office of the National Coordinator for Health Information Technology (ONC). Patient Identification and Matching Final Report 2014. https://www.healthit.gov/sites/default/files/patient_identification_matching_final_report.pdf. Accessed August 1, 2024.

93. Elkins S. Patient matching: Are we any closer to a solution? For The Record. 2018;30(9):18. https://www.fortherecordmag.com/archives/1018p18.shtml. Accessed July 23, 2024.

94. National Quality Forum. Identification and prioritization of Health IT patient safety measures. 2016. https://www.qualityforum.org/publications/2016/02/identification_and_prioritization_of_hit_patient_safety_measures.aspx. Accessed August 1, 2024.

95. Moscovitch B, Halamka JD, Grannis S. Better patient identification could help fight the coronavirus. NPJ Digit Med. 2020;3:83. https://pubmed.ncbi.nlm.nih.gov/32529044/. 10.1038/s41746-020-0289-4; PMID 32529044; PMC7264357.

96. Grannis SJ, Williams JL, Kasthuri S, Murray M, Xu H. Evaluation of real-world referential and probabilistic patient matching to advance patient identification strategy. J Am Med Inform Assoc. 2022;29(8):1409-1415. https://pubmed.ncbi.nlm.nih.gov/35568993/. 10.1093/jamia/ocac068; PMID 35568993; PMC9277641.

References

97. Arndt RZ. Fail-safe patient matching remains just out of reach. Mod Healthc. 2018;48(29):22. https://psnet.ahrq.gov/issue/fail-safe-patient-id-matching-remains-just-out-reach. Accessed July 23, 2024.

98. Gilbert R, Lafferty R, Hagger-Johnson G, et al. Guild: Guidance for information about linking data sets. J Public Health (Oxf). 2018;40(1):191-198. https://pubmed.ncbi.nlm.nih.gov/28369581/. 10.1093/pubmed/fdx037; PMID 28369581; PMC5896589.

99. SHOT Steering Group. 2019 Annual SHOT Report. Serious Hazards of Transfusion (SHOT). 2019. https://hospital.blood.co.uk/the-update/2019-annual-shot-report/.

100. Hua Y, Wang L, Nguyen V, et al. A deep learning approach for transgender and gender diverse patient identification in electronic health records. J Biomed Inform. 2023;147:104507. https://pubmed.ncbi.nlm.nih.gov/37778672/. 10.1016/j.jbi.2023.104507; PMID 37778672; PMC10687838.

101. Ross MK, Sanz J, Tep B, Follett R, Soohoo SL, Bell DS. Accuracy of an electronic health record patient linkage module evaluated between neighboring academic health care centers. Appl Clin Inform. 2020;11(5):725-732. https://pubmed.ncbi.nlm.nih.gov/33147645/. 10.1055/s-0040-1718374; PMID 33147645; PMC7641664.

102. Nagels J, Wu S, Gorokhova V. Deterministic vs. Probabilistic: Best practices for patient matching based on a comparison of two implementations. J Digit Imaging. 2019;32(6):919-924. https://pubmed.ncbi.nlm.nih.gov/31292769/. 10.1007/s10278-019-00253-9; PMID 31292769; PMC6841798.
