# Supplementary material for: Revisions to the Safety Assurance Factors for Electronic Health Record Resilience (SAFER) Guides to update national recommendations for safe use of electronic health records
Source: J Am Med Inform Assoc. 2025 Apr 12;32(4):755–60. doi: 10.1093/jamia/ocaf018 (PMC12005625; doi:10.1093/jamia/ocaf018)
Supplement: ocaf018_Supplementary_Data [file ocaf018_supplementary_data.zip › ocaf018_Supplementary_Data/System Management Final.pdf]

Self-Assessment

# System Management

## General Instructions for the SAFER Self-Assessment Guides

The Safety Assurance Factors for EHR Resilience (SAFER) guides are designed to help healthcare organizations conduct proactive self-assessments to evaluate the safety and effectiveness of their electronic health record (EHR) implementations. The 2025 SAFER guides have been updated and streamlined to focus on the highest risk, most commonly occurring issues that can be addressed through technology or practice changes to build system resilience in the following areas:

- Organizational Responsibilities
- Patient Identification
- Clinician Communication
- Test Results Reporting and Follow-up
- Computerized Provider Order Entry with Decision Support
- Systems Management
- Contingency Planning
- High Priority Practices - A collection of 16 Recommendations from the other 7 Guides

Each of the eight SAFER Guides begins with a Checklist of recommended practices. The downloadable SAFER Guides provide fillable circles that can be used to indicate the extent to which each recommended practice has been implemented in the organization using a 5-point Likert scale. The Practice Worksheet gives a rationale for the practice and provides examples of how to implement each recommended practice. It contains fields to record team member involvement and follow-up actions based on the assessment. The Worksheet also lists the stakeholders who can provide input to assess each practice (sources of input). In addition to the downloadable version, the content of each SAFER Guide, with interactive references and supporting materials, can also be viewed on ONC's website at: https://www.healthit.gov/topic/safety/safer-guides.

The SAFER guides are based on the best available (2024) evidence from the literature and consensus expert opinion. Subject matter experts in patient safety, informatics, quality improvement, risk management, human factors engineering, and usability developed them. Furthermore, they were reviewed by an external group of practicing clinicians, informaticians, and information technology professionals.

Each guide contains between 6 and 18 recommended practices including its rationale, implementation guidance, and evidence level. The recommended practices in the SAFER Guides are intended to be useful for all EHR users. However, every organization faces unique circumstances and may implement a particular recommended practice differently. As a result, some of the specific implementation guidance in the SAFER Guides for recommended practices may not be applicable to an organization.

The High Priority Practices guide consists of 16 of the most important and relevant recommendations selected from the other 7 guides. It is designed for practicing clinicians to help them understand, implement, and support EHR safety and safe use within their organization. The other seven guides consist of 88 unique recommendations that are relevant for all healthcare providers and organizations.

The SAFER Guides are designed in part to help deal with safety concerns created by the continuously changing sociotechnical landscape that healthcare organizations face. Therefore, changes in technology, clinical practice standards, regulations, and policy should be taken into account when using the SAFER Guides. Periodic self-assessments using the SAFER Guides may also help organizations identify areas where it is particularly important to address the implications of these practice or EHR-based changes for the safety and safe use of EHRs. Ultimately, the goal is to improve the overall safety of our health care system and improve patient outcomes.

The SAFER Guides are not intended to be used for legal compliance purposes, and implementation of a recommended practice does not guarantee compliance with the HIPAA Security or Privacy Rules, Medicare or Medicaid Conditions of Participation, or any other laws or regulations. The SAFER Guides are for informational purposes only and are not intended to be an exhaustive or definitive source. They do not constitute legal advice. Users of the SAFER Guides are encouraged to consult with their own legal counsel regarding compliance with Medicare or Medicaid program requirements, and any other laws.

For additional information on Medicare and Medicaid program requirements, please visit the Centers for Medicare & Medicaid Services website at www.cms.gov. For more information on HIPAA, please visit the HHS Office for Civil Rights website at www.hhs.gov/ocr.

Self-Assessment

# System Management

## Introduction

The System Management Guide identifies recommended safety practices associated with the configuration, validation, and maintenance of electronic health record (EHR) hardware, software, and system-to-system application programming interfaces (APIs). This includes the physical environment in which the EHR will operate and the implementation and testing of technically complex components of the clinical information system.

This guide focuses on the management and monitoring protocols, policies, and practices required to enhance the safety of the EHR's technical components. It is intended to be worked through by a multidisciplinary team representing clinicians, technical staff, administrative specialists, frequent end-users, and any relevant subject matter experts who can provide additional perspectives for enhancing safety through an efficient and effective process.

**Technical Component Management**
The configuration, testing, and maintenance of the system's technical components are vulnerable processes that can result in unintended consequences and errors. EHRs are profoundly influenced by their initial configuration. Configuration decisions must be made after careful consideration of clinical processes and desired outcomes. Similarly, the technical team should make updates to the system or subsystem components in close collaboration with multidisciplinary subject matter experts including clinicians. This assures that care processes and clinical workflows are factored into system changes and ongoing technical maintenance, and that system interactions continue to function as intended and expected.

**Application Programming Interface (API) Safety Challenges**
Implementing APIs between software applications is particularly challenging. The APIs and the integrated system are often created and managed by different developers and entities external to the healthcare organization. Additionally, different APIs may have different maintenance or upgrade cycles. The APIs and their data concepts (e.g., protocols and vocabularies) may not be standardized, and they may be influenced by layers of customized mappings that are exclusive to the healthcare organization. These differences in data concepts and meanings introduce the risk of safety issues as the data may be misinterpreted or misrepresented while crossing interfaces. Meticulous ongoing attention to vocabulary code(s) and concept mappings between clinical code sets (e.g., SNOMED, LOINC, ICD-10) or between external standard and internal customized code sets, catalogs, and libraries (e.g., medication orders, diagnosis and billing codes) is imperative to ensuring patient safety.

**Timely EHR Updates and Ongoing Maintenance**
EHR systems, applications, and APIs should be regularly updated to align with the latest industry code sets, cybersecurity measures, and performance improvements. A coordinated change management process, including diligent testing targeting performance, data integrity, basic safety, usability, and including user notification, can reduce business and clinical operation disruptions while ensuring ongoing safety, effectiveness, and user satisfaction. Healthcare organizations should know in advance the EHR version and code set release cadence and sufficiently prepare to implement timely updates. Testing should be performed by healthcare organization representatives including clinicians and other frequent end users who are not part of the development group and thus are not responsible for or committed to the product's design and build.

In addition to the substantial coordination of efforts for initial setup and testing after updates, a continuous and reliable technical system monitoring, maintenance, and review process is necessary to maximize EHR benefits and identify and mitigate any patient safety risks.

**Engaging and Collaborating in the SAFER Guide Self-Assessment**
Completing the self-assessment in the System Management SAFER Guide requires the engagement of people both within and outside the organization. Because this guide is designed to help organizations prioritize EHR-related safety concerns, clinical leaders in the organization must be engaged to assess whether and how any particular recommended practice affects the organization's ability to deliver safe, high-quality care.

Collaboration between IT staff, clinicians, and other stakeholders while completing the self-assessment in this guide will enable an accurate snapshot of the organization's EHR's clinical information systems, applications, and API technical components. More importantly, this process should forge consensus on the organization's strategy to enhance EHR-related safety, quality, and effectiveness by: 1) prioritizing and addressing unmet recommended practices; 2) maintaining current recommended practices; and 3) collaborating to allocate resources for targeted improvements and mitigation of other high-priority technical safety risks introduced by the EHR.

Self-Assessment

# System Management

## Table of Contents


| | |
|---|---|
| General Instructions | 1 |
| Introduction | 2 |
| About the Checklist | 5 |
| Checklist | 6 |
| Team Worksheet | 8 |
| About the Recommended Practice Worksheets | 9 |
| Worksheets | 10 |
| References | 23 |

# Authors and Peer Reviewers

The SAFER Self-Assessment Guides were developed by health IT safety researchers and informatics experts whose contributions are acknowledged as follows:

Primary authors who contributed to the development of all guides:

**Trisha Flanagan, RN, MSN, CPPS,** Health Informatics Nurse, Center for Innovations in Quality, Effectiveness and Safety, Michael E. DeBakey Veterans Affairs Medical Center, Houston TX

**Hardeep Singh, MD, MPH,** Co-Chief, Health Policy, Quality and Informatics Program, Center for Innovations in Quality, Effectiveness and Safety and Professor of Medicine at the Michael E. DeBakey Veterans Affairs Medical Center and Baylor College of Medicine, Houston, TX

**Dean F. Sittig MS, PhD, FACMI, FAMIA, FHIMSS, FIAHSI,** Professor of Biomedical Informatics, Department of Clinical and Health Sciences, McWilliams School of Biomedical Informatics, University of Texas Health Science Center at Houston, TX and Informatics Review LLC, Lake Oswego, OR

Support staff for the primary authorship team

**Rosann Cholankeril, MD, MPH,** Center for Innovations in Quality, Effectiveness and Safety, Michael E. DeBakey Veterans Affairs Medical Center and Baylor College of Medicine

**Sara Ehsan, MBBS, MPH,** Center for Innovations in Quality, Effectiveness and Safety, Michael E. DeBakey Veterans Affairs Medical Center and Baylor College of Medicine

Additional authors who contributed to at least one guide:

**Jason S. Adelman, MD, MS,** (Patient ID) Chief Patient Safety Officer & Associate Chief Quality Officer, Executive Director, Patient Safety Research, Co-Director, Patient Safety Research Fellowship in Hospital Medicine, New York-Presbyterian Hospital/Columbia University Irving Medical Center, New York, NY

**Daniel R. Murphy, MD, MBA,** (Clinician Communication, Test Results) Chief Quality Officer, Baylor Medicine, Houston, TX

**Patricia Sengstack, DNP, NI-BC, FAAN, FACMI,** (Organizational Responsibilities) Senior Associate Dean for Informatics, Director, Nursing Informatics Specialty Program, Vanderbilt University School of Nursing, Vanderbilt University, Nashville, TN

Additional contributors who provided feedback on various guides or parts of guides

**Miriam Callahan, MD (Patient ID)**
**David C. Classen, MD (CPOE, AI recommendation)**
**Anne Grauer, MD, MS (Patient ID)**
**Ing Haviland (Patient ID)**
**Amanda Heidemann, MD (All Guides)**
**I-Fong Sun Lehman, DrPH, MS (Patient ID)**
**Christoph U. Lehmann, MD (AI recommendation)**
**Christopher A. Longhurst, MD, MS (AI recommendation)**
**Edward R. Melnick, MD (Clinician Communication)**
**Robert E. Murphy, MD (Organizational Responsibilities)**
**Ryan P. Radecki, MD, MS (AI recommendation)**
**Raj Ratwani, PhD (AI recommendation)**
**Trent Rosenbloom, MD (Clinician Communication)**
**Lisa Rotenstein, MD (Clinician Communication)**
**Hojjat Salmasian, MD, PhD (All Guides)**
**Richard Schreiber, MD (CPOE)**
**Danny Sands, MD (Clinician Communication)**
**Debora Simmons, PhD, RN (Organizational Responsibilities)**
**Carina Sirochinsky (Patient ID)**
**Neha Thummala, MPH (Patient ID)**
**Emma Weatherford (Patient ID)**
**Adam Wright, PhD (CPOE)**
**Andrew Zimolzak, MD, MMSc (Test Results, Clinician Communication)**

This guide was developed under the contract Unintended Consequences of Health IT and Health Information Exchange, Task Order HHSP23337003T/HHSP23320095655WC.

The ATSP/ONC composite mark is a mark of the U.S. Department of Health and Human Services. The contents of the publication or project are solely the responsibility of the authors and do not necessarily represent the official views of the U.S. Department of Health and Human Services, Asssistant Secretary for the Technology Policy/Office of the National Coordinator for Health Information Technology.

>Table of Contents | >About the Checklist | >Team Worksheet | >About the Practice Worksheets | >Practice Worksheets

The *Checklist* is structured as a quick way to enter and print your self-assessment.

Select the level of implementation achieved by your organization for each Recommended Practice. Your Implementation Status will be reflected on the Recommended Practice Worksheet in this PDF. The implementation status scales are as followed:

**Not Implemented – (0%)** The organization has not implemented this recommendation.

**Making Progress (1 - 30%)** The organization is in the early or pilot phase of implementing this recommendation as evidenced by following or adopting less than 30% of the implementation guidance.

**Halfway there (31 – 60%)** The organization is implementing this recommendation and is following or has adopted approximately half of the implementation guidance.

**Substantial Progress (61-90%)** The organization has nearly implemented this recommendation and is following or has adopted much of the implementation guidance.

**Fully Implemented (91-100%)** The organization follows this recommendation, and most implementation guidance is followed consistently and widely adopted.

The organization should check the following box if there are some limitations with the current version of their EHR that preclude them from fully implementing this recommendation.

**EHR Limitation -** The EHR does not offer the features/functionality required to fully implement this recommendation or the implementation guidance.

The *Domain* associated with the *Recommended Practice(s)* appears at the top of the column

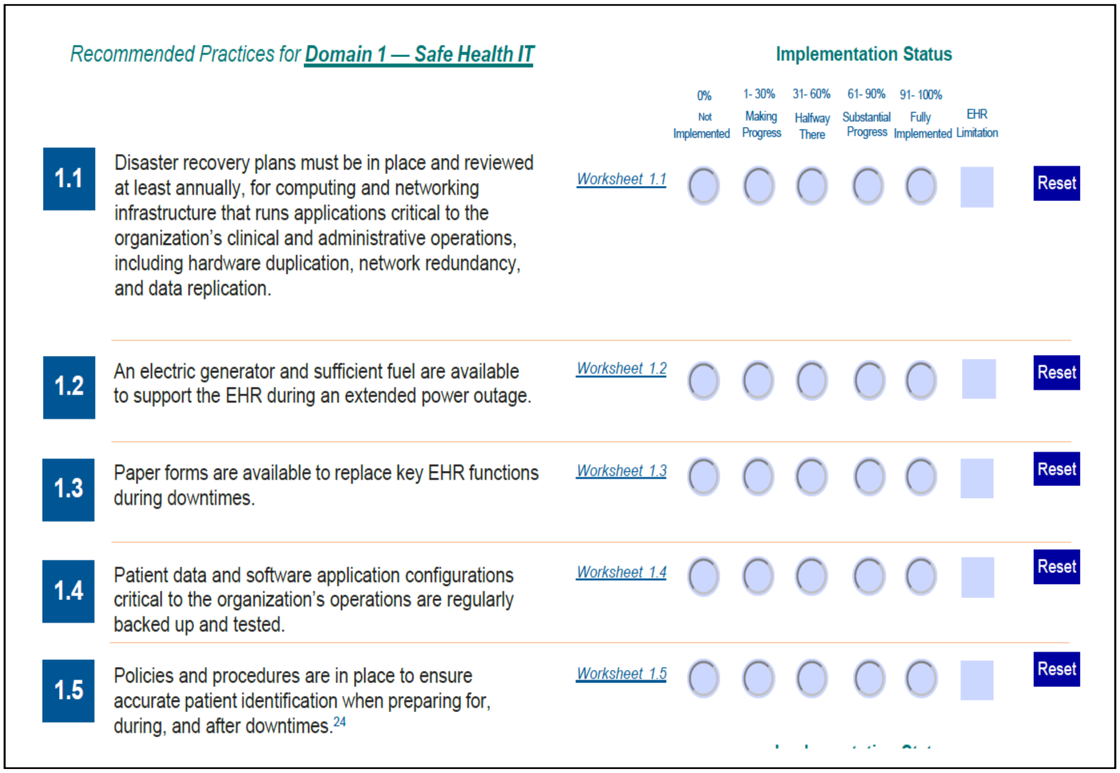

*Recommended Practices for* ***Domain 1 — Safe Health IT***

| | Recommended Practice | | Implementation Status: 0% Not Implemented | 1-30% Making Progress | 31-60% Halfway There | 61-90% Substantial Progress | 91-100% Fully Implemented | EHR Limitation | |
|---|---|---|---|---|---|---|---|---|---|
| 1.1 | Disaster recovery plans must be in place and reviewed at least annually, for computing and networking infrastructure that runs applications critical to the organization's clinical and administrative operations, including hardware duplication, network redundancy, and data replication. | Worksheet 1.1 | | | | | | | Reset |
| 1.2 | An electric generator and sufficient fuel are available to support the EHR during an extended power outage. | Worksheet 1.2 | | | | | | | Reset |
| 1.3 | Paper forms are available to replace key EHR functions during downtimes. | Worksheet 1.3 | | | | | | | Reset |
| 1.4 | Patient data and software application configurations critical to the organization's operations are regularly backed up and tested. | Worksheet 1.4 | | | | | | | Reset |
| 1.5 | Policies and procedures are in place to ensure accurate patient identification when preparing for, during, and after downtimes.[24] | Worksheet 1.5 | | | | | | | Reset |

The *Recommended Practice(s)* for the topic appears below the associated *Domain*.

To the right of reach *Recommended Practice* is a link to the Recommended Practice Worksheet in this PDF.

The *Worksheet* provides guidance on implementing the practice.

## Recommended Practices for Domain 1 — Safe Health IT

| | Recommended Practice | Worksheet | 0% Not Implemented | 1- 30% Making Progress | 31- 60% Halfway There | 61- 90% Substantial Progress | 91- 100% Fully Implemented | EHR Limitation | |
|---|---|---|---|---|---|---|---|---|---|
| 1.1 | A sufficient number and type of computer devices are available and configured to ensure that protected health information (PHI) is physically and electronically protected within and outside the healthcare setting. | Worksheet 1.1 | ○ | ○ | ○ | ○ | ○ | | Reset |
| 1.2 | Robust physical and logistical technical controls are in place to restrict access to patient-level protected health information and other organizational data, including limiting access to EHR application-level settings and system-to-system interface configurations to authorized users only. | Worksheet 1.2 | ○ | ○ | ○ | ○ | ○ | | Reset |
| 1.3 | Current versions of the EHR application software and its associated operating systems, cybersecurity protections, software applications, interface protocols, and clinical vocabularies are implemented. | Worksheet 1.3 | ○ | ○ | ○ | ○ | ○ | | Reset |
| 1.4 | The EHR uses standardized data interchange protocols and clinical and administrative terminologies for exchanging data between internal and external computer systems, adhering to national recommendations when possible. | Worksheet 1.4 | ○ | ○ | ○ | ○ | ○ | | Reset |
| 1.5 | Administrative, financial, and clinical data interchange specifications are clearly documented for data elements being received and sent via each application programming interface (API), describing how data will be used, stored, and who is responsible for maintaining each API and connected systems. | Worksheet 1.5 | ○ | ○ | ○ | ○ | ○ | | Reset |

## Recommended Practices for Domain 2 — Using Health IT Safely

| | Recommended Practice | Worksheet | 0% Not Implemented | 1- 30% Making Progress | 31- 60% Halfway There | 61- 90% Substantial Progress | 91- 100% Fully Implemented | EHR Limitation | |
|---|---|---|---|---|---|---|---|---|---|
| 2.1 | The EHR integrates data generated by Food and Drug Administration (FDA)-approved medical devices (e.g., IV pumps and physiological monitors) and FDA-approved, personal wearable devices (e.g., continuous glucose monitors, atrial fibrillation, or sleep apnea detection). | Worksheet 2.1 | ○ | ○ | ○ | ○ | ○ | | Reset |
| 2.2 | The organization maintains a separate and visually distinct EHR computing environment for live production use by clinicians along with other environments for application and content building, testing, and user training. | Worksheet 2.2 | ○ | ○ | ○ | ○ | ○ | | Reset |

## Recommended Practices for Domain 2 — Using Health IT Safely

| | Practice | Worksheet | 0% Not Implemented | 1- 30% Making Progress | 31- 60% Halfway There | 61- 90% Substantial Progress | 91- 100% Fully Implemented | EHR Limitation | |
|---|---|---|---|---|---|---|---|---|---|
| 2.3 | System hardware, operating and network software, and clinical application version updates, modifications, or local customizations are tested individually and in the context of other integrated systems using a standardized approach. This consists of:<br>▪ Testing before go-live and as installed in production to ensure adequate performance and data integrity<br>▪ Monitoring all systems for a short time following any hardware or software changes<br>▪ Notifying end users before, and reminding them after, potentially impactful changes to applications or clinical content assets | Worksheet 2.3 | ○ | ○ | ○ | ○ | ○ | | Reset |
| 2.4 | System-to-system interface testing is conducted prior to and following go-live, as well as after hardware or software updates, to ensure data integrity and sufficient transaction volume capacity. | Worksheet 2.4 | ○ | ○ | ○ | ○ | ○ | | Reset |
| 2.5 | The EHR and its components are tested prior to implementation and after major system updates or customizations to ensure the human-computer interface meets basic safety and usability requirements for different user roles, clinical contexts, and individuals. | Worksheet 2.5 | ○ | ○ | ○ | ○ | ○ | | Reset |
| 2.6 | Software and application testing is clinically authentic and relevant, based on real-world scenarios incorporating collaborative workflows, and designed to identify high-risk patient safety concerns. | Worksheet 2.6 | ○ | ○ | ○ | ○ | ○ | | Reset |

## Recommended Practices for Domain 3 — Monitoring Safety

| | Practice | Worksheet | 0% Not Implemented | 1- 30% Making Progress | 31- 60% Halfway There | 61- 90% Substantial Progress | 91- 100% Fully Implemented | EHR Limitation | |
|---|---|---|---|---|---|---|---|---|---|
| 3.1 | Key configuration and API settings are monitored to ensure they work as intended, using automated surveillance when possible. | Worksheet 3.1 | ○ | ○ | ○ | ○ | ○ | | Reset |
| 3.2 | System hardware, software, clinical applications, and any modifications or customizations are closely monitored after updates to the operating system or applications to ensure components continue to work as expected and data integrity is maintained. | Worksheet 3.2 | ○ | ○ | ○ | ○ | ○ | | Reset |

>Table of Contents | >About the Checklist | >Team Worksheet | >About the Practice Worksheets | >Practice Worksheets

Clinicians should complete this self-assessment and evaluate potential health IT-related patient safety risks addressed by this specific SAFER Guide within the context of your particular healthcare organization.

This Team Worksheet is intended to help organizations document the names and roles of the self-assessment team, as well as individual team members' activities. Typically team members will be drawn from a number of different areas within your organization, and in some instances, from external sources. The suggested Sources of Input section in each Recommended Practice Worksheet identifies the types of expertise or services to consider engaging. It may be particularly useful to engage specific clinician and other leaders with accountability for safety practices identified in this guide.

The Worksheet includes fillable boxes that allow you to document relevant information. The Assessment Team Leader box allows documentation of the person or persons responsible for ensuring that the self-assessment is completed. The section labeled Assessment Team Members enables you to record the names of individuals, departments, or other organizations that contributed to the self-assessment. The date that the self-assessment is completed can be recorded in the Assessment Completion Date section and can also serve as a reminder for periodic reassessments. The section labeled Assessment Team Notes is intended to be used, as needed, to record important considerations or conclusions arrived at through the assessment process. This section can also be used to track important factors such as pending software updates, vacant key leadership positions, resource needs, and challenges and barriers to completing the self-assessment or implementing the Recommended Practices in this SAFER Guide.

Assessment Team Leader

Assessment Completion Date

Assessment Team Members

Assessment Team Notes

Reset

Each *Recommended Practice Worksheet* provides guidance on implementing a specific *Recommended Practice*, and allows you to enter and print information about your self-assessment.

The *Suggested Sources of Input* section indicates categories of personnel who can provide information to help evaluate your level of implementation.

The *Rationale* section provides guidance about "why" the safety activities are needed.

Strength of Recommendation section provides an estimate of the strength of evidence available in the scientific literature, or states that it is "required" due to a federal rule, regulation, or conditions of participation, for each recommendation.

**Recommended Practice- Disaster Recovery Plans**

**1.1** Disaster recovery plans must be in place and reviewed at least annually, for computing and networking infrastructure that runs applications critical to the organization's clinical and administrative operations, including hardware duplication, network redundancy, and data replication.
Checklist

**Implementation Status**

EHR Limitation

**Rationale for Practice or Risk Assessment**

Organizations should take steps to prevent and minimize the impact of technology failures.[6] A single point of failure, whether it be a database server, a connection to the Internet, or data backup tapes stored in racks adjacent to the production servers, greatly increases risks for loss of data availability and integrity.

**Suggested Sources of Input**

1. Clinicians, support staff, and/or clinical administration
2. EHR developer
3. Health IT support staff (in-house or external)

**Strength of Recommendation**

Required

**Implementation Guidance**

- A large healthcare organization that provides care 24 hours per day has a remotely located (i.e., > 50 miles away and > 20 miles from the coastline) "warm-site" (i.e., a site with current patient data that can be activated in less than 8 hours) backup facility that can run the entire EHR.[7]
- The backup computer system (e.g., warm-site) is tested at least quarterly.[8]
- The organization maintains a redundant path to the Internet consisting of two different cables in different trenches[6]
(Note: a microwave or other form of wireless connection is also acceptable), provided by two different Internet providers.)[9,10]
- Smaller ambulatory clinics have at least a cellphone-based, wireless Internet access point that is capable of running a cloud-hosted EHR as a backup to their main cable-based Internet connection.

Assessment Notes

Enter any notes about your self-assessment.

Follow-up Actions

Enter any follow-up activities required.

Person Responsible for Follow-up Action

Enter the name of the person responsible for the follow-up activities.

Reset

The Implementation Guidance section lists potentially useful practices or scenarios to inform your assessment and implementation of the specific Recommended Practice.

## Recommended Practice - Adequate and Privacy-Protected EHR Access Points

**1.1** A sufficient number and type of computer devices are available and configured to ensure that protected health information (PHI) is physically and electronically protected within and outside the healthcare setting.

*Checklist*

### Implementation Status

### EHR Limitation

### Rationale for Practice or Risk Assessment

Rapid, reliable clinical information and EHR access by all authorized users while protecting patient privacy is essential for safe and effective care. A sufficient number of fixed and mobile devices (e.g., desktop workstations, portable laptops, tablets, smartphones, and other electronic displays) should be available to clinical staff to support an individual's data entry and ability to review tasks, team communication, and efficient clinical and administrative workflows. In addition, these devices must be configured to ensure that Protected Health Information (PHI) remains inaccessible and out of view of unauthorized individuals regardless of the type of device or the location in which it is used to access patient information.[1,2,3] This includes appropriate security during transmission of data between devices.

| Suggested Sources of Input | Strength of Recommendation |
|---|---|
| 1. Clinicians, support staff, and clinical administration<br>2. Health IT support staff | Required |

### Implementation Guidance

- The organization has a mobile device management policy determining allowable functionality and access.[4]
- Consideration is given to mobile devices and other electronic sources of data input and storage (e.g., infusion pumps, and automated medication dispensing cabinets).
- Devices used to access patient data are positioned with screens facing away from publicly accessible locations or have privacy screens restricting viewing at angles.
- Tracking dashboards or electronic patient lists in public areas do not display full patient names (e.g., first initial and first three letters of the last name).

Assessment Notes

Follow-up Actions

Person Responsible for Follow-up Action

Reset

## Recommended Practice - EHR access point protections

**1.2** Robust physical and logistical technical controls are in place to restrict access to patient-level protected health information and other organizational data, including limiting access to EHR application-level settings and system-to-system interface configurations to authorized users only.[4]

*Checklist*

**Implementation Status**

**EHR Limitation**

### Rationale for Practice or Risk Assessment

Physical and logistical security procedures, including rigorous authentication processes and specific role-based permissions, are necessary to control access to patient data and system-level EHR configurations. The extent of technical security and authentication solutions should be commensurate with the levels of the importance of the data and risk of data breach (e.g., strong passwords for patient data access, multi-factor authentication, and restricted permissions for remote access to patient data and system-level privileges, firewall-protected networks, and physically secured data servers)

Assessment Notes

Follow-up Actions

Person Responsible for Follow-up Action

Reset

### Suggested Sources of Input

1. Health IT support staff

### Strength of Recommendation

Required

### Implementation Guidance

- EHR mobile device connection is restricted to the healthcare system's secured internal Wi-Fi or via virtual private network (VPN) for remote access.[5]
- The organization conducts regular assessments to define, implement, and monitor user authentication and track hardware inventory including personal devices used to access the EHR.[6]
- The organization has a policy describing privacy and security precautions required when accessing the EHR from outside the organization (e.g., two-factor authentication), including but not limited to tasks such as documentation, chart review, telehealth encounters, order and result management (e.g., work should be performed in a private location not visible by family or other unauthorized individuals).
- Multifactor authentication processes (e.g., a mix of strong passwords, smart ID cards, 2-factor authentication applications, or biometric data) are used to restrict access to information systems, data, and system configuration functionality.[7]
- EHR access devices have enabled automatic screen locking after a non-modifiable appropriate time (e.g., 2-minutes in public areas).
- User roles with different data input and review capabilities are defined for clinical and non-clinical users based on education, training, and job function, with specific features and functions assigned to each role. Within each of these groups, subcategories of users are defined with very specific capabilities (e.g., only prescribing MDs, DOs, or NPs can order Schedule 2 medications without a co-signature, and only credentialed IT staff can access or manipulate data servers and system-level settings).[8]
- Employees who change jobs within the organization are reassigned to the appropriate level of EHR access and capability, and employee login credentials are revoked as soon as employment with the organization ends.
- Supervisors periodically review and re-authorize (or revoke) clinical and administrative staff roles and associated EHR authorizations to access various clinical systems, functions, and data.
- EHR access devices are physically secured at all times, including in the healthcare setting or when in the possession of the user.[6]
- Users are trained to report lost or stolen EHR access devices immediately, and the organization can wipe data remotely.[6]

## Recommended Practice - Current Versions

**1.3** Current versions of the EHR application software and its associated operating systems, cybersecurity protections, software applications, interface protocols, and clinical vocabularies are implemented.

*Checklist*

### Implementation Status

### EHR Limitation

### Rationale for Practice or Risk Assessment

Maintaining outdated versions of clinical software and associated components can result in missing updates and enhancements intended to mitigate patient safety risks, correct software design flaws, improve efficiency and user experience, or support interoperability.

### Suggested Sources of Input

1. EHR Vendor
2. Health IT Staff

### Strength of Recommendation

Medium

### Implementation Guidance

- A configuration and patch management process exists to enable maintenance of IT assets and the relationship between different components.[9]
- The organization has a process ensuring advanced preparation and timely implementation of both regular and off-cycle updates to the EHR version, clinical vocabularies, drug databases, and other recurring functionality and content releases.[6]
- The organization provides role-specific user training about enhancements and other changes impacting their workflows and data.[3]
- The organization performs a risk-benefit analysis prior to delaying or declining updates to system hardware and software components and communicates the resulting findings and rationale to key stakeholders.
- Patient safety remediations introduced in a new EHR version should be available for enablement, if possible, in previous versions of the EHR (i.e., backward compatible) without requiring implementation of the updated version in its entirety.

Assessment Notes

Follow-up Actions

Person Responsible for Follow-up Action

Reset

## Recommended Practice - Standardized Data Exchange

**1.4** The EHR uses standardized data interchange protocols and clinical and administrative terminologies for exchanging data between internal and external computer systems, adhering to national recommendations when possible.

*Checklist*

**Implementation Status**

**EHR Limitation**

### Rationale for Practice or Risk Assessment

Standards, such as HL7 Fast Healthcare Interoperability Resources (FHIR), and clinical terminologies, such as the Systematized Nomenclature of Medicine (SNOMED), promote consistency and semantic interoperability to ensure safe, secure, and effective data transmission between EHRs and external systems including Health Information Exchanges (HIEs), medical devices, and third-party applications. Aligning with current standards and terms provides a foundation for future development while reducing data exchange errors.[11]

Assessment Notes

Follow-up Actions

Person Responsible for Follow-up Action

Reset

### Suggested Sources of Input

1. EHR Vendor
2. Health IT Staff

### Strength of Recommendation

Required

### Implementation Guidance

- The EHR has implemented the recommended versions of standards and data elements supporting interoperability (e.g., United States Core Data for Interoperability [USCDI], Fast Healthcare Interoperability Resources [FHIR], Health Level 7 [HL7] Consolidated Clinical Document Architecture [C-CDA], provider identification numbers, and others).
- Clinical applications and interfaces allow for regular and timely updates of clinical code sets (e.g., SNOMED, LOINC, ICD-10) to assure data integrity and protect against distortion or loss.[12]
- Digital health information (e.g., medications, allergies, and problems) is stored and exchanged using standard terminologies.[13]
- The organization has established a data governance group to administer standards and maintain a data dictionary within and across systems.
- Variation from established standards occurs only when necessary, and data or format localizations and other custom software development are documented and maintained to avoid confusion or loss of historical knowledge. The organization consistently works to eliminate or reduce these local variations.

## Recommended Practice - API Specifications

**1.5** Administrative, financial, and clinical data interchange specifications are clearly documented for data elements being received and sent via each application programming interface (API), describing how data will be used, stored, and who is responsible for maintaining each API and connected systems.

Checklist

### Implementation Status

### EHR Limitation

### Rationale for Practice or Risk Assessment

Thorough documentation of the technical specifications and management responsibilities for APIs clarifies requirements of data elements for each interface and ensures accountability for any issues that arise.

Assessment Notes

Follow-up Actions

Person Responsible for Follow-up Action

Reset

### Suggested Sources of Input

1. EHR Vendor
2. Health IT staff

### Strength of Recommendation

Medium

### Implementation Guidance

- API quality controls are in place to ensure high-quality usable data.
- The organization has a policy specifying what can be written into the EHR with or without further evaluation or validation (e.g., providers must approve clinical data from outside sources before it can be added to the local database).[14]
- All types of data to be exchanged via the interface are clearly specified including: allowable values (e.g., text vs. numeric, length or size of fields); clinical vocabularies used; and how associated values (i.e., metadata) will be communicated (e.g., representation of units on measurements including reference ranges, sources of data, clinical High/Low flags).
- APIs exchanging orders and other critical clinical information include accurate status messaging, including cancellation and acknowledgment details.[15]
- The interface is monitored to assess usage, and designed to handle the estimated mean and maximum amounts of data expected to cross the interface with acceptable performance.
- The interface's error log is monitored and errors are investigated and fixed in a timely manner.
- The organization maintains a comprehensive data dictionary that includes, for each data element:
  - Data type (e.g., coded, text, numeric)
  - Size of data field (e.g., number of free-text characters or size of integer or real numbers)
  - Data Definition
  - Metadata (e.g., creator, date created, users)
- The organization maintains a comprehensive interface data map that includes data recodes or conversions, as required.
- The organization maintains a set of system-to-system interface performance requirements including the expected throughput of the system, uptime requirements, and protocols supported.

## Recommended Practice - Device Integration

**2.1** The EHR integrates data generated by Food and Drug Administration (FDA)-approved medical devices (e.g., IV pumps and physiological monitors) and FDA-approved, personal wearable devices (e.g., continuous glucose monitors, atrial fibrillation, or sleep apnea detection).

Checklist

**Implementation Status**

**EHR Limitation**

### Rationale for Practice or Risk Assessment

Data generated from medical devices in the healthcare setting and some approved personal devices should flow to the EHR effectively and efficiently. Patient-generated health data (PGHD) from devices within and outside the healthcare setting is increasingly utilized in clinical specialties and in primary care practices[16]. Providers may access longitudinal PGHD for detailed health status information for a variety of conditions requiring occasional or continuous physiologic monitoring. Efficient and effective analysis of large volumes of patient-generated data requires streamlined automated integration into the EHR to achieve full benefit without overburdening clinicians.

Assessment Notes

Follow-up Actions

Person Responsible for Follow-up Action

Reset

### Suggested Sources of Input

1. EHR Vendor
2. Health IT staff
3. Clinicians

**Strength of Recommendation**

Medium

### Implementation Guidance

- The clinical user interface is designed to display PGHD without overwhelming the clinician.[17]
- A process exists to validate that physiological monitoring data is aligned with the correct patient and frequency of interface transmission.
- Advanced analytics and/or AI machine learning are leveraged to identify patterns and predict potential problems.[18]
- Data most important to providers in the clinical decision making process is prioritized in the integration of PGHD to the EHR.[19]
- The organization has governance, protocols, and processes for the approval and use of structured PGHD entered into the EHR.[20,21]
- The system has sufficient capacity to receive and store increasing volumes of patient data.
- PGHD data is encrypted during transmission and storage.
- Only data from FDA-approved medical or personal physiologic monitoring devices should be routinely integrated into EHRs.[22]

## Recommended Practice - Separate Environments

**2.2** The organization maintains a separate and visually distinct EHR computing environment for live production use by clinicians along with other environments for application and content building, testing, and user training.
Checklist

### Implementation Status

### EHR Limitation

### Rationale for Practice or Risk Assessment

Development of new EHR features, functions, and content, exploratory testing, and user training should take place in realistically configured environments that are clearly distinguishable from the live production version. However, comprehensive end-to-end, integrated testing should also be performed using test patients in the live version with appropriate controls and safeguards in place to ensure key features, applications, configurations, and interfaces are available and functional.[23]

Assessment Notes

Follow-up Actions

Person Responsible for Follow-up Action

Reset

### Suggested Sources of Input

1. EHR Vendor
2. Health IT Staff

### Strength of Recommendation

Medium

### Implementation Guidance

- The EHR has a visually distinct environment including the functionality necessary for basic user training, with de-identified patients and real-world data, including up to date clinical decision support and bidirectional workflow capability (e.g., diagnostic ordering and result review).
- The EHR has a separate environment for testing clinical applications, interfaces, version, and vocabulary updates with enabled functionality to validate end-to-end ordering and other data transmissions as well as quality measure performance.
- The EHR is designed to make it difficult to confuse the live production version with the training, testing, or backup read-only versions (e.g., different icons for access and different background colors, or other visually distinct features to highlight the version's identity).
- The organization has policies and protocols for testing end-to-end functionality in the production environment including the use of test patients.[23]
- The ability to create test patients in the production environment is tightly controlled and restricted to specific users with special permissions
- The organization maintains up-to-date EHR environment management documentation that clearly describes any differences between the production version and the environments used for testing and training. This documentation includes the environment name, EHR version, interface versions, and other ancillary application versions along with its update frequency.
- Enhancements are developed in a build environment, tested in a test environment, and then enabled in the training environment before being moved to the production environment for final testing and use.
- The organization has a policy and process for creating and naming test patients in the production environment, and that policy requires names that cannot be confused with genuine patients (e.g., BWH73Test or ZZZZtest).

## Recommended Practice - Testing at Go-Live, Updates

**2.3** System hardware, operating and network software, and clinical application version updates, modifications,or local customizations are tested individually and in the context of other integrated systems using a standardized approach. This consists of:

Testing before go-live and as installed in production to ensure adequate performance and data integrity

Monitoring all systems for a short time following any hardware or software changes

Notifying end users before, and reminded them after, potentially impactful changes to applications or clinical content assets.

Checklist

### Implementation Status

### EHR Limitation

### Rationale for Practice or Risk Assessment

Failure to adequately test system hardware, software, and configuration or customization of clinical applications can lead to data integrity issues and impede response time, reliability, and error-free operation.

### Suggested Sources of Input

1. Health IT support staff
2. EHR Vendor

### Strength of Recommendation

Medium

Assessment Notes

Follow-up Actions

Person Responsible for Follow-up Action

### Implementation Guidance

- Software enhancements and updates are installed and tested in a test environment prior to moving into the production environment.
- New versions of the EHR system are enabled in a test environment with functionality sufficient for end-to-end testing of multidisciplinary workflows prior to release in the live/ production environment.
- Customizations made by the organization, department, or user are tested to ensure they do not adversely impact other aspects of the system or interoperability with internal or external systems.[24]
- Simulation testing is conducted for clinical processes such as order entry, pharmacy review, nurse notification, medication fill, medication administration, and multidisciplinary clinical documentation to ensure that the application addresses the organization’s needs.
- Data migration processes and protocols are in place to ensure data integrity after transmitting data from one EHR system to another, changing the format of data (e.g., free text to structured), and clinical code updates (e.g., SNOMED, ICD-10, LOINC).
- Users are provided with a concise, relevant summary of software or component updates that impact their workflows or the data they rely on.

Reset

## Recommended Practice - Interface Testing

**2.4** System-to-system interface testing is conducted prior to and following go-live, as well as after hardware or software updates, to ensure data integrity and sufficient transaction volume capacity.

Checklist

### Implementation Status

### EHR Limitation

### Rationale for Practice or Risk Assessment

Interface configuration validation and testing confirming the reliability of coded and free text data, as well as links to content resources and other information, is necessary on both sides of the interface to ensure the reliability and integrity of transmitted data elements. Additionally, it is important to verify that the interfaces are able to handle the transaction load without slowdown or corruption of data integrity.[14]

Assessment Notes

Follow-up Actions

Person Responsible for Follow-up Action

Reset

### Suggested Sources of Input

1. EHR Vendor
2. Health IT staff

### Strength of Recommendation

Medium

### Implementation Guidance Suggestions

- The intensity and extent of interface testing are consistent with the technical complexity of the interface, the potential for patient harm, and the importance of the accuracy and timeliness of the data that traverses the interface.
- API test plans include high-risk examples identified by the organization during safety monitoring or event reviews.
- Validate that the API can transmit relevant metadata to provide the context necessary for data interpretation (e.g., measurement value with units of measurement).
- Users are clearly notified if the data they enter is not transmissible across the interface (e.g., free text exceeding character limit or task or order is canceled).
- Special attention is paid to the accuracy of clinical content transmission.
- API testing is performed for conversion programs, changes within databases, vocabularies, or other significant data or processing elements.[15]
- Changes are clearly communicated to impacted users relying on interface data after any upgrades or system-level modifications.
- There is a policy describing API configuration controls including required notifications before changes are made and specifying who is authorized to make, approve, and test the changes prior to implementation.

## Recommended Practice - Testing Usability

**2.5** The EHR and its components are tested prior to implementation and after major system updates or customizations to ensure the human-computer interface meets basic safety and usability requirements for different user roles, clinical contexts, and individuals.

*Checklist*

### Implementation Status

### EHR Limitation

### Rationale for Practice or Risk Assessment

Adequately supporting differences in user interface preferences and varying requirements for different specialties, locations, roles, and devices can improve system safety and effectiveness.

Assessment Notes

Follow-up Actions

Person Responsible for Follow-up Action

Reset

### Suggested Sources of Input

1. EHR developer
2. Health IT staff

### Strength of Recommendation

Required

### Implementation Guidance Suggestions

- Testing is performed on the most commonly used devices, including but not limited to desktops, laptops, workstations on wheels, tablets, smartphones, and patient-facing devices to ensure consistency in visual displays and interactions.
- The EHR user interface is evaluated after system or department level customizations to ensure no adverse impact on usability and safety.[25]
- Font size is adequate to allow users to clearly distinguish alphanumeric data.[26]
- Color coding is accompanied by redundant information (e.g. labels, bold, or italics) to accommodate users with color vision deficiency.[26]
- Relevant contextual data (e.g., recent laboratory test results when ordering medications that may affect that laboratory value) are displayed when needed or useful without requiring the user to navigate out of context.[27]
- Major CDS and CPOE changes and interventions are tested with representative end users.[28]

## Recommended Practice - Testing Scenarios

**2.6** Software and application testing is clinically authentic and relevant, based on real-world scenarios incorporating collaborative workflows, and designed to identify high-risk patient safety concerns.

*Checklist*

### Implementation Status

**EHR Limitation**

### Rationale for Practice or Risk Assessment

Robust, realistic, and dynamic test plans represent end-to-end multidisciplinary clinical workflows while incorporating known high-risk areas (e.g., medication management, diagnostic decision-making, patient identification). The complexity of clinical care should be addressed by including authentic actions (e.g., tapered medication dosing and administration, appropriate clinical decision support) while also using implausible data and testing at the limits of functionality.

Assessment Notes

Follow-up Actions

Person Responsible for Follow-up Action

Reset

### Suggested Sources of Input

1. Health IT staff
2. EHR Vendor
3. Clinicians and other end users

### Strength of Recommendation

Medium

### Implementation Guidance Suggestions

- When EHR developers conduct clinical application testing on behalf of the health care organization, end users at the health care organization have an opportunity to review the developer's test plan for relevance and appropriateness.
- Clinical interactions and unique workflows that may cause serious harm in addition to low-risk, more frequent tasks are included in test plans.[29]
- The organization has created a comprehensive test plan that validates the performance of each major function, including screen appearance, the graphic representation of data, alerts, and the accurate generation of reports.[30]
- Clinical and other staff most familiar with patient care, financial management, and other administrative processes have an opportunity to review test plans for comprehensiveness and relevance to their specific roles and responsibilities.
- Testing is coordinated and considers various user groups.
- Test plans are dynamic, and updated at regular intervals to incorporate new risks identified by the organization during safety monitoring or adverse event review.
- The organization has a test plan governance policy specifying the indications for and frequency of test plan review and required authorization for changes to test plans.

## Recommended Practice - Monitoring System Safety

**3.1** Key configuration and API settings are monitored to ensure they work as intended, using automated surveillance when possible.[30]

Checklist

### Implementation Status

### EHR Limitation

### Rationale for Practice or Risk Assessment

Monitoring the performance of key clinical components (e.g., system performance, interface transaction volumes, error logs) can help identify technical issues that may lead to serious safety events. Whenever feasible, automated surveillance should be utilized to detect issues rapidly.

Assessment Notes

Follow-up Actions

Person Responsible for Follow-up Action

Reset

### Suggested Sources of Input

1. EHR Vendor
2. Health IT support staff

### Strength of Recommendation

Medium

### Implementation Guidance Suggestions

- Proactive monitoring of external services (e.g., providers of CDS content) is enabled for the rapid detection of malfunctions in the production environment.[31]
- When a vendor performs monitoring on behalf of the healthcare organization, there is an established process to ensure rapid notification of any changes in operation or potential patient safety issues to the healthcare organization.
- High-risk error logs (e.g., order entry and referral queues) are continuously monitored and issues are promptly resolved.
- Real-time surveillance of interface transaction volumes is in place to rapidly identify and investigate significant changes.
- When available, automated audit log monitoring is enabled.[32]
- The organization has policies and procedures that identify and define the key configuration settings and the individuals or teams responsible for monitoring them. Key settings may include the system response time, EHR database server capacity, password strength, and system timeouts.
- The organization has a method of automatically monitoring (e.g., by periodically checking) all internet-based links presented within the EHR.
- System response time is measured and reported regularly.

## Recommended Practice - Monitoring Safety After Updates

**3.2** System hardware, software, clinical applications, and any modifications or customizations are closely monitored after updates to the operating system or applications to ensure components continue to work as expected and data integrity is maintained.

*Checklist*

### Implementation Status

### EHR Limitation

### Rationale for Practice or Risk Assessment

Monitoring the performance of system components after updates can ensure rapid identification, mitigation, and communication of potential problems.

### Suggested Sources of Input

1. Health IT support staff
2. EHR Vendor

### Strength of Recommendation

Medium

### Implementation Guidance Suggestions

- System and software application performance and safety are monitored for a period of time in the production environment after the introduction of new enhancements or updates.[33]
- Interfaces between key clinical applications (e.g., CPOE and pharmacy, laboratory, and EHR) are continuously monitored to detect errors.
- Patient portal documents, reports, and other components viewed by patients are monitored after core system changes to ensure clinical information is accurately rendered.
- Standard, regularly used clinical and administrative reports (e.g., length of stay, readmission rates, alert override rates) are generated and reviewed periodically to ensure that the data on which they are based has not changed in a way that renders the report meaningless.

Assessment Notes

Follow-up Actions

Person Responsible for Follow-up Action

Reset

## References

1. Kraushaar J, Bohnet-Joschko S. Prevalence and patterns of mobile device usage among physicians in clinical practice: A systematic review. Health Informatics J. 2023;29(2):14604582231169296. pubmed.ncbi.nlm.nih.gov/37063054/. doi: 10.1177/14604582231169296; PMID: 37063054.

2. Martin G, Khajuria A, Arora S, King D, Ashrafian H, Darzi A. The impact of mobile technology on teamwork and communication in hospitals: A systematic review. J Am Med Inform Assoc. 2019;26(4):339–355. pubmed.ncbi.nlm.nih.gov/30689893/. doi: 10.1093/jamia/ocy175; PMID: 30689893; PMC7647195.

3. Soegaard Ballester JM, Bass GD, Urbani R, et al. A mobile, electronic health record-connected application for managing team workflows in inpatient care. Appl Clin Inform. 2021;12(5):1120–1134. https://pubmed.ncbi.nlm.nih.gov/34937103/. doi: 10.1055/s-0041-1740256; PMID: 34937103; PMC8695057.

4. Office of the National Coordinator for Health Information Technology (ONC). Managing mobile devices in your health care organization. https://www.healthit.gov/sites/default/files/fact-sheet-managing-mobile-devices-in-your-health-care-organization.pdf. Accessed Jun 12, 2024.

5. HIPAA. Electronic code of federal regulations. HIPAA privacy rule. title 45, part 164.310. https://www.ecfr.gov/current/title-45/part-164. Updated 2024. Accessed Jun 26, 2024.

6. U.S. Department of Health and Human Services, Health Sector Cybersecurity Coordination Center (HC3), Office of Information Security. HC3: HPH mobile device security checklist, Report: 202303231700. https://www.hhs.gov/sites/default/files/hph-mobile-device-security-checklist-tlpclear.pdf. Updated 2023. Accessed Jun 12, 2024.

7. Office for Civil Rights. June 2023 OCR cybersecurity newsletter. US Department of Health and Human Services. 2023. https://www.hhs.gov/hipaa/for-professionals/security/guidance/cybersecurity-newsletter-june-2023/index.html. Accessed Jun 12, 2024.

8. Ahlness EA, Orlander J, Brunner J, et al. "Everything's so role-specific": VA employee perspectives' on electronic health record (EHR) transition implications for roles and responsibilities. J Gen Intern Med. 2023;38(Suppl 4):991–998. https://pubmed.ncbi.nlm.nih.gov/37798577/. doi: 10.1007/s11606-023-08282-5; PMID: 37798577; PMC10593626.

9. Argaw ST, Troncoso-Pastoriza JR, Lacey D, et al. Cybersecurity of hospitals: Discussing the challenges and working towards mitigating the risks. BMC Med Inform Decis Mak. 2020;20(1):146. https://pubmed.ncbi.nlm.nih.gov/32620167/. doi: 10.1186/s12911-020-01161-7; PMID: 32620167; PMC7333281.

10. Alotaibi YK, Federico F. The impact of health information technology on patient safety. Saudi Med J. 2017;38(12):1173–1180. https://pubmed.ncbi.nlm.nih.gov/29209664/. doi: 10.15537/smj.2017.12.20631. PMID: 29209664; PMC5787626.

11. Barker W, Maisel N, Strawley CE, Israelit GK, Adler-Milstein J, Rosner B. A national survey of digital health company experiences with electronic health record application programming interfaces. J Am Med Inform Assoc. 2024;31(4):866–874. https://pubmed.ncbi.nlm.nih.gov/38281124/. doi: 10.1093/jamia/ocae006; PMID: 38281124; PMC10990546.

12. Huang C, Koppel R, McGreevey JD, Craven CK, Schreiber R. Transitions from one electronic health record to another: Challenges, pitfalls, and recommendations. Appl Clin Inform. 2020;11(5):742–754. https://pubmed.ncbi.nlm.nih.gov/33176389/. doi: 10.1055/s-0040-1718535; PMID: 33176389; PMC7657707.

13. Health and Human Services. Notice of publication of the trusted exchange framework and common agreement. Federal Register, the daily journal of United States government. 2022. https://www.federalregister.gov/documents/2022/01/19/2022-00948/notice-of-publication-of-the-trusted-exchange-framework-and-common-agreement. Accessed Jun 23, 2024.

14. Dullabh P, Hovey L, Heaney-Huls K, Rajendran N, Wright A, Sittig DF. Application programming interfaces in health care: Findings from a current-state sociotechnical assessment. Appl Clin Inform. 2020;11(1):59–69. https://pubmed.ncbi.nlm.nih.gov/31968383/. doi: 10.1055/s-0039-1701001; PMID: 31968383; PMC6976305.

15. Schreiber R, Sittig DF, Ash J, Wright A. Orders on file but no labs drawn: Investigation of machine and human errors caused by an interface idiosyncrasy. J Am Med Inform Assoc. 2017;24(5):958–963. https://pubmed.ncbi.nlm.nih.gov/28339629/. doi: 10.1093/jamia/ocw188; PMID: 28339629; PMC6080845.

## References

16. Kompala T, Wong J, Neinstein A. Diabetes specialists value continuous glucose monitoring despite challenges in prescribing and data review process. J Diabetes Sci Technol. 2023;17(5):1265–1273. https://pubmed.ncbi.nlm.nih.gov/35403469/. doi: 10.1177/19322968221088267; PMID: 35403469; PMC10563522.

17. Shenvi E, Boxwala A, Sittig D, et al. Visualization of patient-generated health data: A scoping review of dashboard designs. Appl Clin Inform. 2023;14(5):913–922. https://pubmed.ncbi.nlm.nih.gov/37704021/. doi: 10.1055/a-2174-7820; PMID: 37704021; PMC10665122.

18. Khatiwada P, Yang B, Lin J, Blobel B. Patient-generated health data (PGHD): Understanding, requirements, challenges, and existing techniques for data security and privacy. J Pers Med. 2024;14(3):282. https://pubmed.ncbi.nlm.nih.gov/38541024/. doi: 10.3390/jpm14030282; PMID: 38541024; PMC10971637.

19. Tiase VL, Sward KA, Del Fiol G, Staes C, Weir C, Cummins MR. Patient-generated health data in pediatric asthma: Exploratory study of providers' information needs. JMIR Pediatr Parent. 2021;4(1):e25413. https://pubmed.ncbi.nlm.nih.gov/33496674/. doi: 10.2196/25413; PMID: 33496674; PMC8414476.

20. Shaw RJ, Boazak M, Tiase V, et al. Integrating patient-generated digital health data into electronic health records (EHRs) in ambulatory care settings: EHR vendor survey and interviews. AMIA Jt Summits Transl Sci Proc. 2022;2022:439–445. https://www.ncbi.nlm.nih.gov/pmc/articles/PMC9285170/; PMID: 35854713; PMCID: PMC9285170.

21. Sittig DF, Boxwala A, Wright A, et al. Patient-centered clinical decision support challenges and opportunities identified from workflow execution models. J Am Med Inform Assoc. 2024:ocae138. https://pubmed.ncbi.nlm.nih.gov/38907738/. doi: 10.1093/jamia/ocae138; PMID: 38907738; PMC11258405.

22. Food and Drug Administration Safety Communication. Do not use smartwatches or smart rings to measure blood glucose levels: FDA safety communication. 2024. https://www.fda.gov/medical-devices/safety-communications/do-not-use-smartwatches-or-smart-rings-measure-blood-glucose-levels-fda-safety-communication. Accessed July 29, 2024.

23. Wright A, Aaron S, Sittig DF. Testing electronic health records in the “production” environment: An essential step in the journey to a safe and effective health care system. Journal of the American Medical Informatics Association. 2017;24(1):188–192; PMID: 27107450; PMC5201179.

24. Tutty MA, Carlasare LE, Lloyd S, Sinsky CA. The complex case of EHRs: Examining the factors impacting the EHR user experience. J Am Med Inform Assoc. 2019;26(7):673–677. https://pubmed.ncbi.nlm.nih.gov/30938754/. doi: 10.1093/jamia/ocz021; PMID: 30938754; PMC6562154.

25. Hettinger AZ, Melnick ER, Ratwani RM. Advancing electronic health record vendor usability maturity: Progress and next steps. J Am Med Inform Assoc. 2021;28(5):1029–1031. https://pubmed.ncbi.nlm.nih.gov/33517394/. doi: 10.1093/jamia/ocaa329; PMID: 33517394; PMC8068416.

26. Pruitt ZM, Howe JL, Bocknek LS, et al. Informing visual display design of electronic health records: A human factors cross-industry perspective”. Patient Safety. 2023;5(2):i–xiii. https://patientsafetyj.com/article/77769-informing-visual-display-design-of-electronic-health-records-a-human-factors-cross-industry-perspective.

27. Senathirajah Y, Kaufman DR, Cato KD, Borycki EM, Fawcett JA, Kushniruk AW. Characterizing and visualizing display and task fragmentation in the electronic health record: Mixed methods design. JMIR Hum Factors. 2020;7(4):e18484. https://pubmed.ncbi.nlm.nih.gov/33084580/. doi: 10.2196/18484; PMID: 33084580; PMC7641790.

28. Kawamoto K, McDonald CJ. Designing, conducting, and reporting clinical decision support studies: Recommendations and call to action. Ann Intern Med. 2020;172(11 Suppl):S101–S109. https://pubmed.ncbi.nlm.nih.gov/32479177/. doi: 10.7326/M19-0875; PMID: 32479177.

29. Pew Charitable Trusts. Ways to improve electronic health record safety. 2018. https://www.pewtrusts.org/en/research-and-analysis/reports/2018/08/28/ways-to-improve-electronic-health-record-safety.

30. Aguirre RR, Suarez O, Fuentes M, Sanchez-Gonzalez MA. Electronic health record implementation: A review of resources and toolshttps://Pubmed.ncbi.nlm.nih.gov/31700751/. Cureus. 2019;11(9):e5649. pubmed.ncbi.nlm.nih.gov/31700751/. doi: 10.7759/cureus.5649; PMID: 31700751; PMC6822893.

## References

31. Wright A, Hickman TT, McEvoy D, et al. Analysis of clinical decision support system malfunctions: A case series and survey. J Am Med Inform Assoc. 2016;23(6):1068–1076. https://pubmed.ncbi.nlm.nih.gov/27026616/. doi: 10.1093/jamia/ocw005; PMID: 27026616; PMC5070518.

32. Adler-Milstein J, Adelman JS, Tai-Seale M, Patel VL, Dymek C. EHR audit logs: A new goldmine for health services research? J Biomed Inform. 2020;101:103343. https://pubmed.ncbi.nlm.nih.gov/31821887/. doi: 10.1016/j.jbi.2019.103343; PMID: 31821887.

33. Singh H, Wilson L, Petersen LA, et al. Improving follow-up of abnormal cancer screens using electronic health records: Trust but verify test result communication. BMC Med Inform Decis Mak. 2009;9:49. https://pubmed.ncbi.nlm.nih.gov/20003236/. doi: 10.1186/1472-6947-9-49; PMID: 20003236; PMC2797509.
