# Supplementary material for: Revisions to the Safety Assurance Factors for Electronic Health Record Resilience (SAFER) Guides to update national recommendations for safe use of electronic health records
Source: J Am Med Inform Assoc. 2025 Apr 12;32(4):755–60. doi: 10.1093/jamia/ocaf018 (PMC12005625; doi:10.1093/jamia/ocaf018)
Supplement: ocaf018_Supplementary_Data [file ocaf018_supplementary_data.zip › ocaf018_Supplementary_Data/Test Results Reporting Final.pdf]

Self-Assessment

Test Results Reporting and Follow-Up

General Instructions for the SAFER Self-Assessment Guides

The Safety Assurance Factors for EHR Resilience (SAFER) guides are designed to help healthcare organizations conduct proactive self-assessments to evaluate the safety and effectiveness of their electronic health record (EHR) implementations. The 2025 SAFER guides have been updated and streamlined to focus on the highest risk, most commonly occurring issues that can be addressed through technology or practice changes to build system resilience in the following areas:

- Organizational Responsibilities
- Patient Identification
- Clinician Communication
- Test Results Reporting and Follow-up
- Computerized Provider Order Entry with Decision Support
- Systems Management
- Contingency Planning
- High Priority Practices - A collection of 16 Recommendations from the other 7 Guides

Each of the eight SAFER Guides begins with a Checklist of recommended practices. The downloadable SAFER Guides provide fillable circles that can be used to indicate the extent to which each recommended practice has been implemented in the organization using a 5-point Likert scale. The Practice Worksheet gives a rationale for the practice and provides examples of how to implement each recommended practice. It contains fields to record team member involvement and follow-up actions based on the assessment. The Worksheet also lists the stakeholders who can provide input to assess each practice (sources of input). In addition to the downloadable version, the content of each SAFER Guide, with interactive references and supporting materials, can also be viewed on ONC's website at: https://www.healthit.gov/topic/safety/safer-guides.

The SAFER guides are based on the best available (2024) evidence from the literature and consensus expert opinion. Subject matter experts in patient safety, informatics, quality improvement, risk management, human factors engineering, and usability developed them. Furthermore, they were reviewed by an external group of practicing clinicians, informaticians, and information technology professionals.

Each guide contains between 6 and 18 recommended practices including its rationale, implementation guidance, and evidence level. The recommended practices in the SAFER Guides are intended to be useful for all EHR users. However, every organization faces unique circumstances and may implement a particular recommended practice differently. As a result, some of the specific implementation guidance in the SAFER Guides for recommended practices may not be applicable to an organization.

The High Priority Practices guide consists of 16 of the most important and relevant recommendations selected from the other 7 guides. It is designed for practicing clinicians to help them understand, implement, and support EHR safety and safe use within their organization. The other seven guides consist of 88 unique recommendations that are relevant for all healthcare providers and organizations.

The SAFER Guides are designed in part to help deal with safety concerns created by the continuously changing sociotechnical landscape that healthcare organizations face. Therefore, changes in technology, clinical practice standards, regulations, and policy should be taken into account when using the SAFER Guides. Periodic self-assessments using the SAFER Guides may also help organizations identify areas where it is particularly important to address the implications of these practice or EHR-based changes for the safety and safe use of EHRs. Ultimately, the goal is to improve the overall safety of our health care system and improve patient outcomes.

The SAFER Guides are not intended to be used for legal compliance purposes, and implementation of a recommended practice does not guarantee compliance with the HIPAA Security or Privacy Rules, Medicare or Medicaid Conditions of Participation, or any other laws or regulations. The SAFER Guides are for informational purposes only and are not intended to be an exhaustive or definitive source. They do not constitute legal advice. Users of the SAFER Guides are encouraged to consult with their own legal counsel regarding compliance with Medicare or Medicaid program requirements, and any other laws.

For additional information on Medicare and Medicaid program requirements, please visit the Centers for Medicare & Medicaid Services website at www.cms.gov. For more information on HIPAA, please visit the HHS Office for Civil Rights website at www.hhs.gov/ocr.

Self-Assessment

Test Results Reporting and Follow-Up

Introduction

The Test Results Reporting and Follow-Up SAFER Guide identifies recommended safety practices intended to optimize the safety and safe use of processes and EHR technology for the electronic communication and management of test results. Processes relating to test results are vulnerable to breakdowns, requiring careful planning, implementation, and maintenance to deliver correct information promptly to the intended recipients.[1] In the EHR-enabled healthcare environment, clinicians rely on technology to support and manage the reporting and follow-up of test results. This guide enables the assessment of EHR-based communication of test results. It provides guidance on recommended practices to ensure that an EHR's design and implementation help close the loop on test results to minimize the potential for errors and delays.[2-9]

EHRs can potentially improve test result reporting and follow-up if implemented and used correctly. Initial evaluation of the use of health IT for test results reporting and follow-up has produced mixed results.[4,5,10,11] Furthermore, laboratory and radiology/imaging results reporting in EHRs remain vulnerable to safety events.[12] Failure to follow up appropriately on diagnostic test results can lead to misdiagnosis, patient harm, and liability.

Completing the self-assessment requires the engagement of people both within and outside the organization (e.g., EHR technology developers, and diagnostic services providers). Clinician leadership in the organization should be engaged in assessing whether and how any particular recommended practice affects the organization's ability to deliver safe, high-quality care.

Collaboration between clinicians and staff members while completing the self-assessment in this guide will enable an accurate snapshot of the organization's EHR status in terms of test results reporting. In addition, it should lead to a consensus about the organization's future path to optimize EHR-related safety and quality: setting priorities among the recommended practices not yet addressed, ensuring a plan is in place to maintain recommended practices already in place, dedicating the required resources to make necessary improvements, and working together to mitigate the test results-related safety risks introduced by the EHR.

Self-Assessment

Test Results Reporting and Follow-Up

Table of Contents

General Instructions	1
Introduction	2
About the Checklist	5
Checklist	6
Team Worksheet	8
About the Recommended Practice Worksheets	9
Recommended Practice Worksheets	10
References	28

Authors and Peer Reviewers

The SAFER Self-Assessment Guides were developed by health IT safety researchers and informatics experts whose contributions are acknowledged as follows:

Primary authors who contributed to the development of all guides:

Trisha Flanagan, RN, MSN, CPPS, Health Informatics Nurse, Center for Innovations in Quality, Effectiveness and Safety, Michael E. DeBakey Veterans Affairs Medical Center, Houston TX

Hardeep Singh, MD, MPH, Co-Chief, Health Policy, Quality and Informatics Program, Center for Innovations in Quality, Effectiveness and Safety and Professor of Medicine at the Michael E. DeBakey Veterans Affairs Medical Center and Baylor College of Medicine, Houston, TX

Dean F. Sittig MS, PhD, FACMI, FAMIA, FHIMSS, FIAHSI, Professor of Biomedical Informatics, Department of Clinical and Health Sciences, McWilliams School of Biomedical Informatics, University of Texas Health Science Center at Houston, TX and Informatics Review LLC, Lake Oswego, OR

Support staff for the primary authorship team

Rosann Cholankeril, MD, MPH, Center for Innovations in Quality, Effectiveness and Safety, Michael E. DeBakey Veterans Affairs Medical Center and Baylor College of Medicine

Sara Ehsan, MBBS, MPH, Center for Innovations in Quality, Effectiveness and Safety, Michael E. DeBakey Veterans Affairs Medical Center and Baylor College of Medicine

Additional authors who contributed to at least one guide:

Jason S. Adelman, MD, MS, (Patient ID) Chief Patient Safety Officer & Associate Chief Quality Officer, Executive Director, Patient Safety Research, Co-Director, Patient Safety Research Fellowship in Hospital Medicine, New York-Presbyterian Hospital/Columbia University Irving Medical Center, New York, NY

Daniel R. Murphy, MD, MBA, (Clinician Communication, Test Results) Chief Quality Officer, Baylor Medicine, Houston, TX

Patricia Sengstack, DNP, NI-BC, FAAN, FACMI, (Organizational Responsibilities) Senior Associate Dean for Informatics, Director, Nursing Informatics Specialty Program, Vanderbilt University School of Nursing, Vanderbilt University, Nashville, TN

Additional contributors who provided feedback on various guides or parts of guides

Miriam Callahan, MD (Patient ID)
David C. Classen, MD (CPOE, AI recommendation)
Anne Grauer, MD, MS (Patient ID)
Ing Haviland (Patient ID)
Amanda Heidemann, MD (All Guides)
I-Fong Sun Lehman, DrPH, MS (Patient ID)
Christoph U. Lehmann, MD (AI recommendation)
Christopher A. Longhurst, MD, MS (AI recommendation)
Edward R. Melnick, MD (Clinician Communication)
Robert E. Murphy, MD (Organizational Responsibilities)
Ryan P. Radecki, MD, MS (AI recommendation)
Raj Ratwani, PhD (AI recommendation)
Trent Rosenbloom, MD (Clinician Communication)
Lisa Rotenstein, MD (Clinician Communication)
Hojjat Salmasian, MD, PhD (All Guides)
Richard Schreiber, MD (CPOE)
Danny Sands, MD (Clinician Communication)
Debora Simmons, PhD, RN (Organizational Responsibilities)
Carina Sirochinsky (Patient ID)
Neha Thummala, MPH (Patient ID)
Emma Weatherford (Patient ID)
Adam Wright, PhD (CPOE)
Andrew Zimolzak, MD, MMSc (Test Results, Clinician Communication)

This guide was developed under the contract Unintended Consequences of Health IT and Health Information Exchange, Task Order HHSP23337003T/HHSP23320095655WC.

The ATSP/ONC composite mark is a mark of the U.S. Department of Health and Human Services. The contents of the publication or project are solely the responsibility of the authors and do not necessarily represent the official views of the U.S. Department of Health and Human Services, Asssistant Secretary for the Technology Policy/Office of the National Coordinator for Health Information Technology.

>Table of Contents | >About the Checklist | >Team Worksheet | >About the Practice Worksheets | >Practice Worksheets

The *Checklist* is structured as a quick way to enter and print your self-assessment.

Select the level of implementation achieved by your organization for each Recommended Practice. Your Implementation Status will be reflected on the Recommended Practice Worksheet in this PDF. The implementation status scales are as followed:

Not Implemented – (0%) The organization has not implemented this recommendation.

Making Progress (1 - 30%) The organization is in the early or pilot phase of implementing this recommendation as evidenced by following or adopting less than 30% of the implementation guidance

Halfway there (31 – 60%) The organization is implementing this recommendation and is following or has adopted approximately half of the implementation guidance.

Substantial Progress (61-90%) The organization has nearly implemented this recommendation and is following or has adopted much of the implementation guidance.

Fully Implemented (91-100%) The organization follows this recommendation, and most implementation guidance is followed consistently and widely adopted.

The organization should check the following box if there are some limitations with the current version of their EHR that preclude them from fully implementing this recommendation.

EHR Limitation - The EHR does not offer the features/functionality required to fully implement this recommendation or the implementation guidance.

The *Domain* associated with the *Recommended Practice(s)* appears at the top of the column

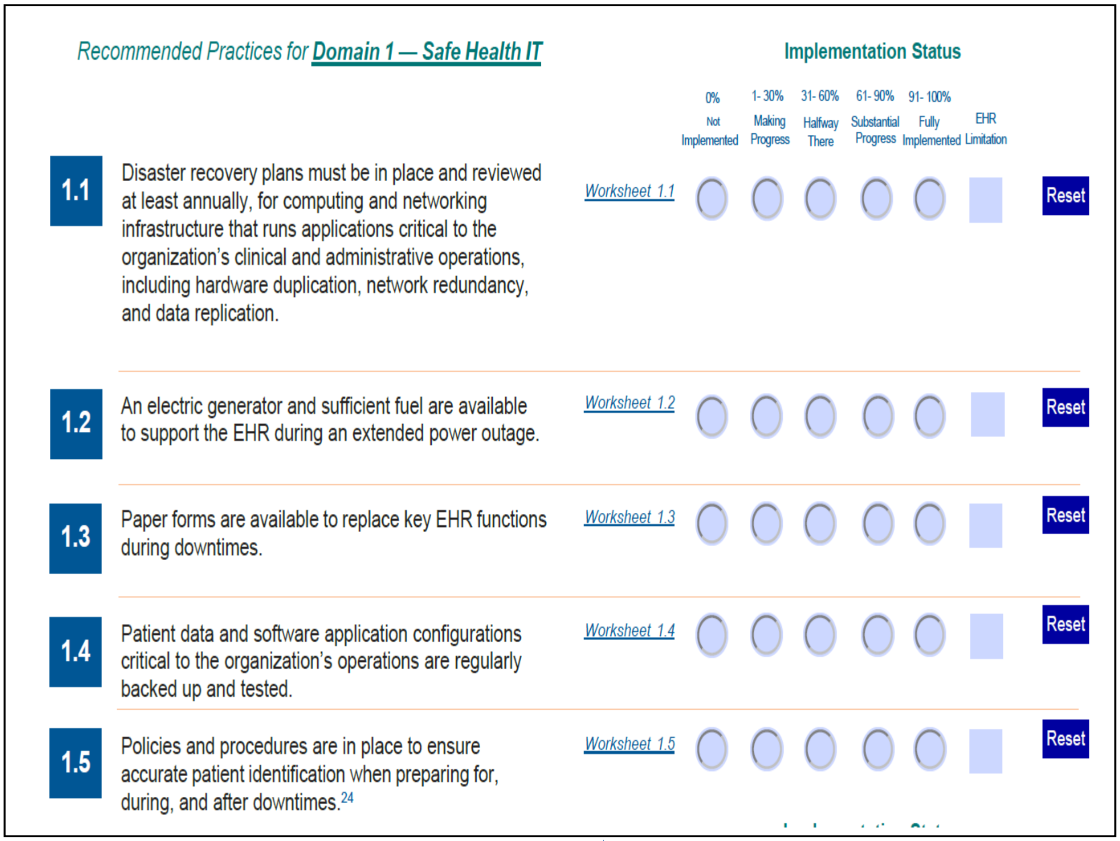
Recommended Practices for Domain 1 — Safe Health IT

	Recommended Practice		Implementation Status: 0% Not Implemented	1-30% Making Progress	31-60% Halfway There	61-90% Substantial Progress	91-100% Fully Implemented	EHR Limitation	
1.1	Disaster recovery plans must be in place and reviewed at least annually, for computing and networking infrastructure that runs applications critical to the organization's clinical and administrative operations, including hardware duplication, network redundancy, and data replication.	Worksheet 1.1							Reset
1.2	An electric generator and sufficient fuel are available to support the EHR during an extended power outage.	Worksheet 1.2							Reset
1.3	Paper forms are available to replace key EHR functions during downtimes.	Worksheet 1.3							Reset
1.4	Patient data and software application configurations critical to the organization's operations are regularly backed up and tested.	Worksheet 1.4							Reset
1.5	Policies and procedures are in place to ensure accurate patient identification when preparing for, during, and after downtimes.[24]	Worksheet 1.5							Reset

The *Recommended Practice(s)* for the topic appears below the associated *Domain*.

To the right of reach *Recommended Practice* is a link to the Recommended Practice Worksheet in this PDF.

The *Worksheet* provides guidance on implementing the practice.

*Recommended Practices for **Domain 1 — Safe Health IT***

	Practice	Worksheet	Implementation Status: 0% Not Implemented	1- 30% Making Progress	31- 60% Halfway There	61- 90% Substantial Progress	91- 100% Fully Implemented	EHR Limitation	
1.1	Test names, values, and interpretations (i.e., outside of normal reference ranges) for laboratory results are stored in the EHR as structured data using standardized nomenclature.[5,9,13-17]	Worksheet 1.1	○	○	○	○	○		Reset
1.2	Predominantly text-based test reports (e.g., radiology or pathology reports) are coded by the interpreting clinician as abnormal/normal at a minimum.[20-24]	Worksheet 1.2	○	○	○	○	○		Reset

*Recommended Practices for **Domain 2 — Using Health IT Safely***

	Practice	Worksheet	Implementation Status: 0% Not Implemented	1- 30% Making Progress	31- 60% Halfway There	61- 90% Substantial Progress	91- 100% Fully Implemented	EHR Limitation	
2.1	The EHR is able to track the status of all test-related orders and procedures associated with them (e.g., specimen received and collected; test completed, reported, and acknowledged).[10,29]	Worksheet 2.1	○	○	○	○	○		Reset
2.2	The ordering clinician is identifiable on all ordered tests and test reports, and if another clinician is responsible for follow-up, that clinician is also identified in the EHR.[6]	Worksheet 2.2	○	○	○	○	○		Reset
2.3	When test results are changed or amended, the ordering clinician and other clinicians responsible for follow-up are notified electronically, and the changed results and amended flag should be clearly visible in the EHR.[32] For clinically significant changes, the clinicians are also contacted directly.[43]	Worksheet 2.3	○	○	○	○	○		Reset
2.4	Written policies specify unambiguous responsibility for test result follow-up with a shared understanding of that responsibility among all involved in providing follow-up care.[5,7,10,13,14,34,37,46-48]	Worksheet 2.4	○	○	○	○	○		Reset
2.5	Workflows that are particularly vulnerable to mishandling of test results, especially critical test results,[33] are identified,[53] and fail-safe procedures ensure these results are received by someone responsible for the affected patient's care.[5,43,54]	Worksheet 2.5	○	○	○	○	○		Reset
2.6	Results outside normal reference ranges or otherwise determined to be abnormal are flagged (i.e., presented in a visually distinct way).[5,7]	Worksheet 2.6	○	○	○	○	○		Reset
2.7	Display of results (e.g., numeric, text, graphical, image) should be easily accessible, clearly visible, not easily overlooked, and understandable.[65]	Worksheet 2.7	○	○	○	○	○		Reset

Recommended Practices for Domain 2 — Using Health IT Safely

	Recommended Practice	Worksheet	0% Not Implemented	1-30% Making Progress	31-60% Halfway There	61-90% Substantial Progress	91-100% Fully Implemented	EHR Limitation	
2.8	There is an EHR-based process for clinicians to either assign surrogates[5,6,58,67] for receiving test result notifications or enables surrogates to access the principal clinicians inboxes.	Worksheet 2.8							Reset
2.9	There are mechanisms to forward results and results notifications from one clinician to another.[9,46]	Worksheet 2.9							Reset
2.10	Summarization tools to trend and graph laboratory data are available in the EHR.[70]	Worksheet 2.10							Reset
2.11	Test results can be sorted, or filtered, in the clinician s EHR inbox according to clinically relevant criteria (e.g. test collection date/time, result date/time, severity, hospital location, patient).[5,9,43,47]	Worksheet 2.11							Reset
2.12	The EHR has the capability for clinicians to set reminders for themselves and other responsible clinical staff for future tasks to facilitate test result follow-up.[47,75]	Worksheet 2.12							Reset

Recommended Practices for Domain 3 — Monitoring Safety

	Recommended Practice	Worksheet	0% Not Implemented	1-30% Making Progress	31-60% Halfway There	61-90% Substantial Progress	91-100% Fully Implemented	EHR Limitation	
3.1	As part of quality assurance activities, organizations monitor selected practices or indicators[78] related to test result reporting and follow-up. Monitored practices include clinician acknowledgment of test results and clinician follow-up on abnormal test results.[4,5,10,13,37,43,58,79-81]	Worksheet 3.1							Reset
3.2	As part of quality assurance, the organization monitors and addresses test results sent to the wrong clinician (e.g., via reports from clinicians) or never transmitted to any clinician (e.g., due to an interface problem or patient/provider misidentification).[37,84]	Worksheet 3.2							Reset
3.3	As part of quality assurance, the organization monitors clinical decision support tools that are based on laboratory test results to ensure they are updated when changes are made to the laboratory system or the way laboratory data is recorded.[85,86]	Worksheet 3.3							Reset
3.4	Organizational policies and procedures ensure timely patient notification of both normal and abnormal test results, and the timeliness of notification is monitored.[88]	Worksheet 3.4							Reset

Clinicians should complete this self-assessment and evaluate potential health IT-related patient safety risks addressed by this specific SAFER Guide within the context of your particular healthcare organization.

This Team Worksheet is intended to help organizations document the names and roles of the self-assessment team, as well as individual team members' activities. Typically, team members will be drawn from a number of different areas within your organization, and in some instances, from external sources. The suggested Sources of Input section in each Recommended Practice Worksheet identifies the types of expertise or services to consider engaging. It may be particularly useful to engage specific clinician and other leaders with accountability for safety practices identified in this guide.

The Worksheet includes fillable boxes that allow you to document relevant information. The Assessment Team Leader box allows documentation of the person or persons responsible for ensuring that the self-assessment is completed. The section labeled Assessment Team Members enables you to record the names of individuals, departments, or other organizations that contributed to the self-assessment. The date that the self-assessment is completed can be recorded in the Assessment Completion Date section and can also serve as a reminder for periodic reassessments. The section labeled Assessment Team Notes is intended to be used, as needed, to record important considerations or conclusions arrived at through the assessment process. This section can also be used to track important factors such as pending software updates, vacant key leadership positions, resource needs, and challenges and barriers to completing the self-assessment or implementing the Recommended Practices in this SAFER Guide.

Assessment Team Leader

Assessment Completion Date

Assessment Team Members

Assessment Team Notes

Reset

Each *Recommended Practice Worksheet* provides guidance on implementing a specific *Recommended Practice*, and allows you to enter and print information about your self-assessment.

The *Suggested Sources of Input* section indicates categories of personnel who can provide information to help evaluate your level of implementation.

The *Rationale* section provides guidance about "why" the safety activities are needed.

Strength of Recommendation section provides an estimate of the strength of evidence available in the scientific literature, or states that it is "required" due to a federal rule, regulation, or conditions of participation, for each recommendation.

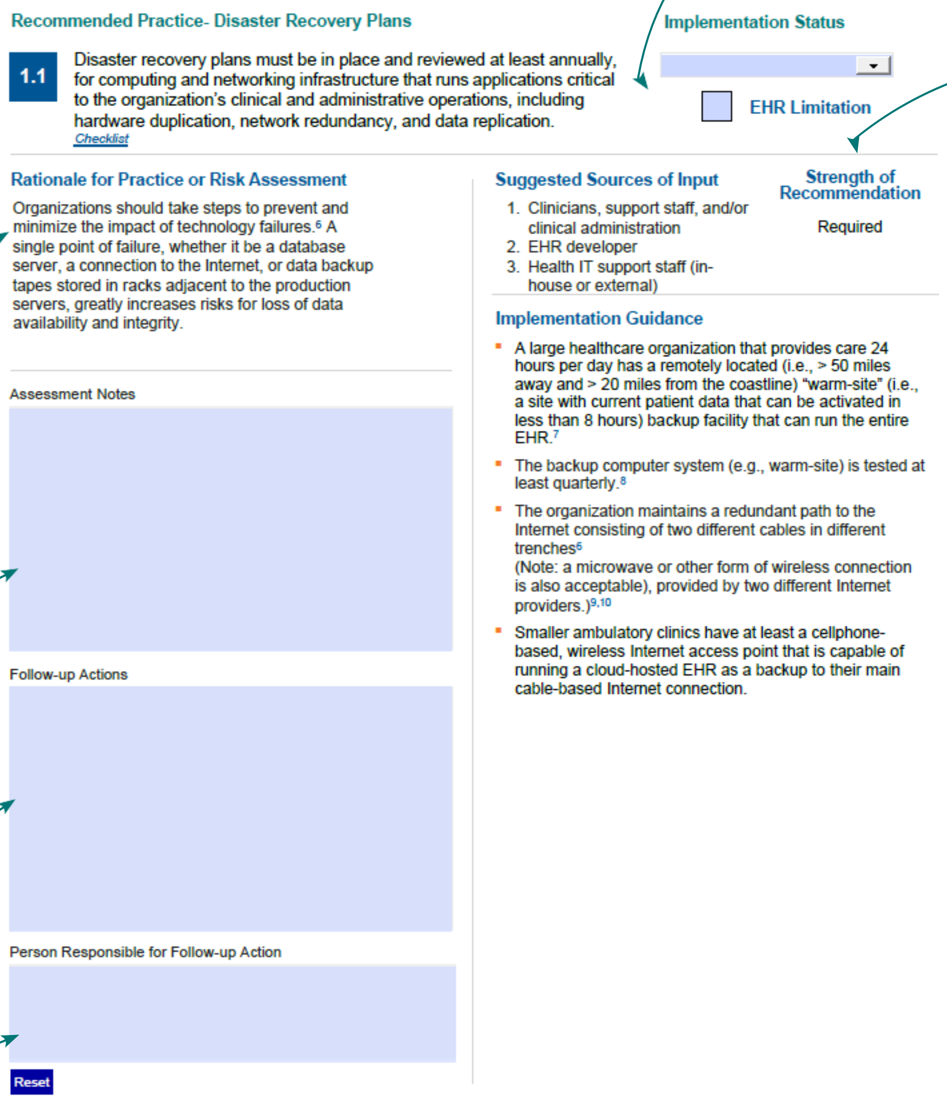
Recommended Practice- Disaster Recovery Plans

1.1 Disaster recovery plans must be in place and reviewed at least annually, for computing and networking infrastructure that runs applications critical to the organization's clinical and administrative operations, including hardware duplication, network redundancy, and data replication.
Checklist

Implementation Status

EHR Limitation

Rationale for Practice or Risk Assessment

Organizations should take steps to prevent and minimize the impact of technology failures.[6] A single point of failure, whether it be a database server, a connection to the Internet, or data backup tapes stored in racks adjacent to the production servers, greatly increases risks for loss of data availability and integrity.

Suggested Sources of Input

1. Clinicians, support staff, and/or clinical administration
2. EHR developer
3. Health IT support staff (in-house or external)

Strength of Recommendation

Required

Implementation Guidance

- A large healthcare organization that provides care 24 hours per day has a remotely located (i.e., > 50 miles away and > 20 miles from the coastline) "warm-site" (i.e., a site with current patient data that can be activated in less than 8 hours) backup facility that can run the entire EHR.[7]
- The backup computer system (e.g., warm-site) is tested at least quarterly.[8]
- The organization maintains a redundant path to the Internet consisting of two different cables in different trenches[6] (Note: a microwave or other form of wireless connection is also acceptable), provided by two different Internet providers.)[9,10]
- Smaller ambulatory clinics have at least a cellphone-based, wireless Internet access point that is capable of running a cloud-hosted EHR as a backup to their main cable-based Internet connection.

Assessment Notes

Follow-up Actions

Person Responsible for Follow-up Action

Reset

Enter any notes about your self-assessment.

Enter any follow-up activities required.

Enter the name of the person responsible for the follow-up activities.

The Implementation Guidance section lists potentially useful practices or scenarios to inform your assessment and implementation of the specific Recommended Practice.

Recommended Practice- Structured Test Names

1.1 Test names, values, and interpretations (i.e., outside of normal reference ranges) for laboratory results are stored in the EHR as structured data using standardized nomenclature.[5,9,13-17]

Implementation Status

Fully Implemented

EHR Limitation

Rationale for Practice or Risk Assessment

Structured laboratory results facilitate EHR-based result reporting and tracking functions.[10] Structured data enables the use of clinical decision support (CDS) that can avoid errors and optimize patient safety.

Assessment Notes

Follow-up Actions

Person Responsible for Follow-up Action

Reset

Suggested Sources of Input

1. Diagnostic services
2. EHR developer
3. Health IT support staff

Strength of Recommendation

Medium

Implementation Guidance

- Test result names (e.g., sodium, potassium) that are sent along with LOINC codes are stored as coded data.[18]
- Abnormal test result values and interpretations are defined and stored in a standardized, coded format (e.g., high/low sodium, critical potassium, positive/negative fecal occult blood test).[7,19]
- There is a process to handle paper-based test results that includes, at a minimum, the entry of coded values into the EHR to indicate Test Result Name, Test Result Value, Units, Normal Range, Abnormal Flag, and Date/Time, along with a scanned copy of the report in the EHR.

Recommended Practice- Code Results of Text-Based Reports

1.2 Predominantly text-based test reports (e.g., radiology or pathology reports) are coded by the interpreting clinician as abnormal/normal at a minimum.[20-24]

Checklist

Implementation Status

EHR Limitation

Rationale for Practice or Risk Assessment

Coded results in structured fields facilitate EHR-based result reporting and tracking functions.[10]

Suggested Sources of Input

1. Diagnostic services
2. EHR developer
3. Health IT support staff

Strength of Recommendation

Medium

Implementation Guidance

- Abnormal test result values and interpretations are defined and stored in a standardized format.
- Mammography results are stored according to BI-RADS® criteria.[25,26]
- The organization considers using standardized reporting criteria for selected imaging tests where such standards exist, for instance, the Lung Rads for lung cancer screening CT reporting[27] and the TI-RADS criteria to code thyroid image reporting.[28]

Assessment Notes

Follow-up Actions

Person Responsible for Follow-up Action

Reset

Recommended Practice- Track Test Orders

2.1 The EHR is able to track the status of all test-related orders and procedures associated with them (e.g., specimen received and collected; test completed, reported, and acknowledged).[10,29]

Checklist

Implementation Status

EHR Limitation

Rationale for Practice or Risk Assessment

Order tracking facilitates closed-loop communication.[30] This enables the detection of problems related to order processing and test result delivery.

Suggested Sources of Input

1. Diagnostic services
2. EHR vendor
3. Health IT support staff

Strength of Recommendation

Medium

Implementation Guidance

- The EHR can record, display, and report whether orders were received, specimens collected, tests completed, results reported, and results acknowledged.[31-38]
- The EHR facilitates the tracking of "send-out" tests at the point of ordering and provides a mechanism to allow clinicians or organizations to incorporate these results into the EHR and assign them to the correct patient.[39]
- Procedures exist to ensure that all test results, including those received from outside the organization through fax or mail, are properly incorporated into the EHR.[40]
- Clinical practices where test result information is not fully integrated into the EHR use additional tracking strategies to enable follow-up.[38]

Assessment Notes

Follow-up Actions

Person Responsible for Follow-up Action

Reset

> Table of Contents | > About the Checklist | > Team Worksheet | > About the Practice Worksheets | >Practice Worksheets

Recommended Practice- Ordering Clinical Identifiable

2.2 The ordering clinician is identifiable on all ordered tests and test reports, and if another clinician is responsible for follow-up, that clinician is also identified in the EHR.[6]

Checklist

Implementation Status

EHR Limitation

Rationale for Practice or Risk Assessment

Clear identification of the ordering clinician facilitates closed-loop communication. Ambiguous responsibility increases the risk of follow-up failure.[10]

Assessment Notes

Follow-up Actions

Person Responsible for Follow-up Action

Reset

Suggested Sources of Input

1. Clinicians, support staff, and/or clinical administration
2. EHR developer
3. Health IT support staff

Strength of Recommendation

Medium

Implementation Guidance

- Result routing systems support the delivery of results to the ordering clinician.[4,7,9,37]
- The EHR supports assignment or transfer of responsibility for test order follow-up.[37,41]
- Policies and procedures address situations vulnerable to follow-up failures, including shift hand-offs, clinician rotation off-service, transitions of care settings, and when clinicians are out of the office or have departed the organization.
- There are escalation processes for high-priority or urgent test results that are not responded to by clinicians within a pre-specified time period, including an alternate communication method.[42]
- When a user other than the ordering clinician enters an order under the clinicians name (e.g., per-protocol ordering), the entering user's name is visible on the order information.

Recommended Practice- Amended test Results

2.3 When test results are changed or amended, the ordering clinician and other clinicians responsible for follow-up are notified electronically, and the changed results and amended flag should be clearly visible in the EHR.[32] For clinically significant changes, the clinicians are also contacted directly.[43]

Checklist

Implementation Status

EHR Limitation

Rationale for Practice or Risk Assessment

Results that are subsequently changed carry a significant potential for delayed or wrong treatment based on outdated, incorrect results.

Suggested Sources of Input

1. Clinicians, support staff, and/or clinical administration
2. Diagnostic services
3. EHR developer
4. Health IT support staff

Strength of Recommendation

Medium

Implementation Guidance

- The individual changing the results is responsible for notifying appropriate clinicians of those changes. Electronic systems may not always ensure that critical communications are received and reviewed promptly. Thus, for clinically important changes to results, appropriate clinicians should be contacted directly.[7]
- Policies and procedures ensure that changes in test results and accompanying documentation are effectively communicated to the appropriate clinicians responsible for patient care, including after the patient has transitioned to another setting of care.[44,45]
- Changed results are clearly flagged as such in the EHR (e.g., marked as “amended”).[7]

Assessment Notes

Follow-up Actions

Person Responsible for Follow-up Action

Reset

Recommended Practice - Responsibility for Result Follow-Up

2.4 Written policies specify unambiguous responsibility for test result follow-up with a shared understanding of that responsibility among all involved in providing follow-up care.[5,7,10,13,14,34,37,46-48]

Checklist

Implementation Status

EHR Limitation

Rationale for Practice or Risk Assessment

New workflows resulting from the introduction of EHRs can introduce new hazards related to miscommunication of responsibility for follow-up. Ambiguous responsibility increases the risk of follow-up failure.[49,50]

Suggested Sources of Input

1. Clinicians, support staff, and/or clinical administration
2. Diagnostic services

Strength of Recommendation

Medium

Implementation Guidance

- In the outpatient setting, the ordering clinician is responsible for follow-up unless he or she delegates this responsibility (e.g., to a covering clinician). Delegation should be documented in the EHR and accepted by the delegate.[51,52]
- In organizations with trainees (e.g., residents or fellows), ultimate responsibility defaults to the supervising attending in the event of a change of service by the trainee acting as an ordering clinician.
- Ordering clinicians in any setting assume responsibility for follow-up care, unless that responsibility is unambiguously transferred to another clinician who accepts responsibility.[37]

Assessment Notes

Follow-up Actions

Person Responsible for Follow-up Action

Reset

> Table of Contents | > About the Checklist | > Team Worksheet | > About the Practice Worksheets | >Practice Worksheets

Recommended Practice- Critical Test Results

2.5 Workflows that are particularly vulnerable to mishandling of test results, especially critical test results,[33] are identified,[53] and fail-safe procedures ensure these results are received by someone responsible for the affected patient's care.[5,43,54]

Checklist

Implementation Status

EHR Limitation

Rationale for Practice or Risk Assessment

Lost or mishandled test results, especially critical results, are a significant risk to patient safety, especially in situations where workflows are particularly vulnerable to such failures (e.g., shift changes, transitions of care).[55]

Suggested Sources of Input

1. Clinicians, support staff, and/or clinical administration
2. Diagnostic services
3. EHR developer
4. Health IT support staff

Strength of Recommendation

Required

Implementation Guidance

- Situations that are vulnerable to test results follow-up failures are identified.[57-59] These include handoffs between clinicians (e.g., between residents, part-time physicians, ER physicians, and hospitalists),[55] care transitions[15,60,61] between clinical settings (e.g., between different units of a hospital; between the hospital and home or a post-acute facility), and tests pending at discharge. In these situations, processes should be in place to ensure that test results are communicated to a clinician responsible for follow-up care.[51]
- Life-threatening results are communicated verbally or electronically with rapid acknowledgment and automatic escalation if there is no response to ensure confirmation of receipt.[7,62] The fact that these notifications occurred is also documented in the legal medical record (including information on who performed the notification, who was notified, the contents of the message, and the date and time notification occurred).
- Notifications of abnormal test results that remain unacknowledged after a pre-specified time period are forwarded (or escalated) to an alternate responsible provider.[37,63]
- Diagnostic services should ensure that test results are communicated to a back-up provider in a timely fashion if the ordering provider is not available. The necessary timeliness is dependent on the significance of the test result.[64]
- The organization maintains an updated contact list of all practicing clinicians, and this list includes their coverage schedules.[4,37]
- The organization maintains a patient-provider link (e.g., the patient s PCP is identified) in the EHR as a back-up. If the ordering provider does not acknowledge the result, a responsible clinician in the ordering practice is notified.

Assessment Notes

Follow-up Actions

Person Responsible for Follow-up Action

Reset

Recommended Practice- Abnormal Results Flagged

2.6 Results outside normal reference ranges or otherwise determined to be abnormal are flagged (i.e., presented in a visually distinct way).[5,7]
Checklist

Implementation Status

EHR Limitation

Rationale for Practice or Risk Assessment

Although the absence of flags does not necessarily mean the result is normal, flagging can reduce the likelihood of missing abnormal or critical results.

Suggested Sources of Input

1. Diagnostic services
2. EHR developer
3. Health IT support staff

Strength of Recommendation

Medium

Implementation Guidance

- Abnormal results are flagged (e.g., bolded font, asterisk beside values, use of “H” or “L,” different colors) or marked for better visualization in the EHR.
- Color is not used as the only visual indicator of clinical significance.[65]
- Critical values are flagged in a distinct way from simply abnormal values.

Assessment Notes

Follow-up Actions

Person Responsible for Follow-up Action

Reset

Recommended Practice- Clear Display of Results

2.7 Display of results (e.g., numeric, text, graphical, image) should be easily accessible, clearly visible, not easily overlooked, and understandable.[65]

Checklist

Implementation Status

EHR Limitation

Rationale for Practice or Risk Assessment

Missed or misunderstood test results due to a poorly designed human-computer interface are as dangerous to patients as lost or inaccurate results. Results visualization and display should maximize safety to ensure critical information is not missed.

Suggested Sources of Input

1. Diagnostic services
2. EHR developer
3. Health IT support staff

Strength of Recommendation

Medium

Implementation Guidance

- Displays of test results undergo usability testing for the intended clinical users.
- Information is displayed in columns that are sufficiently wide to allow review of all pertinent information (i.e., clinicians do not need to drag columns on the user interface to detect abnormalities).[9]
- Multicomponent results are reported together (e.g., lupus anticoagulant has 2-3 subcomponents that may be individually positive or negative but should be reported together).
- Result details are reported on one screen, eliminating the need for horizontal scrolling. For example, clinicians should not have to use additional scrolling (e.g., on the "next page") to access critical information.[5,9]
- The most recent test results should, by default, be displayed first (e.g., at the top of a row-based display or at the left side on a columnar display) to ensure that clinicians are always aware of current data.[66]

Assessment Notes

Follow-up Actions

Person Responsible for Follow-up Action

Reset

Recommended Practice- Surrogates for Results

2.8 There is an EHR-based process for clinicians to either assign surrogates for receiving test result notifications or enable surrogates to access the principal clinician's inboxes.[5,6,58,67]

Checklist

Implementation Status

EHR Limitation

Rationale for Practice or Risk Assessment

If clinicians cannot assign coverage for their inbox messages when they are unavailable, this increases the risk of delays in following up on test results. Availability and use of EHR surrogate features enable coverage of test result inbox messages by a backup or alternate clinician.

Suggested Sources of Input

1. Clinicians, support staff, and/or clinical administration
2. EHR developer
3. Health IT support staff

Strength of Recommendation

Medium

Implementation Guidance

- If clinicians plan to be away, they assign a covering clinician to whom the system can automatically forward test results or notify senders that they are unavailable and another provider is covering.
- The organization has policies and procedures that establish expectations for timely review of test results and specifically address planned and unplanned absences.

Assessment Notes

Follow-up Actions

Person Responsible for Follow-up Action

Reset

> Table of Contents | > About the Checklist | > Team Worksheet | > About the Practice Worksheets | >Practice Worksheets

Recommended Practice- Forwarding Test Results

2.9 There are mechanisms to forward results and results notifications from one clinician to another.[9,46]

Checklist

Implementation Status

EHR Limitation

Rationale for Practice or Risk Assessment

Notifications are sometimes sent to incorrect clinicians, and this functionality allows clinicians to forward them to the correct person.

Suggested Sources of Input

1. Clinicians, support staff, and/or clinical administration
2. EHR developer
3. Health IT support staff

Strength of Recommendation

Medium

Implementation Guidance

- In addition to automatic forwarding - such as when a clinician is on vacation or when a patient has transferred care to another provider- a clinician can forward results manually for a specific notification (e.g., when the notification was transmitted to that clinician incorrectly).
- Mechanisms are in place for tracking acknowledgment and acceptance of forwarded notifications.
- “Close the loop” processes exist to notify safety teams of incidental findings identified in radiology tests to ensure the proper follow-up occurs in a timely manner.[68,69]

Assessment Notes

Follow-up Actions

Person Responsible for Follow-up Action

Reset

Recommended Practice- Graph Laboratory Data

2.10 Summarization tools to trend and graph laboratory data are available in the EHR.[70]
Checklist

Implementation Status

EHR Limitation

Rationale for Practice or Risk Assessment

Displaying certain laboratory test results over time helps identify clinically relevant anomalies or trends. Summarization tools in the EHR improve visualization, interpretation, and accessibility of results.

Suggested Sources of Input

1. EHR developer
2. Health IT support staff

Strength of Recommendation

Medium

Assessment Notes

Follow-up Actions

Person Responsible for Follow-up Action

Reset

Implementation Guidance

- The EHR incorporates tools and reports that enable selected laboratory results to be graphed and displayed to view trends over time. The associated graphs follow standardized display criteria.[70-72]
- The EHR includes logic to enable clinicians to identify laboratory tests by criteria other than name (such as LOINC) so they can be grouped regardless of performing entity or codified test name (e.g., point of care blood glucose testing along with glucose tests conducted in the laboratory).
- The EHR should also be able to alert clinicians to the presence of test results that may not be included in the longitudinal display due to being performed by a different entity or under a different name.
- The patient portal offers test results to patients, along with tools to support summarization and graphical display of laboratory test result data.[32,73]

Recommended Practice- Sort Test Results

2.11 Test results can be sorted, or filtered, in the clinician s EHR inbox according to clinically relevant criteria (e.g. test collection date/time, result date/time, severity, hospital location, patient).[5,9,43,47]

Checklist

Implementation Status

EHR Limitation

Rationale for Practice or Risk Assessment

Clinicians need ways to prioritize results review so that they can address the most pressing issues first and cope with information overload.[74] Sorting also improves visualization and accessibility of results.

Suggested Sources of Input

1. EHR developer
2. Health IT support staff

Strength of Recommendation

Medium

Implementation Guidance

- Results can be sorted according to important parameters (e.g., ordering provider, date, type, read/ unread, urgency, patient, location).

Assessment Notes

Follow-up Actions

Person Responsible for Follow-up Action

Reset

Recommended Practice- Set Reminders for Follow-Up

2.12 The EHR has the capability for clinicians to set reminders for themselves and other responsible clinical staff for future tasks to facilitate test result follow-up.[47,75]

Checklist

Implementation Status

EHR Limitation

Rationale for Practice or Risk Assessment

The EHR can help clinicians follow up with patients regarding test results.[76] Unless they set reminders for themselves, clinicians may forget about follow-up tasks that need to be performed.[41]

Suggested Sources of Input

1. EHR developer
2. Health IT support staff

Strength of Recommendation

Medium

Implementation Guidance

- The EHR has a function to set reminders for follow-up actions due on a future date.[38,77]
- The EHR has a function for reporting of future follow-ups whose due dates have passed without appropriate action.
- The organization has policies for future follow-ups whose due dates have passed without appropriate action.

Assessment Notes

Follow-up Actions

Person Responsible for Follow-up Action

Reset

Recommended Practice- Monitor Test Results Follow-Up

3.1 As part of quality assurance activities, organizations monitor selected practices or indicators[78] related to test result reporting and follow-up. Monitored practices include clinician acknowledgment of test results and clinician follow-up on abnormal test results.[4,5,10,13,37,43,58,79-81]

Checklist

Implementation Status

EHR Limitation

Rationale for Practice or Risk Assessment

Effective quality assurance and patient safety programs include monitoring of core clinical metrics.[82] Errors related to missed or delayed follow-up of test results are a significant cause of adverse events that harm patients.

Assessment Notes

Follow-up Actions

Person Responsible for Follow-up Action

Reset

Suggested Sources of Input

1. Clinicians, support staff, and/or clinical administration
2. EHR developer
3. Health IT support staff

Strength of Recommendation

Strong

Implementation Guidance

- The organization has in place processes to monitor and report notification responses (e.g., acknowledged or not,[35] time to acknowledgment[6]) and test result follow-up with patients.[4]
- Clinicians document communication of test results to patients in the EHR, including whether follow-up is needed, when it should occur, and any other steps recommended.[83]
- Organizational quality assurance activities select and measure test results-related benchmarks for ongoing monitoring, starting in areas of identified concern and high risk.[57] For example, an organization could develop a measurement system for test results reporting and take actions along the following lines:
  - Investigate test results with the lowest follow-up rate to understand the root cause of the problem.[5,81]
  - Determine the percentage of all test results reviewed by the ordering provider within two business days (ambulatory setting) or 12 hours (inpatient setting), or sooner if results are considered more urgent.
  - Determine results not reviewed for more than one week (should be minimal).

Recommended Practice- Monitor Lost Test Results

3.2 As part of quality assurance, the organization monitors and addresses test results sent to the wrong clinician (e.g., via reports from clinicians) or never transmitted to any clinician (e.g., due to an interface problem or patient/ provider misidentification).[37,84]
Checklist

Implementation Status

EHR Limitation

Rationale for Practice or Risk Assessment

When test results are "lost in the system," there is a danger of no follow-up, which poses a significant risk of patient harm.

Suggested Sources of Input

1. Clinicians, support staff, and/or clinical administration
2. Diagnostic services
3. EHR developer
4. Health IT support staff

Strength of Recommendation

Strong

Implementation Guidance

- The organization has policies regarding the frequency of error log monitoring, responsibility for investigating and fixing errors, and how errors are communicated to the ordering clinician or responsible party.
- Error logs are used to detect anomalies such as results that were never delivered, results without any ordering provider, or results with unidentifiable providers.
- National Provider Identification (NPI) numbers are used for provider attribution of orders.
- Monitor provider master files (e.g., address book) to ensure that they are synchronized to avoid scenarios in which the ordering provider's contact information is outdated or unknown.
- Error queues are used to monitor for proper system performance; results that cannot be automatically delivered are manually delivered.

Assessment Notes

Follow-up Actions

Person Responsible for Follow-up Action

Reset

Recommended Practice- Monitor CDS Based on Laboratory Results

3.3 As part of quality assurance, the organization monitors clinical decision support tools that are based on laboratory test results to ensure they are updated when changes are made to the laboratory system or the way laboratory data is recorded.[85,86]

Checklist

Implementation Status

EHR Limitation

Rationale for Practice or Risk Assessment

When test results (or their absence) are used as a criterion in the logic used for clinical decision support, changes to the way laboratory results are recorded may result in CDS malfunctions that could put patients at risk of significant harm.

Assessment Notes

Follow-up Actions

Person Responsible for Follow-up Action

Reset

Suggested Sources of Input

1. Clinicians, support staff, and/or clinical administration
2. Diagnostic services
3. EHR developer
4. Health IT support staff

Strength of Recommendation

Strong

Implementation Guidance

- CDS audit logs are used to generate summaries (e.g., graphs, statistical analyses, etc.) to detect CDS malfunctions that may be associated with changes in the way laboratory results are recorded.[85,87]

Recommended Practice- Monitor Patient Notification

3.4 Organizational policies and procedures ensure timely patient notification of both normal and abnormal test results, and the timeliness of notification is monitored.[88]

Checklist

Implementation Status

EHR Limitation

Rationale for Practice or Risk Assessment

Failure in timely patient notification of test results is a major source of diagnostic error and liability. Standardized policies and procedures for timely patient notification reduce the risk of loss of follow-up.

Suggested Sources of Input

1. Clinicians, support staff, and/or clinical administration
2. Diagnostic services

Strength of Recommendation

Required

Implementation Guidance

- National VA policy "Communicating Test Results to Providers and Patients" Directive 1088[88] states that: "It is VHA policy that all test results must be communicated by the diagnostic provider to the ordering provider, or designee, within a time-frame that allows for prompt attention and appropriate action to be taken. All test results requiring action must be communicated by the ordering provider, or designee, to patients no later than 7 calendar days from the date on which the results are available. For test results that require no action, results must be communicated by the ordering provider, or designee, to patients no later than 14 calendar days from the date on which the results are available. Depending on the clinical context, certain test results may require review and communication in shorter time-frames."
- Notification of test results to patients is monitored for timeliness (i.e., whether the clinician notified the patient within the correct time frame).
- Certain time-sensitive test results, as well as results for which clear, unambiguous communication is essential (e.g., HIV status, cancer diagnosis), are discussed in person or via the telephone rather than using asynchronous electronic means (e.g., secure messaging, voicemail, or patient portals).
- Organizations use patient portals to automatically release test results to patients who have activated their accounts. To explain their test results in more detail, portal users are provided with a link to lab test interpretations (https://medlineplus.gov/lab-tests/).
- For patients who have not activated their online accounts, traditional methods such as letters or phone calls are used to inform them of their results on a timely basis.
- If patient communication and acknowledgment of abnormal results are unable to be confirmed, alternative strategies are used to ensure follow-up (e.g., if the secure message is not read, telephone or send a letter).

Assessment Notes

Follow-up Actions

Person Responsible for Follow-up Action

Reset

References

1. Singh H, Naik AD, Rao R, Petersen LA. Reducing diagnostic errors through effective communication: Harnessing the power of information technology. J Gen Intern Med. 2008;23(4):489-494. pubmed.ncbi.nlm.nih.gov/18373151/. doi: 10.1007/s11606-007-0393-z; PMID: 18373151; PMC2359508.

2. Hickner JM, Fernald DH, Harris DM, Poon EG, Elder NC, Mold JW. Issues and initiatives in the testing process in primary care physician offices. Jt Comm J Qual Patient Saf. 2005;31(2):81-89. pubmed.ncbi.nlm.nih.gov/15791767/. doi: 10.1016/s1553-7250(05)31012-9; PMID: 15791767.

3. Schiff GD. Medical error: A 60-year-old man with delayed care for a renal mass. JAMA. 2011;305(18):1890-1898. https://pubmed.ncbi.nlm.nih.gov/21486963/. doi: 10.1001/jama.2011.496; PMID: 21486963.

4. Singh H, Thomas EJ, Sittig DF, et al. Notification of abnormal lab test results in an electronic medical record: Do any safety concerns remain? Am J Med. 2010;123(3):238-244. https://pubmed.ncbi.nlm.nih.gov/20193832/. doi: 10.1016/j.amjmed.2009.07.027; PMID: 20193832; PMC2878665.

5. Sittig DF, Singh H. Improving test result follow-up through electronic health records requires more than just an alert. J Gen Intern Med. 2012;27(10):1235-1237. https://pubmed.ncbi.nlm.nih.gov/22790618/. doi: 10.1007/s11606-012-2161-y; PMID: 22790618; PMC3445682.

6. Tenner CT, Shapiro NM, Wikler A. Improving health care provider notification in an academic setting: a cascading system of alerts. Arch Intern Med. 2010 Feb 22;170(4):392. doi: 10.1001/archinternmed.2010.9. PMID: 20177047.

7. Singh H, Vij MS. Eight recommendations for policies for communicating abnormal test results. Jt Comm J Qual Patient Saf. 2010;36(5):226-232. https://pubmed.ncbi.nlm.nih.gov/20480756/. doi: 10.1016/s1553-7250(10)36037-5; PMID: 20480756.

8. Singh H, Kadiyala H, Bhagwath G, et al. Using a multifaceted approach to improve the follow-up of positive fecal occult blood test results. Am J Gastroenterol. 2009;104(4):942-952. https://pubmed.ncbi.nlm.nih.gov/19293786/. doi: 10.1038/ajg.2009.55; PMID: 19293786; PMC2921791.

9. Singh H, Wilson L, Reis B, Sawhney MK, Espadas D, Sittig DF. Ten strategies to improve management of abnormal test result alerts in the electronic health record. J Patient Saf. 2010;6(2):121-123. https://www.ncbi.nlm.nih.gov/pmc/articles/PMC2885732/. doi: 10.1097/PTS.0b013e3181ddf652; PMID: 20563228; PMC2885732.

10. Singh H, Thomas EJ, Mani S, et al. Timely follow-up of abnormal diagnostic imaging test results in an outpatient setting; are electronic medical records achieving their potential? Arch Int Med. 2009;169(17):1578-1586. pubmed.ncbi.nlm.nih.gov/19786677/; PMID: 19786677; PMC2919821.

11. Laxmisan A, Sittig DF, Pietz K, Espadas D, Krishnan B, Singh H. Effectiveness of an electronic health record-based intervention to improve follow-up of abnormal pathology results: A retrospective record analysis. Med Care. 2012;50(10):898-904. https://pubmed.ncbi.nlm.nih.gov/22929995/. doi: 10.1097/MLR.0b013e31825f6619; PMID: 22929995; PMC3444625.

12. ECRI Institute PSO. ECRI institute PSO deep dive: Health information technology. www.ecri.org Web site. https://www.ecri.org/components/PSOCore/Documents/Deep%20Dive/Deep%20Dive%20-%20Health%20Information%20Technology%200113.pdf. Updated 2012. Accessed Jul 15, 2024.

13. Callen JL, Westbrook JI, Georgiou A, Li J. Failure to follow-up test results for ambulatory patients: A systematic review. J Gen Intern Med. 2012;27(10):1334-1348. https://pubmed.ncbi.nlm.nih.gov/22183961/. doi: 10.1007/s11606-011-1949-5; PMID: 22183961; PMC3445672.

14. Dalal AK, Poon EG, Karson AS, Gandhi TK, Roy CL. Lessons learned from implementation of a computerized application for pending tests at hospital discharge. J Hosp Med. 2011;6(1):16-21. https://pubmed.ncbi.nlm.nih.gov/21241037/. doi: 10.1002/jhm.794; PMID: 21241037.

References

15. El-Kareh R, Roy C, Williams DH, Poon EG. Impact of automated alerts on follow-up of post-discharge microbiology results: A cluster randomized controlled trial. J Gen Intern Med. 2012;27(10):1243-1250. https://pubmed.ncbi.nlm.nih.gov/22278302/. doi: 10.1007/s11606-012-1986-8; PMID: 22278302; PMC3445692.

16. Elder NC, McEwen TR, Flach J, Gallimore J, Pallerla H. The management of test results in primary care: Does an electronic medical record make a difference? Fam Med. 2010;42(5):327-333. https://pubmed.ncbi.nlm.nih.gov/20455108/; PMID: 20455108.

17. Murphy DR, Laxmisan A, Reis BA, et al. Electronic health record-based triggers to detect potential delays in cancer diagnosis. BMJ Qual Saf. 2014;23(1):8-16. https://pubmed.ncbi.nlm.nih.gov/23873756/. doi: 10.1136/bmjqs-2013-001874; PMID: 23873756.

18. Vreeman DJ, McDonald CJ, Huff SM. LOINC® - A universal catalog of individual clinical observations and uniform representation of enumerated collections. Int J Funct Inform Personal Med. 2010;3(4):273-291. https://pubmed.ncbi.nlm.nih.gov/22899966/. doi: 10.1504/IJFIPM.2010.040211; PMID: 22899966; PMC3418707.

19. Centers for Disease Control and Prevention. Public health information network vocabulary access and distribution system (PHIN VADS). application version 4.0.2. www.phinvads.cdc.gov Web site. https://phinvads.cdc.gov/vads/SearchVocab.action. Updated 2016. Accessed Jul 15, 2024.

20. Burnside ES, Sickles EA, Bassett LW, et al. The ACR BI-RADS® experience: Learning from history. J Am Coll Radiol. 2009;6(12):851-860. https://www.ncbi.nlm.nih.gov/pmc/articles/PMC3099247/. doi: 10.1016/j.jacr.2009.07.023; PMID: 19945040; PMC3099247.

21. Russ G, Bigorgne C, Royer B, Rouxel A, Bienvenu-Perrard M. [The thyroid imaging reporting and data system (TIRADS) for ultrasound of the thyroid]. J Radiol. 2011;92(7-8):701-713. https://pubmed.ncbi.nlm.nih.gov/21819912/. doi: 10.1016/j.jradio.2011.03.022; PMID: 21819912.

22. Kahn CE, Heilbrun ME, Applegate KE. From guidelines to practice: How reporting templates promote the use of radiology practice guidelines. J Am Coll Radiol. 2013;10(4):268-273. https://www.ncbi.nlm.nih.gov/pmc/articles/PMC3615027/. doi: 10.1016/j.jacr.2012.09.025; PMID: 23332496; PMC3615027.

23. Murphy DR, Wu L, Thomas EJ, Forjuoh SN, Meyer AND, Singh H. Electronic trigger-based intervention to reduce delays in diagnostic evaluation for cancer: A cluster randomized controlled trial. J Clin Oncol. 2015;33(31):3560-3567. https://pubmed.ncbi.nlm.nih.gov/26304875/. doi: 10.1200/JCO.2015.61.1301; PMID: 26304875; PMC4622097.

24. Murphy DR, Meyer AND, Bhise V, et al. Computerized triggers of big data to Detect Delays in follow-up of chest Imaging Results. Chest. 2016;150(3):613-620. https://pubmed.ncbi.nlm.nih.gov/27178786/. doi: 10.1016/j.chest.2016.05.001; PMID: 27178786.

25. PenRad HL7 interface specifications. www.penrad.comcastbiz.net Web site. http://www.penrad.comcastbiz.net/pdf/5023B%20Penrad%20HL7%20Interface%20Specs.pdf. Updated 2005. Accessed Jul 15, 2024.

26. Langlotz CP. ACR BI-RADS for breast imaging communication: A roadmap for the rest of radiology. J Am Coll Radiol. 2009;6(12):861-863. https://pubmed.ncbi.nlm.nih.gov/19945041/. doi: 10.1016/j.jacr.2009.09.015; PMID: 19945041.

27. Lung CT screening reporting & data system (lung-RADS®) | american college of radiology. https://www.acr.org/Clinical-Resources/Reporting-and-Data-Systems/Lung-Rads. Accessed Jul 22, 2024.

References

28. Tessler FN, Middleton WD, Grant EG, et al. ACR thyroid imaging, reporting and data system (TI-RADS): White paper of the ACR TI-RADS committee. J Am Coll Radiol. 2017;14(5):587-595. https://pubmed.ncbi.nlm.nih.gov/28372962/. doi: 10.1016/j.jacr.2017.01.046; PMID: 28372962.

29. Schreiber R, Sittig DF, Ash J, Wright A. Orders on file but no labs drawn: Investigation of machine and human errors caused by an interface idiosyncrasy. J Am Med Inform Assoc. 2017;24(5):958-963. https://pubmed.ncbi.nlm.nih.gov/28339629/. doi: 10.1093/jamia/ocw188; PMID: 28339629; PMC6080845.

30. ECRI Institute. Closing the loop: Using health IT to mitigate delayed, missed, and incorrect diagnoses related to diagnostic testing and medication changes. . www.ecri.org Web site. https://www.ecri.org/Resources/HIT/Closing_Loop/Closing_the_Loop_Toolkit.pdf. Updated 2018. Accessed Jul 15, 2024.

31. Dalal AK, Pesterev BM, Eibensteiner K, Newmark LP, Samal L, Rothschild JM. Linking acknowledgement to action: Closing the loop on non-urgent, clinically significant test results in the electronic health record. J Am Med Inform Assoc. 2015;22(4):905-908. https://pubmed.ncbi.nlm.nih.gov/25796594/. doi: 10.1093/jamia/ocv007; PMID: 25796594; PMC6283058.

32. Murphy DR, Singh H, Berlin L. Communication breakdowns and diagnostic errors: A radiology perspective. Diagnosis (Berl). 2014;1(4):253-261. https://pubmed.ncbi.nlm.nih.gov/27006890/. doi: 10.1515/dx-2014-0035; PMID: 27006890; PMC4799783.

33. Lacson R, Prevedello LM, Andriole KP, et al. Four-year impact of an alert notification system on closed-loop communication of critical test results. AJR Am J Roentgenol. 2014;203(5):933-938. https://pubmed.ncbi.nlm.nih.gov/25341129/. doi: 10.2214/AJR.14.13064; PMID: 25341129; PMC4426858.

34. Litchfield I, Bentham L, Lilford R, McManus RJ, Hill A, Greenfield S. Test result communication in primary care: A survey of current practice. BMJ Qual Saf. 2015;24(11):691-699. https://pubmed.ncbi.nlm.nih.gov/26243888/. doi: 10.1136/bmjqs-2014-003712; PMID: 26243888; PMC4680128.

35. O'Connor SD, Dalal AK, Sahni VA, Lacson R, Khorasani R. Does integrating nonurgent, clinically significant radiology alerts within the electronic health record impact closed-loop communication and follow-up? J Am Med Inform Assoc. 2016;23(2):333-338. https://pubmed.ncbi.nlm.nih.gov/26335982/. doi: 10.1093/jamia/ocv105; PMID: 26335982; PMC5009922.

36. Weiss DL, Kim W, Branstetter BF, Prevedello LM. Radiology reporting: A closed-loop cycle from order entry to results communication. J Am Coll Radiol. 2014;11(12 Pt B):1226-1237. https://pubmed.ncbi.nlm.nih.gov/25467899/. doi: 10.1016/j.jacr.2014.09.009; PMID: 25467899.

37. Roy CL, Rothschild JM, Dighe AS, et al. An initiative to improve the management of clinically significant test results in a large health care network. Jt Comm J Qual Patient Saf. 2013;39(11):517-527. https://pubmed.ncbi.nlm.nih.gov/24294680/. doi: 10.1016/s1553-7250(13)39068-0; PMID: 24294680.

38. Sloan CE, Chadalavada SC, Cook TS, Langlotz CP, Schnall MD, Zafar HM. Assessment of follow-up completeness and notification preferences for imaging findings of possible cancer: What happens after radiologists submit their reports? Acad Radiol. 2014;21(12):1579-1586. https://pubmed.ncbi.nlm.nih.gov/25179562/. doi: 10.1016/j.acra.2014.07.006; PMID: 25179562; PMC4825815.

39. Cole B, Dickerson JA, Graber ML, et al. A prospective tool for risk assessment of sendout testing. Clin Chim Acta. 2014;434:1-5. https://pubmed.ncbi.nlm.nih.gov/24685573/. doi: 10.1016/j.cca.2014.03.028; PMID: 24685573.

References

40. Barry C, Edmonston TB, Gandhi S, Ganti K, Kim N, Bierl C. Implementation of laboratory review of test builds within the electronic health record reduces errors. Arch Pathol Lab Med. 2020;144(6):742-747. https://pubmed.ncbi.nlm.nih.gov/31647317/. doi: 10.5858/arpa.2019-0239-OA; PMID: 31647317.

41. Al-Mutairi A, Meyer AND, Chang P, Singh H. Lack of timely follow-up of abnormal imaging results and radiologists' recommendations. J Am Coll Radiol. 2015;12(4):385-389. https://pubmed.ncbi.nlm.nih.gov/25582812/. doi: 10.1016/j.jacr.2014.09.031; PMID: 25582812.

42. Meyer AND, Murphy DR, Singh H. Communicating findings of delayed diagnostic evaluation to primary care providers. J Am Board Fam Med. 2016;29(4):469-473. https://pubmed.ncbi.nlm.nih.gov/27390378/. doi: 10.3122/jabfm.2016.04.150363; PMID: 27390378.

43. Poon EG, Wang SJ, Gandhi TK, Bates DW, Kuperman GJ. Design and implementation of a comprehensive outpatient results manager. J Biomed Inform. 2003;36(1-2):80-91. https://pubmed.ncbi.nlm.nih.gov/14552849/. doi: 10.1016/s1532-0464(03)00061-3; PMID: 14552849.

44. Mohta VJ. Amended lab results: Communication slip. www.psnet.ahrq.gov Web site. https://psnet.ahrq.gov/web-mm/amended-lab-results-communication-slip. Updated 2012. Accessed Jul 15, 2024.

45. Liberatore K. Beyond the lab: The link between health IT and laboratory test problems. Pa Patient Saf Advis. 2018(Suppl 1):16-24. https://patientsafety.pa.gov/ADVISORIES/Pages/201810_HealthITandLaboratory.aspx#.

46. Dalal AK, Schnipper JL, Poon EG, et al. Design and implementation of an automated email notification system for results of tests pending at discharge. J Am Med Inform Assoc. 2012;19(4):523-528. https://pubmed.ncbi.nlm.nih.gov/22268214/. doi: 10.1136/amiajnl-2011-000615; PMID: 22268214; PMC3384118.

47. Hysong SJ, Sawhney MK, Wilson L, et al. Understanding the management of electronic test result notifications in the outpatient setting. BMC Med Inform Decis Mak. 2011;11:22. https://pubmed.ncbi.nlm.nih.gov/21486478/. doi: 10.1186/1472-6947-11-22; PMID: 21486478; PMC3100236.

48. Litchfield IJ, Bentham LM, Lilford RJ, Greenfield SM. Test result communication in primary care: Clinical and office staff perspectives. Fam Pract. 2014;31(5):592-597. https://pubmed.ncbi.nlm.nih.gov/25070182/. doi: 10.1093/fampra/cmu041; PMID: 25070182; PMC4169669.

49. Powell L, Sittig DF, Chrouser K, Singh H. Assessment of health information Technology–Related outpatient diagnostic delays in the US veterans affairs health care system. JAMA Netw Open. 2020;3(6):e206752. https://www.ncbi.nlm.nih.gov/pmc/articles/PMC7317596/. doi: 10.1001/jamanetworkopen.2020.6752; PMID: 32584406; PMC7317596.

50. Kwan JL, Singh H. Assigning responsibility to close the loop on radiology test results. Diagnosis (Berl). 2017;4(3):173-177. https://www.ncbi.nlm.nih.gov/pmc/articles/PMC5673267/. doi: 10.1515/dx-2017-0019; PMID: 29119073; PMC5673267.

51. Department of Veteran Affairs. VHA directive 1088: Communicating test results to providers and patients. www.va.gov Web site. https://www.va.gov/vhapublications/publications.cfm?pub=1&order=desc&orderby=pub_Number. Updated 2023. Accessed Jul 15, 2024.

52. Atlas SJ, Tosteson ANA, Wright A, et al. A multilevel primary care intervention to improve follow-up of overdue abnormal cancer screening test results: A cluster randomized clinical trial. JAMA. 2023;330(14):1348-1358. https://pubmed.ncbi.nlm.nih.gov/37815566/. doi: 10.1001/jama.2023.18755; PMID: 37815566; PMC10565610.

53. Roy CL, Poon EG, Karson AS, et al. Patient safety concerns arising from test results that return after hospital discharge. Ann Intern Med. 2005;143(2):121-128. https://pubmed.ncbi.nlm.nih.gov/16027454/. doi: 10.7326/0003-4819-143-2-200507190-00011; PMID: 16027454.

References

54. Zimolzak AJ, Shahid U, Giardina TD, et al. Why test results are still getting "lost" to follow-up: A qualitative study of implementation gaps. J Gen Intern Med. 2022;37(1):137-144. https://pubmed.ncbi.nlm.nih.gov/33907982/. doi: 10.1007/s11606-021-06772-y; PMID: 33907982; PMC8739406.

55. Beckwith BA, Aller RD, Brassel JH, Brodsky VB, de Baca ME. Laboratory interoperability best practices: Ten mistakes to avoid. www.cap.org Web site. https://uatcap.objects.frb.io/documents/laboratory-interoperability-best-practices.pdf. Updated 2013. Accessed Jul 15, 2024.

56. The Joint Commission. National patient safety goals® effective january 2021 for the hospital program. www.jointcommission.org Web site. https://www.jointcommission.org/-/media/tjc/documents/standards/national-patient-safety-goals/2021/npsg_chapter_hap_jan2021.pdf. Updated 2021. Accessed Jul 15, 2024.

57. Bowie P, Price J, Hepworth N, Dinwoodie M, McKay J. System hazards in managing laboratory test requests and results in primary care: Medical protection database analysis and conceptual model. BMJ Open. 2015;5(11):e008968. https://pubmed.ncbi.nlm.nih.gov/26614621/. doi: 10.1136/bmjopen-2015-008968; PMID: 26614621; PMC4663465.

58. Menon S, Smith MW, Sittig DF, et al. How context affects electronic health record-based test result follow-up: A mixed-methods evaluation. BMJ Open. 2014;4(11):e005985. https://pubmed.ncbi.nlm.nih.gov/25387758/. doi: 10.1136/bmjopen-2014-005985; PMID: 25387758; PMC4244393.

59. Singh R, Hickner J, Mold J, Singh G. "Chance favors only the prepared mind": Preparing minds to systematically reduce hazards in the testing process in primary care. J Patient Saf. 2014;10(1):20-28. https://pubmed.ncbi.nlm.nih.gov/24553441/. doi: 10.1097/PTS.0b013e3182a5f81a; PMID: 24553441.

60. Dalal AK, Roy CL, Poon EG, et al. Impact of an automated email notification system for results of tests pending at discharge: A cluster-randomized controlled trial. J Am Med Inform Assoc. 2014;21(3):473-480. https://pubmed.ncbi.nlm.nih.gov/24154834/. doi: 10.1136/amiajnl-2013-002030; PMID: 24154834; PMC3994865.

61. Ong M, Magrabi F, Jones G, Coiera E. Last orders: Follow-up of tests ordered on the day of hospital discharge. Arch Intern Med. 2012;172(17):1347-1349. https://pubmed.ncbi.nlm.nih.gov/22892677/. doi: 10.1001/archinternmed.2012.2836; PMID: 22892677.

62. Lynn TJ, Olson JE. Improving critical value notification through secure text messaging. J Pathol Inform. 2020;11:21. pubmed.ncbi.nlm.nih.gov/33042600/. doi: 10.4103/jpi.jpi_19_20; PMID: 33042600; PMC7518196.

63. Lacson R, O'Connor SD, Sahni VA, Roy C, Dalal A, Desai S, Khorasani R. Impact of an electronic alert notification system embedded in radiologists' workflow on closed-loop communication of critical results: a time series analysis. BMJ Qual Saf. 2016 Jul;25(7):518-24. doi: 10.1136/bmjqs-2015-004276. Epub 2015 Sep 15. PMID: 26374896.

64. Kuperman GJ, Teich JM, Bates DW, et al. Detecting alerts, notifying the physician, and offering action items: A comprehensive alerting system. Proc AMIA Annu Fall Symp. 1996:704-708. https://pubmed.ncbi.nlm.nih.gov/8947756/; PMID: 8947756; PMC2233134.

65. Cadamuro J, Hillarp A, Unger A, et al. Presentation and formatting of laboratory results: A narrative review on behalf of the european federation of clinical chemistry and laboratory medicine (EFLM) working group "postanalytical phase" (WG-POST). Crit Rev Clin Lab Sci. 2021;58(5):329-353. pubmed.ncbi.nlm.nih.gov/33538219/. doi: 10.1080/10408363.2020.1867051; PMID: 33538219.

66. Horsky J, Kuperman GJ, Patel VL. Comprehensive analysis of a medication dosing error related to CPOE. J Am Med Inform Assoc. 2005;12(4):377-382. https://pubmed.ncbi.nlm.nih.gov/15802485/. doi: 10.1197/jamia.M1740; PMID: 15802485; PMC1174881.

References

67. Singh H, Spitzmueller C, Petersen NJ, Sawhney MK, Sittig DF&. Information overload and missed test results in EHR-based settings. JAMA Intern Med. 2013;173(8). https://www.ncbi.nlm.nih.gov/pmc/articles/PMC3822526/. doi: 10.1001/2013.jamainternmed.61; PMID: 23460235; PMC3822526.

68. DeSimone AK, Kapoor N, Lacson R, et al. Impact of an automated closed-loop communication and tracking tool on the rate of recommendations for additional imaging in thoracic radiology reports. J Am Coll Radiol. 2023;20(8):781-788. https://pubmed.ncbi.nlm.nih.gov/37307897/. doi: 10.1016/j.jacr.2023.05.004; PMID: 37307897.

69. Desai S, Kapoor N, Hammer MM, et al. RADAR: A closed-loop quality improvement initiative leveraging A safety net model for incidental pulmonary nodule management. Jt Comm J Qual Patient Saf. 2021;47(5):275-281. https://pubmed.ncbi.nlm.nih.gov/33478839/. doi: 10.1016/j.jcjq.2020.12.006; PMID: 33478839.

70. Sittig DF, Murphy DR, Smith MW, Russo E, Wright A, Singh H. Graphical display of diagnostic test results in electronic health records: A comparison of 8 systems. J Am Med Inform Assoc. 2015;22(4):900-904. https://pubmed.ncbi.nlm.nih.gov/25792704/. doi: 10.1093/jamia/ocv013; PMID: 25792704; PMC4482275.

71. Nystrom DT, Singh H, Baldwin J, Sittig DF, Giardina TD. Methods for patient-centered interface design of test result display in online portals. EGEMS (Wash DC). 2018;6(1):15. pubmed.ncbi.nlm.nih.gov/30094287/. doi: 10.5334/egems.255; PMID: 30094287; PMC6078112.

72. Shneiderman B. The eyes have it: A task by data type taxonomy for information visualizations. In:The craft of information visualization. San Francisco: Morgan Kaufmann; 2003:364-371. https://www.sciencedirect.com/science/article/pii/B9781558609150500469. 10.1016/B978-155860915-0/50046-9.

73. Zikmund-Fisher BJ, Scherer AM, Witteman HO, et al. Graphics help patients distinguish between urgent and non-urgent deviations in laboratory test results. J Am Med Inform Assoc. 2017;24(3):520-528. pubmed.ncbi.nlm.nih.gov/28040686/. doi: 10.1093/jamia/ocw169; PMID: 28040686; PMC5565988.

74. Woods DD, Patterson ES, Roth EM, Christoffersen K. Can we ever escape from data overload? A cognitive systems diagnosis. 1999;43(3):174-178. https://journals.sagepub.com/doi/10.1177/154193129904300310. doi: 10.1177/154193129904300310.

75. Poon EG, Kuperman GJ, Fiskio J, Bates DW. Real-time notification of laboratory data requested by users through alphanumeric pagers. J Am Med Inform Assoc. 2002;9(3):217-222. https://pubmed.ncbi.nlm.nih.gov/11971882/. doi: 10.1197/jamia.m1009; PMID: 11971882; PMC344581.

76. Smith M, Murphy D, Laxmisan A, et al. Developing software to “Track and catch” missed follow-up of abnormal test results in a complex sociotechnical environment. Appl Clin Inform. 2013;4(3):359-375. https://www.ncbi.nlm.nih.gov/pmc/articles/PMC3799004/. doi: 10.4338/ACI-2013-04-RA-0019; PMID: 24155789; PMC3799004.

77. Thomas J, Dahm MR, Li J, et al. Variation in electronic test results management and its implications for patient safety: A multisite investigation. J.Am.Med.Inform.Assoc. 2020;27(8):1214-1224. https://pubmed.ncbi.nlm.nih.gov/32719839/. doi: 10.1093/jamia/ocaa093; PMID: 32719839; PMC7481032.

78. Murphy DR, Zimolzak AJ, Upadhyay DK, et al. Developing electronic clinical quality measures to assess the cancer diagnostic process. J Am Med Inform Assoc. 2023;30(9):1526-1531. https://pubmed.ncbi.nlm.nih.gov/37257883/. doi: 10.1093/jamia/ocad089; PMID: 37257883; PMC10436145.

79. Boohaker EA, Ward RE, Uman JE, McCarthy BD. Patient notification and follow-up of abnormal test results. A physician survey. Arch Intern Med. 1996;156(3):327-331. https://pubmed.ncbi.nlm.nih.gov/8572844/; PMID: 8572844.

References

80. Greenes DS, Fleisher GR, Kohane I. Potential impact of a computerized system to report late-arriving laboratory results in the emergency department. Pediatr Emerg Care. 2000;16(5):313-315. https://pubmed.ncbi.nlm.nih.gov/11063357/. doi: 10.1097/00006565-200010000-00002; PMID: 11063357.

81. Singh H, Wilson L, Petersen LA, et al. Improving follow-up of abnormal cancer screens using electronic health records: Trust but verify test result communication. BMC Med Inform Decis Mak. 2009;9:49. https://pubmed.ncbi.nlm.nih.gov/20003236/. doi: 10.1186/1472-6947-9-49; PMID: 20003236; PMC2797509.

82. Epner PL, Gans JE, Graber ML. When diagnostic testing leads to harm: A new outcomes-based approach for laboratory medicine. BMJ Qual Saf. 2013;22 Suppl 2(Suppl 2):ii6-ii10. https://pubmed.ncbi.nlm.nih.gov/23955467/. doi: 10.1136/bmjqs-2012-001621; PMID: 23955467; PMC3786651.

83. Veterans Health Administration. VHA directive 2009-019: Ordering and reporting test results. www.va.gov Web site. Updated 2009. Accessed Jul 15, 2024.

84. Yackel TR, Embi PJ. Unintended errors with EHR-based result management: A case series. J Am Med Inform Assoc. 2010;17(1):104-107. https://pubmed.ncbi.nlm.nih.gov/20064810/. doi: 10.1197/jamia.M3294; PMID: 20064810; PMC2995631.

85. Liu S, Wright A, Hauskrecht M. Change-point detection method for clinical decision support system rule monitoring. Artif Intell Med. 2018;91:49-56. https://www.ncbi.nlm.nih.gov/pmc/articles/PMC7416776/. doi: 10.1016/j.artmed.2018.06.003; PMID: 30041919; PMC7416776.

86. Wright A, Hickman TT, McEvoy D, et al. Analysis of clinical decision support system malfunctions: A case series and survey. J Am Med Inform Assoc. 2016;23(6):1068-1076. https://pubmed.ncbi.nlm.nih.gov/27026616/. doi: 10.1093/jamia/ocw005; PMID: 27026616; PMC5070518.

87. Raebel MA, Haynes K, Woodworth TS, et al. Electronic clinical laboratory test results data tables: Lessons from mini-sentinel. Pharmacoepidemiol Drug Saf. 2014;23(6):609-618. https://pubmed.ncbi.nlm.nih.gov/24677577/. doi: 10.1002/pds.3580; PMID: 24677577.

88. Department of Veteran Affairs. VHA directive 1088: Communicating test results to providers and patients. www.va.gov Web site. https://www.va.gov/vhapublications/ViewPublication.asp?pub_ID=10366. Updated 2023. Accessed Jul 15, 2024.
